# 37th International Symposium on Intensive Care and Emergency Medicine (part 3 of 3): Brussels, Belgium. 21-24 March 2017

**DOI:** 10.1186/s13054-017-1629-x

**Published:** 2017-03-21

**Authors:** M. Von Seth, L. Hillered, A. Otterbeck, K. Hanslin, A. Larsson, J. Sjölin, M. Lipcsey, ME Cove, N. S. Chew, L. H. Vu, R. Z. Lim, Z. Puthucheary, K. Hanslin, F. Wilske, P. Skorup, E. Tano, J. Sjölin, M. Lipcsey, I. Derese, S. Thiessen, S. Derde, T. Dufour, L. Pauwels, Y. Bekhuis, G. Van den Berghe, I. Vanhorebeek, M. Khan, D. Dwivedi, J. Zhou, A. Prat, N. G. Seidah, P. C. Liaw, A. E. Fox-Robichaud, M. Von Seth, P. Skorup, L. Hillered, A. Larsson, J. Sjölin, M. Lipcsey, A. Otterbeck, K. Hanslin, M. Lipcsey, A. Larsson, M. Von Seth, T. Correa, J Pereira, J Takala, S Jakob, P. Skorup, L. Maudsdotter, E. Tano, M. Lipcsey, M. Castegren, A. Larsson, J Sjölin, M. Xue, J. Y. Xu, L. Liu, Y. Z. Huang, F. M. Guo, Y. Yang, H. B. Qiu, A. Kuzovlev, V. Moroz, A. Goloubev, A. Myazin, A. Chumachenko, V. Pisarev, N. Takeyama, M. Tsuda, H. Kanou, R. Aoki, Y. Kajita, M. Hashiba, T. Terashima, A. Tomino, R. Davies, K. P. O’Dea, S. Soni, J. K. Ward, D. J. O’Callaghan, M. Takata, A. C. Gordon, J. Wilson, Y. Zhao, M. Singer, J. Spencer, M. Shankar-Hari, K. Roveran Genga, C. Lo, M. S. Cirstea, K. R. Walley, J. A. Russell, A. Linder, J. H. Boyd, A. Sedlag, C. Riedel, M. Georgieff, E. Barth, H. Bracht, A. Essig, D. Henne-Bruns, F. Gebhard, K. Orend, M. Halatsch, M. Weiss, M. Chase, E. Freinkman, A. Uber, X. Liu, M. N. Cocchi, M. W. Donnino, M. Peetermans, L. Liesenborghs, J. Claes, T. Vanassche, M. Hoylaerts, M. Jacquemin, K. Vanhoorelbeke, S. De Meyer, P. Verhamme, A. Vögeli, M. Ottiger, M. Meier, C. Steuer, L. Bernasconi, A. Huber, M. Christ-Crain, C. Henzen, C. Hoess, R. Thomann, W. Zimmerli, B. Müller, P. Schütz, D. Hoppensteadt, A. Walborn, M. Rondina, K. Tsuruta, J. Fareed, S. Tachyla, T. Ikeda, S. Ono, T. Ueno, S. Suda, T. Nagura, E. Damiani, R. Domizi, C. Scorcella, S. Tondi, S. Pierantozzi, S. Ciucani, N. Mininno, E. Adrario, P. Pelaia, A. Donati, M. Schou Andersen, S. Lu, G Lopez, AT Lassen, I. Ghiran, N. I. Shapiro, U. Trahtemberg, S. Sviri, M. Beil, Z. Agur, P. Van Heerden, E. Jahaj, A. Vassiliou, Z. Mastora, S. E. Orfanos, A. Kotanidou, Y. Wirz, R. Sager, D. Amin, A. Amin, S. Haubitz, P. Hausfater, A. Huber, A. Kutz, B. Mueller, P. Schuetz, R. S. Sager, Y. W. Wirz, D. A. Amin, A. A. Amin, P. H. Hausfater, A. H. Huber, S. Haubitz, A. Kutz, B Mueller, P Schuetz, L. Gottin, C. Dell’amore, G. Stringari, G. Cogo, M. Ceolagraziadei, M. Sommavilla, F. Soldani, E. Polati, M. Meier, T. Baumgartner, G. Zurauskaité, S. Gupta, B. Mueller, A. Devendra, P. Schuetz, D. Mandaci, G. Eren, F. Ozturk, N. Emir, O. Hergunsel, S. Azaiez, S. Khedher, A. Maaoui, M. Salem, E. Chernevskaya, N. Beloborodova, A. Bedova, Y. U. Sarshor, A. Pautova, V. Gusarov, N. Öveges, I. László, M. Forgács, T. Kiss, P. Hankovszky, P. Palágyi, A. Bebes, B. Gubán, I. Földesi, Á. Araczki, M. Telkes, Z. Ondrik, Z. Helyes, Á. Kemény, Z. Molnár, E. Spanuth, H. Ebelt, B. Ivandic, R. Thomae, K. Werdan, M. El-Shafie, K. Taema, M. El-Hallag, A. Kandeel, O. Tayeh, K. Taema, M. Eldesouky, A. Omara, M. S. Winkler, M. Holzmann, A. Nierhaus, E. Mudersbach, E. Schwedhelm, G. Daum, S. Kluge, C. Zoellner, G. Greiwe, H. Sawari, E. Schwedhelm, A. Nierhaus, S. Kluge, J. Kubitz, R. Jung, G. Daum, H. Reichenspurner, C. Zoellner, M. S. Winkler, M. Groznik, A. Ihan, L. W. Andersen, M. Chase, M. J. Holmberg, A. Wulff, M. N. Cocchi, M. W. Donnino, C. Balci, M. Haliloglu, B. Bilgili, H. Bilgin, U. Kasapoglu, I. Sayan, M. Süzer, L. Mulazımoglu, I. Cinel, V. Patel, S. Shah, P. Parulekar, C. Minton, J. Patel, C. Ejimofo, H. Choi, R. Costa, P. Caruso, P. Nassar, J. Fu, J. Jin, Y. Xu, J. Kong, D. Wu, A. Yaguchi, A. Klonis, S. Ganguly, M. Kollef, C. Burnham, B. Fuller, A. Mavrommati, D. Chatzilia, E. Salla, E. Papadaki, S. Kamariotis, S. Christodoulatos, A. Stylianakis, G. Alamanos, M. Simoes, E. Trigo, N. Silva, P. Martins, J. Pimentel, D. Baily, L. A. Curran, E. Ahmadnia, B. V. Patel, D. Adukauskiene, J Cyziute, A. Adukauskaite, D. Pentiokiniene, F. Righetti, E. Colombaroli, G. Castellano, F. Wilske, P. Skorup, M. Lipcsey, K. Hanslin, A. Larsson, J. Sjölin, M. Man, H. P. Shum, Y. H. Chan, K. C. Chan, W. W. Yan, R. A. Lee, S. K. Lau, P. Dilokpattanamongkol, P. Thirapakpoomanunt, R. Anakkamaetee, P. Montakantikul, V. Tangsujaritvijit, S. Sinha, J. Pati, S. Sahu, D. Adukauskiene, D. Valanciene, A. Dambrauskiene, D. Adukauskiene, D. Valanciene, A. Dambrauskiene, K. Hernandez, T. Lopez, D. Saca, M. Bello, W. Mahmood, K. Hamed, N. Al Badi, S. AlThawadi, S. Al Hosaini, N. Salahuddin, C. C. Cilloniz, A. C. Ceccato, G. L. Li Bassi, M. F. Ferrer, A. G. Gabarrus, O. R. Ranzani, A. S. San Jose, C. G. Garcia Vidal, J. P. Puig de la Bella Casa, F. B. Blasi, AT Torres, D. Adukauskiene, A. Ciginskiene, A. Dambrauskiene, R. Simoliuniene, G. Giuliano, D. Triunfio, E. Sozio, E. Taddei, E. Brogi, F. Sbrana, A. Ripoli, G. Bertolino, C. Tascini, F. Forfori, C. Fleischmann, D. Goldfarb, P. Schlattmann, L. Schlapbach, N. Kissoon, N. Baykara, H. Akalin, M. Kemal Arslantas, And Sepsis Study Group, S. G. Gavrilovic, M. V. Vukoja, M. H. Hache, R. K. Kashyap, Y. D. Dong, O. G. Gajic, O. Ranzani, M. Shankar-Hari, D. Harrison, L. Rabello, K. Rowan, J. Salluh, M. Soares, A. M. Markota, J. F. Fluher, D. K. Kogler, Z. B. Borovšak, A. S. Sinkovic, I. László, N. Öveges, M. Forgács, T. Kiss, P. Hankovszky, P. Palágyi, A. Bebes, B. Gubán, I. Földesi, Á. Araczki, M. Telkes, Z. Ondrik, Z. Helyes, Á. Kemény, Z. Molnár, J. Fareed, Z Siddiqui, P. Aggarwal, O. Iqbal, D. Hoppensteadt, M. Lewis, R. Wasmund, S. Abro, S. Raghuvir, K. Tsuruta, P. S. Barie, D. Fineberg, A. Radford, K. Tsuruta, A. Casazza, A. Vilardo, E. Bellazzi, R. Boschi, D. Ciprandi, C. Gigliuto, R. Preda, R. Vanzino, M. Vetere, L. Carnevale, E. Kyriazopoulou, A. Pistiki, C. Routsi, I. Tsangaris, E. Giamarellos-Bourboulis, E. Kyriazopoulou, I. Tsangaris, C. Routsi, I. Pnevmatikos, G. Vlachogiannis, E. Antoniadou, K. Mandragos, A. Armaganidis, E. Giamarellos-Bourboulis, P. Allan, R. Oehmen, J. Luo, C. Ellis, P. Latham, J. Newman, C. Pritchett, D. Pandya, A. Cripps, S. Harris, M. Jadav, R. Langford, B. Ko, H. Park, C. M. Beumer, R. Koch, D. V. Beuningen, A. M. Oudelashof, F. L. Vd Veerdonk, E. Kolwijck, J. G. VanderHoeven, D. C. Bergmans, C. Hoedemaekers, J. B. Brandt, J. Golej, G. Burda, G. Mostafa, A. Schneider, R. Vargha, M. Hermon, P. Levin, C Broyer, M. Assous, Y. Wiener-Well, M. Dahan, S. Benenson, E Ben-Chetrit, A. Faux, R. Sherazi, A. Sethi, S. Saha, M. Kiselevskiy, E. Gromova, S. Loginov, I. Tchikileva, Y. Dolzhikova, N. Krotenko, R. Vlasenko, N. Anisimova, S. Spadaro, A. Fogagnolo, F. Remelli, V. Alvisi, A. Romanello, E. Marangoni, C. Volta, A. Degrassi, F. Mearelli, C. Casarsa, N. Fiotti, G. Biolo, M. Cariqueo, C. Luengo, R. Galvez, C. Romero, R. Cornejo, O. Llanos, N. Estuardo, P. Alarcon, B. Magazi, S. Khan, J. Pasipanodya, M. Eriksson, G. Strandberg, M. Lipsey, A. Larsson, Z. Rajput, F. Hiscock, T. Karadag, J. Uwagwu, S. Jain, A. Molokhia, H. Barrasa, A. Soraluce, E. Uson, A. Rodriguez, A. Isla, A. Martin, B. Fernández, F. Fonseca, J. A. Sánchez-Izquierdo, F. J. Maynar, M. Kaffarnik, R. Alraish, O. Frey, A. Roehr, M. Stockmann, S. Wicha, D. Shortridge, M. Castanheira, H. S. Sader, J. M. Streit, R. K. Flamm, K. Falsetta, T. Lam, S. Reidt, J. Jancik, T. Kinoshita, J. Yoshimura, K. Yamakawa, S. Fujimi, A. Armaganidis, A. Torres, S. Zakynthinos, C. Mandragos, E. Giamarellos-Bourboulis, P. Ramirez, M. De la Torre-Prados, A. Rodriguez, G. Dale, A. Wach, L. Beni, L. Hooftman, C. Zwingelstein, B. François, G. Colin, P. F. Dequin, P. F. Laterre, A. Perez, R. Welte, I. Lorenz, P. Eller, M. Joannidis, R. Bellmann, S. Lim, S. Chana, S. Patel, J. Higuera, D. Cabestrero, L. Rey, G. Narváez, A. Blandino, M. Aroca, S. Saéz, R De Pablo, S. Thiessen, I. Vanhorebeek, S. Derde, I. Derese, T. Dufour, C. Nadège Albert, L. Langouche, C. Goossens, N. Peersman, P. Vermeersch, S. Vander Perre, J. Holst, P. Wouters, G. Van den Berghe, X. Liu, A. U. Uber, M. Holmberg, V. Konanki, M. McNaughton, J. Zhang, M. W. Donnino, O. Demirkiran, A. Byelyalov, C. Luengo, J. Guerrero, M Cariqueo, C. Scorcella, R. Domizi, E. Damiani, S. Tondi, S. Pierantozzi, N. Rossini, U. Falanga, V. Monaldi, E. Adrario, P. Pelaia, A. Donati, O. Cole, N. Scawn, M. Balciunas, I. Blascovics, A. Vuylsteke, K. Salaunkey, A. Omar, A. Salama, M. Allam, A. Alkhulaifi, S. Verstraete, I. Vanhorebeek, E. Van Puffelen, I. Derese, C. Ingels, S. Verbruggen, P. Wouters, K. Joosten, J. Hanot, G. Guerra, D. Vlasselaers, J. Lin, G. Van den Berghe, R. Haines, P. Zolfaghari, R. Hewson, C. Offiah, J. Prowle, H. Park, B. Ko, H. Buter, J. A. Veenstra, M. Koopmans, E. C. Boerma, J. A. Veenstra, H. Buter, M. Koopmans, E. C. Boerma, A. Taha, A. Shafie, S. Hallaj, D. Gharaibeh, H. Hon, M. Bizrane, A. A. El Khattate, N. Madani, R. Abouqal, J. Belayachi, N. Kongpolprom, N. Sanguanwong, S. Sanaie, A. Mahmoodpoor, H. Hamishehkar, P. Biderman, P. Van Heerden, Y. Avitzur, S. Solomon, Z. Iakobishvili, U. Carmi, D Gorfil, P. Singer, C. Paisley, J. Patrick-Heselton, M. Mogk, J. Humphreys, I. Welters, S. Pierantozzi, C. Scorcella, R. Domizi, E. Damiani, S. Tondi, E. Casarotta, S. Bolognini, E. Adrario, P. Pelaia, A. Donati, M. J. Holmberg, A. Moskowitz, P. Patel, A. Grossestreuer, A. Uber, L. W. Andersen, M. W. Donnino, S. Malinverni, D. Goedeme, P. Mols, P. L. Langlois, C. Szwec, F. D’Aragon, D. K. Heyland, W. Manzanares, W. Manzanares, C. Szwec, P. Langlois, I. Aramendi, D. Heyland, N. Stankovic, J. Nadler, A. Uber, M. Holmberg, L. Sanchez, R. Wolfe, M. Chase, M. Donnino, M. Cocchi, H. K. Atalan, B. Gucyetmez, M. E. Kavlak, S. Aslan, A. Kargi, S. Yazici, R. Donmez, K. Y. Polat, M Piechota, A. Piechota, M. Misztal, S. Bernas, I. Pietraszek-Grzywaczewska, M. Saleh, A. Hamdy, A. Hamdy, M. Elhallag, F. Atar, A. Kundakci, E. Gedik, H. Sahinturk, P. Zeyneloglu, A. Pirat, M. Popescu, D. Tomescu, R. Van Gassel, M. Baggerman, F. Schaap, M. Bol, G. Nicolaes, D. Beurskens, S. Olde Damink, M. Van de Poll, M. Horibe, M. Sasaki, M. Sanui, E. Iwasaki, H. Sawano, T. Goto, T. Ikeura, T. Hamada, T. Oda, T. Mayumi, T. Kanai, G. Kjøsen, R. Horneland, K. Rydenfelt, E. Aandahl, T. Tønnessen, H. Haugaa, P. Lockett, L. Evans, L. Somerset, F. Ker-Reid, S. Laver, E. Courtney, S. Dalton, A. Georgiou, K. Robinson, T. Lam, B. Haas, S. Reidt, K. Bartlett, J. Jancik, M. Bigwood, R. Hanley, P. Morgan, D. Marouli, A. Chatzimichali, S. Kolyvaki, A. Panteli, E. Diamantaki, E. Pediaditis, P. Sirogianni, P. Ginos, E. Kondili, D. Georgopoulos, H. Askitopoulou, F. G. Zampieri, A. B. Liborio, B. A. Besen, A. B. Cavalcanti, C. Dominedò, A. M. Dell’Anna, A. Monayer, D. L. Grieco, R. Barelli, S. L. Cutuli, A. Ionescu Maddalena, E. Picconi, C. Sonnino, C. Sandroni, M. Antonelli, B. Gucyetmez, H. K. Atalan, F. Tuzuner, N. Cakar, M. Jacob, S Sahu, Y. P. Singh, Y. Mehta, K. Y. Yang, S. Kuo, V. Rai, T. Cheng, C. Ertmer, P Czempik, S. Hutchings, S. Watts, C. Wilson, C. Burton, E. Kirkman, D. Drennan, A. O’Prey, A. MacKay, R. Forrest, A. Oglinda, G. Ciobanu, M. Casian, C. Oglinda, C. T. Lun, H. J. Yuen, G. Ng, A. Leung, S. O. So, H. S. Chan, K. Y. Lai, P. Sanguanwit, W. Charoensuk, B. Phakdeekitcharoen, G. Batres-Baires, I. Kammerzell, T. Lahmer, U. Mayr, R. Schmid, W. Huber, E. Spanuth, H. Bomberg, M. Klingele, R. Thomae, H. Groesdonk, S. Bernas, M. Piechota, K. Mirkiewicz, A. González Pérez, J. Silva, A. Ramos, F. Acharta, M. Perezlindo, L. Lovesio, P. Gauna Antonelli, A. Dogliotti, C. Lovesio, J. Baron, J. Schiefer, D. M. Baron, P. Faybik, H. P. Shum, W. W. Yan, T. M. Chan, D. Marouli, A. Chatzimichali, S. Kolyvaki, A. Panteli, E. Diamantaki, E. Pediaditis, P. Sirogianni, P Ginos, E. Kondili, D. Georgopoulos, H. Askitopoulou, V. Vicka, D. Gineityte, D. Ringaitiene, J. Sipylaite, J. Pekarskiene, D. M. Beurskens, T. C. Van Smaalen, P. Hoogland, B. Winkens, M. H. Christiaans, C. P. Reutelingsperger, E. Van Heurn, G. A. Nicolaes, F. S. Schmitt, E. S. Salgado, J. F. Friebe, T. F. Fleming, J. Z. Zemva, T. S. Schmoch, F. U. Uhle, L. K. Kihm, C. M. Morath, C. N. Nusshag, M. Z. Zeier, T. B. Bruckner, A. M. Mehrabi, P. N. Nawroth, M. W. Weigand, S. H. Hofer, T. B. Brenner, G. Fotopoulou, I. Poularas, S. Kokkoris, E. Brountzos, S. Zakynthinos, C. Routsi, M. Saleh, M. Elghonemi, K. F. Nilsson, J. Sandin, L. Gustafsson, R. Frithiof, I. Skorniakov, A. Varaksin, D. Vikulova, O. Shaikh, C. Whiteley, M. Ostermann, G. Di Lascio, L. Anicetti, M. Bonizzoli, G. Fulceri, M. L. Migliaccio, P. Sentina, M. Cozzolino, A. Peris, D. Khadzhynov, F. Halleck, O. Staeck, L. Lehner, K. Budde, T. Slowinski, T. Slowinski, D. Kindgen-Milles, D. Khadzhynov, N. Huysmans, M. Vander Laenen, A. Helmschrodt, W. Boer, A. Debain, J. Jonckheer, W. Moeyersons, K. Van zwam, L. Puis, K. Staessens, P. M. Honoré, H. D. Spapen, E. De Waele, A. Perez Ruiz de Garibay, B. Ende-Schneider, C. Schreiber, B. Kreymann, A. Bini, E. Votino, G. Giuliano, I. Steinberg, L. Vetrugno, D. Trunfio, A. Sidoti, E. Brogi, F. Forfori, M. Conroy, B. Marsh, J O’Flynn

**Affiliations:** 1 Uppsala University, Uppsala, Sweden; 2 National University Hospital, Singapore, Singapore; 3 Yong Loo Lin School of Medicine, National University Singapore, Singapore, Singapore; 4 University College London, London, UK; 5 Uppsala University, Uppsala, Sweden; 6 Department of Medical Sciences, Uppsala University, Uppsala, Sweden; 7 University Hospital, Leuven, Belgium; 8 McMaster University, Hamilton, Canada; 9 Montreal Clinical Research Institute, Montreal, Canada; 10 Uppsala University, Uppsala, Sweden; 11 Uppsala Universitet, Uppsala, Sweden; 12 Hospital Israelita Albert Einstein, São Paulo, Brazil; 13 Inselspital, Bern University Hospital, University of Bern, Bern, Switzerland; 14 Department of Medical Sciences, Uppsala, Sweden; 15 Department of Molecular Biosciences, Stockholm, Sweden; 16 Department of Surgical Sciences, Uppsala, Sweden; 17 Southeast University, Nanjing, China; 18 V.A. Negovsky Research Institute of General Reanimatology, Moscow, Russia; 19 Aichi Medical University, Aichi, Japan; 20 Imperial College, London, UK; 21 King’s College London, London, UK; 22 Bloomsbury Institute of Intensive Care Medicine, London, UK; 23 Center for Heart Lung Innovation - University of British Columbia (UBC), Vancouver, Canada; 24 Lund University, Lund, Sweden; 25 University, Ulm, Germany; 26 University Hospital Medical School, Ulm, Germany; 27 Beth Israel Deaconess Medical Center, Boston, MA USA; 28 Whitehead Institute, Cambridge, MA USA; 29 UZ Leuven, Leuven, Belgium; 30 KU Leuven, Leuven, Belgium; 31 Kantonsspital Aarau, Aarau, Switzerland; 32 University Hospital Basel, Basel, Switzerland; 33 Kantonsspital Luzern, Lucerne, Switzerland; 34 Kantonsspital Münsterlingen, Münsterlingen, Switzerland; 35 Bürgerspital Solothurn, Solothurn, Switzerland; 36 Loyola University, Chicago, IL USA; 37 University of Utah School of Medicine, Eccles Institute of Human Genetics, Salt Lake City, UT USA; 38 Asahi Kasei Pharma America Corporation, Waltham, UK; 39 Mogilev Regional Hospital, Mogilev, Belarus; 40 Hachiouji medical center, Tokyo medical university, Tokyo, Japan; 41 Università Politecnica delle Marche, Ancona, Italy; 42 Beth Israel Deaconess Medical Center, Boston, MA USA; 43 Odense University Hospital, Odense, Denmark; 44 Hadassah University Hospital, Jerusalem, Israel; 45 Marienhaus-Kliniken, Saarlouis, Germany; 46 IMBM, Bene Ataroth, Israel; 47 Medical School of Athens University, Evangelismos, Athens, Greece; 48 Medical School of Athens University, “Attikon” Hospital, Athens, Greece; 49 Kantonsspital Aarau, Aarau, Switzerland; 50 Morton Plant Hospital, Clearwater, FL USA; 51 Pitié-Salpêtrière, Paris, France; 52 University Department of Medicine, Kantonsspital Aarau, Aarau, Switzerland; 53 Morton Plant Hospital, Clearwater, FL USA; 54 Groupe Hospitalier Pitié-Salpêtrière, Paris, France; 55 University Hospital, Verona, Italy; 56 Kantonsspital Aarau, Aarau, Switzerland; 57 Morton Plant Hospital, Clearwater, FL USA; 58 Bakirkoy Dr.Sadi Konuk Training and Research Hospital, Istanbul, Turkey; 59 Charles Nicolle Hospital, Tunis, Tunisia; 60 Negovsky Research Institute of General Reanimatology, Moscow, Russia; 61 Pirogov National Medical and Surgical Center, Moscow, Russia; 62 University of Szeged, Szeged, Hungary; 63 University of Pécs, Pécs, Hungary; 64 DIAneering GmbH, Heidelberg, Germany; 65 Department of Medicine III, University Clinics Halle (Saale), Martin-Luther-University, Halle-Wittemberg, Germany; 66 Mitsubishi Chemical Europe, Düsseldorf, Germany; 67 Cairo University, Cairo, Egypt; 68 El-Sahel Teaching Hospital, Cairo, Egypt; 69 Cairo University, Cairo, Egypt; 70 Electricity Hospital, Cairo, Egypt; 71 Department of Anaesthesiology, University Medical Center Eppendorf, Hamburg, Germany; 72 Department of Intensive Care Medicine, University Medical Center Eppendorf, Hamburg, Germany; 73 Institute of Clinical Pharmacology and Toxicology, University Medical Center Hamburg-Eppendorf, Hamburg, Germany; 74 Department of Vascular Medicine, University Medical Center Hamburg-Eppendorf, Hamburg, Germany; 75 Department of Anaesthesiology, University Medical Center Eppendorf, Hamburg, Germany; 76 Department of Cardiovascular Surgery, University Heart Center Hamburg-Eppendorf, Hamburg, Germany; 77 Department of Clinical Pharmacology and Toxicology, University Medical Center Hamburg-Eppendorf, Hamburg, Germany; 78 Department of Intensive Care Medicine, University Medical Center Hamburg-Eppendorf, Hamburg, Germany; 79 Department of Laboratory Medicine, University Medical Center Hamburg-Eppendorf, Hamburg, Germany; 80 Department of Vascular Medicine, University Heart Center Hamburg-Eppendorf, Hamburg, Germany; 81 University Clinical Centre Ljubljana, Ljubljana, Slovenia; 82 University of Ljubljana, Faculty of Medicine, Ljubljana, Slovenia; 83 Beth Israel Deaconess Medical Center, Boston, MA USA; 84 Health Sciences University, Kocaeli Derince Traning Hospital, Kocaeli, Turkey; 85 Marmara university hospital, Istanbul, Turkey; 86 Royal Sussex County Hospital, Brighton, UK; 87 University Hospital Lewisham, London, UK; 88 AC Camargo Cancer Center, Sao Paulo, Brazil; 89 The First Affiliated Hospital of Suzhou University, Suzhou, China; 90 Tokyo Women’s Medical University Hospital, Tokyo, Japan; 91 New Cross Hospital, Wolverhampton, UK; 92 Washington University School of Medicine, St Louis, Missouri USA; 93 Alamanos KAT hospital, Kifisia, Greece; 94 Centro Hospitalar Universitario de Coimbra, Coimbra, Portugal; 95 Royal Brompton Hospital, London, UK; 96 Royal Brompton and Harefield NHS Trust, London, UK; 97 Hospital of Lithuanian University of Health Sciences, Kaunas, Lithuania; 98 Lithuanian University of Health Sciences, Kaunas, Lithuania; 99 Innsbruck Medical University Hospital, Innsbruck, Austria; 100 Intensive Care Unit. St. Boniface Hospital, Verona, Italy; 101 Uppsala University, Uppsala, Sweden; 102 Department of Surgical Sciences, Uppsala University, Uppsala, Sweden; 103 Department of ICU, PYNEH, Hong Kong, China; 104 Department of Anaesthesia and Intensive Care, Tuen Mun Hospital, Hong Kong, China; 105 Hong Kong East Cluster Department of Clinical Pathology, Hong Kong, China; 106 Department of Microbiology, The University of Hong Kong, Hong Kong, China; 107 Faculty of Pharmacy, Mahidol University, Bangkok, Thailand; 108 Faculty of Medicine, Ramathibodi Hospital, Mahidol University, Bangkok, Thailand; 109 Apollo hospitals, Bhubaneswar, India; 110 Hospital Kaunas Clinics of Lithuanian University of Health Sciences, Kaunas, Lithuania; 111 Hospital Kaunas Clinics of Lithuanian University of Health Sciences, Kaunas, Lithuania; 112 Universidad Dr. Jose Matias Delgado, Santa tecla, El Salvador; 113 King Faisal specialist hospital and research centre, Riyadh, Saudi Arabia; 114 Hospital Clinic, Barcelona, Spain; 115 Università degli Studi di Milano, IRCCS Fondazione Ca Granda Ospedale Maggiore Policlinico, Milan, Italy; 116 Hospital of Lithuanian University of Health Sciences, Kaunas, Lithuania; 117 Hospital of Lithuanian University of Health Sciences, Kaunas, Lithuania; 118 Lithuanian University of Health Sciences, Kaunas, Lithuania; 119 AOUP, Pisa, Italy; 120 Fondazione Toscana Gabriele Monasterio, Pisa, Italy; 121 First Division of Infectious Diseases, Cotugno Hospital, Napoli, Italy; 122 Jena University Hopital, Jena, Germany; 123 University of British Columbia, Vancouver, Canada; 124 University of Queensland, Brisbane, Australia; 125 British Columbia Childrens Hospital, Vancouver, Canada; 126 University of Kocaeli, Kocaeli, Turkey; 127 Uludag University, Bursa, Turkey; 128 Marmara University, Istanbul, Turkey; 129 Turkish Society of Intensive Care Medicine, Istanbul, Turkey; 130 Istitute for Pulmonary Diseases of Vojvodina, Sremska Kamenica, Serbia; 131 CEDIMAT, Santo Domingo, Dominican Republic; 132 Mayo Clinic, Rochester, New York USA; 133 Intensive Care National Audit & Research Centre, London, UK; 134 Guy’s and St Thomas’ NHS Foundation Trust, London, UK; 135 DOr Institute for Research and Education - IDOR, Rio De Janeiro, Brazil; 136 University Medical Centre Maribor, Maribor, Slovenia; 137 University of Szeged, Szeged, Hungary; 138 University of Pécs, Pécs, Hungary; 139 Loyola University, Chicago, Illinois USA; 140 Asahi Kasei Pharma America Corporation, Waltham, Massachusetts USA; 141 Weill Cornell Medicine, New York, New York USA; 142 Asahi Kasei Pharma America, Waltham, Massachusetts USA; 143 ASST di Pavia, Vigevano, Italy; 144 Scuola di Specialità Anestesia e Rianimazione, Università Degli Studi, Pavia, Italy; 145 National and Kapodistrian University of Athens, Medical School, Athens, Greece; 146 National and Kapodistrian University of Athens, Medical School, Athens, Greece; 147 University of Thrace, Alexandroupolis, Greece; 148 Aghios Dimitrios General Hospital, Thessaloniki, Greece; 149 G.Gennimatas General Hospital, Thessaloniki, Greece; 150 Korgialeneion Benakeion Hospital, Athens, Greece; 151 Rockingham General Hospital, Cooloongup, Australia; 152 University of Notre Dame, Fremantle, Australia; 153 Mackay Hospital, Mackay, Australia; 154 Queen Alexandra Hospital, Portsmouth, UK; 155 Royal Cornwall Hospital, Truro, UK; 156 Kyung Hee University Hospital at Gangdong, Kyung Hee University School of Medicine, Seoul, South Korea; 157 UMC Nijmegen, Nijmegen, Netherlands; 158 Maastricht University Medical Centre, Maastricht, Netherlands; 159 Medical University of Vienna, Vienna, Austria; 160 Shaare Zedek Medical Center, Jerusalem, Israel; 161 Hadassah Hospital, Jerusalem, Israel; 162 Queens Hospital, Essex, UK; 163 King George Hospital, Ilford, UK; 164 Russian Cancer Research Center, Moscow, Russia; 165 SP Botkin Hospital, Moscow, Russia; 166 Sant’Anna Hospital, Ferrara, Italy; 167 University of Trieste, Trieste, Italy; 168 Hospital Clínico Universidad de Chile, Santiago, Chile; 169 MSD, Midrand, South Africa; 170 BRI, Dallas, Texas USA; 171 Surgical Sciences, Uppsala, Sweden; 172 Medical Sciences, Uppsala, Sweden; 173 Lewisham and Greenwich NHS Trust, London, UK; 174 Hospital Universitario Alava - Santiago, Vitoria, Spain; 175 University of the Basque Country UPV/EHU, Vitoria, Spain; 176 Hospital Universitario Doce de Octubre, Madrid, Spain; 177 Charite Berlin Campus Virchow, Berlin, Germany; 178 Pharmacy, Clinic of Heidenheim, Heidenheim, Germany; 179 Department of Pharmaceutical Biosciences, Uppsala University, Uppsala, Sweden; 180 JMI Laboratories, North Liberty, Iowa, USA; 181 Hennepin County Medical Center, Minneapolis, Minnesota USA; 182 Osaka General Medical Center, Osaka, Japan; 183 Sakai City Medical Center, Osaka, Japan; 184 National and Kapodestrian University of Athens, Athens, Greece; 185 Hospital Clinic, Barcelona, Spain; 186 Evangelismos Hospital Medical School of Athens, Athens, Greece; 187 Korgialenio-Benakio E.E.S, Athens, Greece; 188 University General Hospital “ATTIKON”, Athens, Greece; 189 Hospital Universitario Politècnic la Fe, Valencia, Spain; 190 Hospital Universitario Virgen de la Victoria, Malaga, Spain; 191 Hospital Universitario de Tarragona Joan XXIII, Tarragona, Spain; 192 Polyphor Ltd, Allschwil, Switzerland; 193 Medical-Surgical ICU, Inserm CIC-1435, CHU Dupuytren, 87042 Limoges, France; 194 Medical-Surgical ICU, District Hospital Center, La Roche-sur-Yon, France; 195 Medical-Surgical ICU, University Hospital, Tours, France; 196 ICU, Cliniques universitaires Saint-Luc, Université catholique de Louvain (UCL), Brussels, Belgium; 197 Combioxin SA, Geneva, Switzerland; 198 Medical University of Innsbruck, Innsbruck, Austria; 199 Medical University of Graz, Graz, Austria; 200 King’s College Hospital, London, UK; 201 Ramón y Cajal University Hospital, Madrid, Spain; 202 University Hospital, Leuven, Belgium; 203 Panum Institute, Copenhagen, Denmark; 204 Beth Israel Deaconess Medical Center, Boston, Massachusetts USA; 205 Istanbul University Cerrahpasa Medical School, Istanbul, Turkey; 206 Hospital Clínico Universidad de Chile, Santiago, Chile; 207 Universidad de Chile, Santiago, Chile; 208 Università Politecnica delle Marche, Ancona, Italy; 209 Papworth Hospital, Cambridge, UK; 210 Liverpool Heart and Chest Hospital, Liverpool, UK; 211 Hamad medical corporation, Doha, Qatar; 212 KU Leuven, Leuven, Belgium; 213 Erasmus Medical Centre, Rotterdam, Netherlands; 214 University of Alberta, Edmonton, Canada; 215 University of California, San Francisco, USA; 216 Barts Health NHS Trust, London, UK; 217 Queen Mary University, London, UK; 218 Kyung Hee University Hospital at Gangdong, Kyung Hee University School of Medicine, Seoul, South Korea; 219 MCL, Leeuwarden, Netherlands; 220 MCL, Leeuwarden, Netherlands; 221 Sheikh Khalifa Medical City, Abu Dhabi, United Arab Emirates; 222 Ibn sina university hospital, Rabat, Morocco; 223 Chulalongkorn University, Bangkok, Thailand; 224 Tabriz University of Medical Sciences, Tabriz, Iran; 225 Rabin Medical Center, Petah Tikvah, Israel; 226 Hadassah University Hospital, Jerusalem, Israel; 227 Hospital for Sick Kids, Toronto, Canada; 228 Lunguard Pty Ltd, Yavne, Israel; 229 Royal Liverpool University Hospital, Liverpool, UK; 230 MoreData GmbH, Giessen, Germany; 231 New Marton Farm, Oswestry, UK; 232 Università Politecnica delle Marche, Ancona, Italy; 233 Beth Israel Deaconess Medical Center, Boston, Massachusetts USA; 234 CHU Saint Pierre, Brussels, Belgium; 235 Centre Hospitalier Universitaire de Sherbrooke, Sherbrooke, Canada; 236 Hospital Posadas, Buenos Aires, Argentina; 237 Queen’s University, Kingston, Canada; 238 Hospital de Clinicas, Montevideo, Uruguay; 239 University Hospital, Montevideo, Uruguay; 240 Hospital Posadas, Buenos Aires, Argentina; 241 Université de Sherbrooke, Sherbrooke, Canada; 242 Queen’s University, Kingston, Canada; 243 Beth Israel Deaconess Medical Center, Boston, USA; 244 Acibadem Fulya Hospital, Istanbul, Turkey; 245 Acibadem University School of Medicine, Istanbul, Turkey; 246 Atasehir Memorial Hospital, Istanbul, Turkey; 247 Dr Wł. Biegański Regional Specialist Hospita, Department of Anaesthesiology and Intensive Therapy – Centre for Artificial Extracorporeal Kidney and Liver Support, Łódź, Poland; 248 Department of Insurance, University of Łódź, Łódź, Poland; 249 University of Łódź, Chair of Statistical Methods, Łódź, Poland; 250 Kasr Alainy, Cairo University, Cairo, Egypt; 251 Ankara Baskent Hospital, Ankara, Turkey; 252 Carol Davila University of Medicine and Pharmacy, Bucharest, Romania; 253 Maastricht University Medical Centre, Maastricht, Netherlands; 254 Maastricht University, Maastricht, Netherlands; 255 Keio University School of Medicine, Tokyo, Japan; 256 National Cancer Center, Tokyo, Japan; 257 Jichi Medical University Saitama Medical Center, Saitama, Japan; 258 Osaka Saiseikai Senri Hospital, Osaka, Japan; 259 Hiroshima City Hiroshima Citizens Hospital, Hiroshima, Japan; 260 Kansai Medical University, Osaka, Japan; 261 The University of Tokyo, Tokyo, Japan; 262 Iizuka Hospital, Fukuoka, Japan; 263 School of Medicine University of Occupational and Environmental Health, Fukuoka, Japan; 264 Oslo University Hospital, Oslo, Norway; 265 Norfolk & Norwich University Hospital, Norwich, UK; 266 Royal United Hospital NHS Foundation Trust, Bath, UK; 267 Hennepin County Medical Center, Minneapolis, Minnesota USA; 268 Queen Elizabeth Hospital, Kings Lynn, UK; 269 East Surrey Hospital, Redhill, UK; 270 University Hospital, Heraklion, Greece; 271 HCor-Hospital of the Heart, São Paulo, Brazil; 272 Universidade de Fortaleza - UNIFOR, Fortaleza, Brazil; 273 Hospital das Clínicas - FMUSP, São Paulo, Brazil; 274 Catholic University of the Sacred Heart, A. Gemelli University Hospital, Rome, Italy; 275 Acibadem University School of Medicine, Istanbul, Turkey; 276 Acibadem Fulya Hospital, Istanbul, Turkey; 277 Arnavutkoy Hospital, Istanbul, Turkey; 278 Klinikum St. Elisabeth Straubing GmbH, Straubing, Germany; 279 Krishna Institue of Medical Science, Secunderabad, Andhra Pradesh India; 280 Max Hospital, Patparganj, New Delhi India; 281 Medanta-The Medicity, Haryana, India; 282 Taipei Veterans General Hospital, School of Medicine, National Yang-Ming University, Taipei, Taiwan; 283 University of Malaya, Kuala Lumpur, Malaysia; 284 Sultanah Aminah Hospital, Bangunan Induk, Jalan Persiaran Abu Bakar Sultan, Johor, Malaysia; 285 University Hospital Muenster, Muenster, Germany; 286 Medical University of Silesia, Katowice, Poland; 287 Kings College Hospital, London, UK; 288 Defence Science & Technology Laboratory, Salisbury, UK; 289 Queen Elizabeth University Hospital (QEUH), NHS Greater Glasgow and Clyde, Glasgow, UK; 290 Institute of Mother and Child, Chisinau mun., Moldova; 291 State University of Medicine and Pharmacy, Chisinau, Moldova; 292 Alice Ho Miu Ling Nethersole Hospital, Hong Kong, Hong Kong; 293 Queen Elizabeth Hospital, Hong Kong, Hong Kong; 294 Faculty of medicine Ramathibodi, Bangkok, Thailand; 295 Srisungwan Hospital, Mae Hong Son, Thailand; 296 Faculty of Medicine, Ramathibodi Hospital, Mahidol University, Bangkok, Thailand; 297 Klinikum rechts der Isar, Technical University of Munich, Munich, Germany; 298 DIAneering GmbH, Heidelberg, Germany; 299 Department of Anaesthesiology, Intensive Care Medicine and Pain Medicine, Saarland University, University Medical Centre, Homburg/Saar, Germany; 300 Department of Medicine, Division of Nephrology and Hypertension, Saarland University, University Medical Centre, Homburg/Saar, Germany; 301 Mitsubishi Chemical Europe, Düsseldorf, Germany; 302 Dr Wł. Biegański Regional Specialist Hospital, Department of Anaesthesiology and Intensive Therapy – Centre for Artificial Extracorporeal Kidney and Liver Support, Łódź, Poland; 303 Hospital Universitario Central de Asturias, Oviedo, Spain; 304 Sanatorio Parque, Rosario, Argentina; 305 Grupo Oroño, Rosario, Argentina; 306 Medical University Vienna, Vienna, Austria; 307 Pamela Youde Nethersole Eastern Hospital, Hong Kong, Hong Kong; 308 The University of Hong Kong, Hong Kong, Hong Kong; 309 University Hospital, Heraklion, Greece; 310 Vilnius University, Vilnius, Lithuania; 311 Cardiovascular Research Institute Maastricht (CARIM), Maastricht, Netherlands; 312 Maastricht University Medical Center (MUMC), Maastricht, Netherlands; 313 School for Public Health and Primary Care (CAPHRI), Maastricht, Netherlands; 314 Academic Medical Center Amsterdam, Amsterdam, Netherlands; 315 University Hospital Heidelberg, Heidelberg, Germany; 316 National and Kapodistrian University of Athens, Athens, Greece; 317 General Hospital of Athens, Athens, Greece; 318 Kasr Alainy, Cairo University, Cairo, Egypt; 319 Örebro University Hospital, Örebro, Sweden; 320 Karolinska Institutet, Stockholm, Sweden; 321 Uppsala University, Uppsala, Sweden; 322 Guy’s & St Thomas’ NHS Foundation Trust, London, UK; 323 Institute of Industrial Ecology, Russian Academy of Sciences, Ekaterinburg, Russia; 324 Sverdlovsk Regional Clinical Hospital 1, Ekaterinburg, Russia; 325 Careggi Teaching Hospital, Florence, Italy; 326 Dmytro Khadzhynov, Berlin, Germany; 327 Charité Campus Mitte, Berlin, Germany; 328 University Hospital Duesseldorf, Duesseldorf, Germany; 329 Ziekenhuis Oost Limburg, Genk, Belgium; 330 Immundiagnostiek AG, Bensheim, Germany; 331 UZ Brussel, Jette, Belgium; 332 Hepa Wash GmbH, Munich, Germany; 333 University of Pisa, Pisa, Italy; 334 University of Udine, Udine, Italy; 335 Mater Misericordiae University Hospital, Dublin, Ireland

## P349 Muscle mitochondrial function and N+/K+ -ATPase activity are unaffected by sepsis in pigs

### M Von Seth, L Hillered, A Otterbeck, K Hanslin, A Larsson, J Sjölin, M Lipcsey

#### Uppsala University, Uppsala, Sweden

**Introduction**

Imbalance in cellular energetics has been suggested to be an important mechanism for organ failure in sepsis and septic shock. We hypothesized that such energy imbalance would either be caused by metabolic changes leading to decreased energy production or by increased energy consumption. Thus, we set out to investigate if mitochondrial dysfunction or decreased energy consumption alters cellular metabolism in muscle tissue in experimental sepsis.

**Methods**

We submitted anesthetized piglets to sepsis (n = 12) or placebo (n = 4) and monitored them for 3 hours. Plasma lactate and markers of organ failure were measured hourly, as was muscle metabolism by microdialysis. Energy consumption was intervened locally by infusing ouabain through one microdialysis catheter to block major energy expenditure of the cells, by inhibiting the major energy consuming enzyme, N+/K + -ATPase. Similarly, energy production was blocked infusing sodium cyanide (NaCN), in a different region, to block the cytochrome oxidase in muscle tissue mitochondria.

**Results**

All animals submitted to sepsis fulfilled sepsis criteria as defined in Sepsis-3, whereas no animals in the placebo group did. Muscle glucose decreased during sepsis independently of N+/K + -ATPase or cytochrome oxidase blockade. Muscle lactate did not increase during sepsis in naïve metabolism. However, during cytochrome oxidase blockade, there was an increase in muscle lactate that was further accentuated during sepsis. Muscle pyruvate did not decrease during sepsis in naïve metabolism. During cytochrome oxidase blockade, there was a decrease in muscle pyruvate, independently of sepsis. Lactate to pyruvate ratio increased during sepsis and was further accentuated during cytochrome oxidase blockade. Muscle glycerol increased during sepsis and decreased slightly without sepsis regardless of N+/K + -ATPase or cytochrome oxidase blocking. There were no significant changes in muscle glutamate or urea during sepsis in absence/presence of N+/K + -ATPase or cytochrome oxidase blockade.

**Conclusions**

These results indicate increased metabolism of energy substrates in muscle tissue in experimental sepsis. Our results do not indicate presence of energy depletion or mitochondrial dysfunction in muscle and should similar physiologic situation be present in other tissues, other mechanisms of organ failure must be considered.

## P350 Pilot study showing reduced bone strength at 96 hours in rodent sepsis

### ME Cove^1^, NS Chew^2^, LH Vu^2^, RZ Lim^2^, Z Puthucheary^3^

#### ^1^National University Hospital, Singapore, Singapore; ^2^Yong Loo Lin School of Medicine, National University Singapore, Singapore, Singapore; ^3^University College London, London, United Kingdom

**Introduction**

Bone mineral density (BMD) is reduced in critical care survivors [1], and long-term follow up has shown increased fracture risk [2]. It is unclear if these changes are a consequence of acute critical illness, or reduced activity afterwards. Bone health assessment during critical illness is challenging, and direct bone strength measurement is not possible. We used a rodent sepsis model to test the hypothesis that critical illness causes early reduction in bone strength and changes in bone architecture.

**Methods**

20 Sprague-Dawley rats (350 ± 15.8g) were anesthetised and randomised to receive cecal ligation and puncture (CLP) (50% cecum length, 18G needle single pass through anterior and posterior walls) or sham surgery (cecum mobilised, no CLP), and then returned to their cages. 10 rodents (5 CLP, 5 sham) were sacrificed at 24 hours, and the remaining 10 at 96 hours. Femur bones were harvested and bone strength testing was conducted using the Instron 5543 (Instron Corp, USA). Trabecular bone strength was measured using a femoral neck break and cortical bone strength tested using a femoral shaft 3-point bending test. Bone architecture was assessed using micro-computerised tomography (microCT) imaging (PerkinElmer, USA), and images analysed with BoneJ [3].

**Results**

All 20 rats survived to the end of the protocol. The load required to fracture the femoral neck and shaft was not significantly different for CLP and sham groups at 24 hours (97 ± 19N vs 81 ± 10N p = 0.12 and 127 ± 8N vs 119 ± 18N p = 0.35, respectively). However, at 96 hours there was a significant reduction in the fracture force at both the femoral neck and shaft in the CLP group, compared to sham (75 ± 11N vs 97 ± 13N p = 0.02 and 102 ± 20N vs 139.9 ± 28N p = 0.04). In contrast, there were no bone architecture differences, as measured by bone volume/total volume, trabecular thickness/separation, connectivity density, anisotropy and BMD (all p > 0.20) using microCT at 24 or 96 hours.

**Conclusions**

In this rodent model of sepsis, there is a significant reduction in trabecular and cortical bone strength at 96 hours. In the absence of changes in bone architecture, these findings suggest sepsis may induce early biochemical changes affecting bone strength. We plan further rodent experiments to confirm these results, increase our power, assess nano-mechanics and complete a histological analysis.

**References**

1 Orford NR et al: Am J Resp Crit Care 2016,193:736–744

2 Rawal J et al: Crit Care 2015,19:165

3 Doube M et al: Bone 2010, 47:1076–1079

## P351 Endotoxin clearance by the spleen is unaffected by pre-existing systemic inflammation in porcine septic shock

### K Hanslin^1^, F Wilske^2^, P Skorup^2^, E Tano^2^, J Sjölin^2^, M Lipcsey^1^

#### ^1^Uppsala University, Uppsala, Sweden; ^2^Uppsala University, Department of Medical Sciences, Uppsala, Sweden

**Introduction**

As part of the mononuclear phagocytic system, the spleen participates in bacterial and endotoxin clearance. In our previous study we saw decreased endotoxin clearance by the liver in pigs with pre-existing systemic inflammatory response (SIR). We therefore hypothesized that immunosuppression induced by SIR may also lead to decreased trans-splenic endotoxin clearance, and set out to investigate this in a porcine model of sepsis.

**Methods**

15 anesthetized pigs received an [i]E. coli[/i] infusion intravenously (i.v.) for 3 hours (h). In group Pre-existing SIR (n = 6), SIR was induced by 24 h of i.v. endotoxin infusion prior to the [i]E. coli[/i] infusion. Group Non-Pre-existing SIR (n = 6) received the bacterial infusion without prior endotoxin exposure. To study the effects of 24 h of anesthesia alone, “Controls” (n = 3) received saline instead of endotoxin for 24 h prior to the bacterial infusion (not included in the primary analysis). The kinetic chromogenic LAL-test was used to analyze endotoxin in arterial and splenic venous blood samples.

**Results**

**Fig. 1 (abstract P351).**
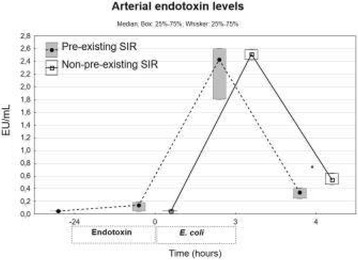
See text for description

All animals receiving endotoxin developed SIR prior to the [i]E. coli[/i] infusion. The amounts of [i]E. coli[/i] given to the groups were comparable. Endotoxin levels were similar at 3 h, just before the end of the [i]E. coli[/i] infusion, in the Pre-existing SIR and Non-Pre-existing SIR groups in arterial (Fig. 1) and splenic venous blood (2.40 (1.94-2.60) vs. 2.81 EU/mL (2.73-2.91) median (IQR)). Furthermore, endotoxin levels at 4 h, one hour after completed [i]E. coli[/i] infusion, were lower in Pre-existing SIR vs. Non-Pre-existing SIR group both in arterial (Fig. 1) and splenic venous blood (0.38 (0.31-0.42) vs. 0.51 EU/mL (0.47-0.71); p < 0.05). There was no difference in the ratio of splenic venous to arterial endotoxin levels between Pre-existing SIR and Non-Pre-existing SIR groups.

**Conclusions**

In our model, the endotoxin clearance by the spleen is not affected by pre-existing inflammatory response in porcine [i]E. coli[/i] septic shock.

## P352 The role of autophagy in critical illness-induced organ failure

### I Derese, S Thiessen, S Derde, T Dufour, L Pauwels, Y Bekhuis, G Van den Berghe, I Vanhorebeek

#### University Hospital, Leuven, Belgium

**Introduction**

Increasing evidence implicates mitochondrial dysfunction and endoplasmic reticulum (ER) stress, which activates the unfolded protein response (UPR), as contributors to critical illness-induced organ failure. Both can be alleviated by autophagy, a cellular defense mechanism. However, a phenotype of insufficiently activated autophagy has been observed during critical illness. We hypothesized that insufficient hepatic autophagy during critical illness aggravates liver damage/failure, hallmarked by mitochondrial dysfunction and ER stress.

**Methods**

In a centrally catheterized mouse model of critical illness, induced by cecal ligation and puncture, the effect of genetic inactivation of hepatic autophagy (via inducible deletion of autophagy gene 7 in liver) on survival, markers of organ damage, apoptosis, UPR and mitochondrial content and function was evaluated in the acute (30 hrs) and prolonged (3 days) phase. For each time point, 2 groups of critically ill mice and 2 groups of healthy pair-fed mice were included (at least 10 surviving mice per group), where each time autophagy was inactivated in one group but not in the other.

**Results**

Hepatic autophagy deficiency during critical illness did not affect survival, but increased hepatic damage/dysfunction. In the acute phase, this was illustrated by higher plasma ALT (P = 0.0001), and by elevated markers of apoptosis (P = 0.001) and more mitochondrial dysfunction (Complex V activity, P = 0.02) in liver. In the prolonged phase, hepatic autophagy inactivation increased apoptosis (P = 0.01) and aggravated mitochondrial dysfunction (Complex V activity, P = 0.005) in liver. Autophagy deficiency did not affect mitochondrial DNA content (day 1 P = 0.98, day 3 P = 0.57). Autophagy deficiency time-dependently modulated several branches of the UPR in liver. On day 1, it decreased activation of the IRE1alpha-XBP1s (P = 0.003) and ATF6-CREB3L3 pathway (P = 0.003), coinciding with a diminished inflammatory response as shown by lower C-reactive protein gene expression (P = 0.006), but did not affect the p-eIF2alpha pathway (P = 0.26). At day 3, autophagy deficiency increased the activation of the p-eIF2alpha pathway (P = 0.03), but not the IRE1alpha-XBP1s (P = 0.37) or ATF6-CREB3L3 (P = 0.14) pathway.

**Conclusions**

Insufficient hepatic autophagy during critical illness aggravates liver damage, coinciding with more mitochondrial dysfunction and a time-dependent modulation of the UPR, hereby likely aggravating liver failure.

## P353 Role of leptin and proprotein convertase subtilisin/kexin type 9 in modulating pulmonary inflammation in a murine model of early sepsis

### M Khan^1^, D Dwivedi^1^, J Zhou^1^, A Prat^2^, NG Seidah^2^, PC Liaw^1^, AE Fox-Robichaud^1^

#### ^1^McMaster University, Hamilton, Canada; ^2^Montreal Clinical Research Institute, Montreal, Canada

**Introduction**

Obesity increases the risk of sepsis but how obesity shapes the immune responses to infection is unknown. Similar to patients, we previously demonstrated that Western diet fed obese mice have reduced lung inflammation during early sepsis. In this study we explore the potential mechanisms to explain this finding. Proprotein Convertase Subtilisin/Kexin Type 9 (PCSK9) is a protein involved in cholesterol homeostasis that is implicated in sepsis survival. Leptin is a hormone produced by adipocytes that regulates energy homeostasis and is increased in obesity and sepsis. We hypothesized that either PCSK9 and/or leptin contributes to the obesity-associated lung protection in sepsis.

**Methods**

PCSK9 deficient, PCSK9 overexpressing and wild type mice on a C57/Bl6 background were fed either a high fat Western diet (WD) or a normal chow diet (NCD) for 15 weeks (n = 5/group). Sepsis was induced by cecal ligation and puncture (CLP). Tissues were harvested six hours post surgery. For the leptin studies mice were housed in static cages for 10-12 weeks. Mice were injected with recombinant leptin protein (1mg/kg)) one hour prior to CLP, then re-anesthetized and tissues collected at 6 hours. All mice were resuscitated with 2ml of lactated Ringers SQ pre surgery, and 1ml IV post surgery. Lung injury was assessed by myeloperoxidase (MPO in U/mg of tissue) assay of lung tissues and histopathology scores. Data are expressed as mean ± SEM and analyzed using ANOVA or t-test.

**Results**

Septic PCSK9 over expressing mice fed NCD had greater lung MPO levels (46.5 ± 4.5) compared to PCSK9 deficient mice (31.1 ± 1.7) on NCD (p < 0.01). In mice fed the WD for 15 wks the protection from the loss of PCSK9 was no longer present, however the injury was reduced. Septic PCSK9 deficient (14.4 ± 1.4), wildtype (17.6 ± 0.9) and overexpressing (17.9 ± 1.0) mice on WD had no significant differences in lung MPO levels. This correlated with histopathology scores for PCSK9 deficient (0.7 ± 0.2), wildtype (1.1 ± 0.2) and PCSK9 overexpressing (1.3 ± 0.7) septic mice. We found that leptin treated septic mice had lower lung MPO (32.6 ± 1.6) levels compared to saline treated septic mice (46.6 ± 3.5) (p < 0.001). Sham operated mice had significantly lower MPO levels (12.7 ± 1.7 for leptin and 11.8 ± 1.2 for saline) compared to septic counterparts.

**Conclusions**

Our data suggests that both lack of PCSK9 and increases in leptin contribute to the lung protection in early sepsis. However, when exposed to a WD the potential benefits of PCSK9 deficiency to further reduce lung injury are no longer evident. These findings have implications for potential therapeutic strategies to reduce sepsis-induced lung injury.

## P354 The role of oxygen delivery on plasma lactate and organ failure in experimental septic shock

### M Von Seth, P Skorup, L Hillered, A Larsson, J Sjölin, M Lipcsey

#### Uppsala University, Uppsala, Sweden

**Introduction**

The concept resuscitation of patients with septic shock, aiming at normalization of oxygen delivery (DO2), to limit tissue dysoxia and organ failure has not been confirmed in recent trials. Elevated plasma lactate in septic shock is considered as a key marker of inadequate DO2. We hypothesized that, apart from severely decreased levels, DO2 is not associated to plasma lactate in a model of septic shock.

**Methods**

We investigated the effects of circulatory shock and inflammation on plasma lactate in a retrospective analysis of 105 anesthetized endotoxemic (N = 61) or bacteremic (N = 44) piglets in shock. Tumor Necrosis Factor alpha (TNF-α) and Interleukin-6 (IL-6) were measured hourly during 6 hours (h) of shock. Muscle metabolism was monitored by microdialysis. The animals were stratified per degree of shock by DO2. The primary analysis was the breakpoint of insufficient DO2 to yield an elevated plasma lactate. ANOVA and regression models were used.

**Results**

All animals developed macrocirculatory shock, elevated plasma and muscle lactate levels, elevated levels of cytokines in plasma, as well as renal and pulmonary failure. At 3 h, DO2 was 289 ± 68 mL x min-1 x m-2 (mean ± SD) and plasma lactate levels were 2.7 (2.0-3.6) mmol x L-1 (median(IQR)). Mixed venous saturation (SvO2) decreased and oxygen extraction increased linearly with DO2 (p > 0.001). Oxygen consumption (VO2) was not DO2 dependent.

Plasma lactate increased at DO2 < 250 mL x min-1 x m-2 (p < 0.001). Urinary output decreased at DO2 < 250 mL x min-1 x m-2 (p < 0.01), but static lung compliance was not DO2-dependent. Muscle glucose, lactate and pyruvate, urea and glutamate were not DO2-dependent. Muscle glycerol was DO2-dependent without breakpoint.

Plasma lactate correlated to Mean Arterial Blood Pressure (MAP), DO2 and peak IL-6, but not Systemic Vascular Resistance Index (SVRI) and peak TNF-α.Urinary output correlated to DO2 and MAP. Static lung compliance did not correlate to any parameters above.

Over time, muscle pyruvate increased and muscle glycerol and glucose decreased but no changes in muscle lactate and glutamate were seen. Muscle pyruvate correlated to MAP. Muscle glycerol correlated to MAP and to TNF-α.

**Conclusions**

In porcine experimental sepsis, elevated plasma lactate was only associated with very low DO2 while oxygen consumption was unaffected by low DO2 despite development of organ failure. Tissue metabolism was associated with both inflammatory and circulatory changes. Our findings suggest that the current concepts of resuscitation focusing on restoration of oxygen delivery must be combined with measures to limit the inflammatory response.

## P355 The lung is not a significant source of lactate in a pig model of sepsis

### A Otterbeck, K Hanslin, M Lipcsey, A Larsson, M Von Seth

#### Uppsala Universitet, Uppsala, Sweden

**Introduction**

We used a porcine sepsis model to investigate pulmonary hypoxia as an explanation for lactate elevation in sepsis. We measured the pulmonary lactate production and shunt fraction during bacteremia. Sepsis is a condition characterized by severe organ failure as a result of a dysregulated response to infection. Central in many pathophysiological theories is the decreased delivery or utilization of oxygen by tissues. Normal physiology dictates that hypoxia leads to lactate production, a prognostic marker in sepsis. Thus, hypoxia has been used as an explanation for both organ dysfunction and elevated plasma (p-)lactate in sepsis. However, new research has implied other mechanisms. Previous studies have reported increased pulmonary lactate production in sepsis [1], the lungs being a generally well-oxygenated organ. However, these studies have not measured pulmonary shunt fraction and hence cannot estimate if the entire lung is ventilated.

**Methods**

We used 13 anesthetized pigs where 9 were randomized to a sepsis group and 4 were randomized to a sham group. All pigs received a pulmonary artery catheter and an arterial line. Pigs in the sepsis group were infused with live Escherichia coli for 3 hours (h) and in the sham group with NaCl. Blood cultures were used to confirm bacteremia. Blood gases, blood tests and physiological parameters were collected hourly. Lactate production was calculated by the p-lactate gradient from pulmonary artery to systemic artery and cardiac index. Shunt fraction was estimated at 3 h after ventilation with 100% O2 for 5 minutes.

**Results**

**Fig. 2 (abstract P355).**
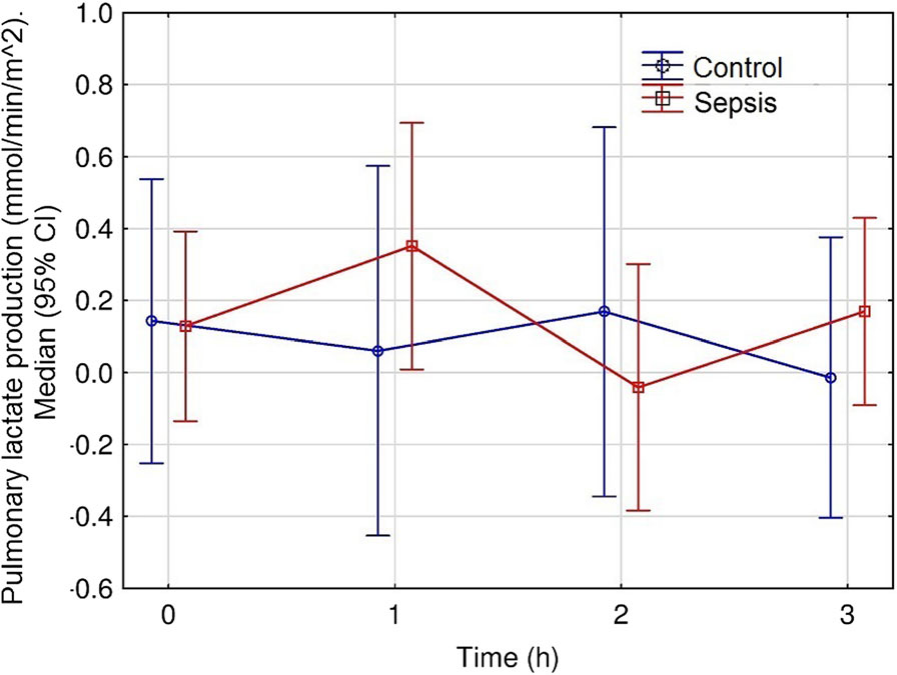
See text for description

Sepsis occurred in all pigs in the sepsis group, according to the criteria from Sepsis-3, and in no pigs in the sham group (p = 0.03). Global oxygen delivery (DO2) remained equal in both groups. Arterial lactate was higher (p = 0.003) in the sepsis group after 1 hour with a median value of 2.3 mmol/L vs. 0.95 mmol/L. Negative and positive lactate production occurred over the lungs in both groups (Fig. 2). There was no difference in pulmonary lactate production or pulmonary shunt fraction between groups. Neither group had significant shunt formation.

**Conclusions**

In this study we found a high p-lactate in septic pigs despite a high DO2. In absence of pulmonary shunts, the lung was not a major source, nor a major scavenger, of plasma lactate.

**Reference**

1. Garcia-Alvarez, M. et al. Sepsis-associated hyperlactatemia. Crit. Care Lond. Engl. 18, 503 (2014).

## P356 Association between inflammation, mitochondrial function and lactate clearance

### T Correa^1^, J Pereira^1^, J Takala^2^, S Jakob^2^

#### ^1^Hospital Israelita Albert Einstein, São Paulo, Brazil; ^2^Inselspital, Bern University Hospital, University of Bern, Bern, Switzerland

**Introduction**

Systemic lactate clearance (LaCl) higher than 10% during the first hours of sepsis resuscitation is associated with better outcomes, but the mechanisms are unclear. We aimed to investigate the relationship between lactate clearance, inflammatory response, and mitochondrial respiration.

**Methods**

Original data from two previously published studies were re-analyzed [1,2]. In cohort 1, pigs were randomized to be resuscitated for 48h starting 6, 12 and 24h, respectively, after fecal peritonitis induction (n = 8, each) [1]. Hemodynamics, inflammatory parameters, and mitochondrial function were analyzed. In cohort 2, 16 pigs with fecal peritonitis were immediately resuscitated for 24h [2]. Regional lactate exchange was measured. Animals of both groups were categorized according to LaCl > =10% or <10% during 6h of resuscitation.

**Results**

Overall mortality was 20% (4/20) for animals with LaCl > =10% and 60% (12/20) for animals with LaCl > =10% (p = 0.022). In cohort 1, systemic hemodynamics were similar in LaCl > =10% (n = 13) and LaCl < 10% (n = 11) groups. Plasma interleukin-6 levels were lower at study end in LaCl > =10% [45 (37-204) vs. 166 (128-310; median, IQR), p = 0.047]. Complex 1 state 3 [586 (386-688) vs. 353 (242-483), p = 0.026] and state 4 [157 (117-247) vs. 122 (89-151), p = 0.045], and Complex 2 state 3 [1156 (828-1401) vs. 761 (664-932), p = 0.041] and state 4 [376 (281-451) vs. 269 (225-326), p = 0.032] isolated brain but not hepatic, myocardial or skeletal muscle mitochondrial respiration were higher at study end in LaCl > =10% compared to LaCl < 10%. In cohort 2, mesenteric, total hepatic and renal blood flows were higher at study end in LaCl > =10% (n = 7) vs. LaCl < 10% group (n = 9), despite similar cardiac output. Hepatic lactate influx and uptake were approximately 1.5 and 3 times, respectively, higher in LaCl > =10% vs. LaCl < 10% (p = 0.066, both).

**Conclusions**

Systemic lactate clearance > =10% vs. <10% during early resuscitation after abdominal sepsis was associated with lower plasma interleukin-6, and higher brain mitochondrial respiration. Blood flow redistribution to abdominal organs in animals with high systemic lactate clearance increases the potential to deliver and extract lactate.

**References**

1. Correa TD et al. Effect of treatment delay on disease severity and need for resuscitation in porcine fecal peritonitis. Crit Care Med 40:2841–9, 2012.

2. Brandt S et al. Effect of fluid resuscitation on mortality and organ function in experimental sepsis models. Crit Care 13:R186, 2009.

## P357 Dynamics of endotoxin, interleukin-6 and organ dysfunction after treatment with antibiotics in an E.coli porcine intensive care sepsis model

### P Skorup^1^, L Maudsdotter^2^, E Tano^1^, M Lipcsey^3^, M Castegren^1^, A Larsson^1^, J Sjölin^1^

#### ^1^Dept. of Medical Sciences, Uppsala, Sweden; ^2^Dept. of Molecular Biosciences, Stockholm, Sweden; ^3^Dept. of Surgical Sciences, Uppsala, Sweden

**Introduction**

Endotoxin released during Gram-negative bacterial infections induces the production of pro-inflammatory cytokines and may accentuate the development of septic shock [1]. Gram-negative bacteria exposed to â-lactam antibiotics in vitro release endotoxin but in a minor extent when the â-lactam antibiotic is combined with an aminoglycoside [2]. The primary purpose of the study was to investigate the dynamics in endotoxin and interleukin-6 (IL-6) concentration as well as leukocyte activation and subsequent organ dysfunction in a large animal intensive care sepsis model in order to explore the relevance of antibiotic-induced endotoxin liberation and inflammatory response in vivo. Whether the addition of an aminoglycoside to a â-lactam antibiotic results in a reduced endotoxin release and systemic inflammation constituted a secondary aim.

**Methods**

A prospective placebo-controlled study was conducted on anesthetized pigs in an intensive care setting. All pigs were administered Escherichia coli as a 3h intravenous infusion. At 2h the animals were subjected to antibiotic treatment (n = 18) receiving either cefuroxime alone (n = 9) or the combination of cefuroxime and tobramycin (n = 9), whereas controls received saline (n = 18). During 4h after administration of antibiotics/saline, plasma endotoxin, IL-6, leukocytes and organ dysfunction variables were recorded hourly and differences to the values before treatment were calculated.

**Results**

All animals developed sepsis. Antibiotic-treated animals demonstrated a higher IL-6 response (p < 0.001), stronger leukocyte activation (p < 0.001) and more pronounced deterioration in pulmonary static compliance (p < 0.01) over time in comparison with controls. Animals treated with the combination demonstrated only a trend towards less inflammation in comparison with animals treated with cefuroxime alone. In plasma no differences in endotoxin concentration were observed between the groups.

**Conclusions**

Treatment with antibiotics elicits an inflammatory IL-6 response which is associated with leukocyte activation and pulmonary organ dysfunction, whereas no observable differences were seen in plasma endotoxin concentration. The reduction in cefuroxime-induced endotoxin release after the addition of an aminoglycoside in vitro could not be reproduced in vivo.

**References**

1. Bozza FA et al.: Crit Care 2007; 11: 1.

2. Goscinsky G et al.: Scand J Infect Dis 2003; 35: 40–6

## P358 Persisitent shift of th1 to th2 in one week after diagnosis of community-acquired severe sepsis predicts mortality

### M Xue, JY Xu, L Liu, YZ Huang, FM Guo, Y Yang, HB Qiu

#### Southeast University, Nanjing, China

**Introduction**

Recent studies have revealed that inflammation mediated by CD4+ T cells may contribute to the pathogenesis of sepsis. The role of the Th(T helper)1/Th2 balance in sepsis remains largely unknown. The aim of this study was to investigate the th2/th1 pattern and its impact on disease severity and outcomes in patients with new onset community-acquired severe sepsis.

**Methods**

This was a prospective observational study. Patients with community-acquired severe sepsis admitted to ICU within 24 hours were included. Blood sample was collected on day of admission(Day0, D0), 3rd Day (D3) and 7th Day (D7) after admission. Th2 and Th1 in lymphocyte were tested by flow cytometry. The increase of th2/th1 (>0.22) indicated immunosuppression. According to the change of th2/th1, patients were divided into 3 groups: immunosuppression recovered in early stage (th2/th1 began to decrease on D3, Group 1), immunosuppression recovered in late stage (th2/th1 began to decrease on D7, Group 2), immunosuppression worsened (th2/th1 kept increasing in a week, Group 3). The organ dysfunction, hospital-acquired infection(HAI) and 28-day prognosis was recorded. All patients or their legal representatives provided written informed consent. The study is registered with ClinicalTrials.gov, NCT 02883218.

**Results**

Seventy-four patients were eligible for study during Sept 18, 2014 to Sept 30, 2016. There were 34 cases in Group 1, 19 cases in Group 2, and 21 cases in Group 3. Baseline characteristics(age, sex, source of bacteremia, presence of comorbidities) of the population in different groups were similar.

**Fig. 3 (abstract P358).**
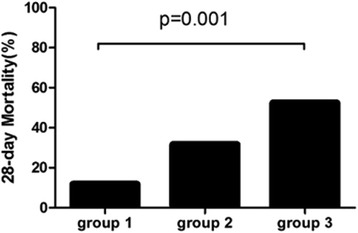
28-day mortality in different groups

**Fig. 4 (abstract P358).**
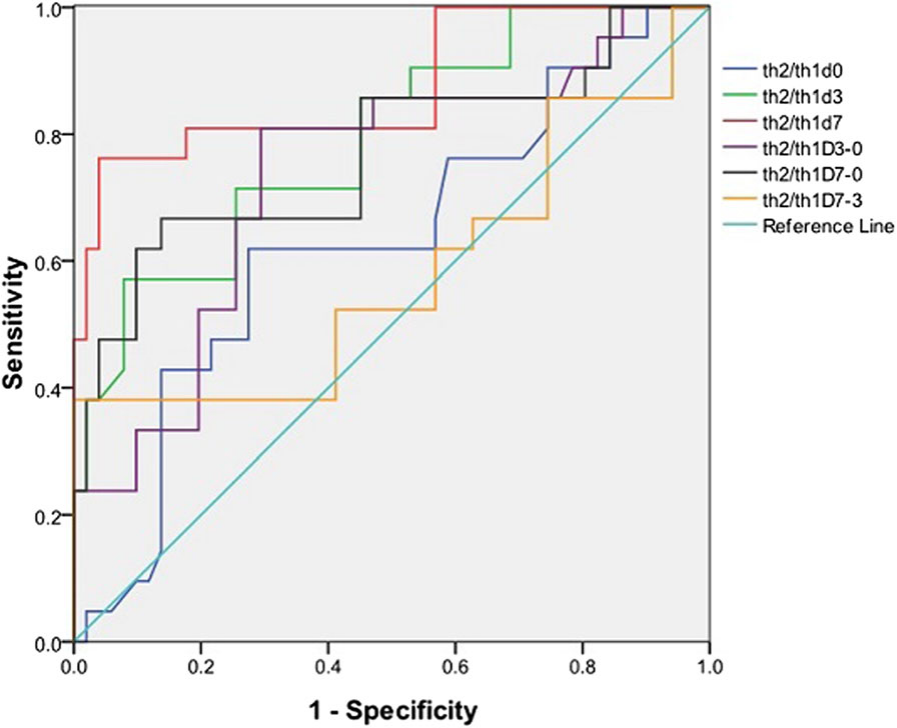
Receiver operating characteristics (ROC) curves comparing the ability of value of th2/th1 on Day0, Day3, Day7 as well as change of th2/th1 in one week. D3-0, change of th2/th1 between Day3 and Day0; D7-0, change of th2/th1 between Day7 and Day0; D7-3, change of th2/th1 between Day7 and Day3. Legend 2 : Figure 4. Receiver operating characteristic (ROC) curves of value of th2/th1

(1) Compared with Group1(11.8%), Group3(52.4%) showed a significantly higher 28-day mortality(P = 0.001), while group 2 showed a higher 28-day mortality of 31.6% with no significant difference (Fig. 3).

(2) There were no significant differences in terms of incidence of HAI and organ dysfunction among groups.

(3) The areas under the receiver operating characteristic (AUC) curves of value of th2/th1 on D7 was of great value(0.875)(Fig. 4). Using a th2/th1 on day7 cutoff value of >2.74 to determine 28-day mortality, the sensitivity was 76.2% with 96.1% specificity.

**Conclusions**

Persisitent shift of th1 to th2 in one week after diagnosis of community-acquired severe sepsis may be a predictor for immunosuppression. Patients with persistently increasing th2/th1 have poor outcome.

**References**

[1] Leentjens J, Kox M, Rebecca M, et al. Reversal of immunoparalysis in humans in vivo. Am J Respir Crit Care Med. 2012, 186: 838–845.

[2] Monneret G, Venet F, Pachot A et al. Monitoring immune dysfunctions in the septic patient: a new skin for the old ceremony. Mol Med. 2008, 14: 64–78.

[3] Inoue S, Suzuki-Utsunomiya K, Okada Y, et al. Reduction of immunocompetent T cells followed by prolonged lymphopenia in severe sepsis in the elderly. Crit Care Med. 2013, 41:810–819.

## P359 Genetic variants of intron region of aquaporin AQP5 gene and development of pulmonary edema in lung infection complicated by septic shock

### A Kuzovlev, V Moroz, A Goloubev, A Myazin, A Chumachenko, V Pisarev

#### V.A. Negovsky Research Institute of General Reanimatology, Moscow, Russia

**Introduction**

Product of AQP5 gene belongs to a family of aquaporins (AQPs), membrane proteins, responsible for the selective transmembrane transport of water. However, the value of polymorphic variants AQP5 in the development and progression of pul_monary edema in severe lung infection was studied so far. The aim of the investigation was to determine the value of genetic variants of a single nucleotide polymorphic site rs3736309 of intron 3 of aquaporin_5 (AQP5) gene in the course of critical illness in patients with documented pulmonary infection.

**Methods**

Patients with critical illness admitted to the intensive care units were examined during the course of treatment (n = 86, age 27 to 82 years, mean age 53.20 ± 14.34 years). Main diagnosis included malignancies (15%), peritonitis (16%) and necrotizing pancreatitis (37%). Patients developed nosocomi_al pneumonia (55%), acute respiratory distress syndrome (ARDS) (54%), septic shock (48%), ARDS combined with septic shock (33%). DNA genotyping was carried out using tetra_primer polymerase chain reaction (PCR). Statistical processing was performed using GraphPad InStat program (GraphPad, USA).

**Results**

The distribution of frequencies of genotypes AA, GA and GG (AQP5, rs3736309) in cohort of patients corresponded to Hardy_Weinberg equilibrium (P = 0.923) and was similar to frequencies of the alleles determined in healthy Caucasian individuals (literature data) (P > 0.05). In a subgroup of patients with septic shock and AQP5 AA (rs3736309) genotype the lower EVLWI values were found compared to patients with geno_types GG and GA with septic shock in spite of the same approach to treatment. Genetic variant AQP5 G+ (rs3736309) contributed to the development of pulmonary edema resistant to treatment (odds ratio, OR = 6,75; P = 0.032). Only the subgroup of patients with septic shock and geno_type G + (but not all patients or the subgroup of patients without septic shock of the same genotype) were char_acterized by significantly elevated levels of surfactant protein SP_D in plasma compared to patients of genotype AQP5 AA with septic shock (P < 0.05).

**Conclusions**

In septic shock, the presence of homozygous variant allele A (AA) of AQP5 rs3736309 is a favor_able factor for patients developing the pulmonary edema. The presence of allele AQP5 G (rs3736309) is a risk fac_tor for developing severe pulmonary edema and unfavorable prognosis in spite of treatment.

## P360 Withdrawn

## P361 Altered T cell repertoire diversity and increased PD-1 expression predict mortality in patients with septic shock

### N Takeyama, M Tsuda, H Kanou, R Aoki, Y Kajita, M Hashiba, T Terashima, A Tomino

#### Aichi Medical University, Aichi, Japan

**Introduction**

Sepsis causes impairment of innate and adaptive immunity by multiple mechanisms, including depletion of immune effector cells and T cell exhaustion. Although lymphocyte dysfunction is associated with increased mortality and potential reactivation of latent viral infection in patients with septic shock, the relation between viral reactivation and lymphocyte dysfunction is obscure. The objectives of this study were 1) to determine the relation of lymphocyte dysfunction to viral reactivation and mortality, and 2) to evaluate recovery of lymphocyte function during septic shock, including T cell receptor (TCR) diversity and the expression of programmed death 1 (PD-1).

**Methods**

In 18 patients with septic shock and latent cytomegalovirus infection, serial blood samples were obtained on days 1, 3, and 7 after the onset of shock, and immune cell subsets and receptor expression were characterized by flow cytometry. TCR diversity of peripheral blood mononuclear cells was analyzed by Multi-N-plex PCR, and cytomegalovirus DNA was quantified using a real-time PCR.

**Results**

Monocytes showed a decrease of TCR diversity and HLA-DR expression in the early stage of septic shock, while CD4+ T cells displayed an increase of PD-1 expression. Normalization of TCR diversity and PD-1 expression was observed by day 7, except in patients who died. cytomegalovirus reactivation was detected in 3 of the 18 patients during the first week of their ICU stay and all 3 patients died.

**Conclusions**

These changes are consistent with the early stage of immune cell exhaustion and indicate the importance of normal lymphocyte function for recovery from septic shock. Ongoing lymphocyte dysfunction is associated with cytomegalovirus reactivation and dissemination, as well as with unfavorable outcomes.

## P362 Vasopressin alone and with noradrenaline attenuates TNF-α production in an in-vitro model of monocyte priming and deactivation

### R Davies, KP O´Dea, S Soni, JK Ward, DJ O´Callaghan, M Takata, AC Gordon

#### Imperial College, London, United Kingdom

**Introduction**

Vasopressin is a safe and effective ‘catecholamine-sparing’ vasopressor in septic shock [1]. It also has anti-inflammatory properties, including inhibition of endotoxin-induced inflammatory cytokine release from macrophages in-vitro [2]. To further assess vasopressin’s anti-inflammatory effects in sepsis, we developed an in-vitro assay of monocyte priming and deactivation to model the pro- and anti-inflammatory responses, respectively.

**Methods**

Healthy volunteer CD14+ monocytes were cultured at 37°C for 20hrs with lipopolysaccharide (LPS, 100pg/ml) or IL-10 (10ng/ml), in combination with noradrenaline (5000pg/ml) and ‘high’ (300pmol/L) or ‘low’ (100pmol/L) dose vasopressin. Monocytes were analysed for HLA-DR and CD86 (T-cell co-stimulatory ligand) expression by flow cytometry, or stimulated with a ‘second-hit’ of LPS (10ng/ml) and TNF release measured by ELISA.

**Results**

**Fig. 5 (abstract P362).**
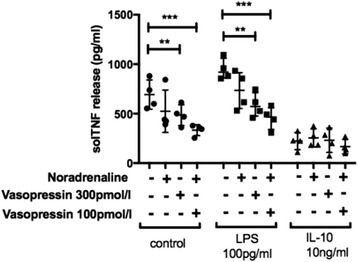
Effect of vasoprissin & noradrenaline on second-hit LPS induced monocyte TNF release. n = 4, **p < 0.01; ***p < 0.001

Pre-treatment of monocytes with LPS resulted in a primed phenotype of higher HLA-DR expression and TNF release on stimulation, whereas IL-10 reduced HLA-DR, CD86, and TNF release indicating a deactivated phenotype. Under normal and priming conditions, co-incubation with vasopressin alone or with noradrenaline significantly reduced TNF release (Fig. 5), but not HLA-DR/CD86 expression. In contrast, neither vasopressin nor noradrenaline affected the IL10-induced deactivated phenotype.

**Conclusions**

The vasopressin-mediated suppression of TNF release in normal or primed monocytes, but not in deactivated monocytes, suggests a selective immune-modulatory activity that may be beneficial in septic patients and warrants further investigation.

**References**

1. Gordon AC et al. JAMA.316:509–18,2016

2. Peng T-C et al. Tzu Chi Medical Journal.25:150–4,2013

Funding: Intensive Care Foundation

## P363 Relationship between lymphocyte subset expression and serum concentrations of pd-1/pd-l1 in sepsis – pilot study

### J Wilson^1^, Y Zhao^1^, M Singer^2^, J Spencer^1^, M Shankar-Hari^1^

#### ^1^King’s College London, London, United Kingdom; ^2^Bloomsbury Institute of Intensive Care Medicine, London, United Kingdom

**Introduction**

Programmed death antigen (PD-1) and ligand (PD-L1) are inducible negative regulators on leukocyte surface. The PD-1/PD-L1 pathway contributes to lymphocyte exhaustion and immunosuppression in sepsis [1]. A clinical trial is currently evaluating the safety of an anti-PD-L1 antibody in sepsis patients [2]. However, serum levels and differences in PD-1/PD-L1 expression by B and T cell subsets are unknown and are likely to influence the efficacy of this intervention. We tested the hypothesis that surface PD-1/PD-L1 expression will differ in B and T cell subsets, and that serum PD-1/PD-L1 levels will be high in sepsis.

**Methods**

A prospective observational cohort study in 22 critically ill adult sepsis patients, excluding those with immune deficiency states, was done with ethics approval and informed consent. Blood was taken on ICU admission day and PBMCs isolated. Patients (11 survivors & 11 non-survivors) were compared with contemporaneously collected and analysed samples from 11 healthy controls. Cell surface staining was performed with antibodies to CD3, CD19 [BD Biosciences], CD4, CD27, PD-1, PD-L1 and PD-L2 [Biolegend], and live dead stain Amcyan [Invitrogen]. FACS analyses were performed on a FACScalibur flow cytometer [BD Biosciences] and using Tree Star FlowJo software. Serum PD-1 and PD-L1 in the same cohort were measured by ELISA [Proteintech]. Data was analysed using PRISM.

**Results**

The percentages of B and CD4+ T cells expressing PD-1 and PD-L1 were significantly higher in survivors and non-survivors of sepsis compared to healthy controls. There were no significant differences between survivors and non-survivors in expression of PD-1 or PD-L1 by any lymphocyte subset. CD4 + CD27- memory T cells had significantly higher mean fluorescence intensity (MFI) and percentage positivity of PD-1 than CD4 + CD27+ T cells. The PD-1 MFI was significantly higher in CD19 + CD27+ memory B cells than CD19 + CD27- B cells. Serum PD-1 and PD-L1 concentrations were not different in sepsis patients compared to controls and values did not correlate with surface expression of PD-1 or PD-L1 by any lymphocyte subset in sepsis patients.

**Conclusions**

We show higher expression of PD-1 and PD-L1 by B cells and CD4+ T cells in sepsis compared to health, and higher expression of PD-1 by memory compared to naïve B cells and CD4+ T cells. Further research to identify patients likely to benefit from PD-1/PD-L1 blockade in sepsis is required.

**References**

1. Hotchkiss R et al. Nat Rev Imm; 13(12):862–74, 2013.

2. https://clinicaltrials.gov/ct2/show/NCT02576457

3. Singer M et al. JAMA; 315(8):801–10, 2016.

## P364 Pcsk9 loss-of-function genotype is associated with better short and long-term outcomes in sepsis

### K Roveran Genga^1^, C Lo^1^, M S. Cirstea^1^, K R. Walley^1^, J A. Russell^1^, A Linder^2^, J H. Boyd^1^

#### ^1^Center for Heart Lung Innovation - University of British Columbia (UBC), Vancouver, Canada; ^2^Lund University, Lund, Sweden

**Introduction**

Reduced activity of proprotein convertase subtilisin/kexin type 9 (PCSK9), by increasing the density of low density lipoprotein (LDL) receptors on hepatic cells, may decrease the systemic inflammatory response to sepsis due to increased clearance of pathogen lipids incorporated into LDL [1]. The purpose of this study was to determine the relationship between PCSK9 loss-of-function (LOF) variants and the risk of short and long-term death and/or hospital readmission(s) within a year following an episode of sepsis, in two distinct cohorts.

**Methods**

This was a retrospective observational study involving two cohorts from St. Paul’s Hospital in Vancouver, Canada: Cohort 1 was composed by 189 patients with septic shock admitted to intensive care unit between July 2000 and January 2004 and who survived at least 60 days post- hospitalization; Cohort 2 included 185 patients admitted to the Emergency Department from January 2011 to July 2013, with clinical diagnosis of sepsis. R46L, A53V, and I474V PCSK9 missense LOF SNPs were genotyped in all patients, who were classified in 3 groups: WT, 1 LOF, and 2 or more LOF according to the number of LOF alleles.

**Results**

Cohort 1: Time to event curves for 5-year mortality showed a trend to statistically lower probability of death within 5 years in patients with PCSK9 LOF (overall p = 0.062) and the presence of 1 PCSK9 LOF allele (Cox model) was independently associated with decreased hazard-ratio for 5-year mortality (0.578, 95% CI = 0.36-0.93, p = 0.024).

Cohort 2: Patients from the 2 or more LOF group had lower probability of death within 90 days in comparison to WT (p = 0.010) or 1 LOF patients (p = 0.028), and the presence of 1 PCSK9 LOF allele (Cox model) was independently associated with decreased HR for 90-day mortality (0.46, 95% CI 0.23-0.92, p = 0.030). Patients from the 2 or more PCSK9 LOF group also had lower probability of death or infection related readmissions when compared to WT (p = 0.015) or 1 LOF allele (p = 0.002), and the presence of 2 or more PCSK9 LOF alleles had the lowest adjusted HR for this outcome (HR = 0.32, 95% C.I. 0.11-0.92, p = 0.035).

**Conclusions**

PCSK9 LOF genotype is associated with decreased risk of 5-year and 90-day mortality, and all-cause 1-year death or readmissions due to infection after an episode of sepsis.

**Reference**

1. Walley KR et al.: Science translational medicine Sci Transl Med. 6(258):258ra143, 2014

## P365 Monocyte surface marker expression profile and cytokine secretion of critically ill patients with pseudomonas aeruginosa induced sepsis

### A Sedlag^1^, C Riedel^1^, M Georgieff^2^, E Barth^2^, H Bracht^2^, A Essig^2^, D Henne-Bruns^2^, F Gebhard^2^, K Orend^2^, M Halatsch^2^, M Weiss^2^

#### ^1^University, Ulm, Germany; ^2^University Hospital Medical School, Ulm, Germany

**Introduction**

Some critically ill patients are at high risk of severe sepsis due to infections with Pseudomonas aeruginosa (PSA) in the lung or abdomen, which is difficult to treat. The present study was performed to find out whether these critically ill patients on a surgical intensive care unit with sepsis due to PSA have a characteristic monocyte surface receptor expression and cytokine secretion patterns.

**Methods**

The surface markers CD163 (hemoglobin scavenger receptor; clearance of hemoglobin, adhesion to endothelial cells, tolerance induction, tissue regeneration; soluble form: antiinflammation), CD206 (mannose receptor for mannose on surface on microorganisms), intracellular levels of IFN-γ, CXCR1 (IL-8α chemokine receptor) and CXCR2 (IL-8β chemokine receptor) of monocytes of critically ill patients with PSA sepsis and of healthy controls were analyzed by flow cytometry. Furthermore, the IL-8 secretion levels in vivo and ex vivo after LPS stimulation were determined by ELISA.

**Results**

20 surgical patients with severe sepsis / septic shock with underlying PSA infections and of 22 healthy controls were monitored. The monocytes of the patients showed differences in the IL-8 secretion level. In line with high IL-8 or low IL-8 secretion in serum (965 ± 139 pg/ml vs. 232 ± 15 pg/ml) as well as in culture after LPS stimulation (2838 ± 259 pg/ml vs. 1097 ± 356 pg/ml), the expression of surface markers IFN-γ, CXCR1, CXCR2 and CD163 was high or low, respectively, however, always above those of the healthy control group (p < 0.05). CD206, only, showed the opposite behavior in that CD206 was highly expressed (3657 ± 279 MFI) on IL-8 low cells, whereas a low expression (17 ± 6 MFI) could be observed on IL-8 high cells (p < 0.001). The IL-8 low group had markedly higher severity of disease scores (SAPSII 34 ± 8) than the IL-8 high group (SAPSII 17 ± 6), and worse outcome (p < 0.001).

**Conclusions**

Different patterns of monocyte surface pattern expressions are associated with low or high IL-8 secretion of monocytes in patients with PSA sepsis. Low IL-8 expression and the respective surface pattern on monocytes may be associated with worse outcome.

## P366 Identification of a distinct metabolomic profile in acute influenza infection

### M Chase^1^, E Freinkman^2^, A Uber^1^, X Liu^1^, MN Cocchi^1^, MW Donnino^1^

#### ^1^Beth Israel Deaconess Medical Center, Boston, MA, United States; ^2^Whitehead Institute, Cambridge, MA, United States

**Introduction**

The goal of this investigation is to characterize the effect of acute influenza infection on the host metabolome. Metabolomics is an emerging field of research studying small molecules or metabolites providing a profile of the physiologic status of an organism at a point in time. The human metablomic response to acute influenza infection is not well-characterized. We hypothesized that acute influenza infection will induce a distinct metabolic response in the host which may enable the identification of host mediators in influenza pathogenesis.

**Methods**

We are conducting a randomized clinical trial administering atorvastatin or placebo to patients with acute influenza infection. As an exploratory aim, we assessed the metabolomic profile of enrolled patients at baseline, prior to study drug administration, compared to healthy controls. T-tests were used to compare 117 metabolites. Raw data were entered into MetaboAnalyst statistical software for analysis. We report findings based on Partial Least Squares-Discriminant Analysis (PLS-DA) which uses multivariate regression techniques to predict class membership based on original variables.

**Results**

**Fig. 6 (abstract P366).**
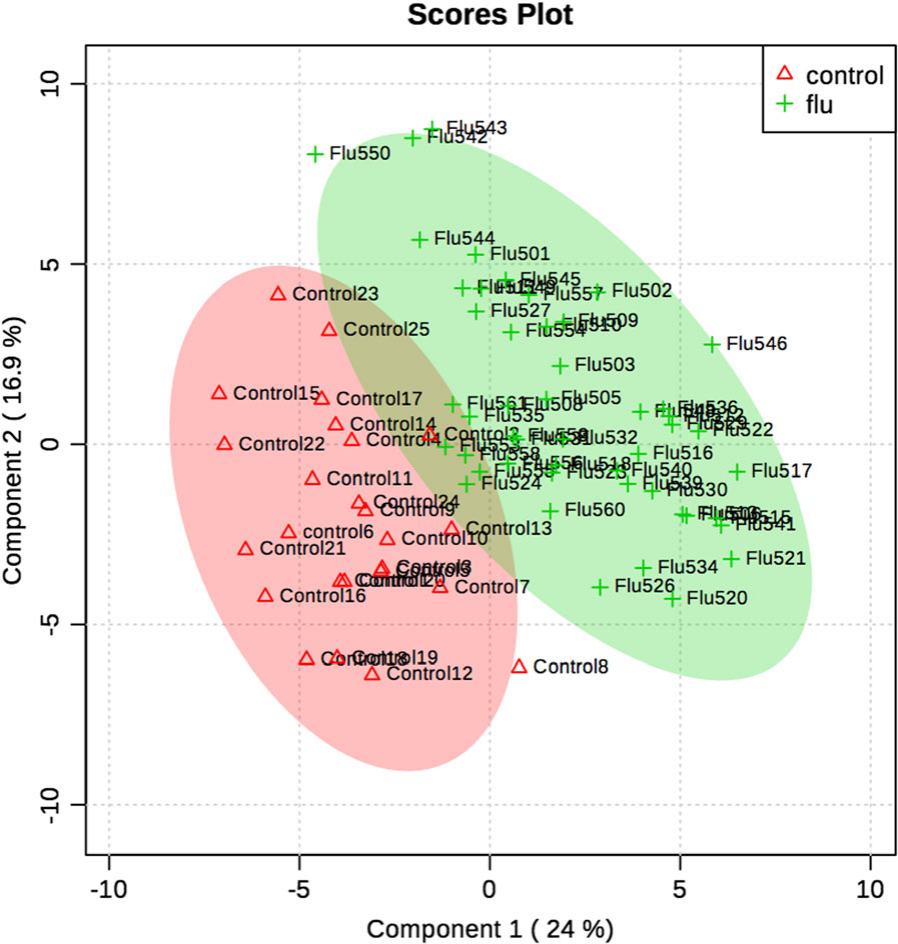
See text for description

We performed metabolomic analysis on serum samples of 49 statin-naïve patients with acute influenza and 25 healthy controls. We found 17 individual metabolites that were significantly different between groups at a threshold of p = 0.05. PLS-DA score plot demonstrated a distinct difference between influenza subjects and controls (Fig. 6) and PLS-DA model validation by permutation tests was highly significant (p < 5e-04). The most significant discriminating metabolites between influenza patients and controls were phosphocholine, phosphoethanolamine, nicotinamide, taurine, ADP, tryptophan, threonine, proline, citrulline (lower in flu samples) and kynurenine, acetoacetate, 3-hydroxybutyrate, hypoxanthine (higher in flu samples). Each of these metabolites had a VIP score of >1.4

**Conclusions**

In our metabolomics analysis of ED patients with acute influenza, we found a statistically significant difference in 17 metabolites as compared to healthy controls. We believe these data represent a potentially unique metabolic fingerprint in influenza infection. Further study is needed to elucidate potential metabolic pathways and host mediators that may contribute to influenza pathogenesis.

## P367 Von Willebrand factor and ADAMTS-13 influence the outcome of s. aureus bacteremia

### M Peetermans^1^, L Liesenborghs^2^, J Claes^2^, T Vanassche^2^, M Hoylaerts^2^, M Jacquemin^2^, K Vanhoorelbeke^2^, S De Meyer^2^, P Verhamme^1^

#### ^1^UZ Leuven, Leuven, Belgium; ^2^KU Leuven, Leuven, Belgium

**Introduction**

The size and functionality of the multimeric von Willebrand factor (VWF) molecule is regulated by its cleaving protease ADAMTS-13. While VWF and ADAMTS-13 levels correlate with disease course and outcome in the heterogenous population of septic patients, animal models have been inconclusive and mainly focused on gram-negative abdominal sepsis. It is unknown if modulation of the VWF/ADAMTS-13 balance can modulate the outcome of [i]S. aureus[/i] sepsis.

**Methods**

We aimed to study the role of VWF and ADAMTS-13 in [i]S. aureus[/i] sepsis, using patient data and an animal model.

**Results**

In patients with [i]S. aureus[/i] bloodstream infection, high VWF levels correlated with inflammatory parameters and inversely with kidney function. Low ADAMTS-13 levels were associated with disease severity and DIC parameters.

In a severe sepsis model, mice deficient in VWF showed improved survival, compared to wildtype mice. In contrast, [i]Adamts13-/-[/i] mice had increased mortality. Immediate clearance of bacterial load in blood was better in VWF deficient mice. The differences in mortality for the studied genotypes were associated with differential loads of organ microthrombi in liver and kidneys. Further experiments will study if administration of ADAMTS-13 can alleviate the course of [i]S. aureus[/i] sepsis.

**Conclusions**

In conclusion, this is the first study that consistently shows the relation of VWF, ADAMTS-13 and their ratio to disease severity in patients and mice with [i]S. aureus[/i] sepsis. Not only is the balance of VWF and its cleaving protease implicated in primary adhesion and bacterial retention, but VWF/ADAMTS-13 ratio also regulates the amount of organ microthrombi containing platelets, neutrophils and bacteria – and thus potentially end organ failure.

Targeting VWF multimers and/or the relative ADAMTS-13 deficiency that occurs in sepsis, should be explored as a potential new therapeutic target in [i]S. aureus[/i] endovascular infections.

## P368 Admission levels of asymmetric and symmetric dimethylarginine predict long-term outcome in patients with community-acquired pneumonia

### A Vögeli^1^, M Ottiger^1^, M Meier^1^, C Steuer^1^, L Bernasconi^1^, A Huber^1^, M Christ-Crain^2^, C Henzen^3^, C Hoess^4^, R Thomann^5^, W Zimmerli^3^, B Müller^1^, P Schütz^1^

#### ^1^Kantonsspital Aarau, Aarau, Switzerland; ^2^University Hospital Basel, Basel, Switzerland; ^3^Kantonsspital Luzern, Lucerne, Switzerland; ^4^Kantonsspital Münsterlingen, Münsterlingen, Switzerland; ^5^Bürgerspital Solothurn, Solothurn, Switzerland

**Introduction**

During infection, there is an activation of the L-arginine-nitric-oxide pathway, with a shift from nitric oxide synthesis to a degradation of L-arginine to its metabolites, asymmetric and symmetric dimethylarginine (ADMA and SDMA). We investigated the association of L-arginine, ADMA, and SDMA with adverse clinical outcomes in a well-defined cohort of patients with community-acquired pneumonia (CAP).

**Methods**

We measured L-arginine, ADMA, and SDMA in 268 CAP patients from a Swiss multicenter trial by mass spectrometry and used Cox regression models to investigate associations between blood marker levels and disease severity as well as mortality over a period of 6.1 years.

**Results**

Six-year mortality was 44.8%. Admission levels of ADMA and SDMA (μ mol/L) were correlated with CAP severity as assessed by the pneumonia severity index (r = 0.32, p < 0.001 and r = 0.56, p < 0.001 for ADMA and SDMA, respectively) and higher in 6-year non-survivors versus survivors (median 0.62 vs. 0.48; p < 0.001 and 1.01 vs. 0.85; p < 0.001 for ADMA and SDMA, respectively). Both ADMA and SDMA were significantly associated with long-term mortality (hazard ratios [HR] 4.44 [95% confidence intervals (CI) 1.84 to 10.74]) and 2.81 [95%CI 1.45 to 5.48], respectively). No association of L-arginine with severity and outcome was found.

**Conclusions**

Both ADMA and SDMA show a severity-dependent increase in patients with CAP and are strongly associated with mortality. This association is mainly explained by age and comorbidities.

**Fig. 7 (abstract P368).**
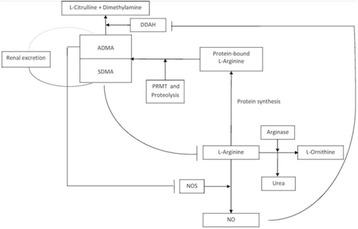
Metabolism of nitric oxide

**Fig. 8 (abstract P368).**
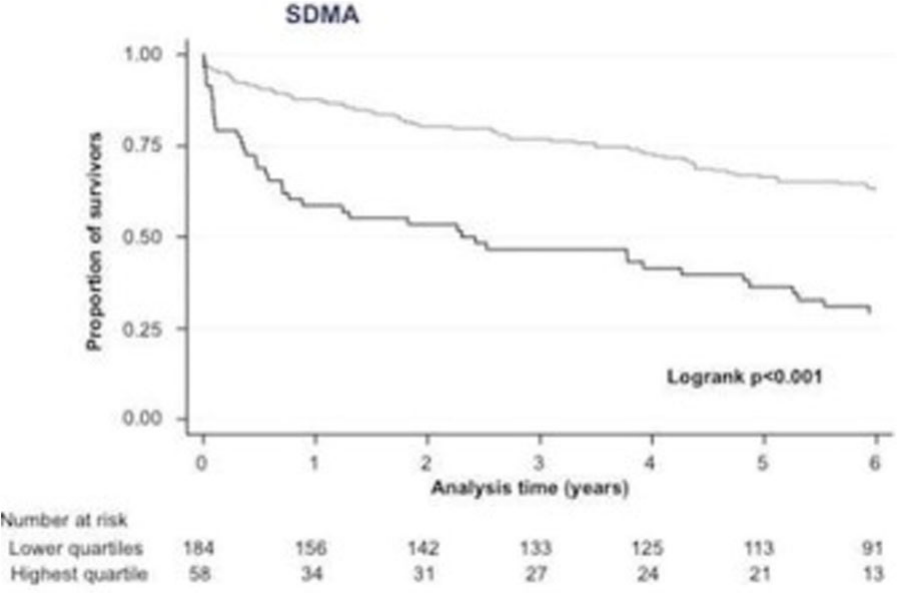
Kaplan-Meier survival estimates for SDMA

## P369 Decreased functional protein c levels may be predictive of the severity of sepsis associated coagulopathy and DIC

### D Hoppensteadt^1^, A Walborn^1^, M Rondina^2^, K Tsuruta^3^, J Fareed^1^

#### ^1^Loyola University, Chicago, IL, United States; ^2^University of Utah School of Medicine, Eccles Institute of Human Genetics, Salt Lake City, UT, United States; ^3^Asahi Kasei Pharma America Corporation, Waltham, United Kingdom

**Introduction**

Sepsis is a severe systemic inflammatory response to infection that manifests with widespread inflammation as well as endothelial and coagulation dysfunction that may lead to hypotension, organ failure, shock, and death. Disseminated intravascular coagulation (DIC) is a complication of sepsis involving systemic activation of the fibrinolytic and coagulation pathways that can lead to multi-organ dysfunction, thrombosis, and bleeding, with a two-fold increase in mortality. Several studies have reported that low levels of protein C predict outcome in patients with severe sepsis. Protein C helps to regulate coagulation by controlling the activation of factors Va and VIIIa. In addition, activated protein C has anti-inflammatory functions. The purpose of this study was to determine the functional protein C levels in this cohort of patients over the 8 day study period and to correlate protein C levels with survival.

**Methods**

De-identified serial plasma samples from patients diagnosed with sepsis-associated coagulopathy (n = 137) were obtained from the University of Utah under an IRB approved protocol. The citrated plasma samples were collected from adult patients in the ICU upon admission and ICU days 4 and 8. In addition, plasma samples from healthy volunteers (n = 50) were purchased from George King Biomedical (Overland, KS). P). Patients were assigned a DIC score based on the International Society of Thrombosis and Hemostasis (ISTH)criteria and categorized as having sepsis and no DIC, non-overt DIC and overt DIC. Plasma samples were analyzed for functional protein C levels using a clot-based method (Diagnostica Stago, Parsippany, NJ).

**Results**

The functional protein C levels on day 0 and day 4 were decreased in the septic patients compared to normal controls (p < 0.0010). In addition, the functional protein C levels decreased with an increase in severity of DIC. On day 8, there was a decrease in the functional protein C levels in both the non-overt and overt DIC groups compared to normal and patients with sepsis and no DIC. There also was a significant decrease in functional protein C levels in survivors compared to non-survivors (p < 0.010).

**Conclusions**

These results underscore the importance of functional protein C levels in the regulation of hemostasis. Furthermore, these studies demonstrate that decreased functional protein C contributes to the pathogenesis of sepsis associated coagulopathy as evident by the observed relationship with the severity of sepsis and decreased protein C levels. Thus functional protein C levels may be a useful prognostic marker to risk stratify patients with sepsis and DIC.

## P370 Cholesterol is a marker of multiple organ dysfunction syndromes after abdominal surgeries.

### S Tachyla

#### Mogilev Regional Hospital, Mogilev, Belarus

**Introduction**

According to the third international consensus sepsis is defined as life-threatening organ dysfunction caused by a dysregulated host response to infection. The aim of the study was to investigate the possibility of using additional laboratory marker cholesterol for early detection of multiple organ dysfunction syndromes (MODS) after abdominal surgeries.

**Methods**

After approval the ethics committee of the Mogilev Regional Hospital in a prospective observational study included 58 patients aged 18 to 85 years. All patients underwent laparotomy abdominal surgeries after which hospitalized in the intensive care unit. In R group (n = 30) of patients in the postoperative period was followed by the development of MODS, in the C group (n = 28) - patients without MODS. Determination of cholesterol produced daily by AU 680 biochemistry analyzer.

**Results**

Patients on the R group marked decrease in the level of cholesterol on the 4th day after surgery with 158.7 (96.8; 189.6) mg / dL to 127.7 (112.2; 174.1) mg / dL (Wilcoxon test, p = 0.0035). In group C was no significant differences in cholesterol levels. In R group value of cholesterol on 4th day was significantly higher in patients (n = 17) with recovery 147.1 (112.2; 170.3) mg / dL, than in patients (n = 13) with a fatal outcome 116.1 (100.6; 127.7) mg / dL (Mann-Whitney U test, p = 0.034). In the surviving patients reduced cholesterol level normalization was observed after 14 days after the operation.

We performed ROC-analysis to determine the diagnostic significance of cholesterol as a marker MODS. The area under the curve was 0.726 (p <0.001) 95% confidence interval from 0.684 to 0.769, sensitivity 73.6%, specificity 63.8%. The optimal threshold level of cholesterol as a predictor of MODS was determined of 130.0 mg / dL.

**Conclusions**

Determining the level of cholesterol, together with an assessment of clinical symptoms and blood formula, will provide early diagnosis of MODS after abdominal surgeries.

## P371 Relationship between white blood cell count and sepsis-related biomarkers in critically ill patients

### T Ikeda, S Ono, T Ueno, S Suda, T Nagura

#### Hachiouji medical center, Tokyo medical university, Tokyo, Japan

**Introduction**

Antibiotic therapy is a very important treatment for critically ill patients, but long-term administration can lead to antimicrobial resistance. Procalcitonin (PCT) has been used as a biomarker to monitor the effectiveness of antibiotic therapy with the aim of shortening the administration period. This study aimed to clarify the relationship of WBC counts to sepsis-related biomarkers (procalcitonin [PCT], endotoxin activity assay [EAA], interleukin-6 [IL-6], and presepsin) and 28-day mortality rate in critically ill patients.

**Methods**

We studied 422 patients (L-group with WBC counts <4000: 79 patients; H-group with WBC counts >12,000: 343 patients). Blood biochemistry and PCT, EAA, IL-6, and presepsin were measured immediately after ICU admission. Results were expressed as the mean ± SD (median). The Mann-Whitney U-test and chi-square test or Fisher’s exact test were used for statistical analysis. A p-value of <0.05 was considered to indicate a statistically significant difference.

**Results**

Regarding background factors, the APACHE2 scores in the L-group and H-group were 24.9 ± 9.2 (23.5) and 22.4 ± 9.1 (23.0), and the SOFA scores were 9.3 ± 3.9 (9) and 12.9 ± 3.4 (8), respectively. These values showed no significant differences between groups. PCT levels in the L-group and H-group were 65 ± 96 (25) and 20 ± 51 (3.2) respectively; EAA levels in the L-group and H-group were 0.59 ± 0.26 (0.62) and 0.36 ± 0.22 (0.32), respectively. IL-6 levels in the L-group and H-group were 61,728 ± 10,8417 (15,150) and 2440 ± 3422 (217), respectively. All of these values in the L-group tended to be higher than in the H-group. On the other hand, presepsin levels in the L-group and H-group were 1277 ± 943 (882) and 2440 ± 422 (1210), respectively. The 28-day mortality rate in the L-group was 34%, and 14.7% in the H-group. There was a significant difference between groups (p < 0.05).

**Conclusions**

In critically ill patients who entered the ICU, most sepsis-related biomarkers (PCT, EAA, IL-6) tended to be higher in the low leukocyte count (WBC < 4000) group, but only presepsin tended to be higher in the high WBC (>12,000) group. Further study to explain this difference is necessary.

## P372 Neutrophil-lymphocyte ratio and mortality during critical illness

### E Damiani, R Domizi, C Scorcella, S Tondi, S Pierantozzi, S Ciucani, N Mininno, E Adrario, P Pelaia, A Donati

#### Università Politecnica delle Marche, Ancona, Italy

**Introduction**

Immunologic alterations are common during critical illness and may determine outcome. We evaluated whether lymphopenia (lymphocyte count <1000*106/mmc) or a higher neutrophil-lymphocyte ratio (NLR, as a marker of inflammation) were associated with mortality in critically ill patients.

**Methods**

Prospective observational study on 221 consecutive adult patients admitted to our 14-bed Intensive Care Unit (ICU). Neutrophil and lymphocyte counts were recorded every day. Receiver operating characteristics (ROC) curves and binary logistic regression analysis were used to test the association between lymphopenia/NLR and ICU-mortality.

**Results**

**Fig. 9 (abstract P372).**
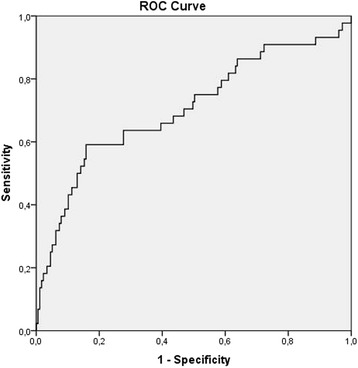
NLR and mortality

83% of patients showed lymphopenia at least once in the ICU stay. The mean lymphocyte count was a weak discriminant of ICU-mortality (area under the ROC curve: 0.63 [95% confidence interval 0.52-0.74]). A mean NLR [>=] 12 was able to discriminate Non-survivors with a sensitivity of 64% and specificity of 71% (ROC: 0.71 [0.61-0.80], Fig. 9). A mean NLR [>=] 12 was associated with higher mortality independently of age, presence of infection, days of mechanical ventilation and the Simplified Acute Physiology Score (adjusted odds ratio: 3.597 [1.760-7.354]).

**Conclusions**

A higher mean NLR was independently associated with mortality.

## P373 Leukocyte activity assessment using magnetic levitation in septic patients

### M Schou Andersen^1^, S Lu^1^, G Lopez^1^, AT Lassen^2^, I Ghiran^1^, NI Shapiro^1^

#### ^1^Beth Israel Deaconess Medical Center, Boston, MA, United States; ^2^Odense University Hospital, Odense, Denmark

**Introduction**

In sepsis, chemotactic factors induce swelling and activation of leukocytes. We have invented a novel portable bedside device based on the principles of magnetic levitation (two opposing magnets with a capillary tube in between that suspend cells) to image and quantify morphological properties of circulating leukocytes using whole blood. The device separates blood cells based on their mass and magnetic properties. Our aim was to determine whether magnetic levitation technique, by measuring leukocyte size and morphology parameters can accurately identify Emergency Department(ED) patients with sepsis.

**Methods**

Single-center, prospective, observational cohort study of a convenience sample of adult (>17y) patients from a 56.000 visit ED or affiliated out-patient lab between 3/2016-11/2016. Inclusion criteria: Sepsis: patients admitted to the hospital with suspected or confirmed infection. Non-infected Controls: ED or outpatient clinic patients without infection or acute illness. Procedures: Half a microliter of whole blood collected in EDTA tube, mixed with a paramagnetic gadolinium solution, transferred to a capillary tube, and placed between magnets for imaging and data analysis. Primary analysis: comparison of sepsis patients vs non-septic controls. Covariates of interest: leukocyte area, length, width, roundness, standard distribution(SD) of levitation height (measure of mass/charge dispersion). Means reported with t-test comparisons and calculation of area under the curve for assessment of diagnostic accuracy.

**Results**

We enrolled 41 non-infected controls and 21 sepsis patients: sepsis(6), severe sepsis(9) and septic shock(6). Our analyses identified a significant increase in the size of the circulating leukocytes in sepsis patients versus controls, as seen by the following morphology parameters: mean cell area, 570 pixels (SD) (±115) vs 411 (±46), p < 0.0001 with AUC = 0.89(0.80-0.99); cell length, 30 pixels (±2.5) vs 25 (±1.9), p < 0.0001, AUC = 0.89(0.81-0.98); and cell width, 27 (±2.4) vs 23 (±1.5), p < 0.0001, AUC = 0.93(0.86-1.00). Cell roundness was 2.2 (±1.1) vs 2.2 (±1.2), p = 0.9, AUC = 0.55(0.40-0.71). For mass:charge, levitation height SD was 71 (±26) vs 47 (±16), p = 0.0012, AUC = 0.80(0.67;0.93).

**Conclusions**

Septic patients had increased area, length, and width with strong diagnostic accuracy. Sepsis patients had leukocytes with a pattern of more variable mass and magnetic properties. Cell roundness was less promising. Trends towards these differences were associated with increased severity. This bedside technique shows promise as a novel diagnostic test for infection.

## P374 Intensive care patients undergoing tracheostomy have different patterns of cytokine and biomarker response

### U Trahtemberg^1^, S Sviri^1^, M Beil^2^, Z Agur^3^, P Van Heerden^1^

#### ^1^Hadassah University Hospital, Jerusalem, Israel; ^2^Marienhaus-Kliniken, Saarlouis, Germany; ^3^IMBM, Bene Ataroth, Israel

**Introduction**

Bedside tracheostomies are standardized, repeated procedures performed on relatively stable patients. The effects of tracheostomies at the biochemical level have not been studied before.

**Methods**

**Fig. 10 (abstract P374).**
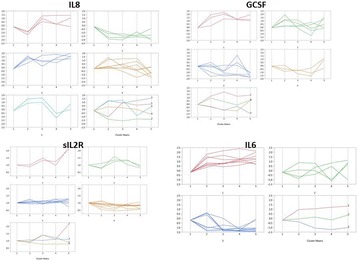
Clusters of cytokine patterns after tracheostomy.

5 blood samples were obtained from patients undergoing bedside tracheostomies at t = 0, 4, 8, 12 and 24 hrs. Vital signs and clinical lab measures were also recorded. The blood samples were assayed for analytes shown in Fig. 10.

**Results**

We report results for 23 patients from this ongoing study. Parametric and nonparametric tests showed few statistically significant changes between the time points. Clearly discernible patterns of response were observed, which were different between patients. Using a self-organizing map clustering algorithm, we were able to cluster the responses over time, for every analyte eg GCSF showed 3 possible patterns of response: peak at t = 2 and then a steady decrease (4 pts); peak at t = 2 and then a plateau starting at t = 3 (12 pts); and peak at t = 2 with an increase at t = 4 (5 pts; 2 pts were excluded by the algorithm). The other cytokines showed between 3 to 5 clusters of response patterns each.

**Conclusions**

Results were not amenable to parametric or non-parametric approaches. We could classify the patients according to response patterns. This highlights the need for the development of individual-level measures and the personalization of clinical care

## P375 The h3 haplotype of the EPCR gene determines high sEPCR levels in critically-ill patients

### E Jahaj^1^, A Vassiliou^1^, Z Mastora^1^, SE Orfanos^2^, A Kotanidou^1^

#### ^1^Medical School of Athens University, Evangelismos, Athens, Greece; ^2^Medical School of Athens University, “Attikon” Hospital, Athens, Greece

**Introduction**

The endothelial protein C receptor (EPCR) is a protein that regulates the protein C anticoagulant and anti-inflammatory pathways. A soluble form of EPCR (sEPCR) circulates in plasma and inhibits activated protein C (APC) activities. The clinical impact of sEPCR and its involvement in the septic process is under investigation. This study investigated possible association of EPCR haplotypes with sEPCR levels in critically-ill patients with suspected infection.

**Methods**

Two polymorphisms in the EPCR gene were previously genotyped in 239 Caucasian critically-ill patients, hospitalized in the intensive care unit (ICU) of “Evangelismos” Hospital, Athens, Greece. The old [1-2] and new [3] sepsis definitions were used to divide patients in groups. Patients were further divided according to their genotype. Plasma sEPCR levels were measured using a dedicated ELISA assay in all patients at the time of admission to the intensive care unit (ICU).

**Results**

Patients were categorized using both old and new sepsis definitions. With the old definitions patients were divided in two groups: severe sepsis/septic shock-positive (SS/SS + ve) and severe sepsis/septic shock-negative (SS/SS-ve). sEPCR levels were slightly higher in the SS/SS-ve group (p < 0.05). The patients were also divided according to their qSOFA score. In the three groups of patients, sEPCR levels were comparable (p > 0.05). However, when patients were divided according strictly to their genotype, plasma levels of sEPCR differed between genotypes (p < 0.0001) and between H3 and non-H3 carriers (p < 0.0001), with higher sEPCR levels in the H3 carriers.

**Conclusions**

Frequencies of SNPs determining EPCR haplotypes were in concordance with Caucasian frequencies. Critically-ill patients carrying at least one H3 allele had significantly higher levels of sEPCR than patients with no H3 alleles. Using both classification systems, sEPCR levels were not associated with sepsis severity.

**References**

1. ACCP/SCCM Consensus Conference: definitions for sepsis and organ failure and guidelines for the use of innovative therapies in sepsis. Critical care medicine 1992, 20(6):864–874.

2. Levy MM et al.: 2001 SCCM/ESICM/ACCP/ATS/SIS International Sepsis Definitions Conference. Intensive care medicine 2003, 29(4):530–538.

3. Singer M et al.: The Third International Consensus Definitions for Sepsis and Septic Shock (Sepsis-3). Jama 2016, 315(8):801–810.

## P376 The vasopressin surrogate marker copeptin reflects stress and predicts adverse clinical outcomes in unselected emergency patients: results of a multinational prospective cohort study

### Y Wirz^1^, R Sager^1^, D Amin^2^, A Amin^2^, S Haubitz^1^, P Hausfater^3^, A Huber^1^, A Kutz^1^, B Mueller^1^, P Schuetz^1^

#### ^1^Kantonsspital Aarau, Aarau, Switzerland; ^2^Morton Plant Hospital, Clearwater, FL, United States; ^3^Pitié-Salpêtrière, Paris, France

**Introduction**

Copeptin (pro-vasopressin) is a hormone derived from the same precursor protein as Vasopressin (AVP). Both hormones are secreted equimolar in response to physical stress and correlate with adverse clinical outcomes. In contrast to AVP, copeptin can be easily quantified by a sandwich immunoassay. Therefore, it serves as a surrogate marker for AVP and has emerged as a new prognostic marker in different settings including cardiovascular and infectious disease. The aim of this study was to evaluate the association between copeptin plasma concentrations and adverse outcomes in a large unselected study population seeking for emergency department (ED) care like under “real life” conditions.

**Methods**

This multicenter, observational cohort study consecutively included adult medical patients seeking for ED care in Switzerland, France and the USA during March 2013 to October 2014. We recorded initial clinical parameters and batch-measured the copeptin levels in a study population involving 7039 patients with available copeptin measures. We used logistic multivariate regression models (expressed in odds ratio, OR; 95% confidence interval, CI) and area under the receiver operating characteristic curve (AUC) to investigate the association of copeptin Plasma concentrations and different adverse outcomes (30-day all-cause mortality, intensive care unit [ICU] admission, initial treatment priority, main diagnoses and abnormal vital signs).

**Results**

During a 30-day follow-up 329 (4.7%) participants reached the primary endpoint of death. In our logistic regression models adjusted for age and gender copeptin levels (log transformed with a base of ten) significantly predicted the 30-day risk of death (OR 3.35, 95%CI 2.78-4.04, AUC 0.78), ICU admission (OR 2.89, 95%CI 2.48-3.36, AUC 0.69) and high initial treatment priority (OR = 2.23, 95%CI = 2.04-2.43, AUC = 0,66). Furthermore we found significant associations between copeptin levels and abnormal vital signs including low blood pressure (OR = 2.71, 95%CI = 2.35-3.11, AUC = 0.70). Stratifying by main admission diagnoses, subgroup analyses showed best performance of copeptin levels for 30-day mortality prediction in metabolic disorders (OR 11.00, 95%CI 2.46-49.20, AUC 0.87) and acute infections (OR 6.90, 95%CI 4.13-11.53, AUC 0.80).

**Conclusions**

Based on our findings copeptin serves as a strong prognostic marker on ED admission and might help to improve risk stratification in unselected medical ED patients.

## P377 The association of admission procalcitonin levels and adverse clinical outcome across different medical emergency patient populations: results from the multi-national, prospective, observational TRIAGE study

### RS Sager^1^, YW Wirz^1^, DA Amin^2^, AA Amin^2^, PH Hausfater^3^, AH Huber^1^, S Haubitz^1^, A Kutz^1^, B Mueller^1^, P Schuetz^1^

#### ^1^University Department of Medicine, Kantonsspital Aarau, Aarau, Switzerland; ^2^Morton Plant Hospital, Clearwater, FL, United States; ^3^Groupe Hospitalier Pitié-Salpêtrière, Paris, France

**Introduction**

Although Procalcitonin (PCT) has been extensively studied in infectious conditions, the clinical relevance of PCT in unselected emergency department (ED) patients remains incompletely understood. We investigated association of admission serum PCT levels and adverse clinical outcomes in a large ED cohort from a previous multicenter study.

**Methods**

We prospectively enrolled 7132 adult medical patients seeking ED care in three tertiary care hospitals in Switzerland, France and the United States. We used adjusted multivariable logistic regression models to examine association of admission PCT levels and 30-day mortality, stratified by principal medical diagnoses and concomitant comorbidities. We calculated regression models across different clinically-established PCT cut-offs (0.05, 0.1, 0.25 and 0.5ng/ml).

**Results**

During a 30-day follow-up 328 (4.9%) participants died. Mortality rate in the cut-off stratified groups were 1%, 3%, 7%, 14 and 17%, respectively. PCT had a high prognostic power for 30-day mortality with an area under the receiver operating characteristic curve of 0.74 (95%CI 0.72- 0.77; SE 0.0133). After adjustment for age, gender and main diagnoses, PCT cut-off levels were associated with a step-wise increase in risk of 30-day mortality with an adjusted odds ratio of 1.9, 4.5, 8.6 and 11.0 for pulmonal disease; 1.9, 4.7, 8.7 and 11.0 for cardiovascular disease and 1.9, 4.6, 8.8 and 11.4 for infectious disease, respectively. These associations were similar among different types of patients in regard to main diagnoses, comorbidities and age of patients.

**Conclusions**

In this large medical ED patient cohort, admission PCT was a strong and independent outcome predictor for 30-days mortality across different medical diagnoses. PCT may help to improve risk assessment in undifferentiated medical ED patients.

## P378 Procalcitonin level in klebsiella pneumoniae MDR infections in icu

### L Gottin, C Dell´amore, G Stringari, G Cogo, M Ceolagraziadei, M Sommavilla, F Soldani, E Polati

#### University Hospital, Verona, Italy

**Introduction**

Infections due to K.Pneumoniae carbapenemase (KPC) are increasing [1]. Trials analyzing the levels of Procalcitonin (PCT) in KPC infections are lacking. Aim of this study was to evaluate the levels of PCT in KPC infections compared with other Gram negative and verify if PCT levels correlate with sepsis severity.

**Methods**

From March 2012 to May 2013 133 adult patients with documented Gram negative bacteria infection, admitted to intensive care department were observed. In 53 patients KPC was isolated (KPC Group, KPCG), while in 80 patients other Gram negative bacteria caused the infection (Control Group, CG). All the patients were classified daily according to sepsis severity. Organ dysfunction was evaluated daily according to the SOFA score. PCT levels were detected daily by means of a quantitative method (Liaison). Microbiological coltures and imaging exams were performed according to clinicians decision. Statistical analysis was performed by means of the SPSS11.0 software.

**Results**

As regards demographic data significant differences were showed except ICU LOS that was longer in KPCG. The main site of isolation of KPCs was the respiratory tract. According to the different sepsis severity diagnosis we found the following PCT levels (ng/ml). Sepsis: KPCG 0.9 ± 1.9, CG 6.6 ± 11.1 (p < 0.01); Severe Sepsis: KPCG 3.4 ± 6, CG 11.6 ± 19.8 (p < 0.01); Septic Shock: KPCG 8.9 ± 15.9, CG 30.5 ± 35.7 (p < 0.01) In KPC Group the mean PCT level in non-survivors was 7ng/ml, while in survivors was 2.7 ng/ml (p < 0.01). PCT mean values increased according to SOFA score increases.

**Conclusions**

Our data demonstrated that in septic patients with KPC infection PCT levels were significantly lower that those in septic patients with infection due to other Gram negative species. As regards outcome PCT levels confirmed to be related to severity of the disease with a mean level significantly higher in non-survivor compared to survivor. Mortality rate was higher in KPC Group compared to Control Group, but the difference was not significant.

**References**

1. NadKami AS, et al. Am J Infect Control 2009; 30: 1180–5

**Table 1 (abstract P378).** See text for description

	KPCG (53pts)	CG (80pts)	p
AGE (years)	63.1 ± 18.3	65.3 ± 16.4	NS
Gender (M/F)	35/18	46/34	NS
ICU LOS (days)	30.9 ± 33.4	16 ± 15.7	<0.01
SOFA	6 ± 4.4	6.9 ± 4.6	NS
Mortality (%)	25 (47.2)	33 (41.2)	NS

Legend : demographic data

## P379 Prognostic value of procalcitonin or lactate clearance, or both, for risk assessment in sepsis patients

### M Meier^1^, T Baumgartner^1^, G Zurauskaité^1^, S Gupta^2^, B Mueller^1^, A Devendra^2^, P Schuetz^1^

#### ^1^Kantonsspital Aarau, Aarau, Switzerland; ^2^Morton Plant Hospital, Clearwater, FL, United States

**Introduction**

Several prognostic scores and biomarkers have been assessed for risk stratification in septic patients in intensive care units (ICUs). Serum lactate is a routinely used biomarker for the management of patients with sepsis and correlates with hypoperfusion and fluid resuscitation. Procalcitonin (PCT) is a bacterial infection marker and kinetics indicates the response to antimicrobial management. There is insufficient data comparing these two markers regarding outcome prediction. Herein, we compared the prognostic accuracy of lactate and PCT kinetics, and the combination of them, in a large, well defined US sepsis patient population.

**Methods**

This is an observational cohort study of adult patients with confirmed severe sepsis or septic shock included in a sepsis database from 14 different BayCare hospitals (Florida). All patients had PCT and lactate measurements on admission and during follow-up based on the treatment protocol. We used logistic regression and area under the curve (AUC) as a measure of discrimination of lactate and PCT with in-hospital mortality.

**Results**

The in-hospital mortality rate of the 1075 included patients (mean age 66.9 years) was 18.8%. Concerning prognosis, the initial lactate level was a better mortality predictor (AUC 0.64) compared to PCT (AUC 0.55). For follow-up measurements, PCT (AUC 0.75) showed a better discrimination than lactate (AUC 0.73). When looking at biomarker kinetics, PCT increase was more strongly associated with fatal outcomes compared to initial levels alone (AUC 0.74) and was a better predictor compared to lactate kinetics (AUC 0.63). A joint logistic regression model combining follow-up measurements of lactate and PCT-kinetics showed a superior prognostic accuracy (AUC 0.82) compared to these markers alone.

**Conclusions**

Both biomarkers, PCT, and lactate, provide prognostic information in ICU patients with sepsis, particularly when looking at kinetics.

**Fig. 11 (abstract P379):**
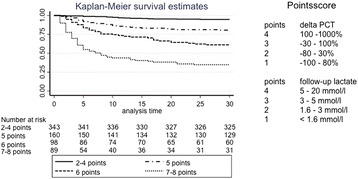
Kaplan-Meier curve over 30-days stratified by score.

## P380 Impact of CRP, procalcitonin and CRP on mortality related with sepsis in ICU

### D Mandaci, G Eren, F Ozturk, N Emir, O Hergunsel

#### Bakirkoy Dr.Sadi Konuk Training and Research Hospital, Istanbul, Turkey

**Introduction**

Sepsis is known to be the leading cause of morbidity and mortality of ICU patients. APACHE 2, SAPS 2 ve SOFA scores are most frequently used for predicting mortality. Herein, it’s aimed to search for the effect of C-reactive protein (CRP), procalcitonin (PCT) and mean platelet volume (MPV) on mortality of sepsis in the ICU.

**Methods**

Retrospectively, 25 sepsis patients admitted to our ICU on May 2016 with at least 5 days of stay in the ICU were searched for APACHE 2, SAPS 2 and SOFA scores. The effect on mortality of CRP, procalcitonin and MPV values on the 1st, 5th and 10th days were analyzed with SPSS version 15 through receiver operating characteristic (ROC) curve analysis. Sensitivity, specificity, positive and negative predictive values were calculated.

**Results**

Patients were between the ages of 18-79 years with a median of 53 and a female-male ratio of 56-44%. ROC analysis revealed that the last day MPV values were highly predictive for mortality (AUC: 0.99, p < 0.01). The cut-off for this value was calculated as 7.73, with a sensitivity of 90%, specificity of 100%, and positive predictive value as100%, negative predictive value as 53%. CRP values on the last day of ICU stay were predictive for mortality as well (AUC: 0.85, p < 0.01) with a cut-off value of 7.55, sensitivity of 92.3% but a specificity of 87.5% (positive predictive value: 92.3% and negative predictive value: 63.6%). Procalcitonin values of neither the first nor the last day of ICU stay were predictive of mortality. APACHE 2, SAPS 2 and SOFA were found to be well correlated with MPV values and the last day CRP values, but not with any of the procalcitonin values.

**Conclusions**

Together with APACHE 2, SAPS 2 and SOFA scores, CRP and MPV values are good predictors of mortality of sepsis in ICU. We consider a further study with a larger sample size to evaluate better the effect of procalcitonin in this issue.

**References**

1. Faix James D. Biomarkers of sepsis. Crit Rev Clin Lab Sci. 2013 Jan; 50(1): 23–36.

2. Tajarernmuang P, Phrommintikul A, Limsukon A, et al. The Role of Mean Platelet Volume as a Predictor of Mortality in Critically Ill Patients: A Systematic Review and Meta-Analysis. Critical Care Research and Practice, 2016. doi:10.1155/2016/4370834

## P381 Serum procalcitonin in cirrhotic patients: a reliable biomarker of bacterial infection?

### S Azaiez, S Khedher, A Maaoui, M Salem

#### Charles Nicolle Hospital, Tunis, Tunisia

**Introduction**

Bacterial infection often leads to decompensated liver cirrhosis in cirrhotic patients. Its diagnosis should be swift, otherwise it is associated with a poor prognosis.

Our aim is to quantitate the various inflammatory markers in cirrhotics with and without obvious infectious disease.

**Methods**

A total of 80 patients admitted due to decompensated liver cirrhosis were included in this study. We explored the key epidemiologic, clinical and biological features of these patients.

**Results**

54 patients (67,5%) were assigned to the infection group of which nine patients (16,6%) had positive blood culture. In this group, the following diagnoses were made : 34 lung infections, 11 spontaneous bacterial peritonitis, nine urinary tract infections, three skin infections, and two gastroenteritis. Three patients had an infection without an identified source.

The mean age for the infected patients (group 1) was 62.8 years and for the uninfected ones 63.4 years (group 2). There were higher levels of C reactive protein (CRP) and procalcitonine (PCT) in group 1 than in group 2 (a mean of 52.7 mg/ L and 1.3 ng/ mL versus 13,3 mg/l and 0,1 ng/mL respectively). The median of CRP for group 1 was 43,15 mg/ L. Infection at admission was associated with significantly higher levels of these proteins (p = 0.000). Leucocyte count was also higher in group 1 (p = 0.04).

49 patients had CRP levels below 43.15 mg/ L, wherein 25 (51%) had infections. In this particular group, CRP was significantly higher in infected patients (p = 0.000), unlike PCT (p = 0,06) and leucocyte count (p = 0.23).

**Conclusions**

Elevated PCT appear only in inflammations of an infectious etiology with systemic signs. Therefore in cirrhotic patients, procalcitonin determination seems appropriate for the diagnosis of severe infections but does not seem more reliable than CRP in other situations.

## P382 The assessment of bacterial load in ICU nosocomial pneumonia

### E Chernevskaya^1^, N Beloborodova^1^, A Bedova^1^, YU Sarshor^1^, A Pautova^1^, V Gusarov^2^

#### ^1^Negovsky Research Institute of General Reanimatology, Moscow, Russia; ^2^Pirogov National Medical and Surgical Center, Moscow, Russia

**Introduction**

Development of new microbial load biomarkers proceeds, but still - there is no perfect one. Procalcitonin (PCT) is commonly used and its level correlates with the extent of microbial invasion. Earlier it was found that aromatic microbial metabolites (AMM) - are associated with the severity and mortality of ICU patients [1, 2]. Also the effectiveness of the treatment can be evaluated by the AMM level [1]. Experimental studies have shown their biological activity [3,4]. The aim of this work is to analyze different criteria of bacterial load in ICU patients with nosocomial pneumonia.

**Methods**

In prospective study 46 patients with nosocomial pneumonia admitted to ICUs were observed in the first day. After liquid-liquid extraction from serum samples 9 phenylcarboxylic acids (benzoic (BA), phenylpropionic, cinnamic, phenyllactic (PhLA), p-hydroxybenzoic, p-hydroxyphenyllactic, p-hydroxyphenylacetic (HPhAA), p-hydroxyphenylpropionic and homovanillic (HVA)) were measured using GC-MS (Trace1310-ISQ, Thermo). DNA was extracted from bronchoalveolar lavage (BAL) samples from 5 of 46 patients for the quantitative detection of nosocomial bacteria by the PCR-real time (IQ5, BIORAD), PCT and presepsin were measured on the 1, 3, 7-9 days after the diagnosis of pneumonia. Spearman’s correlation coefficient was found, data presented as medians with interquartile range (IR, 25-75%) using STATISTICA 10.

**Results**

In serum samples of 46 patients the total concentration of 9 AMM was 3.4 (2.2-17.4) μ M that was higher (p < 0.05) than in healthy donors - 1.59 (1.46-1.85) μ M (n = 20). It correlated with APACHE II - 10 (6-18), SOFA - 3 (1-8) scores and mortality (41%): rs = 0.645, 0.666 and 0.717 respectively, p < 0.01. A closer analysis of 5 patient samples revealed the following correlations: the total concentration of 9 AMM correlated with PCT - 0.580, HVA with PCT and presepsin – 0.810 and 0.709, respectively, PhLA with presepsin-0.770, p-HPhAA with total DNA of bacteria and DNA Enterobactereacea in BAL– 0.635 and 0.724, p < 0.01.

**Conclusions**

The level of aromatic microbial metabolites in blood serum of ICU patients with nosocomial pneumonia reflects the microbial load as well as the quantity of bacterial DNA, PCT and presepsin.

Supported by Russian Science Foundation Grant ^1^15-15-00110

**References**

1. Moroz VV et al. General Reanimatology 12(4): 37–48, 2016

2. Rogers AJ et al. PLoS One 9(1): e87538, 2014

3. Jankowski J et al. J. Am. Soc. Nephrol. 9(7): 1249–57, 1998

4. Beloborodova NV et al Anest. I rean. 61(3): 202–8, 2016

## P383 Procalcitonin elimination during cytokine adsorption therapy in septic shock: a spin-off study of the ACESS trial

### N Öveges^1^, I László^1^, M Forgács^1^, T Kiss^1^, P Hankovszky^1^, P Palágyi^1^, A Bebes^1^, B Gubán^1^, I Földesi^1^, Á Araczki^1^, M Telkes^1^, Z Ondrik^1^, Z Helyes^2^, Á Kemény^2^, Z Molnár^1^

#### ^1^University of Szeged, Szeged, Hungary; ^2^University of Pécs, Pécs, Hungary

**Introduction**

According to in vitro data, levels of pro- and anti-inflammatory mediators can be markedly decreased in septic shock by hemoperfusion using a novel cytokine adsorbent therapy (CytoSorb). However, the capacity and performance of the absorber over time has not yet been investigated. Our aim was determine elimination of procalcitonin (PCT) by the adsorbent in vivo, in patients with septic shock treated with CytoSorb for 24 hours.

**Methods**

The current study is a spin-off part of the ongoing “Adsorption Cytokines Early in Septic Shock”, the ACESS-trial. CytoSorb therapy was commenced early (<48h) after the onset of septic shock and performed for 24 hours. Blood samples were taken from the systemic circulation in every 6 hourly from the beginning (T0) to the end of CytoSorb therapy (T6, T12, T18, T24). Serum PCT, CRP, IL-1, IL-1ra, IL-6, IL-8, IL-10, TNF-α levels were measured. PCT levels were determined from blood taken simultaneously from the pre-, and post-adsorbent samples. The efficacy of PCT elimination was defined at every step by subtracting post-adsorbent (PCTpost) values from pre-adsorbent (PCTpre) values: Δ PCT = PCTpre-PCTpost.

**Results**

Data were obtained from 7 patients and 8 treatments. Pre-adsorbent PCT levels showed a significant decline throughout the study (T0 = 12.2 [5.6-84.2], T6 = 6.8 [4.8-36.5], T12 = 6.7 [4.8-33.2], T18 = 5.9 [4.5-27.3], T24 = 5.1 [3.5-15.7] ng/ml; p = 0.011). Post-adsorbent PCT levels showed a similar pattern (p = 0.012). The efficacy of net PCT elimination (Δ PCT) was most effective at T0 = 11.7 [5.3-78.1] ng/ml, and showed a significant decline over time: T6 = 2.5 [1.4-14.9] ng/ml; T12 = 1.2 [0.3-6.1] ng/ml; T18 = 1.2 [0.5-4.8] ng/ml; T24 = 0.5 [-0.9-3.5] ng/ml; p = 0.004. This corresponded of a median of 90% elimination at T0, 30% at T6 and only 10% at T12 with no further change from T12-T24. This pattern showed consistency in every patient and was independent of the serum levels of PCT.

**Conclusions**

This study is the first to examine PCT elimination over time during CytoSorb therapy. According to these results, PCT elimination showed an exponential decline from 90% to a negligible degree after 12 hours. This phenomenon, on the one hand, shows the early efficacy of CytoSorb therapy, while on the other hand, it raises the question of changing the adsorbent earlier than the current practice of 24 hours.

**Acknowledgements**

NKFIH K116689

ClinicalTrials.gov ID: NCT02288975

## P384 qSOFA (quick SOFA) score, presepsin and procalcitonin for severity assessment in initial sepsis

### E Spanuth^1^, H Ebelt^2^, B Ivandic^1^, R Thomae^3^, K Werdan^2^

#### ^1^DIAneering GmbH, Heidelberg, Germany; ^2^Department of Medicine III, University Clinics Halle (Saale), Martin-Luther-University, Halle-Wittemberg, Germany; ^3^Mitsubishi Chemical Europe, Düsseldorf, Germany

**Introduction**

The SOFA score is associated with an increased probability of mortality in sepsis. Assessment at admission in the ED requires several laboratory variables. The Third International Consensus Definitions for Sepsis and Septic Shock defined the qSOFA, which does not require laboratory tests and can be assessed at patient admission.

Objective: To compare sepsis biomarkers with SOFA and qSOFA to discriminate sepsis, severe sepsis or septic shock and to evaluate the association with increased risk of mortality.

**Methods**

66 Patients admitted to the ED with signs of sepsis and =/>2 SIRS-criteria were included. Severe sepsis and septic shock were defined according to current guidelines. qSOFA score was calculated using the recommended thresholds: respiratory rate =/>22/min, GCS score <15, stystolic blood pressure >100mmHG. Presepsin and procalcitonin were determined using the POC assay PATHFAST Presepsin (PSEP), LSI Medience, Japan and the BRAHMS luminescence immune assay (PCT).

**Results**

SOFA and qSOFA, differentiated significantly between patients with sepsis (n = 30, mortality = 6.6%) and the high-risk group with severe sepsis or septic shock (n = 36, mortality = 36.1%). Discrimination between the groups revealed AUC values of 0.621, 0.627, 0.731, 0.740 and 0.781 for lactate, PCT, qSOFA, PSEP, and the combination qSOFA + PSEP, respectively. 15 patients died during hospitalization. AUC values of mortality prediction were 0.715, 0.558, 0.734, 0.758 and 0.803 for lactate, PCT, qSOFA, PSEP and qSOFA + PSEP, respectively. qSOFA scores =/>2 should identify greater risk of death or prolonged ICU stay. Discrimination between qSOFA <2 and =/>2 revealed AUC values of 0.756, 0.669 and 0.606 for PSEP, lactate and PCT.

Using the threshold =/>2 of qSOFA and =/>500 ng/L of PSEP, the combination qSOFA + PSEP detected 14 non-survivors (93%) and 33 (92%) patients of the high-risk group (n = 36), whereas qSOFA alone detected only 10 non-survivors (67%) and 21 patients of the high-risk group (58%).

**Conclusions**

The results demonstrated that the qSOFA score is not a standalone criterion for risk stratification in sepsis at admission. Simultaneous assessment by combining qSOFA and PSEP improved the validity significantly. The POC assay PATHFAST Presepsin showed superior performance compared to lactate and PCT.

**References**

Singer M et al. The Third International Consensus Definitions for Sepsis and Septic Shock (Sepsis-3). JAMA. 2016;315(8):801–810.

## P385 Role of presepsin compared to c-reactive protein in sepsis diagnosis and prognostication

### M. El-Shafie^1^, K Taema^1^, M El-Hallag^1^, A Kandeel^2^

#### ^1^Cairo University, Cairo, Egypt; ^2^El-Sahel Teaching Hospital, Cairo, Egypt

**Introduction**

Early identification of sepsis and its differentiation from non-infective SIRS are important for sepsis outcome. We intended to evaluate the use of presepsin in differentiating sepsis from non-infectious SIRS and its prognostic value compared to CRP.

**Methods**

We included 31 patients (median age 60 year old, 16 males) admitted with SIRS to El-Sahel Teaching Hospital, Egypt after excluding 21 patients with preadmission corticosteroids therapy, blood transfusion, immunosuppressive illness, and ICU length of stay (ICU-LOS) less than 24-hours. Patients were classified into non-infective SIRS group (13 patients) and sepsis group (18 patients). Presepsin, CRP and SOFA score were measured on admission and on days 2 and 4 of admission. The outcome parameters studied were ICU length of stay (ICU-LOS) and in-hospital survival.

**Results**

Apart from temperature and AST which were significantly higher in sepsis group, the two groups were comparable. All the presepsin levels and CRP on days 2 and 4 were significantly higher in sepsis than in SIRS groups. The ICU-LOS was positively correlated with all the presepsin levels and with the CRP levels on days 2 and 4. All Presepsin values were significantly higher in survivors while none of the CRP levels were significantly different in survivors and non-survivors. The decrease of presepsin over time was significantly associated with better survival. It was found to be 70% sensitive and 91% specific for predicting survival in SIRS patients. This relation was not found in CRP levels.

**Conclusions**

We concluded that the presepsin may be used for early differentiation between sepsis and non-infectious SIRS and predict higher mortality.

## P386 Urinary albumin/creatinine ratio as an early predictor of outcome in critically-ill septic patients

### O Tayeh^1^, K Taema^1^, M Eldesouky^1^, A Omara^2^

#### ^1^Cairo University, Cairo, Egypt; ^2^Electricity Hospital, Cairo, Egypt

**Introduction**

Several cumbersome scoring systems were developed for prognosis and outcome prediction in sepsis. We intended in this study to evaluate the urinary albumin/creatinine ratio (ACR) as a prognostic predictor in sepsis.

**Methods**

We included 40 adult septic patients in a prospective observational study. We excluded patients with preexisting chronic kidney disease or diabetes mellitus. After clinical evaluation, urine spot samples were collected on admission and 24 h later for ACR1 and ACR2. Admission APACHE IV score and the highest recorded SOFA score of their daily estimation were considered. We also evaluated the need for mechanical ventilation, inotropic and/or vasoactive support, renal replacement therapy (RRT), and in-hospital mortality.

**Results**

In a population with 63 (55–71) year old with 29 (72.5%) males, we found that the ACR2 is correlated with the SOFA score (r =0.4, P = 0.03). SOFA was higher in patients with increasing ACR [14(4.8–16.8) vs 5(3–8), P = 0.01]. None of the ACR measures was correlated with APACHE IV score. ACR2 was higher in patients who needed mechanical ventilation and inotropic and/or vasoactive support [140(125–207) and 151(127–218) mg/g respectively] compared to [65(47–174) and 74(54–162) mg/g], P = 0.01 and 0.009. None of the measured parameters was related to the need of RRT. ACR1, ACR2, APACHE IV and increasing ACR were predictors of mortality. The AUC for mortality prediction was largest for APACHE IV (0.90) then ACR2 (0.88). ACR2 of 110.5 mg/g was 100% sensitive and 86% specific to predict mortality.

**Conclusions**

We concluded that the urinary ACR may be used as a simple test for prognosis and mortality prediction in sepsis.

## P387 Syndecan-1 (SDC1) and sphingosine-1-phosphate (S1P) are inversely associated in sepsis

### MS Winkler^1^, M Holzmann^1^, A Nierhaus^2^, E Mudersbach^3^, E Schwedhelm^3^, G Daum^4^, S Kluge^4^, C Zoellner^1^

#### ^1^Dep. of Anaesthesiology, University Medical Center Eppendorf, Hamburg, Germany; ^2^Dep. of Intensive Care Medicine, University Medical Center Eppendorf, Hamburg, Germany; ^3^Institute of Clinical Pharmacology and Toxicology, University Medical Center Hamburg-Eppendorf, Hamburg, Germany; ^4^Department of Vascular Medicine, University Medical Center Hamburg-Eppendorf, Hamburg, Germany

**Introduction**

Sepsis is characterized by endothelial dysfunction. Crucial for endothelial function is the glycocalix (GLY), a carbohydrate-rich layer on the endothelial surface, which regulates vascular permeability, microcirculation and mechanotransduction. The GLY is anchored to the endothelium by glycoproteins and proteoglycans including Syndecan-1 (SDC1). Shedding of SDC1 has been observed in situations when the endothelium is damaged. Another key regulator of vascular permeability is sphingosine-1-phosphate (S1P) that upon binding to endothelial S1P receptor 1 strengthens barrier function. It has been observed that S1P is able to protect the GLY by inhibiting matrix-metalloproteinase (MMP) activity. We have recently observed decreased S1P levels in patients with sepsis, and here, addressed the question whether decreased S1P levels are associated with increased concentrations of SDC1 in serum of septic patients in the cohort we have previously studied for S1P levels [1].

**Methods**

The local ethics committee approved this study by acceptance of an amendment to our previous protocol. 59 ICU patients with sepsis or septic shock were enrolled. Blood samples were drawn at day 1 after admission and serum was prepared. Soluble SDC1 was measured by ELISA and S1P by mass spectrometry. The SOFA Score was used to assess disease severity. Twenty health controls were included.

**Results**

SDC1 are increased and S1P levels are significantly decreased in sepsis patients when compared to controls. Highest SDC1 levels and lowest serum-S1P levels were found in patients with septic shock. The mean serum SDC1 concentration was 33 ± 24 ng/mL in controls, 196 ± 162 ng/mL in patients with sepsis and 260 ± 164 ng/L in patients with septic shock. Using linear regression analyses, S1P and SDC1 were found inversely associated (P < 0.001). The SDC1/S1P ratio was positively associated with the SOFA score (P < 0.01). By generating ROC curves for the SDC1/S1P ratio and the SOFA score to indicate septic shock similar areas under the curve (AUCs) were found with 0.77 for the SDC1/S1P ratio and 0.80 for the SOFA score.

**Conclusions**

We found increased SDC1 levels, which were associated with decreased serum-S1P levels in septic patients. SDC1 and S1P are inversely correlated and the ratio of SDC1/S1P reveals as valuable prognostic marker for septic shock.

**References**

1. Winkler, M.S., et al., Decreased serum concentrations of sphingosine-1-phosphate in sepsis. Crit Care, 2015.

## P388 Serum sphingosine-1-phosphate (S1P) levels are rapidly decreasing during coronary artery bypassgraft

### G Greiwe^1^, H Sawari^2^, E Schwedhelm^3^, A Nierhaus^4^, S Kluge^4^, J Kubitz^1^, R Jung^5^, G Daum^6^, H Reichenspurner^2^, C Zoellner^1^, MS Winkler^1^

#### ^1^Dep. of Anaesthesiology, University Medical Center Eppendorf, Hamburg, Germany; ^2^Dep. of Cardiovascular Surgery, University Heart Center Hamburg-Eppendorf, Hamburg, Germany; ^3^Dep. of Clinical Pharmacology and Toxicology, University Medical Center Hamburg-Eppendorf, Hamburg, Germany; ^4^Dep. of Intensive Care Medicine, University Medical Center Hamburg-Eppendorf, Hamburg, Germany; ^5^Dep. of Laboratory Medicine, University Medical Center Hamburg-Eppendorf, Hamburg, Germany; ^6^Dep. of Vascular Medicine, University Heart Center Hamburg-Eppendorf, Hamburg, Germany

**Introduction**

Postoperative inflammation is associated with a sepsis like syndrome including endothelial barrier disruption, volume depletion and hypotension. Postoperative inflammation is especially associated with surgical procedures which require extracorporeal circulation. Sphingosine-1-phosphate (S1P) is a signaling lipid regulating permeability and vascular tone. In animal models S1P and S1PR1 specific agonists were able to limit trauma induced barrier disruption. Moreover in septic humans decreased serum-S1P levels could be identified as marker for sepsis severity. Low levels were predictive for septic shock. Here we were interested if S1P levels are altered by surgical trauma induced by cardiac bypass surgery.

**Methods**

26 patients with coronary heart disease planned for coronary bypass surgery were prospectively enrolled in this study. Serum samples were drawn pre-, post- procedure and on day 1 and day 4 after surgery. S1P concentration in μM was quantified by mass spectrometry. In addition we analyzed potential S1P sources: Red blood cells (RBC) and platelets. We further quantified levels of other inflammatory markers: C-reactive protein, procalcitonin, interleukin- 6 and von-Willebrand antigen.

**Results**

Mean serum-S1P levels in patients before the procedure were 0.74 ± 0.2μM. S1P post-levels were lowest directly after surgery and dropped by 50% (0.37 ± 0.11μM). In recovery serum-S1P levels were increased to 0.60 ± 0.13μM on day 1 and was higher than pre levels on the fourth day 0.82 ± 0.18μM. There was no difference observed if patients were operated with or without extracorporeal circulation. In both groups the serum-S1P kinetics were similar, with significantly decreasing levels during surgery and increasing levels in recovery. In a correlation analysis serum-S1P levels correlated with its sources red blood cell (RBC) and platelet count. We did not observe a significant correlation of S1P levels with the dosage of vasopressors in the intraoperative phase or other inflammatory markers.

**Conclusions**

Cardiac bypass surgery induces a rapid decrease in serum-S1P levels. S1P might be a marker for early inflammation postoperative systemic inflammation, which is independent from other commonly used inflammatory markers.

## P389 Can the biochemical and immunoinflammatory profile of patients sustaining high-energy tibial fractures predict the development of acute posttraumatic osteomyelitis?

### M Groznik^1^, A Ihan^2^

#### ^1^University Clinical Centre Ljubljana, Ljubljana, Slovenia; ^2^University of Ljubljana | Faculty of Medicine, Ljubljana, Slovenia

**Introduction**

Early diagnosis of acute posttraumatic osteomyelitis (POM) is of vital importance to avoid devastating complications. Lack of specific and sensitive test as in myocardial infarct makes it difficult to diagnose POM. Serum inflammatory markers are not able to differentiate between response to infection and the host response to non-infection insult.

**Methods**

The prospective nonrandomised cohort study included 115 patients after high-energy injury to cruris required to be primary surgical treated. Values of biochemical and immunoinflamatory profile were measured on admission (ADD), first postoperative day (POD1) and fourth-postoperative day (POD4). Collected data from laboratory measurement and inpatient and outpatient medical records were analysed by descriptive statistics, univariate logistic regression and multivariate logistic regression with Firth correction and penalized regression.

**Results**

The best predictors of PO on admission are T regulatory cells CD4 + CD25+, CRP and WBC counts, on POD1: WBC counts, CRP and PCT and on POD4: CRP, albumins, PCT and WBC counts. Performing logistic regression with Firth correction and multivariate penalized regression we found out that we can best predict development of PO by measuring CRP on ADD, POD1, POD4, PCT on ADD and POD4, albumins on POD4 and assessment of extent of soft tissue damage, transfusion rate, need of conversion primary external fixation to intramedullary (IM) nailing or locking plate fixation and patient’s physical status ASA 3-4 according to the American Society of Anesthesiologists. Our LASSO predictive model with cross-validated parameters has shown good performance (AUC = 0,75).

**Conclusions**

We can improve prediction of POM development by using perioperative inflammatory biomarkers PCT and CRP in combination with postoperative albumins and considering independent risk factors.

## P390 Coenzyme q10 in acute influenza infection

### LW Andersen, M Chase, MJ Holmberg, A Wulff, MN Cocchi, MW Donnino

#### Beth Israel Deaconess Medical Center, Boston, MA, United States

**Introduction**

The goal of this investigation was to determine if acute influenza infection is associated with depletion of Coenzyme Q10 (CoQ10) and to determine any associations between CoQ10 levels and severity of illness and inflammatory biomarkers. CoQ10 is a key cofactor in the mitochondrial respiratory chain and may be depleted in acute critical illness. Prior investigations have found a correlation between depleted CoQ10 levels in patients with both septic shock and post-cardiac arrest and the inflammatory cascade. Statin medications have also been associated with decreased CoQ10 levels.

**Methods**

We analyzed serum CoQ10 concentrations of patients with acute influenza infection who were enrolled in a randomized clinical trial administering atorvastatin or placebo. Patients were enrolled at a single urban tertiary care center over 3 influenza seasons (12/2013 to 5/2016). Blood samples were obtained at enrollment prior to administration of any study drug. Healthy adult controls were used for comparison. We used Wilcoxon rank sum test to compare CoQ10 levels between influenza patients and controls. Correlations between CoQ10 levels and other inflammatory biomarkers and patient-reported severity of illness were assessed using Spearman correlation coefficient.

**Results**

We analyzed CoQ10 from 50 patients with influenza and 29 healthy controls. Overall, patients with acute influenza infection had lower levels of CoQ10 compared to controls (0.61 ± 0.31 vs 0.77 ± 0.23 ug/mL, p = 0.004). There were significantly more patients in the influenza group with low CoQ10 levels compared to controls (48% vs 7%, p = 0.0001). There were significant correlations between CoQ10 levels in influenza patients and several inflammatory biomarkers: interleukin-2 (IL-2) (r = -0.297, p = 0.038), tumor necrosis factor alpha (TNF-α) (r = -0.352, p = 0.013) and vascular endothelial growth factor (VEGF) (r = 0.381, p = 0.007). There was no correlation between influenza severity of illness score at time of enrollment and CoQ10 levels.

**Conclusions**

We found that CoQ10 levels were significantly lower in patients with acute influenza infection as compared to controls and that CoQ10 levels had a significant correlation with several inflammatory biomarkers. Further study is warranted to define these relationships and identify potential targets for therapy in acute influenza.

## P391 The research on effects of heparin-binding protein levels on early diagnosis in sepsis, severe sepsis and septic shock

### C Balci

#### Health Sciences University, Kocaeli Derince Traning Hospital, Kocaeli, Turkey

**Introduction**

Sepsis is the body’s systemic inflammatory response to infection and can progress to severe sepsis, septic shock and ultimately multiple organ failure. Rapid detection of severe sepsis and septic shock is crucial for good outcome. Heparin- binding protein (HBP) is a multifunctional inflammatory mediator. HBP has been demonstrated in various infectious diseases caused by a wide array of bacteria. Procalcitonin and heparin -binding protein value may be useful for early diagnosis in sepsis, severe sepsis and septic shock patients in intensive care unit. The present study was conducted to determine the heparin- binding protein, procalcitonin level at early diagnosis in patients with sepsis, in comparison with C-reactive protein, IL-6 and TNF-alfa.

**Methods**

This was a prospective, observational of ICU patients with suspected infection, conducted Health Sciences University, Kocaeli Derince Traning Hospital’s ICU. The study was conducted aver a 7- month period between January and July 2016. Blood samples were taken on the first and second day of hospitalization, and the seventh day, on day of discharge or on the day of death.

**Results**

Heparin-binding protein, PCT, TNF-alaf- IL-6 and C-reactive protein levels increased in parallel with the severity of the clinical condition of the patient. Heparin binding protein exhibited a greatest sensitivity and specificity in differentiating patients with sepsis. Also, when comparing all the data; HBP was the best predictor of early diagnosis sepsis and organ dysfunction (Our sturdy continues).

**Conclusions**

In the present study heparin binding protein was found to be a more accurate diagnostic parameter for differentiating sepsis and organ dysfunction.Heparin binding proetin may be helpful in the follow up of sepsis patients. HBP measurement in the ICU may help monitor treatment in patients with sepsis, severe sepsis and septic shock.

**References**

1. Herwald H, Cramer H, Mörgelin M, et al: M protein, a classical bacterial virulence determinant, forms complexes with fibrinogen that induce vascular leakage. Cell 2004; 116:367–379.

2. Linder A, Soehnlein O, Akesson P: Roles of heparin-binding protein in bacterial infections. J Innate Immun 2010; 2:431–438.

## P392 A new scoring system for early diagnosis of ventilator-associated pneumonia: LUPPIS

### M Haliloglu, B Bilgili, H Bilgin, U Kasapoglu, I Sayan, M Süzer, L Mulazımoglu, I Cinel

#### Marmara university hospital, Istanbul, Turkey

**Introduction**

Ventilator-associated pneumonia (VAP) is the most common nosocomial infection in critically ill patients and associated with increased mortality and morbidity, and prolonged mechanical ventilatory support and length of stay in the intensive care unit. The Clinical Pulmonary Infection Score (CPIS) based on chest X-ray has been developed to facilitate clinical diagnosis; however, this scoring system has a low diagnostic performance. In this study, the authors developed a new scoring system for early diagnosis of VAP called “Lung Ultrasound and Pentraxin-3 Pulmonary Infection Score (LUPPIS)” which is based on pentraxin-3 (PTX-3) levels and lung ultrasonography and evaluated the performance of this new scoring system.

**Methods**

This single center, observational, prospective study included 78 patients, who received therapy as an inpatient in the Intensive Care Unit between January 2015 and April 2016 and who were suspected of having VAP. At the day of study, an endotracheal aspirate was obtained for Gram staining and culture. PTX-3, procalcitonin (PCT), and other biomarkers were recorded. The diagnosis of VAP was confirmed according to either culture positivity or in culture negative patients, all clinical criteria and initiated or modified antibiotic regimen within 48 hours.

**Results**

No significant differences were found between groups with respect to age, mechanical ventilation time, APACHE II score, and SOFA score (p > 0.05). PCT and PTX3 levels were significantly higher in the VAP (+) group (p < 0.001 and p < 0.001, respectively). The threshold for LUPPIS in differentiating VAP (+) patients from VAP (-) patients was >7 according to the highest Youden Index. In predicting VAP, LUPPIS > 7 (sensitivity of 84%, specificity of 87.7%) was superior to CPIS > 6 (sensitivity of 40.1%, specificity of 84.5%).

**Conclusions**

Early diagnosis of VAP currently remains challenging due to lack of a validated gold standard diagnostic method. The diagnosis and follow-up of pulmonary infections rely on the chest x-ray, although this is not feasible for critically ill patients in daily practice. Lung ultrasound (USG) is an important, simple, non-invasive, and cost-effective technique used in the diagnosis and differentiation of lung pathologies. Considering the overlap between sensitivity analysis and confidence intervals in the present study, LUPPIS appears to provide better results in the prediction of VAP compared to CPIS, and the importance of lung USG and PTX-3 is emphasized, which is a distinctive property of LUPPIS.

## P393 An evaluation of the national early warning score in patients with unscheduled admissions to the intensive care unit

### V Patel, S Shah, P Parulekar

#### Royal Sussex County Hospital, Brighton, United Kingdom

**Introduction**

The National Early Warning Score (NEWS) is a UK-wide system measuring physiological variables to standardise evaluation of acute illness. We hypothesise that the NEWS may provide novel prognostic information for patients admitted to ICU.

**Methods**

We retrospectively assessed the NEWS and outcomes of emergency admissions in ward-based patients to the critical care unit in a UK tertiary care hospital. Data was obtained via patient notes and electronic records over a 6-month period. We evaluated: the NEWS trigger, the seniority of ward doctor reviewing the patient prior to ICU admission, Medical Emergency Team (MET) involvement, organ support requirements and mortality outcomes. in two distinct cohorts, NEWS > =7 versus NEWS < =6.

**Results**

50 sets of notes were analysed. In the NEWS > =7 group, 65% of patients were seen by a ward registrar or above prior to critical care involvement. Only 42% of patients admitted to the ICU with NEWS > =7 triggered a MET alert, below the national standard. Over 90% of patients in both groups required at least one form of organ support, with a preponderance of basic cardiac and renal support in the NEWS < =6 group. The one-week mortality was higher in the NEWS > =7 group at 13% versus 4%, but 1-year mortality difference was negligible.

**Conclusions**

The RCP guidance for triggering an emergency review is poorly adhered to in our hospital. The necessity for organ support and one-year mortality were similar in both groups, suggesting that patients with NEWS < =6 are as vulnerable as the higher scoring group. This highlights a need to be identified more accurately. We propose the NEWS is combined with a nurse led observational “level of concern” score, supporting earlier referral to a senior physician.

**References**

National Early Warning Score: Standardising the assessment of acute illness severity in the NHS. Report of a working party. London: RCP, 2012

**Table 2 (abstract P393).** See text for description

	Percentage 1 week mortality	Percentage 1 year mortality
NEWS ≤ 6	4	26
NEWS ≥ 7	13	25

Legend : Difference in percentage mortality based on NEWS

**Fig. 12 (abstract P393).**
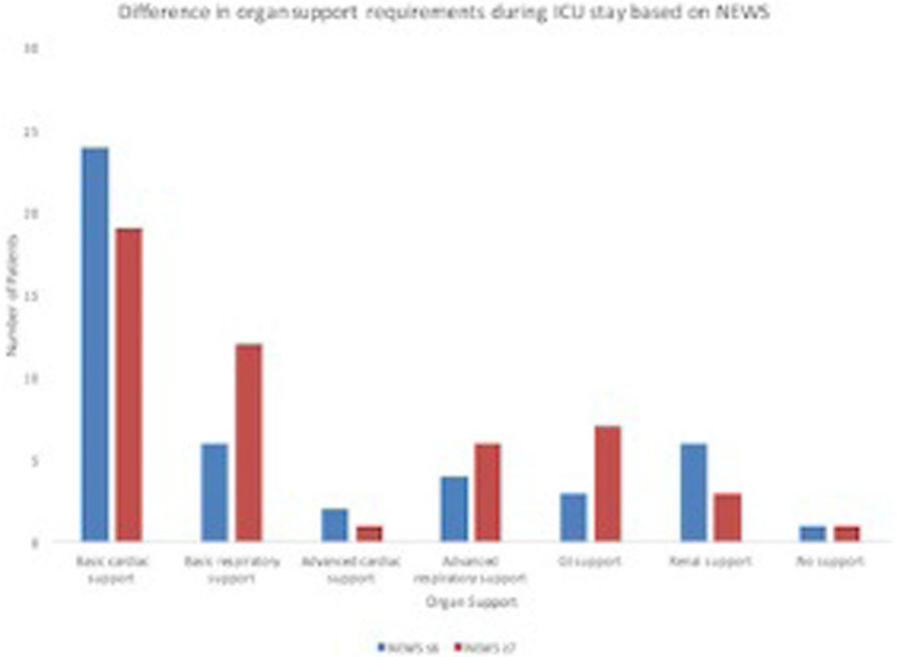
Difference in organ support requirements during ICU stay based on NEWS

## P394 National early warning scores (news) in sepsis – how does this score perform against other available markers routinely applied in the emergency department?

### C Minton, J Patel, C Ejimofo, H Choi

#### University Hospital Lewisham, London, United Kingdom

**Introduction**

Several scoring systems are used to risk stratify patients and alert staff to clinical deterioration. In the UK, the most widely used of these is the NEWS, a scoring tool based on physiological observations. There is increasing evidence that NEWS may be a useful diagnostic and prognostic tool in sepsis [1]. A novel biochemical extension to the NEWS, using parameters from a blood gas (the metabolic score) was found to predict organ failure and death within 48 hours of admission [2].

We compared the predictive ability of NEWS with metabolic score for morbidity and mortality outcomes in patients with sepsis.

**Methods**

This retrospective observational study involved adult patients presenting to the emergency department of University Hospital Lewisham (UK) from September 2015-August 2016 with sepsis requiring inpatient care. Demographic data was gathered from the hospital electronic patient record system. NEWS was calculated from physical observations taken during the patients’ triage. The presence of co-morbidities and metabolic score were collected using a pro forma (completed by the clinician managing each case).

The primary outcomes were evidence of organ failure, escalation of care and death within 48 hours of admission. This information was gathered from the electronic patient record system.

**Results**

Data was gathered from 140 patient attendances (mean age = 65 years [SD = 19.98], male = 65). 61 patients were identified as having evidence of organ failure, 12 patients required escalation of care and 22 patients died.

After controlling for demographic data, NEWS score at triage was predictive for the development of organ failure (p = 0.002) and death (p = 0.003) however it held no predictive value for escalation of care (p = 0.045). Metabolic score was predictive for death (p = 0.013) and the need for escalation of care (p < 0.001), however it was not predictive for the development of organ failure (p = 0.65).

**Conclusions**

Neither the NEWS or metabolic score can accurately predict the development of organ failure, escalation of care and death. We suggest that a combination of these scores should be used to aid risk stratification of septic patients presenting to the emergency department

**References**

1– Smith G et al. Resuscitation 84:465–70, 2013

2– Jafar A et al. Europ J Emerg Med 23: 130–6, 2016

## P395 Sepsis-3 definitions predict hospital mortality better than sepsis-2 in oncological patients in a developing country: it’s time to move forward.

### R Costa, P Caruso, P Nassar

#### AC Camargo Cancer Center, Sao Paulo, Brazil

**Introduction**

We compared the new criteria of sepsis (Sepsis-3) with the previous criteria (Sepsis-2) to predict hospital mortality in oncological patients of a Brazilian Cancer Hospital. Any improvement on sepsis treatment shall begin with the early detection, especially in high-risk populations as the oncological patients in developing countries. Any diagnosis criteria that delay the diagnosis will impact on patients’ outcomes, especially in mortality.

**Methods**

Retrospective study conducted between 2009 and 2014 in a 55-bed ICU of a tertiary and teaching Cancer Hospital. We included all oncological patients admitted in the ICU that received the diagnosis of sepsis, severe sepsis or septic shock at the ICU admission by the attending physician. Mortality was compared across categories of both Sepsis-2 (sepsis, severe sepsis, shock septic) and Sepsis-3 definition (infection, sepsis, septic shock). A p value < 0.05 was considered statistically significant.

**Results**

The medical records of 487 patients were collected from a prospectively recorded database. Mean age was 60 + 14 years, median SOFA was 7 + 7, respiratory tract infection was the most common cause of infection (21%, 101 patients), 398 patients (82%) presented solid tumor, 89 (18%) hematological malignancies and 297 (62%) had metastasis, median ECOG was 2 + 1.3 and 285 patients (59%) died in hospital. The mortality rate was progressively higher across categories of sepsis defined by Sepsis-3 consensus: infection with no organ dysfunction (28%); sepsis (55%) and septic shock (69%) (p < 0.001). There was no statistically significant difference among the categories of Sepsis-2 definitions, neither when they were analyzed separately: sepsis (50%); severe sepsis (57%) and septic shock (62%) (p = 0.2). One-hundred and fifteen patients (24%) did not meet sepsis criteria according to the Sepsis - 2 consensus but were diagnosed by ICU physicians as such. This group also showed a high mortality rate (49%).

**Conclusions**

In oncological patients of a Cancer Hospital located in a developing country, Sepsis 3 criteria were accurate in stratifying hospital mortality and in some aspects superior to the definitions previously used.

**References**

1. Kaukonen K et al.: N ENG JMED 2015; 371 (17): 1–10

2. Seymour et al.: JAMA 2016; 315 (8): 762–774

## P396 Combining usage of national early warning score (NEWS) and rapid response team (RRT) in post HSCT patients might contribute to prognosis

### J Fu^1^, J Jin^1^, Y Xu^1^, J Kong^1^, D Wu^1^, A Yaguchi^2^

#### ^1^The First Affiliated Hospital of Suzhou University, Suzhou, China; ^2^Tokyo Women’s Medical University Hospital, Tokyo, Japan

**Introduction**

Patients with early stage of post hematopoietic stem cell transplantation (HSCT) have a great risk of infectious complication. Sepsis contributes to morbidity and mortality in those patients. Therefore, it’s crucial to early detect sepsis and to treat with rapidly proper management. Our hypothesis is national early warning score (NEWS) and rapid response team (RRT) is useful to early detect and resuscitate sepsis in HSCT patients.

**Methods**

All post HSCT adult patients in sterile room unit who developed persistent fever for 3 days in our university hospital from January to December 2014 were included in this retrospective observational study. Patients were divided into two groups, 1) the control group and 2) the intervention group. Patients in the control group were treated with a local routine protocol. Patients in the intervention group had a local routine protocol treatment and were further evaluated with NEWS and RRT was activated simultaneously. RRT was activated when total NEWS score was exceeded 7 or individual item score was exceeded 3. The 90-day mortality, the number of ICU patients, SOFA score at ICU admission, the number of patients with vasopressor, the number of patients required mechanical ventilation, and days in post HSCT sterile room unit were compared between two groups. Values were expressed as mean ± SD. Data were analyzed by Mann–Whitney U-tests or Χ test. A p < 0.05 was considered as statistically significant.

**Results**

There were 66 patients (35 men, 31 women; age median 37 (18-62)) in this study. The number of patients who admitted to ICU (6 vs. 3), SOFA score at ICU admission (7.6 ± 1.3 vs. 5.2 ± 1.2), the number of patient who required vasopressor (8 vs. 4) and the number of patient who required mechanical ventilation (6 vs. 3) were statistically higher in the control group than in the intervention group (p < 0.05). There were no significant differences in sex in 90-day mortality (17.6% vs. 15.6%) and days in post HSCT sterile room unit (17.4 ± 2.4 vs. 16.7 ± 2.8).

**Conclusions**

The NEWS monitoring and RRT did not statistically improved 90-days mortality in this study. However, the strategy using NEWS and RRT could contribute to early detect deteriorations and to timely ICU care in post HSCT patients who admitted in a special sterile room.

## P397 Comparing criteria and scoring systems in ICU sepsis referrals

### A Klonis, S Ganguly

#### New Cross Hospital, Wolverhampton, United Kingdom

**Introduction**

Following publication of the Sepsis-3 review [1], we aimed to evaluate the awareness and use of the new qSOFA criteria for assessing patients with suspected sepsis and the use of SOFA scoring for subsequent evaluation. We also aimed to assess the correlation between MEWS and qSOFA, and the strength of the 24hr/48hr/mean/maximum SOFA and APACHE2 as predictors of mortality when compared to patient outcome.

**Methods**

This is a retrospective cross-sectional study over a period of 3 months. The patients’ electronic records were reviewed for the use and documentation of qSOFA, SOFA, or any other clinical parameters to justify sepsis requiring ICU input. If not present, the SIRS, qSOFA, 24hr, 48hr, mean & maximum SOFA scores were then calculated. Outcome (discharge vs death) was tested against the mortality risk scores. Inter-test agreement independent of outcome was also analysed. Pearson correlation and binary logistic regression analysis was used to assess for statistical significance.

**Results**

Of the 214 patients admitted over the three-month period, 81% were admitted with a SIRS score of > =2 regardless of diagnosis. 33/214 patients (15.42%) were referred and admitted due to sepsis. None had SIRS or qSOFA documented either prior to or during their ICU admission. All had MEWS, APACHE2 and SOFA scores done only on ICU admission. They all had a retrospectively calculated admission qSOFA of 2 and their 24hr SOFA scores ranged between 5 and 16. Two patients admitted with sepsis had SOFA scores of 10 and 16 respectively, a qSOFA score of 2, but only 1 SIRS criterion. Three patients had a MEWS of 2, nine between 4-5 and twenty-two between 6-20; 94% of patients with sepsis admitted to ICU had MEWS of > =4. The top 5 clinical parameters used to justify ICU input due to sepsis were: reduced GCS, hypotension, tachycardia, pyrexia and Type 1 respiratory failure. The strongest correlation for patient outcome was with 48hr SOFA (p < =0.01), followed by mean SOFA (p < =0.01) and APACHE-II (p < =0.05). The best agreement between the mortality prediction scores was with APACHE2 and the mean SOFA score (p < =0.01), independent of outcome.

**Conclusions**

Despite the dichotomy of approach to sepsis, clinical judgement has remained constant. We observed that the 48hr SOFA had the best correlation with patient outcome. All septic patients had a qSOFA of 2, correlating well with a MEWS of > =4 seen in the majority of septic patients prior to their ICU admission, however we agree with Vincent et al [2] that until qSOFA is validated further, it should be used as a guide and not a replacement of SIRS.

**References**

1. Singer et al. Sepsis-3. JAMA. 2016

2. Vincent et al. qSOFA does not replace SIRS in the definition of sepsis. Critical Care 2016

## P398 Automated microscopy for rapid directed antibiotic treatment of sepsis

### M Kollef, C Burnham, B Fuller

#### Washington University School of Medicine, St Louis, Missouri, United States

**Introduction**

Antibiotic resistance is increasing in frequency due to higher rates of inappropriate antimicrobial therapy and empiric use of broad-spectrum antibiotics.

**Methods**

We employed a real-time multiplexed automated microscopy system (ID/AST; Accelerate Diagnostics, Tucson, Arizona) capable of evaluating antibiotic susceptibility and resistance directly from positive blood culture broth in septic patients using automated phenotypic growth pattern analysis.

**Results**

**Fig. 13 (abstract P398).**
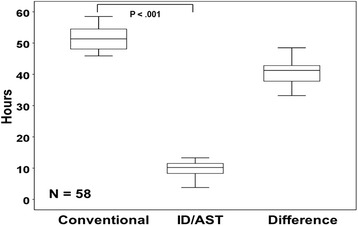
See text for description

Pathogens included Klebsiella species (N = 17), E. coli (N = 16), Enterobacter species (N = 7), P. aeruginosa (N = 7), other Gram-negative species (N = 10), and Candida species (N = 11). Mortality was greater for patients treated with an inactive initial regimen (63.2% versus 6.5%; P < .001). Antimicrobial de-escalation occurred in 41 (63.1%). Time to patient identification and antimicrobial susceptibility using conventional methods was 51.4 hours [48.0, 54.6] versus 10.2 hours [8.3, 11.5] for ID/AST (see Fig. 13). For patients receiving an inactive regimen, ID/AST would have potentially allowed appropriate therapy to be administered a median of 35.8 hours sooner; while de-escalation could potentially have occurred 41.1 hours sooner.

**Conclusions**

The ID/AST system provided accurate pathogen identification and susceptibility more than 1 day sooner compared to standard blood culture processing.

## P399 Comparison of the sonication method to roll plate method to diagnose central vascular catheter colonization and catheter–related infection in patients of ICU: a randomized prospective study

### A Mavrommati, D Chatzilia, E Salla, E Papadaki, S Kamariotis, S Christodoulatos, A Stylianakis, G Alamanos

#### Alamanos KAT hospital, Kifisia, Greece

**Introduction**

Catheter-related bloodstream infection (CRBSI) requires a positive blood culture and a positive result by the semiquantitative culture method (SQC) or the quantitative sonication method (SON). Our aim is to compare the yields of both techniques to detect the colonization and infection, in short and long term catheters in ICU patients.

**Methods**

We prospectively studied central venous antimicrobial catheter. Both methods were performed on tips cut into 2 equal segments each, to avoid loss of microbial colonies during serial examination and contamination. SQC method was performed by rolling the tip on a Columbia agar plate. Sonication was applied by placing the tip in 5 ml of 0.9NaCl, sonicating for 5 min, vortexing for 15s and culturing 0.1 mL of the fluid on a Columbia agar plate too. The cutoff for tip colonization was 15 cfu for SQC and 100 cfu for SON.

**Results**

112 catheter tips (81long, 31short term) from 73 patients were included. The overall catheter tip colonization was 38.4% (43/112). The SON method detected colonization in 39/43 (90.6%) and SQC in 25/43 (58.1%) catheters p < 0.01). 18/112 patients (16%) had a definite CRBSI according to the current guidelines. 17/18 (94.4%) were detected by SON method and 9/18 (50%) by SQC method. Most commonly bacteria were Enterobacteriaceae, isolated by SON in 54.5% and by SQC in 45.5%, followed by coagulase-negative staphylococci in 22.7% and 11.4% respectively. SON detected Candida spp. in 8.1% while SQC failed.

**Conclusions**

Our findings, unlike previous studies, imply superiority of the SON method over SQC, in detection of catheter colonization and CRBSI. A hypothetical explanation is based on the prolonged sonication time of 5 min (instead of the usual 1 min), which probably enhances detachment of gram (-) bacteria that as endoluminal bacteria are more difficult to isolate by SQC. Especially for long term catheters, where colonization by gram (-) bacteria is most often, according to the literature, the sonication method may be prove particularly useful for colonization detection and CRBSI diagnosis. Further investigation is envisaged to explore the potential of the method.

**References**

1. Slobbe L et al.:J Clin Microbiol. 2009;47:885–888.

2. Erb S et al.:Clin Infect Dis 2014;59:541–544.

3. Mermel LA et al.: Clin Infect Dis 2009; 49 :1–45

## P400 Retrospective analysis of microbiological samples at ICU admission

### M Simoes, E Trigo, N Silva, P Martins, J Pimentel

#### Centro Hospitalar Universitario de Coimbra, Coimbra, Portugal

**Introduction**

Hospital­-acquired (or nosocomial) infections are important causes of morbidity and mortality despite improved antimicrobial therapy, supportive care, and prevention. However, due to aging, comorbidities and repeated contact with healthcare facilities, many patients are already infected with multidrug-resistant (MDR) pathogens at the time of ICU admission.

**Methods**

Retrospective analysis of microbiologic exams taken at admission in a ICU over one year, using data obtained from microbiology laboratory. Characterization of positive samples regarding pathogens identified and clinical setting (community vs hospital acquired).

**Results**

2788 samples were obtained in 510 patients admitted to the ICU during 2015. Regarding the type of sample, 23.6% were tracheal aspirate cultures, 57.6% blood cultures and 18.7% urine cultures. We collected 560 positive samples, obtained from 280 patients. From the tracheal aspirate cultures, 38.3% were positive. The most frequent pathogen was S. aureus, found in 74 samples – 15 of which were oxacilin resistant. 8 of these patients were admitted from the ER. The other were transferred from wards: 3 from medical wards and 4 from surgical wards, with an average lenght of stay (LOS) before ICU admission of 5 days. 9 samples were positive for A. baumanii, 1 admitted from ER, 3 from surgical and 5 from medical wards. In 7 samples were found ESBL positive pathogens, 5 coming from surgical wards, 1 from ER and 1 from medical ward. From the blood cultures, 11.6% were positive. The most common pathogen was S. epidermidis in 46 samples. Regarding MDRs, we found 9 MRSA, 5 ESBL positive germens and 4 A. baumanii. The MRSA positive samples were obtained from patients coming mostly from surgical wards (5), followed by medical wards (2) and ER (2), with an LOS before ICU admission of 9.4 days. The ESBL positive pathogens were found in 3 patients admitted from ER and 2 patients from surgical wards, with average LOS previous to admission of 4.6 days. A. Baumanii was identified in 2 patients coming from medical wards, 1 from surgical and 1 from ER. In urine samples the most frequent germen was E. coli, found in 7 samples; and K. pneumoniae, found in 5. From these, 3 were ESBL positive, 2 coming from surgical wards and 1 from ER. The average LOS before ICU admission was 5.0 days. 2 samples were positive for A. baumanii. MDR Pseudomonas aeruginosa were found in 2 samples.

**Conclusions**

MDRs were found at ICU admission in 54 samples (9.6% of all positive samples). 44.4% came from surgical wards and 33.3% from ER. The number of MDR found in patients admitted from ER probably results from repeated contact with healthcare facilities. Surgical patients are more prone to MDR infections.

## P401 Microbiological yield of bronchoalveolar lavage in the setting of veno-venous ECMO

### D Baily^1^, LA Curran^2^, E Ahmadnia^2^, BV Patel^2^

#### ^1^Royal Brompton Hospital, London, United Kingdom; ^2^Royal Brompton and Harefield NHS Trust, London, United Kingdom

**Introduction**

Whilst bronchoalveolar lavage (BAL) in the context of adult extracorporeal membrane oxygenation (ECMO) has been shown to be safe [1], there is a paucity of data regarding the microbiological yield and microbiota of BAL in this setting. In community acquired pneumonia (CAP), BAL may be of additive diagnostic value in around 50% of patients [2], with a similar yield in ventilator associated pneumonia (VAP) [3]. The aim of this study was to describe the positive microbiological BAL results and the yield of BAL in the context of adult veno-venous ECMO (VV-ECMO) at a large UK ECMO centre.

**Methods**

A retrospective analysis was conducted of all BAL microbiological results of patients supported with VV-ECMO at a UK specialist centre for severe acute respiratory failure. Data were collected for the period April 2014 to July 2016, restricted to the initial diagnostic BAL done on admission to this centre.

**Results**

Of the 85 patients treated with VV-ECMO, BAL was performed on admission in 71/85 cases (84%). The vast majority (83/85) were on antibiotics for suspected bacterial pneumonia. A total of 58 positive microbiological results were obtained from 45/71 patients, giving an overall BAL yield of 63%. The bacterial yield from BAL was 18%. Fungi were isolated from 23 patients (32%), respiratory viruses from 19 patients (27%), and bacteria from 13 patients (18%). The most commonly isolated fungi were species of Candida (predominantly C. albicans), found in 18 patients. Aspergillus was isolated from 4 patients (6%), but in no case was it the only organism identified. Influenza A, found in 11 patients (15%), accounted for more than half of all the positive virology results. Staphylococcus aureus was the most common bacterium isolated (7%), closely followed by Pseudomonas aeruginosa (6%).

**Conclusions**

BAL on admission to a specialist centre provides an acceptable overall microbiological yield in the context of VV-ECMO (as compared with previous data for CAP and VAP). The bacterial yield is low. The finding of a positive BAL fungal result should be correlated with the clinical context.

**References:**

[1] Sharma NS et al. Respir Care 2016 May;61(5):646–51

[2] Van der Eerden et al. Eur J Clin Microbiol Infect Dis (2005) 24: 241

[3] Medford et al. J Crit Care 2009 Sept;24(3):473.e1–6

## P402 Catheterization, bacteriuria and urinary tract infection in coronary care unit

### D Adukauskiene^1^, J Cyziute^2^, A Adukauskaite^3^, D Pentiokiniene^1^

#### ^1^Hospital of Lithuanian University of Health Sciences, Kaunas, Lithuania; ^2^Lithuanian University of Health Sciences, Kaunas, Lithuania; ^3^Innsbruck Medical University Hospital, Innsbruck, Austria

**Introduction**

The aim was to estimate the rate of urinary tract infection in bacteriuria, duration of urinary tract infection treatment and length of stay also associated factors with poor outcome in Coronary Care Unit patients.

**Methods**

Retrospective analysis of 57 patients data with bacteriuria treated in Coronary Care Unit in Hospital Kaunas Clinics of Lithuanian University of Health Sciences during 5 years was carried out.

**Results**

Urinary tract infection was revealed in 52 patients (91.2%) of 57 with bacteriuria, p < 0.05. Mean length of stay in Coronary Care Unit in case of bacteriuria as colonisation was 3.6 ± 1.8 days, in bacteriuria as urinary tract infection was 10.67 ± 3.9 days, p < 0.05. Length of stay in Coronary Care Unit was 10.68 ± 5.3 days if urinary tract infection was caused by bacteria, 27.75 ± 12.3 days by fungi, p < 0.05. Duration of urinary tract infection in 24 patients with adequate empirical antibacterial treatment was 9.9 ± 4.7 days, but 15.7 ± 3.3 days in 28 patients with inadequate antibacterial treatment, p < 0.05. Length of stay in Coronary Care Unit was 3.3 ± 2.1 days in case of adequate empirical antibacterial treatment, 14.7 ± 5.3 days when inadequate, p < 0.05.

Twenty – one patient (40.4%) of 52 with urinary tract infection has died in Coronary Care Unit. Seventeen patients (39.5%) of 43 have died in group > = 65 years, 4 patients (44.4%) of 9 in group 50 – 64 years, but all 5 patients < 50 years have survived, p < 0.05. 5 patients (45.5%) of 11 with diabetes mellitus have died (95%; OR 1.3; CI 0.33 – 4.99; p < 0.05). Twenty – one patient (42.9%) has died of all 49 with urinary bladder catheterization, 8 patients (25%) of 32 with short – term urinary bladder catheterization, 13 patients (76.5%) of 17 with long – term urinary bladder catheterization, but none of 8 patients without of it, p < 0.05.

**Conclusions**

Rate of urinary tract infection in bacteriuria was 91.2%. Length of stay in Coronary Care Unit was longer in case of bacteriuria due to urinary tract infection and fungi as pathogens of it. The length of urinary tract infection treatment as well as stay in Coronary Care Unit was shorter in case of adequate antibacterial treatment. The mortality rate of urinary tract infection in Coronary Care Unit was 40% related to age > = 50, diabetes mellitus, urinary bladder catheterization, especially long – term.

## P403 Bacterial colonization in tracheoventilated patients

### F Righetti, E Colombaroli, G Castellano

#### Intensive Care Unit. St. Boniface Hospital, Verona, Italy

**Introduction**

Ventilator-associated pneumonia (VAP) is an infection related to high mortality, high costs for antibiotic therapy and long hospital stays and rehabilitation programs. Our retrospective and observational study aims to evaluate the prevalence of bacterial colonization of the airways in tracheostomized and ventilated patients, affected by motoneuron disease [1, 2].

**Methods**

22 tracheostomized patients, ventilated for 24 hours a day, at home, affected by motoneuron disease, followed in the period 2015-2016, tracheostomic cannula change every 90 days, tracheobronchial sample and chest X-ray performed every year in absence of VAP. In the considered period, no patients was hospitalized nor has received targeted antibiotic therapy. It was compared the prevalence of bacterial colonization in 2015 compared to 2016.

**Results**

In 2015 the prevalence of Pseudomonas Aeruginosa was 64%, of MRSA (Stafilococcus Aureus methicillin resistant) 35%, of Klebsiella Pneumoniae 5% and of Enterobacteriacee (Providencia, Serratia, Proteus, Enterococchi) 52%. 52% of patients have polymicrobial infections. In 2016 the prevalence of Pseudomonas Aeruginosa was 63%, of MRSA 45%, of Klebsiella Pneumoniae 9% and of Enterobacteriacee 54%. 54% of patients have polymicrobial infections. In any of the 2 periods are found colonizations by totiresistant bacteria.

**Conclusions**

Comparing the 2 periods, we found that there aren’t substantial percentage differences of prevalence of bacterial colonization by Gram negative bacteria; on the contrary there is an increase of bacterial colonization by Gram positive bacteria - MRSA. Our study shows that tracheostomized patients, ventilated for 24 hours a day at home do not develop totiresistant colonization.

**References**

1. Behnia M. et al.: Nosocomial and ventilator-associated pneumonia in a community hospital intensive care unit: a retrospective review and analysis. BMC Res Notes. 2014 Apr 11;7:232

2. Morar P. et al.: Oropharyngeal carriage and lower airway colonisation/infection in 45 tracheotomised children. Thorax. 2002 Dec;57(12):1015–20

## P404 Lower bacterial growth after induction of endotoxin-tolerance in a porcine intensive care sepsis model

### F Wilske^1^, P Skorup^1^, M Lipcsey^2^, K Hanslin^2^, A Larsson^1^, J Sjölin^1^

#### ^1^Uppsala University, Uppsala, Sweden; ^2^Uppsala University, Department of Surgical Sciences, Uppsala, Sweden

**Introduction**

Endotoxin tolerance leads to an attenuated inflammatory response at a second hit [1]. From this it was hypothesized that bacterial killing might be affected similarly. If so, studies whether this might be of importance for the choice of antibiotics are warranted. In a previous study it has been shown that bacterial elimination and killing in bacteraemic animals occur in the spleen and liver [2]. Therefore, the objective of this study was to compare the killing of bacteria in the spleen and liver in animals made endotoxin tolerant by a 24h-endotoxin infusion with that in animals not exposed to endotoxin.

**Methods**

The endotoxin exposed group (Exp) (n = 18) were, prior to the bacterial exposure, given 0.063 μg/kg/h endotoxin during 24 h. Thereafter, an *Escherichia coli* intravenous infusion of 8.3 log_10_ Colony Forming Unit (CFU)/h was administered for 3 h. The endotoxin unexposed group (Unexp) (n = 18) was given an identical bacterial challenge but without the preceding exposure of endotoxin. The animals were observed for signs of sepsis and inflammatory response and treated in accordance with an intensive care protocol [2]. Blood cultures were obtained hourly. After 6 h the animals were killed by a potassium chloride injection. Biopsies for quantitative culture were taken from the liver and spleen immediately post mortem.

**Results**

All animals developed signs of sepsis. Peak TNF-α levels 1 h after start of the bacterial infusion were 2.27 ± 0.13 and 4.17 ± 0.13 log10 pg/ml (mean ± SE) in the Exp and Unexp groups, respectively. Clearance of bacteria from the blood was rapid and within 1 h. The Exp group demonstrated a significantly increased killing and attenuated growth of bacteria in the spleen and a similar trend in the liver. Mean bacterial growths in the spleen of the Exp and Unexp groups were 3.16 ± 0.13 and 3.73 ± 0.17 log_10_ CFU/g (mean ± SE), respectively (p = 0.01). Mean bacterial growths in the liver of the Exp and Unexp groups were 2.01 ± 0.18 and 2.40 ± 0.23 log_10_ CFU/g (mean ± SE), respectively (p = 0.20).

**Conclusions**

Contrary to the hypothesis the reduced cytokine response characteristic for endotoxin tolerant animals was not associated with a reduced bacterial killing in the spleen and the liver. This indicates that these processes do not run in parallel in vivo. The effect on bacterial killing after more prolonged activation of inflammatory and anti-inflammatory responses needs further investigation.

**References**

[1] Castegren et al. Shock 37:501–510, 2012

[2] Skorup et al. PLoS ONE, e90441, 2014

## P405 Clinical predictors and outcome of Klebsiella pneumoniae bacteremia in a regional hospital in Hong Kong

### M Man^1^, HP Shum^1^, YH Chan^1^, KC Chan^2^, WW Yan^1^, RA Lee^3^, SK Lau^4^

#### ^1^Department of ICU, PYNEH, Hong Kong, Chin; ^2^Department of Anaesthesia and Intensive Care, Tuen Mun Hospital, Hong Kong, China; ^3^Hong Kong East Cluster Department of Clinical Pathology, Hong Kong, China; ^4^Department of Microbiology, The University of Hong Kong, Hong Kong, China

**Introduction**

Klebsiella pneumoniae (KP) infection is associated with high morbidity and mortality in different clinical settings. Multi-drug resistance associated with extended spectrum beta-lactamase (ESBL) among KP is endemic worldwide. Our study aims to evaluate the clinical characteristics and outcomes of patients with KP bacteraemia in the critical care and general ward settings.

**Methods**

Adult patients admitted to a regional hospital in Hong Kong from 1 January 2009 to 30 June 2016 (7.5 years) with KP bacteremia were included. Demographics, disease severity, clinical features, microbiological characteristics and outcome were analyzed.

**Results**

Among the 853 patients, 178 (20.9%) required critical care and 176 (20.6%) died within 30 days of hospital admission. 30-day survivors were younger (p < 0.001), had lower disease severity (defined by SOFA score) (p < 0.001), presented with hepatobiliary sepsis (p < 0.001) or urosepsis (p < 0.001), less septic shock (p = 0.013) or requirement of invasive organ support (p < 0.001) and received appropriate empirical antibiotics (p < 0.001). Cox regression analysis showed that respiratory tract (HR = 2.99; 95% CI = 2.061-4.337;p = <0.001), gastrointestinal tract (excluding hepatobiliary system) infection (HR = 2.763; 95% CI = 1.761-4.337; p = <0.001), use of mechanical ventilation (HR = 2.202; 95% CI = 1.506-3.221; p = <0.001), medical case (HR = 1.830; 95% CI = 1.253-2.672; p = 0.002), inappropriate empirical antibiotics (HR = 1.716; 95% CI = 1.267-2.324; p = <0.001), female (HR = 1.699, CI = 1.251-2.307, p < 0.001), age >65 (HR = 1.692; 95% CI = 1.160-2.467; p = 0.006) and presence of solid tumor (HR = 1.457; 95% CI = 1.056-2.009; p = 0.022) were independent risk factors for 30-day mortality. Unexpectedly, presence of diabetes mellitus was associated with a better 30-day survival (p = 0.002). ESBL-producing strains were identified in 102 patients (12.0%). Non-hepatobiliary sepsis, use of systemic steroid within 30 days before positive blood culture, presence of solid tumor and use of central venous catheter independently predicted the occurrence of ESBL KP bacteremia. However, presence of an ESBL strain was not associated with higher 30-day mortality.

**Conclusions**

KP bacteremia is associated with high 30-day mortality. Site of infection, patient’s comorbidities and appropriate use of empirical antibiotics are important predictors of patients’ outcome. Early empirical antibiotics in high-risk groups is warranted.

## P406 Mortality rate of ventilator-associated pneumonia patients receiving tigecycline in intensive care unit, university hospital, Thailand

### P Dilokpattanamongkol^1^, P Thirapakpoomanunt^1^, R Anakkamaetee^1^, P Montakantikul^1^, V Tangsujaritvijit^2^

#### ^1^Faculty of Pharmacy, Mahidol University, Bangkok, Thailand; ^2^Faculty of Medicine, Ramathibodi Hospital, Mahidol University, Bangkok, Thailand

**Introduction**

Several studies have shown that the use of tigecycline, especially in ventilator associated pneumonia (VAP) patients, was significantly associated with increased risk of all-cause mortality. However, tigecycline is one of the options for multidrug resistant Acinetobacter baumannii which is a growing problem in Thailand. Moreover, there have not been any studies showing such correlation at a University Hospital, Thailand. The purpose of this study was to investigate the mortality rate and the length of hospital stay of Acinetobacter baumannii VAP patients receiving tigecycline compared with other antibacterial agents.

**Methods**

A retrospective study chart review was performed in patients admitted to Intensive Care Units, University Hospital during 2007 and 2015 and received the treatment for Acinetobacter baumannii VAP. Primary outcome, mortality rate, and secondary outcome, ventilator days, in both groups were collected.

**Results**

There were 126 patients; 80 patients in tigecycline group and 46 patients in non-tigecycline group. We selected only 41 Acinetobacter baumannii VAP patients in each group with matched baseline characteristics. The mortality rate of tigecycline group was significantly higher than non-tigecycline group (73.20% and 46.30% respectively, P = 0.013). The median length of hospital stay was also significantly higher in tigecycline group than non-tigecycline group (46.99 days and 30.24 days respectively, P = 0.004).

**Conclusions**

The use of tigecycline in Acinetobacter baumannii VAP patients increased the mortality rate and the length of hospital stay compared with the use of other antibacterial agents. However, a prospective cohort study with larger sample size is necessary.

**References**

1. Stein GE, Babinchak T. Review: Tigecycline: an update. Diagn Microbiol Infect Dis 2013; 75: 331–6.

Walker T. FDA warns of increased death risk with tigecycline use. 2013: 30.

2. Pongpech P, Amornnopparattanakul S, Panapakdee S, et al. Antibacterial activity of carbapenem-based combinations againts multidrug-resistant Acinetobacter baumannii. J Med Assoc Thai 2010; 93 (2):161–71.

## P407 Outbreak of colistin resistant bacteria in a tertiary care center-analysis of risk factors and outcome

### S Sinha, J Pati, S Sahu

#### Apollo hospitals, Bhubaneswar, India

**Introduction**

The incidence of carbapenem resistant enterobacteriacae (CRE) has been steadily rising [1]. The morbidity, mortality and financial implications of such patients are significant. We recently had few reports of colistin resistance in our centre. This prompted us to analyse the risk factors for the outbreak of pan drug-resistant (PDR) organism.

**Methods**

We did a retrospective analysis of the case records of the patients who had any culture report positive for pan drug resistant (PDR) organisms. Cultures were done in our centre using VITEK 2 compact for all specimens. We followed the report of VITEK-2 and EUCAST breakpoints were followed (S < = 2, R > =2) for Enterobacteriacae. Pseudomonas and Acinetobacter isolates were considered resistant to colistin if the MIC > = 8 and 4 respectively. Their APACHE II score on admission, all culture reports, antibiotics received, length of stay in ICU and hospital along with outcome were scrutinized.

**Results**

There were total 10 isolates of PDR organisms in 8 patients. Five patients had received prior colistin therapy due to growth of carbapenem resistant bacteria isolates while 3 cases had no prior colistin treatment. Two out of 5 such cases had colistin monotherapy. Among 10 such isolates, there was Klebsiella pneumonia in 7, Pseudomonas aeruginosa in 2 and Acinetobacter baumanii in one case. Among these 7 isolates were considered to be coloniser and three were infective pathogens. These PDR isolates were associated with CAUTI (5), tracheitis (2), bacteremia (1), meningitis (1) and soft tissue infection (1). Average APACHE II score was 24 indicating sicker patients with multiple co-morbidities and organ dysfunction. Mean age of the patients was 50.4years. Average length of hospital stay was 42.8 days. Four of these patients died while other 4 were discharged home. Six of the 8 patients belonged to neurosciences (stroke and traumatic brain injury) and had overall poor status with Glassgow coma scale (GCS) <8 with long hospital stay.

**Conclusions**

Critically ill patients with longer hospital stay are more likely to get affected by PDR organisms. Patients requiring long-term rehabilitation should be cared for in dedicated centers. Culture reports should be judiciously interpreted to differentiate between colonizer and infective pathogen before treatment. Widespread indiscriminate use of colistin to treat CRE and other gram negative organisms can lead to emergence of PDR organisms. Strict implementation of antibiotic stewardship programme are essential to limit use and prevent abuse of colistin.

**References**

1. Antoniadou A, Kontopidou F et al:Colistin-resistant isolates of Klebsiella pneumoniae emerging in intensive care unit patients: first report of a multiclonal cluster. J Antimicrob Chemother. 2007 Apr;59(4):786–90.

## P408 Where are we going? Analysis of MDR and ESBL E. coli monobacteremia in ICU of university hospital

### D Adukauskiene, D Valanciene, A Dambrauskiene

#### Hospital Kaunas Clinics of Lithuanian University of Health Sciences, Kaunas, Lithuania

**Introduction**

The aim of study was to analyze multidrug-resistant (MDR) and extended spectrum beta – lactamases (ESBL) producing E. coli strains of monobacteremia also associated factors with length of stay in Intensive Care Unit (ICU) > =7 days and mortality.

**Methods**

The retrospective data analysis of patients (pts) treated in surgical and medical ICU of Kaunas Clinics with E. coli positive blood culture during 2005-2015 was carried out.

**Results**

There were found 87 (70.7%) MDR strains among 123 cases of E. coli monobacteremia (P = 0.046). Rate of MDR strains during study period was: 6 (42.9%) cases in 2005, 12 (66.7%) in 2010, 12 (85.7%) in 2015 (P = 0.027, RR = 17.324).

There were found 57/87 (65.5%) cases of ESBL producing strains among E. coli MDR strains (P = 0.04). Rate of ESBL producers during study period was: 2 (16.7%) cases in 2005, 7 (58.3%) in 2010, 10 (76.0%) in 2015 (P = 0.03, RR = 14.856).

28/57 (49.1%) pts with both MDR and ESBL E. coli strain stayed in ICU > =7 days. All 28 (100%) pts had SOFA score > =13, were mechanically ventilated > =3 days and were in shock, respectively (P = 0.001), 25/28 (89.3%) were admitted from emergency department (P = 0.03).

79/87 (90.8%) pts with E. coli MDR strain have died. All 79 (100%) pts were mechanically ventilated (P = 0.04), 78 (98.7%) were in shock (P = 0.001, OR = 5.909, CI95% = 2.176–16.049), 68 (81.0%) had diabetes mellitus (P = 0.03), 66 (78.6%) had renal dysfunction (P = 0.046), 59 (70.2%) had SOFA score > =13 (P = 0.032, RR = 12.00) and 54 (65.5%) pts had ESBL strain (P = 0.04).

54/57 (94.7%) pts with ESBL producing E. coli strain have died. All these 54 (100%) pts were mechanically ventilated (P = 0.001), were in shock (P = 0.001), 50 (92.6%) had SOFA score > =13 (P = 0.01), 45 (83.3%) had diabetes mellitus (P = 0.02) and 35 (64.8%) pts had renal dysfunction (P = 0.04).

**Conclusions**

MDR E. coli was found in 2/3 of all E. coli monobacteremia strains, 2/3 of them were ESBL producers. During 11 year rates of both MDR and ESBL strains were constantly increasing: MDR doubled, ESBL have increased in 4.5 times. Associated factors for stay in ICU > =7 days were admission from emergency department, ESBL producing strain, mechanical ventilation > =3 days, shock, SOFA score > =13. Very high mortality rate of E. coli monobacteremia due to MDR or ESBL strain was associated with diabetes mellitus, ESBL producing strain, mechanical ventilation, shock, renal dysfunction, SOFA score > =13.

## P409 E. coli bacteremia in intensive care unit: associated factors with length of stay and mortality

### D Adukauskiene, D Valanciene, A Dambrauskiene

#### Hospital Kaunas Clinics of Lithuanian University of Health Sciences, Kaunas, Lithuania

**Introduction**

The aim of study was to analyze antimicrobial resistance of E. coli in monobacteremia and associated factors with length of stay in Intensive Care Unit (ICU) > =7 days and mortality.

**Methods**

The retrospective data analysis of patients (pts) treated in surgical and medical ICU of Kaunas Clinics with E. coli positive blood culture during 2005-2015 was carried out.

**Results**

There were found 123 cases of E. coli monobacteremia, and this group was homogenous in point of gender, age, comorbidities, source of bacteremia (P > 0.05).

104 (84.6%) strains of E. coli were resistant to ampicillin, 94 (76.4%) - ampicillin/sulbactam, 90 (73.2%) - gentamicin, 88 (71.5%) – cefotaxim, 57 (46.3%) – ciprofloxacin, 56 (45.5%) - piperacillin, 34 (27.6%) - piperacillin/tazobactam, 29 (23.6%) - amikacin. Resistance to carbapenems was 0/123 (0%). 87/123 (70.7%) E. coli strains were found to be multidrug-resistant (MDR) (P = 0.046). 57/87 (65.5%) E. coli MDR strains were producers of extended spectrum beta-lactamases (ESBL) (P = 0.04). 32/123 (26.0%) pts stayed in ICU > =7 days. 30 (93.6%) pts among them were on mechanical ventilation > =3 days (P = 0.004, RR = 23.097), 29 (90.6%) had SOFA score > =13 (P = 0.036, RR = 18.762), 28 (87.5%) were in shock (P = 0.019, RR = 2.956), 25 (78.13%) were admitted from emergency department (P = 0.049, RR = 19.987), 23 (71.9%) had MDR strain (P = 0.049, RR = 15.085), 19 (59.4%) had ESBL strain of E. coli (P = 0.049, RR = 12.476). 88/123 (71.5%) pts with E. coli monobacteremia have died. Mortality rate’s dynamic during study period was: 12 (66.7%) cases in 2005, 13 (72.2%) in 2010 and 13 (92.9%) in 2015 (P = 0.012). 84 (95.5%) pts had MDR strain (P = 0.001, OR = 3.215, CI95% = 1.176–5.095), 82 (93.2%) were on mechanical ventilation (P = 0.04), 80 (90.9%) were with shock (P = 0.001, OR = 5.909, CI95% = 2.176–16.049), 68 (77.3%) had diabetes mellitus (P = 0.03), in 66 (75.0%) hemotransfusion was used (P = 0.03), 66 (75.0%) were with renal dysfunction (P = 0.0046), 59 (67.0%) had SOFA score > =13 (P = 0.032, RR = 12.00) and 57(64.8%) had primary type of bacteremia (P = 0.001).

**Conclusions**

High resistance of E. coli to aminopenicillins without and with beta-lactamases inhibitor, gentamicin, cefotaxim, ciprofloxacin and no resistance to carbapenems was estimated. Stay in ICU > =7 days was associated with admission from emergency department, MDR and ESBL strain of E. coli, mechanical ventilation > =3 days, shock, SOFA score > =13. Mortality rate of E. coli bacteremia 71% is significantly increasing and associated with diabetes mellitus, primary type of bacteremia, MDR strain, mechanical ventilation, shock, renal dysfunction, hemotransfusion and SOFA score > =13.

## P410 Incidence of ESKAPE pathogens and their antibiotic resistance in patients with invasive devices in the ICU of a public hospital

### K Hernandez, T Lopez, D Saca, M Bello

#### Universidad Dr. Jose Matias Delgado, Santa tecla, El Salvador

**Introduction**

The objective of this study is to determine the incidence of ESKAPE pathogens, their antimicrobial resistance and association with invasive devices in the ICU of a public hospital during its inaugural year. Worldwide, most ICU-infections are caused by ESKAPE pathogens, whose high rates of antibiotic resistance are an active threat to public health.

**Methods**

A retrospective, cross-sectional analytical study was conducted, including all patients admitted to this ICU during 2014. Statistical analysis was performed using SPSS 20.0, Graph Pad Prism 6, and Open Epi 3.01.

**Results**

150 cases were evaluated. The average age in the non-infectious, ESKAPE and non-ESKAPE cases was homogenous (Kruskal-Wallis Test, p = 0.053). 54% of cases were infectious. 13.3% of cases were ESKAPE cases. 46.4% (n = 26) of cultures isolated ESKAPE pathogens. Of these, Acinetobacter baumannii was the most common (50%). The most frequently prescribed antibiotics were ceftriaxone, imipenem and vancomycin, reporting resistance in 83%, 56.7% and 11.1% of the cultures respectively. 86.5% (n = 32) of the cases with pneumonia were associated with the use of endotracheal tube (ETT) (Xi^2^ Test, p = 0.0005). The cases with mechanical ventilation presented pneumonia 5.2 times more frequently than cases without it (OR 5.2, IC 95%, 1.9-14.4). 53% (n = 17) of the cases with pneumonia and ETT were caused by ESKAPE pathogens (p = 0.0711). 11.5% (n = 16) of the cases with UTI had a urinary catheter, and 2.8% were caused by an ESKAPE pathogen. The average ICU stay was 7.8 days, 4.9 days for the non-infectious cases, 7.3 days for the non-ESKAPE cases, and 19.5 days for the ESKAPE-cases (Kruskal-Wallis Test p < 0.0001). A moderate positive correlation between the days of mechanical ventilation and days of stay in ICU was established (Pearson r 0.59, p < 0.0001). The mortality rate was 45% in ESKAPE cases and 49% in non-ESKAPE cases. The infectious cases were lethal 3 times more frequently than the non-infectious cases (OR 3.052, IC 95%, 1.512-6.33).

**Conclusions**

Almost half of the cultures isolated ESKAPE pathogens. Ceftriaxone and Imipenem were most frequently prescribed antibiotics. Antibiograms reported high rates of resistance to these two antibiotics. All of the ESKAPE cases had at least two invasive devices. Pneumonia was more frequently diagnosed in patients with ETT. In average, the ESKAPE-cases stayed hospitalized 2.6 times longer than the non-ESKAPE-cases, and 4 times longer than the non-infectious-cases.

## P411 Determinants of acinetobacter sepsis in critically ill patients: a comparative study

### W Mahmood, K Hamed, N Al Badi, S AlThawadi, S Al Hosaini, N Salahuddin

#### King Faisal specialist hospital and research centre, Riyadh, Saudi Arabia

**Introduction**

The incidence of Acinetobacter infections has steadily increased, and now lately, has become a major threat with the emergence of multidrug-resistant strains. Acinetobacter are notorious for their ability to spread amongst hospitalized patients. This study attempts to identify ICU care variables predictive of Acinetobacter sepsis.

**Methods**

In this case-control study, we extracted data from a prospectively collected ICU database on all patients admitted with a diagnosis of sepsis from 2010 to 2015. Identification data on all Acinetobacter isolates was obtained from the Section of Microbiology database. Patients with Acinetobacter sepsis were compared with control patients. The institutional Research Ethics Committee approved the protocol.

**Results**

431 patients were studied, 43 (10%) developed Acinetobacter sepsis. Mean APACHE II score was 26 ± 7.7; median procalcitonin level was 3.9 (IQR 1.1, 18.4). Mean age was 52.5 ± 21.4 years with median ICU length of stay 6 (IQR 4.43) days. ICU mortality was 23% (99 patients) with mortality rate of patients with Acinetobacter sepsis at 60.5% (26 patients of 43). Patients who developed Acinetobacter sepsis had a mean SOFA score 14.1 ± 3.7 with 46.5% in septic shock, 9% organ donors, and 7% post-solid organ transplant. Most common site of isolation was the respiratory tract, 34.6%, followed by bloodstream/line sepsis, 30.8%, 32.5% had a single site infected. Median duration on mechanical ventilation was 15.3 (IQR 7, 15.3) days. On univariate regression analysis Acinetobacter sepsis was predicted by vasopressor dependence, OR 4.1 (95% CI 1.6,9.9, p =0.002), blood-stream infection, OR 6.3 (95% CI 3.2,12.4, p < 0.001), single site of initial sepsis OR 0.4 (95% CI 0.2, 0.9, p = 0.02), APACHE II score, OR 1.05 (95% CI 1.01, 1.1, p = 0.01), malignancy OR 6 (95% CI 2.2,15.7,p < 0.001) and appropriate empiric antibiotics OR 0.04 (95% CI 0.01,0.15, p < 0.001).On multivariate regression, appropriate empiric antibiotics OR 0.04 (95% CI 0.01,0.13, p < 0.001), vasopressor dependence, OR 3.1 (95% CI 1.07,9.2, p =0.03), blood-stream infection, OR 7.5(95% CI 3.2,17.4, p < 0.001), single site of initial sepsis OR 0.1 (95% CI 0.07, 0.4, p < 0.001) remained significant predictors of Acinetobacter sepsis.

**Conclusions**

Acinetobacter sepsis remains a frequent and hazardous ICU acquisition with a higher risk imposed by continued vasoplegia and septicemia and protective effects from appropriate initial antibiotic coverage and limited sites involved.

## P412 Acute respiratory distress syndrome in patients with pneumococcal community-acquired pneumonia

### CC Cilloniz^1^, AC Ceccato^1^, GL Li Bassi^1^, MF Ferrer^1^, AG Gabarrus^1^, OR Ranzani^1^, AS San Jose^1^, CG Garcia Vidal^1^, JP Puig de la Bella Casa^1^, FB Blasi^2^, AT Torres^1^

#### ^1^Hospital Clinic, Barcelona, Spain; ^2^Università degli Studi di Milano, IRCCS Fondazione Ca Granda Ospedale Maggiore Policlinico, Milan, Italy

**Introduction**

Community-acquired pneumonia (CAP) by Streptococcus pneumoniae could result into acute respiratory distress syndrome (ARDS). In patients with severe pneumococcal CAP, we characterized prevalence, risk factors and clinical outcomes of ARDS.

**Methods**

We prospectively enrolled adult patients admitted into a pulmonary intensive care unit (ICU). Patients were clustered based on ARDS occurrence, according to the Berlin definition. Characteristics, risk factors and outcomes were compared between groups.

**Results**

Among 6439 patients with CAP, 1307 (20%) had pneumococcal CAP; of those, 282 (23%) patients were admitted to the ICU and 115 required ventilatory support (70%, invasive mechanical ventilation and 30% non-invasive mechanical ventilation). Thirty-six out of 115 (31%) ventilated patients met the Berlin ARDS criteria. ARDS was mild, moderate and severe in 36, 44 and 20% of the cases, respectively. Multivariate analysis indicated that previous corticosteroid therapy decreased risk of ARDS (odds ratio (OR), 0.33; 95% confidence interval (95% CI), 0.11-0.94). Patients with ARDS presented less ventilation-free days (p = 0.047), higher ICU mortality (39% vs. 18%; p = 0.014) and higher in-hospital mortality (22% vs. 42%; p = 0.025). ARDS was an independent risk factor for ICU mortality (OR, 2.60; 95%CI, 1.04-6.53).

**Conclusions**

ARDS develops in a minor proportion of patients with pneumococcal CAP. Yet, ARDS highly increases mortality risks. Previous treatment with inhaled corticosteroids is the only ARDS protective factor.

## P413 Ventilator-associated pneumonia: MDR Gram-negative bacteria and predictors of mortality

### D Adukauskiene^1^, A Ciginskiene^1^, A Dambrauskiene^2^, R Simoliuniene^3^

#### ^1^Hospital of Lithuanian University of Health Sciences, Kaunas, Lithuania; ^2^Hospital of Lithuanian University of Health Sciences, Kaunas, Lithuania; ^3^Lithuanian University of Health Sciences, Kaunas, Lithuania

**Introduction**

The aim of study was to evaluate antimicrobial resistance of Gram-negative bacteria (GNB) as pathogens of ventilator-associated pneumonia (VAP) and to determinate predictors of in-hospital mortality.

**Methods**

Retrospective data analysis of patients treated in ICU with multidrug-resistant strains of GNB as pathogens of VAP during 2015 was carried out.

**Results**

In 77 VAP pts 98 multidrug-resistant strains of GNB were revealed: 37 (37.8%) of A. baumannii, 16 (16.3%) of Klebsiella spp., 11(11.2%) of Enterobacter spp., 10 (10.2%) of P. aeruginosa and others 24 (24.5%) as S. marcescens, Morganella spp., Citrobacter spp., E. coli, Proteus spp. In 34 (34.7%) of GNB strains multidrug-resistance (MDR), in 63 (64.3%) extensive drug-resistance (XDR) and in 1 (1%) pandrug-resistance (PDR) was ascertained. MDR was found in 7 (63.6%) of Enterobacter spp. and 7 (70%) of P. aeruginosa (p < 0.001), XDR - in 36 (97.3%) of A. baumannii strains (p < 0.001). One (10%) of P. aeruginosa strains was found to be PDR. 37 (48%) pts with multidrug-resistant pathogens of VAP have died. Statistical significant differences of survivors vs nonsurvivors were found in means of hospitalisation days prior of VAP 53.8 (SD = 34) vs 29,1 (SD = 17), p = 0.001, neutrophils percent 81 (SD = 8.9) vs 85 (SD = 6.3), p = 0.045, and between proportions of internal disease 7 (33%) vs 14 (67%), p = 0.042, co-infection 13 (30%) vs 30 (70%), p = 0.001, shock 16 (36%) vs 28 (64%), p = 0.03, multiple organ dysfunction syndrome 17 (40%) vs 25 (60%), p = 0.039, inappropriate initial antimicrobial treatment 18 (40%) vs 27 (60%), p = 0.02. OR for in-hospital mortality was: 8.9 (95% OR CI 2.24; 33.72) for co-infection, 4.67 (95% OR CI 1.7; 12.6) for shock and 3.3 (95% OR CI 1.27; 8.59) for inappropriate initial antimicrobial treatment.

**Conclusions**

A. baumannii and Klebsiella spp. were found to be predominating multidrug-resistant pathogens of VAP. 1/3 of all multidrug-resistant GNB of VAP were MDR and 3/5 were XDR. 2/3 of Enterobacter spp. and P. aeruginosa strains were found to be MDR. A. baumannii strain were found to be exclusively of XDR type. In-hospital mortality of VAP was 48%. Longer hospitalisation prior of VAP, neutrophilosis, internal disease, co-infection, shock, multiple organ dysfunction syndrome, inappropriate initial antimicrobial treatment were found to be associated with in-hospital mortality. Co-infection, shock and inappropriate initial antimicrobial treatment were found to be significant predictors for in-hospital mortality in ICU patients with multidrug resistant GNB in VAP.

## P414 Predictors of mortality in KPC-Kp bloodstream infection in intensive care units in Italy

### G Giuliano^1^, D Triunfio^1^, E Sozio^1^, E Taddei^1^, E Brogi^1^, F Sbrana^2^, A Ripoli^2^, G Bertolino^1^, C Tascini^3^, F Forfori^1^

#### ^1^AOUP, Pisa, Italy; ^2^Fondazione Toscana Gabriele Monasterio, Pisa, Italy; ^3^First Division of Infectious Diseases, Cotugno Hospital, Napoli, Italy

**Introduction**

Over the last decade, the prevalence of strains of Klebsiella Pneumoniae Carbapenemases (KPC) producing K. pneumoniae (Kp) has dramatically increased worldwide and has become a significant problem in terms of public health, especially in some countries.

**Methods**

In this retrospective observational study conducted in Intensive Care Units (ICU) of the the Italian teaching Hospital of Pisa, we recruited critically ill patients with a diagnosis of bloodstream infections (BSI) caused by KPC-Kp. 30-days mortality from the first positive blood culture, septic shock diagnosis, steroid therapy, SOFA, SAPS II, Charlson Score, antibiotics therapy, previous hospitalization were compared between Survivor and Non-Survivor groups.

**Results**

We enrolled 42 patients admitted in ICU between January 2012 and December 2015. The overall 30 days mortality rate was 52,4%. A significantly higher rate was observed among patients with a diagnosis of septic shock at BSI onset, steroid therapy, higher SOFA and SAPS II score. Gentamicin used in combination therapy was associated with lower mortality, regardless of meropenem use. Furthermore, in colonized patients the digestive decontamination with oral Gentamicin decreased BSI mortality. Previous use of meropenem was associated with increased mortality. Additionally, the intravenous admistration of Fosfomicin resulted in a lower mortality rate. Charlson score and optimal empirical therapy seemed not to have a significant influence on survival.

**Conclusions**

KPC-Kp BSI was associated with high mortality. We found that a regime therapy including gentamicin is associeted with lower mortality. Futher prospective studies should be done to confirm our result.

**Table 3 (abstract P414).** See text for description

	Survivor (n = 19)	Non survivor (n = 23)	
3 Months previous hospitalization	2/19 (10.5%)	9/23 (39,1%)	P = 0.054
Steroids use	3/19 (15.8%)	14/23 (60.9%)	P = 0.024
Septic shock	11/19 (57.9%)	15/23 (65.2%)	P = 0.045
SOFA (day 1)	8.65 ± 3.37	11.9 ± 3.37	P = 0.015
SAPS II (day 1)	57.58 ± 17.70	67.59 ± 20.99	P = 0.034
Charlson Score	3.05 ± 1.87	4.00 ± 1.87	Ns
Optimal empirical therapy	17/19 (89.5%)	20/23 (87%)	Ns
Previous carbapenems therapy	2/19 (10.5%)	9/23 (39.1%)	P = 0.081
Oral gentamicin decontamination	2/19 (21.1%)	0/23 (0%)	P = 0.093
Gentamicin therapy	6/14 (42.9%)	1/23 (4.3%)	P = 0.023
Fosfomycin therapy	5/14 (35.7%)	3/23 (13%)	P = 0.066
Gentamicin associated with other antibiotics	6/19 (31.6%)	15/23 (4.3%)	P = 0.072

Legend : Clinical characteristics, therapy and outcomes of study population

## P415 Global burden of neonatal and childhood sepsis

### C Fleischmann^1^, D Goldfarb^2^, P Schlattmann^1^, L Schlapbach^3^, N Kissoon^4^

#### ^1^Jena University Hopital, Jena, Germany; ^2^University of British Columbia, Vancouver, Canada; ^3^University of Queensland, Brisbane, Australia; ^4^British Columbia Childrens Hospital, Vancouver, Canada

**Introduction**

Neonates and children under 5 years are at major risk for sepsis, but knowledge on the global epidemiology of childhood sepsis is scarce. The aim of this study was to estimate the global burden of and mortality from sepsis in children and neonates based on available evidence from observational epidemiological studies.

**Methods**

Systematic review and meta-analysis. We searched 13 international databases for published and grey literature between 1979-2016, supplemented by hand search and expert consultation. Studies reporting on the incidence of sepsis in children (<20 y) defined according to the International Consensus Conference on Pediatric Sepsis Definitions [1], ACCP/SCCM consensus criteria [2,3] or related sepsis-relevant ICD-9/ICD-10 codes on a population level per 100 000 person-years or live births were included.

**Results**

Of 1270 studies, 23 studies from 16 countries met the inclusion criteria. Sixteen were from high-income-countries, seven from middle-income-countries. Fifteen studies reported complete data and were included in the meta-analysis. We found an aggregate estimate of 48 [95% CI, 27-86] sepsis cases and 22 [95% CI, 14-33] severe sepsis cases per 100 000 child population in high-income-countries. Mortality ranged between 1-5% for sepsis and 9-20% for severe sepsis. The population-level estimate for neonatal sepsis was 2062 [95% CI, 1065-3957] per 100 000 live births with a mortality rate between 11-19% in middle- and high-income-countries. Extrapolating these figures on a global scale, we estimate 2.9 Mio. cases of neonatal sepsis with 319 000-551 000 neonates dying from or with sepsis.

**Conclusions**

Sepsis is a major contributor to neonatal and childhood mortality. Further comprehensive studies on sepsis epidemiology especially in low- and middle-income countries are needed, as well as effective measures to reduce the burden of sepsis in children globally.

**References**

(1) Goldstein et al. International pediatric sepsis consensus conference: definitions for sepsis and organ dysfunction in pediatrics. Pediatr Crit Care Med. 2005 Jan;6(1):2–8.

(2) Bone et al. Definitions for sepsis and organ failure and guidelines for the use of innovative therapies in sepsis. Chest 1992;101:1644–1655.

(3) Levy et al. 2001 SCCM/ESICM/ACCP/ATS/SIS international sepsis definitions conference. Crit Care Med 2003;31:1250–1256.

## P416 Epidemiology of sepsis in Turkish intensive care units: a multicenter point- prevalance study.

### N Baykara^1^, H Akalin^2^, M Kemal Arslantas^3^, And Sepsis Study Group^4^

#### ^1^University of Kocaeli, Kocaeli, Turkey; ^2^Uludag University, Bursa, Turkey; ^3^Marmara University, Istanbul, Turkey; ^4^Turkish Society of Intensive Care Medicine, Istanbul, Turkey

**Introduction**

The prevalance and mortality of sepsis in Turkey is largely unknown. Turkey is among the countries that have a high level of antibiotic resistance. Turkish Society of Intensive Care Medicine, Sepsis Study Group conducted a national, multicenter, point-prevalance study to determine the prevalence, causative microorganisms, and outcome of sepsis in Turkish ICUs.

**Methods**

A total of 132 ICUs from 94 hospitals participated the study. All patients (>18 yr old) present on the participating ICU or admitted for any length of time during a 24 hr period between 08:00 on Wednesday 27 January 2016 and 08:00 on Thursday 28 January 2016 were included the study. The International Sepsis Forum definitions for common sites of infection were used [1]. The presence of SIRS, severe sepsis and septic shock was assessed and documented based on consensus criteria of the ACCP/SCCM (SEPSIS-I) [2]. Data were also used to define septic shock according to the SEPSIS-III definitions [3]. Demographics, severity of illness, comorbidities, microbiological data, therapies used, length of stay and outcome (dead or alive through 30 days) were recorded.

**Results**

Of 1499 patients screened, 165 (11%) had sepsis, 260 (17.3%) had severe sepsis without shock, and 204 (13.6%) had septic shock. Mortality rates increased significantly with increasing severity of sepsis (31.3%, 55.8%, and 71.1%, for sepsis, severe sepsis and septic shock, respectively; p < 0.05). According to the SEPSIS-III definitions, 91 (10.5%) patients had septic shock and mortality rate for septic shock was 75.3%. The respiratory system was the most common site of infection, accounting for 62.8% of all infections, followed by the the bloodstream (14.9), and the urinary system (7%). Acinetobacter species were the most common isolated pathogen (34.5%), followed by Klebsiella species (16.7%) and Pseudomonas species (14.5%). While colistin was the last-line therapeutic drug used against multidrug-resistant Gram-negative pathogens, 5% of Klebsiella spp. isolates, 3.3% of Pseudomonas spp. isolates, and 2.4% of Acinetobacter spp. isolates were resistant to colistin.

**Conclusions**

This study found a high prevalance of sepsis and an unacceptable high mortality rate in Turkish ICUs.

**References**

1. Calandra T. Crit Care Med 2005;33:1538–1548.

2. Bone RC. Chest. 1992 Jun;101(6):1644–55.

3. Seymour CW.JAMA. 2016 Feb 23;315(8):762–74.

## P417 Assessment of clinical criteria for sepsis in low and middle income countries

### SG Gavrilovic^1^, MV Vukoja^1^, MH Hache^2^, RK Kashyap^3^, YD Dong^3^, OG Gajic^3^

#### ^1^Istitute for Pulmonary Diseases of Vojvodina, Sremska Kamenica, Serbia; ^2^CEDIMAT, Santo Domingo, Dominican Republic; ^3^Mayo Clinic, Rochester, New York, United States

**Introduction**

In 2016 The Third International Consensus Definitions Task Force defined sepsis as a life-threatening organ dysfunction due to a dysregulated host response to infection [1]. Evaluation of new clinical criteria for this sepsis definition was not done so far in Low and Middle Income Countries (LMC). The aim of this study was to evaluate the prognostic ability of new and old clinical criteria for sepsis in LMCs.

**Methods**

This is a secondary analysis of CERTAIN (Checklist for Early Recognition and Treatmentof Acute Illness and Injury) study data from 15 intensive care units (ICU) from LMC. We included adult patients with discharge diagnosis of infection. Data on admission SOFA were collected prospectively. The presence of SIRS was determinedfrom prospectively collected APACHE II data on admission. Association with mortality was explored using univariate logistic regression.

**Results**

Between October 2013 and July 2016, out of 1398 eligible patients, 131 patients (9.4%) were included in the study with both SIRS and SOFA and a discharge diagnosis of infection. There were 51 female (39%) and 80 male (61%) patients from 15 ICUs. Median SIRS was 2 (IQR 1-3) and median SOFA was 7 (IQR 4-9), 28 days mortality was 47.2%. The SOFA score was higher among non-survivors (7.84 +/- 3.8 vs. 6.42 +/-3.36, p = 0.03). SIRS score did not differ among survivors and non-survivors (1.7 +/- 1.2 vs. 2 +/- 1.3, p = 0.197).The SOFA score > =2 was predictivefor 28 days mortality (AUC 0.62, p = 0.03), while SIRS > = 2was not (AUC 0.56, p = 0.2).

**Conclusions**

In a sample of LMCs ICU patients with infection SOFA, but not SIRS sepsis criteria are predictive for 28 days mortality.

**References**

1. Singer M, Deutschman CS, Seymour CW, Shankar-Hari M, Annane D, Bauer M, et al. The third international consensus definitions for sepsis and septic shock (Sepsis-3). JAMA (2016) 315:801–10.10.1001/jama.2016.0287

## P418 Measuring and comparing sepsis outcomes between countries to explore the impact of heterogeneity: a case study on adult medical admissions in England and Brazil

### O Ranzani^1^, M Shankar-Hari ^2^, D Harrison^1^, L Rabello^3^, K Rowan^1^, J Salluh^3^, M Soares^3^

#### ^1^Intensive Care National Audit & Research Centre, London, United Kingdom; ^2^Guy’s and St Thomas’ NHS Foundation Trust, London, United Kingdom; ^3^DOr Institute for Research and Education - IDOR, Rio De Janeiro, Brazil

**Introduction**

We tested the hypothesis that differences in generic and sepsis-specific patient characteristics explain the observed differences in sepsis outcomes between countries [1].

**Methods**

We studied first ICU episode for adult medical patients with sepsis admitted during 2013 using national data sources from England (ICNARC Case Mix Programme) and Brazil (ORCHESTRA study). After harmonizing relevant variables, the datasets were merged.

Sepsis was defined as infection and > =1 organ dysfunction (OD) (> = 2 points) using a modified SOFA score to align with Sepsis-3 [2]. The primary outcome was acute hospital mortality. We used multilevel logistic regression models to evaluate the impact of country (Brazil vs England) on hospital mortality, after adjustment for generic (age, sex, comorbidities, admission source, time to ICU admission) and sepsis-specific (infection site, OD type and first order interactions) characteristics. We report risk-adjusted mortality stratified by admission source, time in hospital prior to ICU admission, infection site, and decile of predicted risk of death (from our regression model).

**Results**

Of medical ICU admissions, 30.7% (17,921/58,316) in England and 13.2% (4,505/34,150) in Brazil met the sepsis definition. The Brazil sepsis cohort was older and had greater prevalence of serious comorbidities and dependency when compared with England. Respiratory was the commonest infection site (England 61.8%, Brazil 50.7%). The commonest OD was respiratory in England (85.8%) and cardiovascular in Brazil (41.2%). Crude mortality was similar (England 39.3%, Brazil 41.4%). After adjusting for generic characteristics, Brazil had lower odds of mortality (OR 0.88 [0.75-1.02], p = 0.089). However, after adding sepsis-specific characteristics, Brazil had higher risk-adjusted mortality (OR = 1.22 [1.05-1.43]; p = 0.01; AUROC = 0.78; Brier Score = 0.18). We observed statistically significant interactions in the full model when stratifying by time in hospital prior to ICU admission, infection site and deciles of predicted risk of death.

**Conclusions**

We show for the first time that generic and sepsis-specific patient characteristics explain observed differences in sepsis outcomes between countries.

**References:**

1. Shankar-Hari et al. Crit Care Med 2016; 44:2223–2230

2. Singer M et al. JAMA; 2016; 315: 801–10

## P419 Long-term temperature control with the esophageal heat transfer device in non-cardiac-arrest patients

### AM Markota, JF Fluher, DK Kogler, ZB Borovšak, AS Sinkovic

#### University Medical Centre Maribor, Maribor, Slovenia

**Introduction**

Esophageal heat transfer device (Advanced Cooling Therapy, Chicago, Ill, USA) is a new temperature management device, which has mostly been used for temperature management in survivors of cardiac arrest [1]. Our aim was to evaluate the effectiveness of the device in long-term temperature management in non-cardiac-arrest patients.

**Methods**

A retrospective study from January to November 2016 with inclusion criteria: the device used for >120h in non-cardiac arrest patients.

**Results**

**Fig. 14 (abstract P419).**
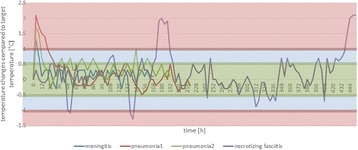
Temperature changes compared to target temperature over time

We included 3 males (age 67, 71 and 74 years) and 1 female (36 years). The duration of treatment with the device was 220, 192, 452 and 168h, respectively. Indications for temperature management were hyperthermia associated with sepsis (1 patient with meningitis, 2 with pneumonia and 1 with necrotizing fasciitis). Target temperature was determined by the attending physician (36-37°C in a patient with meningitis and one patient with pneumonia, 36.5-37.5°C for one patient with pneumonia, and 37-38°C in patient with fasciitis). Temperature was in target range for 81% of time, ±0.5°C outside target range for 11.6% of time, and > =0.6°C outside of target trange for 7.4%. The greatest temperature fluctuations (>2°C outside target temperature) were observed in the patient with fasciitis after return from operating theatre (the device was disconected from the chiller during surgery) (Fig. 14).

**Conclusions**

Esophageal heat transfer device can be used for effective long-term temperature control in non-cardiac arrest patients.

**References**

1. Markota A et al. Am J Emerg Med 34: 741–5, 2016

## P420 Effects of adsorption of cytokines early in septic shock (the ‘ACESS-trial’) – results of the pilot study

### I László^1^, N Öveges^1^, M Forgács^1^, T Kiss^1^, P Hankovszky^1^, P Palágyi^1^, A Bebes^1^, B Gubán^1^, I Földesi^1^, Á Araczki^1^, M Telkes^1^, Z Ondrik^1^, Z Helyes^2^, Á Kemény^2^, Z Molnár^1^

#### ^1^University of Szeged, Szeged, Hungary; ^2^University of Pécs, Pécs, Hungary

**Introduction**

Dysregulated systemic inflammatory response in septic shock often results an overwhelming “cytokine storm”, evolving into fulminant sepsis, with multiple organ dysfunction and early death. Attenuating the cytokine storm by adsorbing cytokines via hemoperfusion (CytoSorb) has pathophysiological rationale. Our aim was to investigate the effects of CytoSorb therapy on organ dysfunction and inflammatory response when started early (<48 h) in septic shock.

**Methods**

Patients fulfilling septic shock criteria were randomized into CytoSorb and Control groups. CytoSorb therapy lasted for 24 hours. Blood samples were taken to determine IL-1, IL-1ra, IL-6, IL-8, IL-10, TNF-α, PCT, CRP levels. At this stage of the study only PCT and CRP levels were analyzed. Organ dysfunction was evaluated by the Sequential Organ Failure Assessment (SOFA) score. Data were recorded on enrollment (T_0_) then at T_12_, T_24_, and T_48_ hours.

**Results**

Within the first 2 years of the study 14 patients were randomized into CytoSorb (n = 7), and Control-groups (n = 7). Both SOFA scores and mortality risk showed significant decrease between T_0_ and T_48_ the CytoSorb group: T_0_ = 12.7 ± 3.5, T_48_ = 9.4 ± 6.2, p = 0.028; T_0_ = 55.4 ± 26.2, T_48_ = 34.0 ± 30.8%, p = 0.013. Dose of the required norepinephrine also showed a significant reduction by T_48_ as compared to T_0_ and T_24_ (T_0_ = 51.4 ± 53.0, T_24_ = 40.7 ± 45.4, T_48_ = 11.9 ± 18.1μg/min, p = 0.035). Regarding the inflammatory response PCT and CRP levels were grossly elevated at T_0_, but there was no difference between the CytoSorb-, and Control-groups within the first 48 hours.

**Conclusions**

According to our interim results CytoSorb therapy resulted in better SOFA scores, and also proved to be safe without significantly affecting PCT and CRP changes within the first 48 hours of septic shock. Based on the results of this current pilot study we are planning to design a prospective randomized multicenter trial.

**Acknowledgements:**

NKFIH K116689

ClinicalTrials.gov ID: NCT02288975

## P421 Comparative anticoagulant effects of recombinant thrombomodulin, antithrombin, and unfractionated heparin – clinical implications

### J Fareed^1^, Z Siddiqui^1^, P Aggarwal^1^, O Iqbal^1^, D Hoppensteadt^1^, M Lewis^1^, R Wasmund^1^, S Abro^1^, S Raghuvir^1^, K Tsuruta^2^

#### ^1^Loyola University, Chicago, Illinois, United States; ^2^Asahi Kasei Pharma America Corporation, Waltham, Massachusetts, United States

**Introduction**

Unfractionated heparin (UFH), antithrombin (AT), and recombinant thrombomodulin (RT) represent two distinct anticoagulant/antithrombotic agents with different targets in the hemostatic process to produce their therapeutic effects. Both of these agents are widely used in various hematologic indications in mono and poly therapeutic approaches. Currently, a recombinant version of thrombomodulin, ART-123 (Recomodulin) is undergoing clinical trials to validate its efficacy in sepsis-associated coagulopathy. Recomodulin is a novel, recombinant and soluble thrombomodulin, and is a human protein with both thrombin inhibiting and protein C stimulating activities. In comparison to both UFH and AT, this agent has relatively weaker anticoagulant activities related to bleeding risk at therapeutic concentrations of < = 1.25 ug/mL. Supratherapeutic concentrations of this agent may occur in some patients with renal dysfunction. The purpose of this study is to compare the anticoagulant and platelet modulatory effects of ART-123, UFH, and AT.

**Methods**

UFH of porcine origin was obtained from Medefil Inc. (Glendale Heights, IL) in powdered form with a specific activity of 175 U/mg. A working concentrations of this agent was made at 100 ug/mL in sterile saline. AT was commercially obtained from Baxter Healthcare Corporation (Deerfield, IL). A working concentration of AT was prepared at 100 U/mL in sterile saline. Recomodulin was commercially obtained and was manufactured by Asahi Kasei Pharma (Japan). A working concentration of Recomodulin at 100 ug/mL was prepared in sterile saline. The effect of Recomodulin, AT, and UFH on the glass activated clotting time and thromboelastographic (TEG) profile was measured at concentrations of 0-5 ug/mL. Global anticoagulant assays including PT, APTT, and TT were also measured in citrated whole blood and retrieved plasma. The effect of these drugs on agonist induced platelet aggregation (arachidonic acid, ADP, collagen, thrombin, and epinephrine) was measured in platelet rich plasma collected from healthy donors.

**Results**

In comparison to both AT and UFH, Recomodulin did not produce any anticoagulant effects in either the TEG or the ACT tests at concentrations of 1.25 ug/mL. At higher concentrations of 2.5 and 5.0 ug/mL, the relative anticoagulant effects of Recomodulin were much weaker in comparison to both AT and UFH. In the TEG profile at 5.0 ug/mL, both the AT and UFH produced complete anticoagulation. However, Recomodulin did not produce a complete anticoagulation at 2.5 and 5.0 ug/mL. In the whole blood global clotting assays, all agents produced a concentration-dependent anticoagulant effect following the order UFH > AT > Recomodulin. In the platelet aggregation studies, while heparin produced a mild increase in the aggregation profile of some of the agonists, AT and Recomodulin did not produce any effects at concentrations of up to 10 ug/ml and 5 U/ml for all of the agonists except thrombin.

**Conclusions**

The circulating levels of Recomodulin for the management of sepsis-associated coagulopathy range from 0.5-1.5 ug/mL. The therapeutic levels of UFH for similar indications range from 1.5-5.0 ug/mL (0.25-1.0 U/mL), whereas the circulating AT levels may range from 1-2.5 U/mL. The results from this study suggest that Recomodulin is a much weaker anticoagulant in comparison to both UFH and AT and at therapeutic concentrations, it does not produce any measurable anticoagulant effects. At supratherapeutic concentrations of > 2.5 ug/mL, Recomodulin exhibits weaker anticoagulant effects which are unlikely to contribute to any hemostatic deficit resulting in potential bleeding complications.

## P422 Safety of thrombomodulin (ART-123) in surgical patients with sepsis and suspected disseminated intravascular coagulation (S + DIC)

### PS Barie^1^, D Fineberg^2^, A Radford^2^, K Tsuruta^2^

#### ^1^Weill Cornell Medicine, New York, New York, United States; ^2^Asahi Kasei Pharma America, Waltham, Massachusetts, United States

**Introduction**

We hypothesized that ART-123 is safe for therapy of S + DIC, particularly re: Bleeding risk. S + DIC occurs in 20%-35% of hospitalized patients with sepsis. Limited safety data exist for surgical patients receiving ART-123 for S + DIC despite substantial clinical use in Japan (>160,000 pts treated).

**Methods**

Retrospective review of a large Phase 2b, randomized, placebo-controlled trial of therapy with ART-123 to assess safety [1] in 741 treated pts. Data were analyzed for the presence and type (Table 4) of an operation within 30 d prior to randomization. On- treatment (during study drug admin or 4 d thereafter) serious major bleeding events [SMBEs] (any intracranial hemorrhage, any life-threatening bleeding, any bleeding investigator-classified as serious by the investigator, or any bleeding that required > 6 U red cell concentrates over two consecutive days, and 28-d mortality were compared. Χ^2^, p < 0.05.

**Results**

226/741 (30.5%) were surgical patients (SP). Most (80%) operations were abdominal. Primary infection source is listed in Table 5. Among non-surgical (NS) patients, SMBEs occurred in 4.8% (12/252, ART-123 group; 3 fatal) vs. 4.2% (11/263, placebo group; 4 fatal) (p = ns). Among SP, SMBEs occurred in 5.9% (7/119, ART-123 group; 1 fatal) vs. 5.6% (6/107, placebo group, 0 fatal) (p = ns). 28-day mortality among SP was 21.0% (25/119, ART-123) vs. 29.0% (31/107, placebo) (p = ns); this compares with NS mortality of 16.3% (41/252, ART-123) vs. 18.6% (49/263, placebo) (p = ns).

**Conclusions**

Similar rates of SMBEs exist between ART-123- and placebo-treated SP; ART-123 appears to be safe regarding bleeding risk after surgery. Further research is needed for SP with S + DIC to evaluate the effect of ART-123 on outcomes.

**References**

1. Vincent JL et al. Crit Care Med 41(9): 2069–79, 2013

**Table 4 (abstract P422).** See text for description

	ART-123 n = 119	Placebo n = 107	Total n = 226
Intra-abdominal	97(81.5)	84(78.5)	181(80.1)
Thoracic	6(5.0)	7(6.5)	13(5.8)
GU	2(1.7)	5(4.7)	7(3.1)
Ortho	7(4.2)	5(4.7)	12(5.3)
Cardiac	4(3.3)	4(3.7)	8(3.5)
SSSI	1(<1)	2(1.9)	3(1.3)
Head /Neck	1(<1)	0	1(<1)
UNK	1(<1)	0	1(<1)

Legend : Type of Surgical Operation

**Table 5 (abstract P422).** See text for description

	SP n = 226	NS n = 515
Lung	46(20.4)	263(51.1)
Intra-abdominal	140(61.9)	65(12.6)
Urinary	10(4.4)	108(21.0)
Miscellaneous	30(13.3)	79(15.3)

## P423 Vasopressin + Norepinephrine in septic shock: effects on renal function – a retrospective analysis

### A Casazza^1^, A Vilardo^2^, E Bellazzi^1^, R Boschi^1^, D Ciprandi^1^, C Gigliuto^1^, R Preda^1^, R Vanzino^1^, M Vetere^1^, L Carnevale^1^

#### ^1^ASST di Pavia, Vigevano, Italy; ^2^Scuola di Specialità Anestesia e Rianimazione, Università Degli Studi, Pavia, Italy

**Introduction**

Norepinephrine (N) is the first-line vasopressor in septic shock, Vasopressin (V) is used in catecholamine-resistant shock to reach intended MAP, however the dosage of N at which V should be added is unknown and outcome effects are debated. New data suggested that low dose V is effective in intermediate severity septic shock [1] and has positive effects on renal function [2].

**Methods**

We retrospectively analysed all septic shock patients admitted to our general ICU from 09/2013 to 08/2016 and treated with N or association V + N. We identified 3 different groups. In N group (low severity), patients received only N < 0.4mcg/kg/min to reach MAP > 65mmhg; in VN-L group (intermediate severity), V at a dosage of 0.02-0.03 U/min was added to N at a dosage < =0.4 mcg/kg/min; in VN-H group (high severity), despite V infusion, N needed to be titrated > 0.4 mcg/kg/min. We analysed SOFA score at admission, mortality rate and need for RRT during ICU stay, vasoactive drugs infusion length, urine output and progression of AKI by RIFLE classification during the first week of ICU stay. Patients with end stage kidney disease at admission were ruled out. Data were analysed by Anova and Fisher’s exact tests.

**Results**

We enrolled 39 patients; 16 in N group, 16 in VN-L and 7 in VN-H. SOFA at baseline was 10 in N patients, 12 in VN-L and 14 in VN-H (p < 0.05). Overall mortality was 39.5%: VN-L patients showed lower mortality rate (31.1%) than VN-H (71.4%) and N group (37.5%) (p 0.38). Need for RRT was 18.8% in N and VN-L series and 57.1% in VN-H. Urine output was higher in VN-L patients than N and VN-H ones without statistical significance (p 0.35). In N group, 3 patients without AKI at baseline didn’t develop renal failure; 9 patients (69.2%), 4 in R, 3 in I and 2 in F RIFLE categories, improved their renal function. In VN-L group, only 1 patient without AKI were admitted and didn’t worse his renal function; 12 patients (80%), 5 R, 4 I and 3 F class, improved renal function. In VN-H series, 1 patient admitted without AKI didn’t develop renal failure, and 1 I category patient (16.7%) improved, but all F patients worsened to L (p 0.40). Vasopressors infusion mean length was 5,6 days in N, 4.3 in VN-L and 10.4 in VN-H series (p < 0.05).

**Conclusions**

Although, in our analysis, several data do not reach statistical significance, the association V + N, in the intermediate severity group, seems to have favorable effects on the urinary flow, on the progression of AKI and on vasoactive drugs infusion length.

**References**

[1] Mehta et al, Crit Care 2013 Jun 20; 17(3).R117

[2] Gordon et al, JAMA.2016;316(5):509–518

## P424 Pentaglobin administration provides feature of trained immunity against multidrug-resistant (MDR) gram-negative bacteria

### E Kyriazopoulou, A Pistiki, C Routsi, I Tsangaris, E Giamarellos-Bourboulis

#### National and Kapodistrian University of Athens, Medical School, Athens, Greece

**Introduction**

Recent data of the Hellenic Sepsis Group showed that treatment of severe infections by the IgM-enriched preparation Pentaglobin delayed the advent of breakthrough bacteremia suggesting a probable trained immune effect [1]. We investigated if Pentaglobin can elicit such trained immune responses.

**Methods**

Interleukin(IL)-10 were selectively measured in serial serum samples from five patients with ventilator-associated pneumonia of a prospective cohort [2] who received Pentaglobin at the discretion of the attending physicians. Fresh peripheral blood mononuclear cells (PBMCs) were isolated from six healthy volunteers and pre-treated for 24 hours with medium or Pentaglobin so that final IgM concentration was 50mg/dl. After washing, PBMCs were stimulated for 24 hours with heat-killed 5log10-growth isolates of Klebsiella pneumoniae producing-carbapenemase (KPC) and multidrug-resistant Pseudomonas aeruginosa (MDRPA). Isolates were selected from those infecting already described Greek patients; isolates were kept frozen and thawed [1]. Tumour necrosis factor (TNF) alpha was measured in PBMC supernatants by an enzyme immnosorbent assay.

**Results**

**Fig. 15 (abstract P424).**
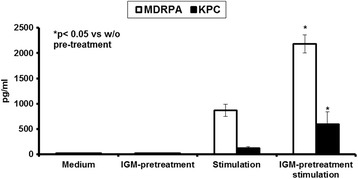
See text for description

Mean IL-10 at baseline was 58.5 +/- 46 pg/ml and on day 3 36.9 +/- 23.1 pg/ml (p: 0.043). IgM pre-treatment did not affect TNFalpha production. After stimulation with MDRPA and KPC TNFalpha was significantly higher from IgM pre-treated PBMCs (Fig. 15).

**Conclusions**

Pentaglobin pre-treatment leads to enhanced TNFα responses after re-stimulation with resistant Gram-negative hospital-acquired pathogens simulating enhanced host responses. This corroborates with the decrease of circulating IL-10 shown in patients and explains the protection from breakthrough bacteremia seen in our recent clinical study [1].

**References**

1.Giamarellos-Bourboulis EJ, et al. CMI 2016; 22: 499

2.Giamarellos-Bourboulis EJ, et al. CID 2008; 46: 1157

## P425 Long-term survival benefit of pentaglobin for severe infections by multidrug-resistant (mdr) gram-negative bacteria

### E Kyriazopoulou^1^, I Tsangaris^1^, C Routsi^1^, I Pnevmatikos^2^, G Vlachogiannis^3^, E Antoniadou^4^, K Mandragos^5^, A Armaganidis^1^, E Giamarellos-Bourboulis^1^

#### ^1^National and Kapodistrian University of Athens, Medical School, Athens, Greece; ^2^University of Thrace, Alexandroupolis, Greece; ^3^Aghios Dimitrios General Hospital, Thessaloniki, Greece; ^4^G.Gennimatas General Hospital, Thessaloniki, Greece; ^5^Korgialeneion Benakeion Hospital, Athens, Greece

**Introduction**

In a recent publication, 100 patients with severe infections by MDR Gram-negative bacteria were treated for five days with one IgM-enriched immunoglobulin preparation (IgGAM, Pentaglobin); they had favorable 28-day outcomes compared to 100 well-matched comparators for all infection variables [1]. We re-analyzed regarding long-term outcomes.

**Methods**

Patients were sub-grouped by SOFA score; survival on day 90 was compared by the log-rank test. Serial procalcitonin (PCT) measurements were available for 22 IgGAM-treated patients and 35 comparators and compared by the Mann-Whitney U test.

**Results**

**Fig. 16 (abstract P425).**
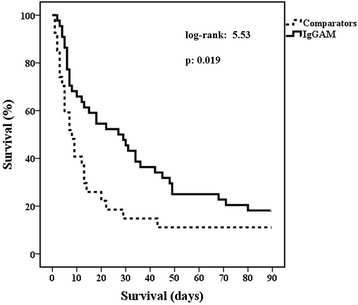
See text for description

In the subgroup with SOFA more than 10, survival until day 90 was prolonged with IgGAM (median 27 vs 8 days, Fig. 16). Although a trend for prolonged survival was shown with SOFA 10 or less (median 42 vs 27 days) this was not statistically significant. PCT started to decrease in the IgGAM group by day 5; median PCT of the IgGAM group was 0.17 (0.02-0.72) ng/ml and of the comparators 0.67 (0.24-13.28) ng/ml (p: 0.012).

**Conclusions**

IgGAM treatment achieved better 90-day outcomes among the more severe patients. This was associated with decrease of PCT.

**References:**

1.Giamarellos-Bourboulis EJ, et al. Clin Microbiol Infect 2016; 22: 499

## P426 Medical application content quality metrics: a cross-sectional survey of current applications used to aid the management of sepsis

### P Allan^1^, R Oehmen^2^, J Luo^1^, C Ellis ^1^, P Latham^3^, J Newman ^4^

#### ^1^Rockingham General Hospital, Cooloongup, Australia; ^2^University of Notre Dame, Fremantle, Australia; ^3^Mackay Hospital, Mackay, Australia; ^4^Queen Alexandra Hospital, Portsmouth, United Kingdom

**Introduction**

The use of mobile applications (apps) by healthcare practitioners is increasing globally [1]. While this has the potential to improve clinical knowledge translation, it also represents a potential source of medical risk due to a lack of both regulation and validation. In this study, we attempt to assess the quality of currently available apps relating to the management of sepsis.

**Methods**

Between the 15th and 19th of April, 2016 a detailed search was completed of all apps available on the two largest global app stores relating to sepsis. Search terms ‘sepsis’, ‘septic’ and ‘septic shock’ were used. Matching apps were assessed against set inclusion criteria such as ‘English language only’, “not a game”. Apps meeting these criteria were then purchased and reviewed for further metrics including intended audience, and content design. Apps not intended for medical practitioners and those not relating to the management of sepsis were further excluded. Remaining apps underwent a detailed review of content including the representation of international sepsis guideline information, author metrics, references, legal disclaimers as well as update and review processes.

**Results**

Search terms returned 236 unique apps. 19 apps passed all barrier conditions for final review. Of these, 15 were information references only and did not modify management information based on user entered patient data. Of the 4 apps that did, only 2 offered a commitment to maintaining privacy. Reference apps were typically not specific to sepsis management but rather covered a broad range of diseases. 5 apps were electronic versions of published medical textbooks. Instances of information conflicting with guidelines was common. Only 12 apps included references. Periodical updates were evident in the majority of surveyed apps, but only 8 apps had been updated within 12 months. Legal disclaimers outlining liability were observed in only 8 of 19 applications. No application explicitly mentioned the presence or absence of any conflicts of interest.

**Conclusions**

The quality of surveyed medical apps concerning sepsis management varied greatly. The potential for clinical risk is high due to the lack of regulation governing currently available medical apps. The development of designer, consumer and market-based processes to regulate medical app content is important as this technology is increasingly integrated into modern clinical practice.

**References**

1.Wyatt, J. C. et al. Clin Med. (Northfield. II). 15, 519–521 (2015)

## P427 Simulating sepsis: can it improve delivery of the sepsis six in the emergency department?

### C Pritchett, D Pandya, A Cripps, S Harris, M Jadav, R Langford

#### Royal Cornwall Hospital, Truro, United Kingdom

**Introduction**

In this study we assessed the effectiveness of our sepsis simulation based education (SBE) course using the Kirkpatrick learning evaluation model [1] in a cohort of Emergency Department (ED) physicians. Few simulation articles have demonstrated change in participant’s behaviour (Kirkpatrick level 3) [2]. Such evidence demonstrates translation of learning into practice. Delivery of the ‘sepsis 6’ within 1 hour reduces mortality and morbidity of septic patients [3]. At the Royal Cornwall Hospital we have instigated multi-disciplinary sepsis study days to improve care of septic patients.

**Methods**

8 ED Drs and 3 nurses received training in sepsis management and human factors followed by 2 in-situ simulated sepsis scenarios in the ED. Data was collected relating to Kirkpatrick level. Level 1 data assessed participant views on delivery, content and relevance using Likert scales. Improvement of knowledge was assessed using a questionnaire before & after the course. To assess behaviour change of the 8 physicians in attendance, time to completion of each of the sepsis 6 steps was collected for all septic patients they cared for 2 weeks before and 2 weeks after the course. Data was obtained from the ED management tool ‘Oceano’, and patient electronic records.

**Results**

Kirkpatrick level 1: Overall satisfaction was high with averages of 4.5/5 for the sepsis seminar & simulation and 4.3/5 for the human factors seminar. Kirkpatrick level 2: Knowledge of sepsis improved from pre-to post test scores by an average of 4.8 marks out of 20. Kirkpatrick level 3: The 8 ED physicians who attended training treated 37 septic patients 2 weeks prior to training and 15 septic patients 2 weeks following training. Documented delivery of all sepsis 6 components improved from 32% to 53% (p = 0.17). Documented delivery of all 6 components within 1 hour improved from 8% to 33% (p = 0.0001).

**Conclusions**

Our course has demonstrated that participants enjoy and gain knowledge in sepsis and human factors in the context of SBE. Importantly it has demonstrated a statistically significant improvement of sepsis 6 delivery within 1 hour.

**References**

1. Kirkpatrick et al. Evaluating Training Programmes: the four levels. 3rd edition. San Francisco (CA): Berrett-Koehler; 2006

2. Boet S et al. Transfer of learning and patient outcome in simulated crisis resource management: A systematic review. Can J Anesth (2014) 61: 571–582

3. Dellinger RP et al, Surviving sepsis campaign: international guidelines for management of severe sepsis and septic shock: 2012. Crit Care Med. 2013 Feb;41(2):580–637

## P428 Quick sequential organ failure assessment compared to systemic inflammatory response syndrome for predicting sepsis in emergency department

### B Ko, H Park

#### Kyung Hee University Hospital at Gangdong, Kyung Hee University School of Medicine, Seoul, South Korea

**Introduction**

It is unclear whether qSOFA also has prognostic value for organ failure in patients with suspected infection. The aim of this study was to determine the prognostic value of qSOFA compared to systemic inflammatory response syndrome (SIRS) for predicting organ failure in patients with suspected infection in emergency department (ED).

**Methods**

We retrospectively reviewed the medical records of 3234 patients with suspected infection in ED of a university-affiliated hospital during a 10-year period. We analyzed the ability of qSOFA compared to SIRS for predicting organ failure development (defined as an increase in the SOFA score of 2 point or more) in patients admitted at ED using area under receiver operating characteristic (AUROC) curve.

**Results**

A total of 1009 patients with suspected infection finally included in the study, 627 (62.1%) experienced organ failure development within 24 hours after ED admission. The predictive validity of qSOFA for organ failure was higher than that of SIRS (AUROC = 0.814; 95% CI: 0.72-0.91 vs. AUROC = 0.662; 95% CI: 0.58-0.75; p = 0.02). The qSOFA was also superior to SIRS for predicting in-hospital mortality (AUROC = 0.733; 95% CI: 0.64-0.83 vs. AUROC = 0.599; 95% CI: 0.51-0.69; p = 0.04). When qSOFA score was equal to or greater than 1, its sensitivity and specificity for predicting organ failure were 75% and 82%, respectively.

**Conclusions**

qSOFA can predict the occurrence of organ failure in patients with suspected infection. It has a superior predicting ability than SIRS. Further prospective study about the optimal cutoff value of qSOFA for predicting organ failure is warranted.

**References**

1. Bone RC, Balk RA, Cerra FB, et al. Definitions for sepsis and organ failure and guidelines for the use of innovative therapies in sepsis. The ACCP/SCCM Consensus Conference Committee. American College of Chest Physicians/Society of Critical Care Medicine. Chest. 1992;101(6):1644–1655.

2. Sprung CL, Sakr Y, Vincent JL, et al. An evaluation of systemic inflammatory response syndrome signs in the Sepsis Occurrence In Acutely Ill Patients (SOAP) study. Intensive Care Med. 2006;32(3):421–427.

## P429 Influenza-associated risk factors for ICU admission and mortality

### CM Beumer^1^, R Koch^1^, D V Beuningen^1^, AM Oudelashof^2^, FL Vd Veerdonk^1^, E Kolwijck^1^, JG VanderHoeven^1^, DC Bergmans^2^, C Hoedemaekers ^1^

#### ^1^UMC Nijmegen, Nijmegen, Netherlands; ^2^Maastricht University Medical Centre, Maastricht, Netherlands

**Introduction**

Influenza is associated with high morbidity and mortality rates worldwide. The severity of influenza infections is increasing over the last years. In this study we aimed to identify risk factors for ICU admission and mortality for influenza patients during the 2015-2016 epidemic in The Netherlands.

**Methods**

We performed a retrospective cohort study in influenza patients who were hospitalized in two university hospitals during the 2015-2016 influenza epidemic. Cases were identified using databases of the microbiology departments at both medical centers. Patients admitted to the hospital with clinical symptoms due to an acute infection with Influenza A or B were included in this study. Virus samples were obtained from nose/throat swab, sputum collection or bronchoalveolar lavage. A laboratory confirmed novel influenza infection was defined as a positive polymerase chain reaction, immunofluorescence assay or simple culture for either influenza A or B. In patients where a rapid influenza test was initially used, one of the above laboratory techniques subsequently had to be positive before a patient could be included in the study as a case.

**Results**

We identified 200 cases with influenza type A or B admitted to one of the index hospitals. Data of 1 patient was of poor quality and excluded from further analysis. Overall mortality was 9%. 45 patients (23%) of the patients were admitted to the ICU, either primarily or later during the disease. Risk factors for ICU admission were smoking (p = 0.01), a history of OSAS/CSAS (p = 0.03) and myocardial infarction (p = 0.007). The development of renal failure and secondary (pulmonary) infections were also associated with ICU admission (P < 0.001). 17 of the 45 (38%) patients admitted to the ICU died during admission. Risk factors for ICU mortality included diabetes (p = 0.04) and renal failure before and during ICU admission (p = 0.01). In 25 out of 45 patients (55.6%) a secondary pulmonary infection developed. The most common bacterial pathogens included staphylococcus aureus (11.1%) and streptococcus pneumoniae (6.7%). Among the secondary fungal infections, Aspergillus fumigatus was most common (17.8%), followed by pneumocystis jirovecii (6.7%). Patients with a secondary infection received oseltamivir more often (100% vs. 65.0%, p = 0.002). No association was observed between other immunosuppressive drugs and the development secondary pulmonary infections.

**Conclusions**

Development of secondary pulmonary infections is a frequent complication in patients with influenza and associated with an increased risk of ICU admission. Early identification and treatment of these patients may prevent ICU admission.

## P430 Infection rate during therapeutic hypothermia in children

### JB Brandt, J Golej, G Burda, G Mostafa, A Schneider, R Vargha, M Hermon

#### Medical University of Vienna, Vienna, Austria

**Introduction**

Several studies showed neuroprotective and immunomodulatory mechanisms of therapeutic hypothermia (TH). TH negatively affects leucocyte migration and synthesis of cytokines and is therefore thought to enhance infection rate due to impaired immunosurveillance. However, data for TH in children is limited. Therefore, we analyzed incidence of infections and outcome of children treated with TH (TH-group) and compared them to normothermic patients (NT- control group).

**Methods**

This study was performed as a retrospective case control study (from 2000 to 2012). All medical records and laboratory files of patients (newborns and children until 18 years of age), receiving TH for 24 to 72 hours, were screened and compared to a NT-group (historic control group with conventional treatment). Both groups were matched according to diagnosis and age. Data was analyzed from the day of admission to ICU until day 6. Indications for TH-initiation were cardiac arrest, peripartal asphyxia, traumatic brain injury, ischemic stroke, cerebral haemorrhage, -edema or –seizures, as well as acute liver failure. Patients with evident infection, medical history of an immunodeficiency disorder and / or an immunosuppressive therapy, before onset of TH, were excluded. TH (32-34°C) was induced by using a non-invasive cooling device. After 72 hours rewarming was started (0.2-0.3°C/hour).

**Results**

108 patients were included (TH-group n = 27, NT-group n = 81). Survival rate showed no significant difference (81% of TH and 78% of NT, p = 0.996). CRP elevation (>1.2 mg/dL) was earlier in the TH-group and showed a significant difference on day 6 (4.93 mg/dL) to NT-group (2.36 mg/dL, p = 0.007). 22,2% of patients in the TH-group had culture proven infections in comparison to 4% within the NT-group. Five patients in the NT-group showed only clinical signs of infection. Pneumonia was the most commonly culture proven infection within both groups (TH 14.8% and NT 6.2%).

**Conclusions**

We report that therapeutic hypothermia did not significantly alter overall survival and length of hospital stay in our pediatric center. Similar to previous observations [1], our results showed a significant increase of CRP levels in TH patients as compared to NT controls, and had more culture proven infections. This data underlines the necessity of continuous monitoring for possible infectious complications when TH is used in pediatric intensive care setting.

**References**

1. Okumus, N. et al.: Am. J. Perinatol. 32: 667–674 (2015)

## P431 The role of the ICU in the spread of Acinetobacter baumannii through the hospital

### P Levin^1^, C Broyer^1^, M Assous^1^, Y Wiener-Well^1^, M Dahan^1^, S Benenson^2^, E Ben-Chetrit^1^

#### ^1^Shaare Zedek Medical Center, Jerusalem, Israel; ^2^Hadassah Hospital, Jerusalem, Israel

**Introduction**

Nosocomial acquisition of multidrug resistant Acinetobacter baumannii in the ICU is common. Despite a high fatality rate, over 50% of ICU Acinetobacter carriers are discharged alive from ICU to hospital wards where they continue to represent a source for cross transmission. We describe an intervention to terminate an ICU Acinetobacter outbreak and investigate the effect on hospital wide Acinetobacter prevalence.

**Methods**

ICU Acinetobacter incidence and prevalence were detected from surveillance and clinical cultures. Hospital prevalence was determined from clinical cultures. The Acinetobacter control intervention included unit closure with intense environmental cleaning, a hand hygiene intervention, improved cleaning protocols and the use of virtual walls. Data for the year preceding and following the intervention were compared for (1) ICU admission prevalence and ICU acquisition of Acinetobacter, (2) hospital prevalence of Acinetobacter and (3) Colistin use.

**Results**

In the year prior to the intervention (6/2014 – 5/2015), Acinetobacter was isolated during 65/513 (13%) ICU admissions. Acinetobacter was isolated within 48 hours of ICU admission on 32/65 (49%) occasions and acquired in the ICU thereafter on 33/65 (51%) occasions. Acinetobacter positive patients were discharged alive to the wards on 48/65 (74%) occasions. In the year following the intervention (6/2015-5/2016) Acinetobacter was cultured during 4/516 (0.8%) ICU admissions (p < 0.001 vs preintervention), only one patient acquired Acinetobacter in the ICU and one Acinetobacter positive patient was discharged alive to the ward. In the hospital at large, Acinetobacter prevalence decreased by 58% from 185/39421 (0.5%) to 107/40292 (0.3%) admissions (p < 0.001). ICU and non-ICU hospital Colistin use (in defined daily doses/1000 patient days) decreased from 250 to 68 (p < 0.001) and 21 to 12 (p < 0.001) respectively.

**Conclusions**

The ICU intervention decreased ICU acquisition of Acinetobacter and ICU discharge of Acinetobacter positive patients to the wards and was associated with a decrease in hospital prevalence of Acinetobacter. Numerically, the decrease in hospital prevalence (from 185 to 107 patients) exceeded the decrease in ICU discharge of Acinetobacter patients (from 48 to 1). This could suggest that decreased ICU discharge of Acinetobacter positive patients leads to a lower Acinetobacter load in the wards and thus decreased transmission in the wards. The decreased load of Acinetobacter also facilitated a decrease in Colistin use. In conclusion, control of an ICU Acinetobacter outbreak had positive ramifications throughout the hospital.

## P432 Observational study of central venous catheter care and reasons for removal

### A Faux^1^, R Sherazi^1^, A Sethi^2^, S Saha^1^

#### ^1^Queens Hospital, Essex, United Kingdom; ^2^King George Hospital, Ilford, United Kingdom

**Introduction**

Vascular catheters are a ubiquitous tool in the critical care setting; however their use does not come without risks. Potentially one of the most serious and preventable complications is catheter-related blood stream infection (CR-BSI). The aim of this study was to examine the reasons why central venous catheters (CVC) are removed, whether there is clinical improvement following removal, and see how this relates to rates of CR-BSI.

**Methods**

We retrospectively studied the insertion, care, and reason for removal of central venous catheters across a single NHS trust. This data was then matched with the corresponding biochemical and microbiological results for each patient. Suspected CR-BSI was defined as clinical evidence of sepsis and with no apparent source of septicaemia except catheter tip colonization. Confirmed CR-BSI was subsequently defined by colonisation of a catheter tip with the same micro-organism grown from a peripheral blood culture. A routine change of CVC or arterial catheter was defined as replacement without evidence of sepsis or problems with the functioning of the catheter.

**Results**

A total of 87 CVCs were studied with an average duration of insertion of 5.5 days. The most common reason for removal was absence of indication (28.7%). 17 out of 87 (19.5%) of CVCs were removed due to suspicion of CR-BSI, and of these only 1 was confirmed (Table 6). We further examined the cultured catheters for evidence of biochemical improvement following removal (Table 7). Of those who experienced improvement of inflammatory markers following CVC removal, we found no difference in mean duration of insertion, and similarly they were no more likely to have colonised CVCs than patients who did not experience improvement of their inflammatory markers (25% vs 29%).

**Conclusions**

In the NHS trust studied we found that 16.1% of central venous catheters were routinely changed after an average of 7.1 days without obvious indication other than to prevent catheter-related infection. Although some of these lines were colonised when cultured, we did not find any biochemical benefit of removing them routinely. This is consistent with evidence from randomised trials showing that routine replacement does not reduce rates of catheter-related bloodstream infection compared with changing them when clinically indicated [1][2].

**References:**

[1] Eyer S, Brummitt C, Crossley K, Siegel R, Cerra F. Catheter related sepsis: prospective, randomized study of three methods of long-term catheter maintenance. Critical Care Medicine. 1990;18:1073–9.

[2] Cobb DK, High KP, Sawyer RG, et al. A controlled trial of scheduled replacement of central venous and pulmonary-artery catheters. New England Journal of Medicine. 1992;327:1062–8.

**Table 6 (abstract P432).** See text for description

	No longer indicated	Suspected CR-BSI	Discharged	Routine change	Death	Other
N=	25	17	17	14	11	3
Mean duration (days)	6.5	6.9	3.6	7.1	2.1	4.7
Cultured	11	17	4	12	0	1
Colonised	2	6	2	3	0	0
Positive peripheral culture	0	1	0	0	0	0

Legend 2: Colonisation rates of CVCs categorised by their reason for removal

**Table 7 (abstract P432).** See text for description

WCC + CRP 48-hours post CVC removal	N=	Mean duration (days)	Colonised	CR-BSI
Increasing trend	9	6.2	2	0
Decreasing trend	12	6.7	3	1
No change	25	6.7	8	0

Legend 2: Comparison of colonised CVCs and biochemical improvement following their removal

## P433 Elimination of LPS and cytokines from blood of septic patients with LPS-adsorber

### M Kiselevskiy^1^, E Gromova^1^, S Loginov^2^, I Tchikileva^1^, Y Dolzhikova^1^, N Krotenko ^2^, R Vlasenko^1^, N Anisimova^1^

#### ^1^Russian Cancer Research Center, Moscow, Russia; ^2^SP Botkin Hospital, Moscow, Russia

**Introduction**

LPS-adsorption by Alteco® devices allows eliminating from blood endotoxin, inflammatory mediators and activated leucocytes [1].

**Methods**

20 patients with septic shock were enrolled ingram-negative the study. The values of IL-4, IL-6, IL-8, IL-10, IL-18 in serum and washout from LPS-adsorber were measured by ELISA. Concentrations of LPS were measured by modified micro gel clot test.

**Results**

**Fig. 17 (abstract P433).**
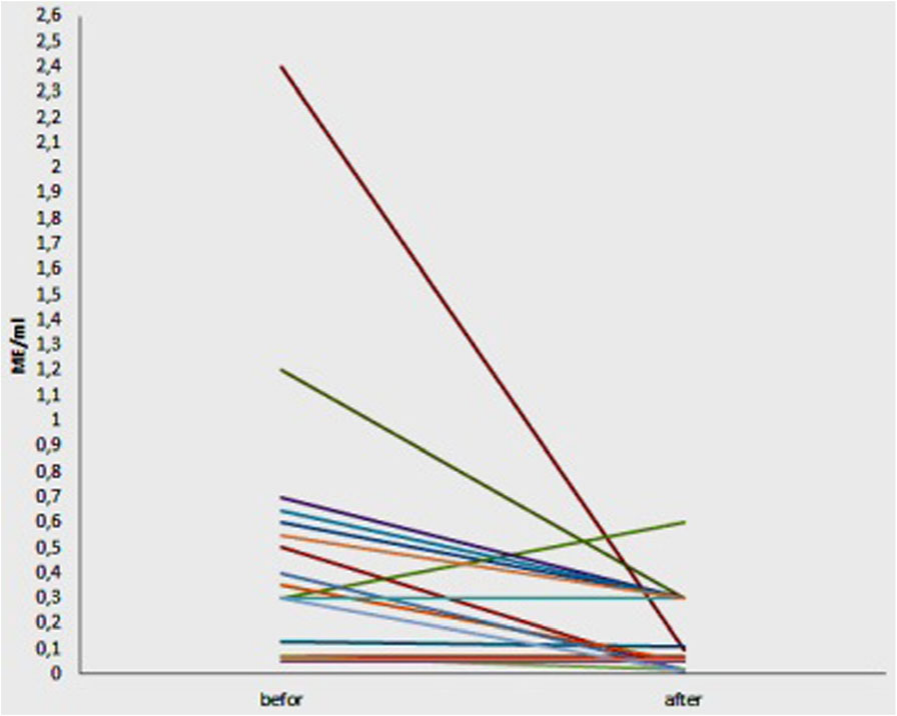
Changing the concentration of LPS in patients before and after hemoperfusion

**Fig. 18 (abstract P433).**
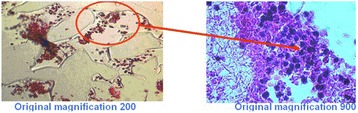
Adhesion of activated neutrophils on the Alteco hemofilter

LPS level in blood significantly reduced after the hemoperfusion procedure (equally or more than twofold) in 50% of the patients with its initially high level (Fig. 17). Significant reduction of serum LPS level required from 2 to 6 hemoperfusion procedures. We did not detect any changes in the patients with initially low level of LPS. IL-6 and IL-8 showed the most prominent concentration changes among the cytokines under the study. Their level dropped down from 2 to 10 times after the procedure in many of the patients with initially high level of the cytokines. Number of functionally active leucocytes with high phagocytic activity and enhanced expression of CD11c adhesion molecules decreased after the hemoperfusion procedure in the patients. Substantial part of the leucocytes adhered on the Alteco hemofilter plates (Fig. 18).

**Conclusions**

Hemoperfusion procedure allows eliminating from the blood not only LPS, but excess inflammatory mediators as well. Adhesion of activated neutrophils on the Alteco hemofilter allows eliminating from the bloodstream of septic patients the most reactogenic phagocytes releasing cytotoxic free radicals.

**References**

1. Adamik B. Arch Immunol Ther Exp 2015;63:475–83

## P434 Early appropriate empirical therapy is associated with increased survival rate in patients with abdominal sepsis

### S Spadaro, A Fogagnolo, F Remelli, V Alvisi, A Romanello, E Marangoni, C Volta

#### Sant’Anna Hospital, Ferrara, Italy

**Introduction**

Sepsis is the most common cause of death by infection. In particular, abdominal sepsis is characterized by the highest death ratio and a complicate management, because of the high variability of microorganisms involved and the growing problem of antibiotic resistance. Therefore, the aim of this study is to identify potentially modifiable risk factor of death in patients with abdominal sepsis.

**Methods**

This is a prospective-observational study conducted in ICU at Sant’Anna Hospital, Ferrara. All patients >18 years admitted with the diagnosis of abdominal sepsis, according to 2016 sepsis criteria, were enrolled. We collected clinical and demographic data for each patient. Moreover, we recorded laboratory and blood gas analysis data microbiological investigations, anti-infective treatment (antimicrobial therapy/source control) and fluid balance. Primary outcome was 90-days mortality.

**Results**

**Fig. 19 (abstract P434).**
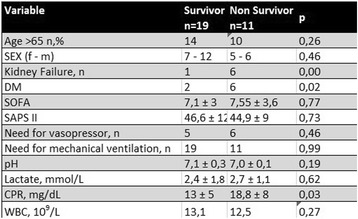
See text for description

Thirty patients were enrolled. Clinical and demographic variable are shown in Fig. 19. The 61% of isolated microorganisms were multiresistant (MDR). Mortality in patients affected by MDR bacteria was not increase (36% vs 35%; p = 0.91). However, an appropriate empirical antibiotic therapy in the first 12 hours was found to be associated with a decreased mortality from 70% to 12% (p = 0.01). Moreover, positive fluid balances for more than 72 hours were more frequent in non-survivor patients (47% vs 18% p = 0.04).

**Conclusions**

In our population, MDR bacteria were highly represented. However, not the resistance itself, but the failure of an appropriate early empirical therapy was strongly associated with increased mortality; therefore, the knowledge of the local epidemiology of microorganisms and of their drug-resistance is crucial. Moreover, the inability to get a null or negative fluid balance after the first 72 hours in the ICU is a risk factor for 90-days mortality.

**References**

Singer et al.JAMA 2016;315:801–810

## P435 Addressing empirical antimicrobial therapy in bacterial pneumonia: a new heterogeneous scoring system

### A Degrassi, F Mearelli, C Casarsa, N Fiotti, G Biolo

#### University of Trieste, Trieste, Italy

**Introduction**

Health care associated pneumonia (HCAP) ability to identify pneumonia sustained by multi drug resistant pathogens is controversial [1]. We sought to provide a new scoring system addressing the empirical antimicrobial therapy based on the correct identification of resistant pathogens in patients with pneumonia.

**Methods**

In this prospective observational study, we considered 93 adult patients with microbiological confirmation of pneumonia. We looked for the prevalence of Community-Acquired Pneumonia-Drug-Resistant Pathogens (CAP-DRP), among patients classified and treated as having CAP or HCAP. The primary goal was to assess whether the appropriateness of initial empiric therapy would affect the short term mortality and if we could improve it with a new scoring system.

**Fig. 20 (abstract P435).**
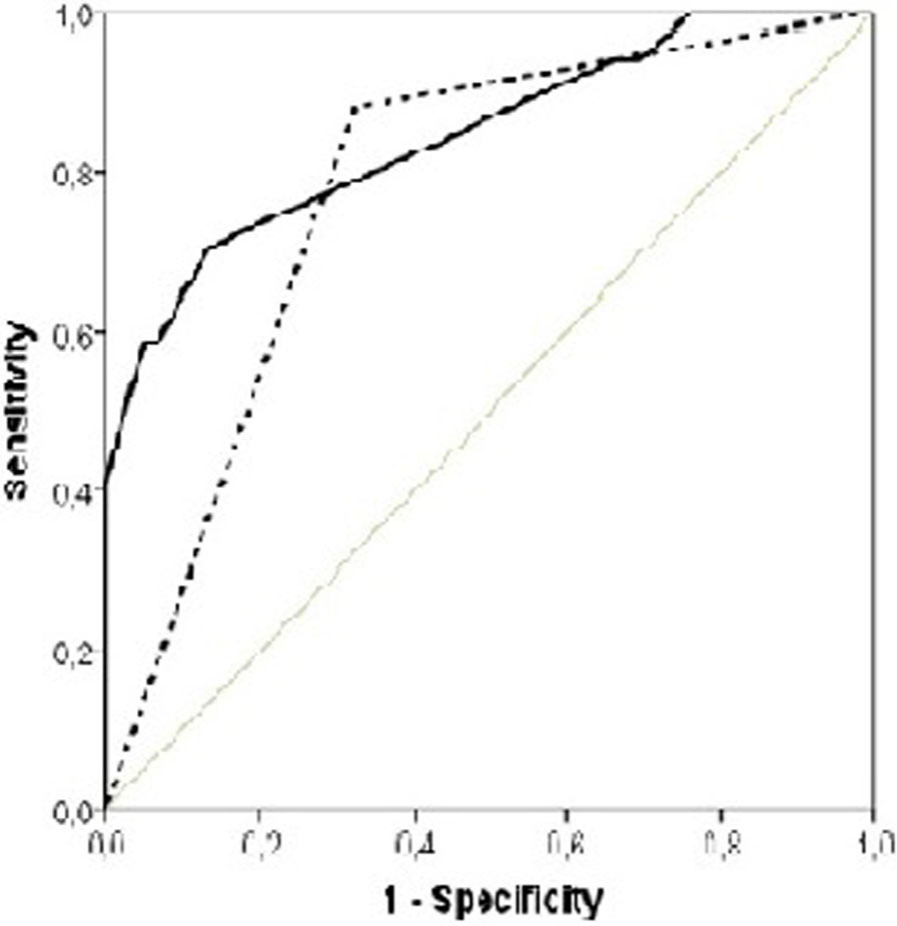
See text for description

**Results:** We considered 51 CAP and 42 HCAP patients with microbiological confirmation of infection. Receiving inappropriate empirical antimicrobial therapy was an independent risk factor for 30 days’ mortality (P = 0.018, OR 5.32 [95%C.I. 1.26-22.73]). Among 76 bacteria-sustained pneumonias, 22.3% had CAP-DRP. After applying HCAP definition, we found 4.7% of CAP-DRP among CAP, and 44% among HCAP. Previous hospitalization, residence in nursing home or long-term facilities, non-ambulatory status and usage of PPI/antiH2 were predictors of CAP-DRP pneumoniae and were turned into an equation that identifies them with AUC of 0.847 [95%C.I. 0.732-0.962], performing better than HCAP model (AUC = 0.780, 95%C.I. 0.663-0.898). Figure 20. DRP score: solid line, HCAP model: dashed line. DRP score = (hospitalization*4.743) + (nursing home residence*4.31) + (non-ambulatory status*-2.444) + (use of PPI/antiH2*-2.361). A score higher than 0.933 is a predictor of CAP-DRP.

**Conclusions:** HCAP-addressed empirical therapy leads to overtreatment and undertreatment of a considerable percentage of patients with pneumonia. Among them, the score system we propose may help to choose the most appropriate empiric antimicrobial therapy, aiming at reducing inappropriate initial therapy and its associated mortality.

**References:** [1] Chalmers JD et al. Clin Infect Dis 2014; 58(3): 330–9

## P436 Implementation of strategies of antimicrobial stewardship program in an intensive care unit in a university hospital in Chile

### M Cariqueo, C Luengo, R Galvez, C Romero, R Cornejo, O Llanos, N Estuardo, P Alarcon

#### Hospital Clínico Universidad de Chile, Santiago, Chile

**Introduction**

Antimicrobials are the 51% of the drugs used in intensive care units (ICUs) [1] and antimicrobial resistance has increased dramatically in recent years. Antimicrobial stewardship (AMS) programs aims to establish strategies to improve the use of antimicrobial and reduce resistance, adverse effects, Clostridium difficile infections and cost [2]. International guideline proposes restrictive, persuasive and structural strategies. In Chile, the feasibility of these programs are not totally studied. We showed a process of implementation AMS programs in our ICU.

**Methods**

We conducted a 3 steps process of implementation of AMS program. We reviewed the literature to identify AMS strategies published from 2010 to 2016, inclusion criteria were studies in adult patients, high complexity hospitals or ICU, and have description of the strategies in their results. A local survey was applied to 8 health care professional to evaluate the feasibility to implement the strategies found, this survey consists of positives, neutral and negative answers and if the strategy counts with a half of positive answers we considered a possible implementation. A team with clinical pharmacist, intensivist and infectious diseases physicians worked on the set to develop the optimal way to implement these strategies in our ICU.

**Results**

**Fig. 21 (abstract P436).**
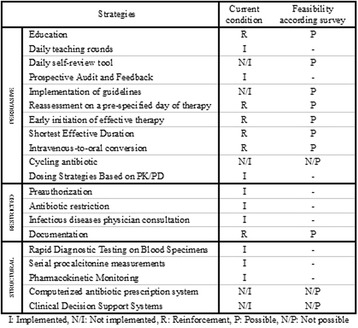
AMS strategies in ICU

We found 362 studies about AMS program, in which 49 included 21 strategies in ICUs. Strategies were classified in restrictive, persuasive and structural. In our unit 9 of those are already implemented. According to local survey 7 strategies were feasible to implement (Fig. 21). Implementation was carried out through two processes: Medical record optimization and local guideline of antimicrobial treatment.

**Conclusions**

With a baseline of 9 strategies we implemented 7 additional. This study is the first step to establish AMS policy in our ICU. The next step is made assessment of the clinical impact of our AMS policy.

**References**

[1] Hernández-Gómez et al. Biomédica; 34 (1): 91–100, 2014

[2] Barlam TF et al. CID 15;62(10): e51–77, 2016

## P437 Carbapenem resistant enterobacteriaceae (CRE) intraabdominal infections (IAI)

### B Magazi ^1^, S Khan^1^, J Pasipanodya^2^

#### ^1^MSD, Midrand, South Africa; ^2^BRI, Dallas, Texas, United States

**Introduction**

To better inform infection control and antibiotic stewardship programs, we investigated carbapenem resistance trends and assessed the molecular characteristics of β -lactamases (ESBLs, AmpC β -lactamases and carbapenamases) among Enterobacteriaceae isolates from IAI patients treated in South African hospitals participating in the Study for monitoring Antimicrobial Resistance Trends (SMART) program 2010 and 2015.

**Methods**

Cochran-Armitage test was used to examine trends in susceptibility. Classification and regression trees (CART) were used to identify minimum inhibitory concentration (MIC) thresholds of various drugs as well as other factors predictive of phenotypic CRE. EUCAST version 6 MIC interpretive criteria were used to identify non-susceptible isolates. (4) Isolates recovered <48 hours after hospitalization were considered community-associated infections (CAI); while those recovered > =48 hours were considered healthcare-associated infections (HAI).

**Results**

**Fig. 22 (abstract P437).**
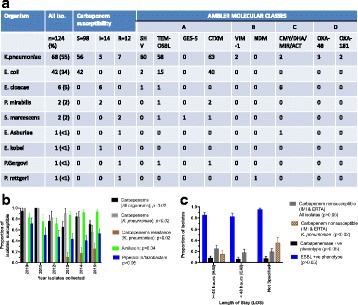
See text for description

Of the 124 isolates 109 (88%) were phenotypically ESBL, 122 (98%) of these had one or more β -lactamases and ampC genes identified and 98 (79%) were fully susceptible to the carbapenems (Fig. 22). Figure 22A shows that K.pneumoniae (68/124) and E coli (42/124) were the majority isolates contributed most of the drug resistance genes identified. Carbapenem susceptibility significantly declined by an average of 35% (2-80), driven in part by increase in resistance in K pneumoniae isolates (Fig. 22B). On the contrary, susceptibility to amikacin significantly increased, while that of piperacillin/tazobactam decreased to 59%. Of the 26 isolates nonsusceptible to carbapenems; 6 (23%) were CAI and were 12 (46%) were HAI; p = 0.468 (Fig. 22C). CTXM-15 lactamase was the most frequent gene identified I 85/124 (65%) of isolates; but, was found in combination with other genes 67/81 (83%) of the time. In fact, the odds for nonsusceptibility to carbapenems rose 26-folds (3-245) when 4 genes were present compared to isolates with only one drug resistance gene was present for all isolates.

**Conclusions**

CTXM-15 carrying K pneumonia are emerging as causes of CRE associated IAI in South Africa. Piperacillin/tazobactam is an unlikely substitute antibiotic for this patient cohort given declining susceptibility trends and overall poor activity.

## P438 Intraosseous administration of antibiotics during experimental septic shock

### M Eriksson^1^, G Strandberg^1^, M Lipsey^1^, A Larsson^2^

#### ^1^Surgical Sciences, Uppsala, Sweden; ^2^Medical Sciences, Uppsala, Sweden

**Introduction**

Intraosseous (IO) access may be lifesaving in medical emergencies, when conventional vascular access is difficult to achieve. In life-threatening infections, early administration of antibiotics is crucial. In septic shock, the circulation is markedly compromised. We wanted to elucidate whether sufficient blood levels are reached, when antibiotics are administered IO [1].

**Methods**

A model of endotoxemic shock was used, where 8 anaesthetized, extensively monitored, pigs were given a continuous infusion of E. Coli endotoxin over 6 hour period, after which, the animals were sacrificed. The Animal Ethics Committee of Uppsala University, Sweden, approved the experiment. IO access was achieved in the tibial bone (EZ-IO®, Teleflex Medical, Morrisville, NC, USA). Cefotaxime at 75mg/kg and gentamicin at 7 mg/kg, respectively were administered IO or intravenously (IV). Central concentrations of these antibiotics were measured at 5, 15, 30, 60, 120, and 180 min.

**Results**

After starting the endotoxin infusion, most of the animals showed signs of hemodynamic instability with reduced mean arterial blood pressure (MAP), cardiac index and markedly elevated mean pulmonary arterial pressure. All animals except one, needed norepinephrine to keep MAP >60 mm Hg. The plasma concentrations of both cefotaxime and gentamicin, when injected IO and IV, respectively, were nearly identical with similar slopes showing decreasing plasma concentration of each antibiotic by time. There were no significant differences between either antibiotic, regardless of injection site, based on analysis of variance. The 95% confidence intervals regarding the difference of means for each antibiotic, were well within the relevant intervals.

**Conclusions**

This study strongly suggests that IO access may be an appropriate alternative to IV administration of antibiotics, in some severe infectious conditions (e.g. septic shock), when venous access is difficult to achieve. Relevant concentrations of these antibiotics were achieved even in severely circulatory comprised animals. Thus, we deduce that IO antibiotic administration also may be considered in the prehospital setting, as early systemic administration of antibiotics may be lifesaving [2].

**References**

1. Strandberg et al. AAS 59: 346–53, 2015

2. Kumar et al. Crit Care Med 34:1589–96, 2006

## P439 Cycling aminoglycosides in critical care: a change to antimicrobial policy

### Z Rajput, F Hiscock, T Karadag, J Uwagwu, S Jain, A Molokhia

#### Lewisham and Greenwich NHS Trust, London, United Kingdom

**Introduction**

Our study was carried out to audit aminoglycoside resistant patterns and assess the need to cycle aminoglycosides. The current trust antimicrobial policy at Lewisham (UHL) and Greenwich (QEH) NHS Trust advises the use of gentamicin as a first line agent against gram-negative organisms except in severe sepsis where it recommends amikacin. Antimicrobial cycling has a role in critical care due to the increasingly rapid spread of resistant bacteria, influencing healthcare costs and mortality [1][2]. Of note, the rotation of gentamicin and amikacin has been found to reduce gentamicin resistance in some studies [3].

**Methods**

This was a 3-month retrospective, cross-site study from 1/8/16 – 31/10/16. Data was obtained from UHL and QEH critical care units. Resistance patterns of samples positive for gram-negative organisms were analysed using WinPath. The total number of resistant organisms were found, and within this cohort; the number of gram-negative organisms resistant to both gentamicin and amikacin. The proportion of extended spectrum beta lactamase (ESBL) and AmpC positive organisms were also analysed.

**Results**

4398 samples were obtained; 2822 (64%) from UHL and 1576 (36%) from QEH. 68% of gram-negative organisms were sensitive to all antimicrobials. Within the 3 month period 1462 (33.2%) of samples displayed antibiotic resistance; of which 4% of organisms were found to be resistant to gentamicin.

**Conclusions**

This audit has demonstrated a significant number of gentamicin resistant organisms. However this does not reach the national threshold for cycling. We have noted the need to reinforce the use of amikacin as a first line aminoglycoside in all critically ill patients.

**References**

[1] Kollef M. Clinical Infectious Diseases. Vol 43: suppl 4: 82–88: 2006

[2] Kollef M. Critical Care. Vol 5: No 4: 189–195: 2001

[3] Gerding D et al. American Society for Microbiology. Vol 35: 1284–1290: 1991

**Table 8 (abstract P439):** See text for description

	ESBL/AMPc	Other gram negatives
Total number of samples	78	4320
Gentamicin sensitive-GS/ Amikacin sensitive-AS	45	4266
Gentamicin resistant- GR/ AS	33	48
GR/ Amikacin resistant-AR	0	6
GS/ AR	0	0

Legend 1: Results

## P440 Continuous infusion of linezolid in critically ill patients: optimizing the dosage regimen through a PK/PD analysis

### H Barrasa^1^, A Soraluce^2^, E Uson^1^, A Rodriguez^2^, A Isla^2^, A Martin^1^, B Fernández^1^, F Fonseca^1^, JA Sánchez-Izquierdo^3^, FJ Maynar^1^

#### ^1^Hospital Universitario Alava - Santiago, Vitoria, Spain; ^2^University of the Basque Country UPV/EHU, Vitoria, Spain; ^3^Hospital Universitario Doce de Octubre, Madrid, Spain

**Introduction**

Linezolid (LZ) is an antibiotic with time-dependent activity. An optimal antibacterial effect is achieved when plasma drug concentrations are above the MIC (T > MIC) for the entire length of treatment. Critically ill patients receiving standard doses of LZ, particularly in those with sepsis and conserved renal function (CRF), are at risk of not attaining PK/PD targets. LZ clearance (LzCl) is usually increased in these patients, often due to augmented renal clearance (ARC). Continuous infusion (CI) could offer advantages in this context [1]. The objective of this work was to study the benefit of CI of LZ in critically ill patients with CRF.

**Methods**

Study developed in two tertiary hospitals in critically ill patients in the absence of severe renal dysfunction (Creatinine clearance (CrCl) >40 ml/min). Each patient received a CI of LZ (50 mg/h) preceded by a 600 mg bolus. Blood samples were taken on days 1, 2, 3 and 4 after infusion onset. CrCl was determined on each day of the study. Treatment was considered satisfactory if steady state plasma concentration (CSS) was > =2mg/L (MIC90 of most Gram-Positive Cocci in Europe). The correlation between CrCl and LzCl was calculated. α significance level of 0.05.

**Results**

22 adult patients (64% males) were included in the study (80 samples). The mean age was 55 years (SD 19), and the average weight was 81 kg (SD 19). The median and interquartile ranges (IR) were: CrCl 119 ml/min (IR 85-168), LzCl 13 L/h (IR 6-24) and Css 3.8 mg/L (IR 2.1-7.9). 55% of patients presented ARC (CrCl > =130 ml/min) and 32% of the patients had CrCl > =165 mL/min, for at least one day. CSS was > =2mg/L in 84% of the samples. The correlation (Spearman’s Rho) between CrCl and LzCl was 0.81 (p < .001). CrCl, with a cut-off point of 165 ml/min, can predict failure in treatment (CSS <2mg/L) with a sensitivity of 92% (95% CI 74-100), specificity of 86% (95%CI 77-95) and AU ROC 0.94 (95%CI 0.87-1.0).

**Conclusions**

Despite the high CrCl values of the patients, 50 mg/h LZ CI ensures a high probability of achieving the PK/PD target if CrCl < 165 ml/min. In the presence of CrCl > =165 ml/min, a higher dose should be considered.

**References**

1. Adembri, C., et al., Linezolid pharmacokinetic/pharmacodynamic profile in critically ill septic patients: intermittent versus continuous infusion. Int J Antimicrob Agents, 2008. 31(2): p. 122–9.

## P441 Pharmacokinetics of tigecycline in liver impairment: quantification of liver function with maximal liver function capacity test (LiMAx test)

### M Kaffarnik^1^, R Alraish^1^, O Frey^2^, A Roehr^2^, M Stockmann^1^, S Wicha^3^

#### ^1^Charite Berlin Campus Virchow, Berlin, Germany; ^2^Pharmacy, Clinic of Heidenheim, Heidenheim, Germany; ^3^Uppsala University, Dept. of Pharmaceutical Biosciences, Uppsala, Sweden

**Introduction**

Tigecycline (TGC) is a key antibiotic in the therapy of intra-abdominal infections in critically ill patients. Liver failure frequently occurs in this patient collective and may lead to altered pharmacokinetics (PK) of TGC in these patients. We aimed to (i) determine the PK of TGC in patients with and without liver impairment and to (ii) compare the value of the novel maximum liver function capacity (LiMAx) test and conventional liver function markers (ALAT, Bilirubin, INR) as covariates of TGC PK.

**Methods**

Patients with and without liver impairment were included in this open, prospective cohort study. All patients received an i.v. TGC loading dose of 100 mg followed by 50 mg twice daily, administered over 0.5 h. TGC plasma concentrations were determined by HPLC after >36 h of therapy at 5 time points. On the day of plasma sampling, ALT, BIL, INR and LiMAx were determined. NONMEM® 7.3 was utilised for the pharmacometric analysis.

**Results**

**Fig. 23 (abstract P441).**
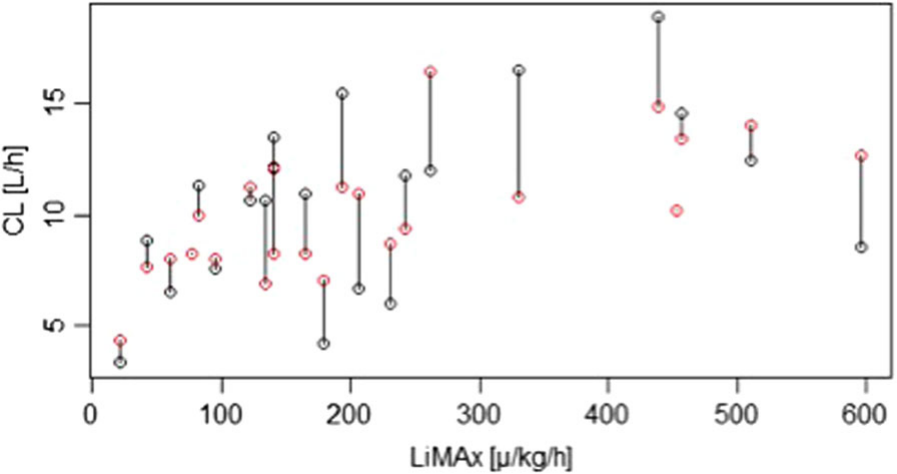
Impact of liver dysfunction on predicted and determined TGC-Clearance

23 patients contributed 114 timed plasma samples and supported estimation of a two-compartment PK model with linear elimination. LiMAx and BIL were low correlated (r2 = 0.11). Including both was significant for TGC clearance (CL) (p < =0.005), whereas ALT and INR were not significant. Parameter estimates (relative standard error) for a typical patient with LiMAx 187 μg/kg/h and BILI 2.1 mg/dL were: CL 9.7 L/h (7%), V1 72.4 L (15%), intercompartimental clearance Q 64.9 L/h (16%), V2 137 L (10%). Inclusion of LiMAx and BIL reduced the interindividual variability of CL from 43.3% to 30.1%. Determined CL were substantially influenced by liver function (Fig. 23; paired typical predicted CL (red) and individually determined CL (black) vs. LiMAx).

**Conclusions**

PK of TGC was substantially altered in patients with liver impairment. The observed variability in TGC clearance was partially explained by LiMAx and BIL. The determined covariate relationships between the liver function markers LiMAx, BIL and TGC CL sets the basis for PK-guided dosing in liver failure patients to avoid therapeutic failure or potential toxic side effects of the drug.

## P442 Susceptibility of ceftolozane/tazobactam against isolates collected from intensive care unit patients in European hospitals (2014-2016)

### D Shortridge, M Castanheira, HS Sader, JM Streit, RK Flamm

#### JMI Laboratories, North Liberty, Iowa, United States

**Introduction**

Ceftolozane/tazobactam (C/T) is an antibacterial combination consisting of a novel antipseudomonal cephalosporin and a β -lactamase inhibitor. C/T was approved by the US Food and Drug Administration in 2014 and the European Medicine Agency in 2015 to treat complicated urinary tract infections, acute pyelonephritis and complicated intraabdominal infections. The Program to Assess Ceftolozane/Tazobactam Susceptibility (PACTS) monitors C/T resistance to Gram-negative (GN) isolates worldwide.

**Methods**

A total of 3984 GN isolates were collected in 2014-2016 from 39 European hospitals’ intensive care unit (ICU) patients and tested for susceptibility by broth microdilution method in a central laboratory. The most common source was tracheal aspirate followed by bloodstream. In addition to C/T, amikacin (AMK), cefepime (FEP), ceftazidime (CAZ), colistin (COL), levofloxacin (LVX), meropenem (MER), and piperacillin/tazobactam (TZP) were tested. Antibiotic resistant phenotypes identified using EUCAST (2016) clinical breakpoints included extended-spectrum beta-lactamase (ESBL), carbapenem-resistant Enterobacteriaceae (ENT; CRE), ceftazidime nonsusceptible (CAZ-NS), meropenem nonsusceptible (MER-NS), and multidrug resistance (MDR).

**Results**

The 5 most common species isolated from ICU patients were *Pseudomonas aeruginosa* (PSA; 887 isolates), *Escherichia coli* (735), *Klebsiella pneumoniae* (674), *Acinetobacter baumannii* complex (481), and *Enterobacter cloacae* (181). For 2,402 ENT isolates, 82.7% were susceptible (S) to C/T (MIC of [<=]1 mg/L), AMK had 94.8% S, FEP 75.0%S, CAZ 69.6%S, COL 79.0%S, LVX 76.4%S, MER 95.1%S, and TZP 74.1%S. For 489 ENT with an ESBL (non-CRE) phenotype, and the most active drugs were COL (93.4%S) and MER (99.2%S). C/T had 64.6%S. A total of 571 ENT were MDR, 42.7% were S to C/T, 78.6%S to AMK, 20.1%S to FEP, 12.1%S to CAZ, 72.6%S to COL, 28.5%S to LVX, 79.5%S to MER and 21.9%S to TZP. A total of 118 ENT were CRE, and 56.9% were S to COL, 44.9%S to AMK, other agents were <13%S. For PSA, 85.9% were S to C/T (MIC [<=]4 mg/L), 80.0%S to AMK, 72.9%S to FEP, 67.8%S to CAZ, 99.9%S to COL, 57.8%S to LVX, 62.1%S to MER, and 64.7%S to TZP. The most active agents against the 300 MDR PSA were COL 99.7%S and C/T 59.0%S. Other agents were AMK 43.5%S, FEP 26.3%S, LVX 12.0%S, MER 14.7%S, and TZP 9.7%S.

**Conclusions**

C/T demonstrated good activity against ICU ENT isolates with 82.7%S. Only AMK and MER were more active. For PSA, C/T demonstrated potent activity (85.9%S) and was more active than all other agents tested except for COL.

## P443 Clinical outcomes of ceftriaxone versus nafcillin or cefazolin for the treatment of methicillin-susceptible staphylococcal aureus bacteremia

### K Falsetta, T Lam, S Reidt, J Jancik

#### Hennepin County Medical Center, Minneapolis, Minnesota, United States

**Introduction**

The objective of this study was to compare clinical outcomes of ceftriaxone (CTX) vs. nafcillin (NAF) or cefazolin (CFZ) for the treatment of methicillin-susceptible staphylococcal aureus (MSSA) bacteremia. Treatment of MSSA bacteremia typically consists of oxacillin (OXA) or NAF. Retrospective data comparing CFZ to OXA for the treatment of MSSA bacteremia suggest that CFZ is an effective alternative agent, while CTX use has yielded mixed results [1, 2, 3].

**Methods**

This retrospective chart review included patients with positive blood cultures for MSSA who received treatment with CTX, NAF, or CFZ between the months of January 2012 and September 2016. Patients were excluded for the following reasons: receiving >3 days of non-study antimicrobial agents with activity for MSSA, age <18 years, polymicrobial blood cultures, therapy for <14 days, or if adequate source control was not achieved. Patients were assigned to one of two groups: CTX therapy or standard of care therapy (SOCT) with either NAF or CFZ. The primary outcome was rate of clinical cure at end of defined therapy for the CTX group compared to the SOCT group. Secondary outcomes evaluated were: treatment failure, time to negative blood culture, time to defervescence, and occurrence of acute kidney injury (AKI). Demographic data were collected. Clinical cure, treatment failure, and occurrence of AKI were assessed with the Fisher’s exact test. Time to negative blood culture and time to defervescence were assessed with the Mann-Whitney U test. Demographic data were assessed with descriptive statistics.

**Results**

Fifty-one patients met inclusion criteria. There were no differences in clinical cure rates between CTX (81.8%) and SOCT (72.5%) (p = 0.7063). There were no differences in treatment failure between CTX (9%) and SOCT (7.5%) (p = 0.99). Median time to negative blood culture was 40 hours for CTX vs. 36.5 hours for SOCT (p = 0.45). Median time to defervescence was 13 hours for CTX and 22 hours for SOCT (p = 0.99). AKI occurred in 0 patients treated with CTX and 4 patients treated with SOCT (p = 0.5726).

**Conclusions**

In this comparison of CTX vs. SOCT for the treatment of MSSA bacteremia we found no difference in clinical cure. We also found no difference in treatment failure, time to negative blood culture, time to defervescence, and occurrence of AKI.

**References**

1. Li J et al. Antimicrob Agents Chemother. 58:5117–24, 2014.

2. Patel UC et al. Int J Clin Pharm. 36:1282–89, 2014.

3. Carr D et al. Abstract presented at IDWeek. 2015: San Diego, CA.

## P444 Development of antibiotic treatment algorithms based on gram stain to restrict usage of broad-spectrum antibiotics in the treatment of ventilator-associated pneumonia: a retrospective analysis

### T Kinoshita^1^, J Yoshimura^2^, K Yamakawa^1^, S Fujimi^1^

#### ^1^Osaka General Medical Center, Osaka, Japan; ^2^Sakai City Medical Center, Osaka, Japan

**Introduction**

Ventilator-associated pneumonia (VAP) is a common and serious problem in intensive care unit (ICU). Several studies have demonstrated that Gram stain of endotracheal aspirate may be useful for accurate diagnosis of VAP. However, the effectiveness of Gram stain on prediction of causative microorganisms has not been elucidated. The purpose of this study is to evaluate whether Gram stain of endotracheal aspirates can be used as a guide of initial antibiotic therapy for VAP.

**Methods**

Data on consecutive episodes of microbiologically confirmed VAP were collected from February 2013 to February 2016 in our ICU. We constructed 2 hypothetical empirical antibiotic treatment algorithms for VAP. The first algorithm was the guideline based algorithm (GLBA) that based on the recommendation of American Thoracic Society-Infectious Diseases Society of America guidelines. The second one was the Gram stain based algorithm (GSBA) that limited spectrum of the initial antibiotic therapy according to the results of bed-side Gram stain. Subsequently, the GLBA and the GSBA were retrospectively reviewed in the same VAP episodes. The initial coverage rates and the recommendation of broad-spectrum antibiotics was compared between the two algorithms.

**Results**

During the study period, 219 suspected VAP episodes were observed and 131 episodes were assessed for analysis. Appropriate antibiotic coverage rates were equivalent in the two algorithms (GLBA: 94.7% vs GSBA: 92.4%, p = 0.221). The number of episodes that anti-methicillin resistant Staphylococcus aureus agents were recommended as an initial treatment was larger in GLBA than GSBA (70.2% vs 31.3%, p < 0.001). Furthermore, the number of episodes that antipseudomonal agents were recommended as an initial treatment was also larger in GLBA than GSBA (70.2% vs 51.9%, p < 0.001).

**Conclusions**

The GSBA may restrict the administration of broad-spectrum antibiotics without increasing risk of treatment failure.

## P445 Efficacy of murepavadin co-administered with standard-of-care in a phase 2 study in patients with ventilator-associated pneumonia due to Pseudomonas aeruginosa infection

### A Armaganidis^1^, A Torres^2^, S Zakynthinos^3^, C Mandragos^4^, E Giamarellos-Bourboulis^5^, P Ramirez^6^, M De la Torre-Prados^7^, A Rodriguez^8^, G Dale^9^, A Wach^9^, L Beni^9^, L Hooftman^9^, C Zwingelstein ^9^

#### ^1^National and Kapodestrian University of Athens, Athens, Greece; ^2^Hospital Clinic, Barcelona, Spain; ^3^Evangelismos Hospital Medical School of Athens, Athens, Greece; ^4^Korgialenio-Benakio E.E.S, Athens, Greece; ^5^University General Hospital “ATTIKON”, Athens, Greece; ^6^Hospital Universitario Politècnic la Fe, Valencia, Spain; ^7^Hospital Universitario Virgen de la Victoria, Malaga, Spain; ^8^Hospital Universitario de Tarragona Joan XXIII, Tarragona, Spain; ^9^Polyphor Ltd, Allschwil, Switzerland

**Introduction**

Murepavadin (POL7080) represents the first member of a novel class of outer membrane protein targeting antibiotics, being developed by Polyphor for the treatment of serious infections by Pseudomonas aeruginosa (PA). This study investigated the PK, safety and tolerability of POL7080 (2.5 mg/kg, 2-hour IV-infusion; TID for 10-14 days) co-administered with standard-of-care (SoC) in ventilator-associated pneumonia (VAP) patients with suspected or confirmed PA infection. Efficacy was a secondary parameter.

**Methods**

This was a multicenter, open-label, non comparative, phase 2 study of the PK, safety, and efficacy of POL7080 when co-administered with SoC. Patients whose pneumonia was subsequently shown by culture results to be caused by PA sensitive to SoC antibiotics, were continued on treatment with SoC plus POL7080. The main efficacy endpoints included all-cause mortality (ACM) at day 28 and clinical cure at the TOC (7 ± 2 days after end-of-treatment).

**Results**

Twelve (48%) out of the 25 enrolled patients were part of the microbiological intention to treat (mITT) population: at inclusion, 11 had [>=] 10^5^ CFU/mL - PA retrieved in their endotracheal aspirate (ETA) and 1 patient had a positive blood culture. The ACM at day 28 was 92% (11 of 12 patients). Overall, 10 of 12 (83.3%) patients in the mITT analysis set had a clinical outcome of cure at the TOC visit, as assessed by the investigator (based on clinical signs and symptoms, chest x-ray, CPIS and SOFA scores). Only 2 of 12 (16.7%) patients had a clinical outcome of failure at TOC. One of those two patients died due to shock and multi-organ dysfunction syndrome, 6 days after completing the full course of POL7080 treatment. There was no emergence of resistance related to POL7080 during this study.

**Conclusions**

The observed efficacy of POL7080 treatment on 28-day ACM and clinical response in this severely ill patient population underscore the potential therapeutic value of POL7080 in the treatment of VAP patients with PA infection.

## P446 Cal02: a liposomal anti-toxin therapy in infections – a new adjunctive therapeutic approach for severe community-acquired pneumonia

### B François^1^, G Colin^2^, PF Dequin^3^, PF Laterre^4^, A Perez^5^

#### ^1^Medical-Surgical ICU, Inserm CIC-1435, CHU Dupuytren, 87042, Limoges, France; ^2^Medical-Surgical ICU, District Hospital Center, La Roche-sur-Yon, France; ^3^Medical-Surgical ICU, University Hospital, Tours, France; ^4^ICU, Cliniques universitaires Saint-Luc, Université catholique de Louvain (UCL), Brussels, Belgium; ^5^Combioxin SA, Geneva, Switzerland

**Introduction**

Bacterial toxins are responsible for serious complications of infections and associated with high morbidity and mortality rates despite best antibiotic treatment. Novel empty liposomes, named CAL02 specifically mimicking domains targeted by bacterial toxins, have shown synergistic effects with antibiotics and the ability to fully rescue mice from deadly acute infections by trapping toxins. CAL02 is active against a broad panel of toxins both from Gram-positive and Gram-negative pathogens, regardless of their resistance profile. Initial results of the first-in-man trial with CAL02 in patients with severe community-acquired pneumococcal pneumonia (CAPP) are presented herein.

**Methods**

The first-in-man clinical study with CAL02 is a randomised, multicentre, double-blind, placebo-controlled, trial carried out in ICU patients with severe CAPP. All patients received standard-of-care antibiotherapy and treatment recommendations for Sepsis. The study is composed of three arms: CAL02 Low-Dose (4 mg/kg), CAL02 High-Dose (16 mg/kg), and Placebo. CAL02 is administered twice, on two consecutive days, starting within 12 hours of the diagnosis of severe CAPP, and within 24 hours of intravenous antibiotic treatment. Endpoints: safety, tolerability, efficacy and pharmacodynamic characteristics.

**Results**

The CAL02 Low-Dose/placebo cohort has been completed and the CAL02 High-Dose/placebo cohort is ongoing. To date, patients characteristics at baseline (mean (min-max)): age: 58 (38-79), CURB65 score: 3.4 (3-4), APACHE II: 20.7 (14-30), SOFA: 8.0 (6-12). 2 cases presented septic shock at screening. 5 out of 7 patients needed mechanical ventilation and 5 were treated with catecholamines due to septic shock. 6 patients achieved cured pneumonia at Test of Cure and 1 patient died on day 2. ICU stay was 11.6 days (2-22) and hospital stay 20 days (2-55). CAL02 was safe and well tolerated. Safety and efficacy data from all patients having participated to this trial will be gathered and analysed by March 2017.

**Conclusions**

This clinical study is the first trial assessing the potential of a broad-spectrum anti-virulence agent, CAL02, and its ability to protect against clinical deterioration in combination with antibiotics in a severely infected population. CAL02 is a first-in-class non-antibiotic drug which appears safe and its potential lies nonetheless far beyond, as it could also be a treatment for most infections caused by ESKAPE pathogens.

## P447 Withdrawn

## P448 Anidulafungin in ascites and pleural effusion of critically ill patients

### R Welte^1^, I Lorenz^1^, P Eller^2^, M Joannidis^1^, R Bellmann^1^

#### ^1^Medical University of Innsbruck, Innsbruck, Austria; ^2^Medical University of Graz, Graz, Austria

**Introduction**

Echinocandins such as anidulafungin are first choice for treatment of invasive candidiasis in critically ill patients. The elimination of anidulafungin is independent from renal and hepatic function. However, data on its target-site penetration are limited so far. Therefore, we assessed anidulafungin pharmacokinetics in ascites and pleural effusion of critically ill patients treated with standard doses of anidulafungin for proven or suspected invasive candidiasis.

**Methods**

Ascites and pleural effusion samples were drawn during routine paracentesis or from drains inserted for therapeutic purpose. When a drainage was in place samples were taken before as well as 1, 4, 8, 12, 18, and 24 hours after start of anidulafungin infusion. Anidulafungin quantification was performed with high pressure liquid chromatography (HPLC) and UV detection after protein precipitation with acetonitrile. For gradient elution, ammonium acetate and acetonitrile were applied. The flowrate was 1.0 ml/min. Anidulafungin was detected at 306 nm. The method was validated according to the European Medicine Agency (EMA) guidelines. Its lower limit of quantification is 0.05 mg/L.

**Results**

So far, seven critically ill patients with severe sepsis and multi-organ dysfunction were enrolled into the study. Anidulafungin pharmacokinetics in ascites has been determined in four patients. Kinetics in pleural effusion has been assessed in two patients. In addition, a single ascites sample obtained at paracentesis has been analysed. Anidulafungin peak concentrations in ascites amounted to 0.34-0.98. Thus, they were lower than the simultaneous plasma levels (peak levels 3.82-7.70 mg/L) and displayed a slower increase and decline than the plasma levels. The time to the peak level amounted to one hour in plasma and to 4-12 hours in ascites. We defined the penetration ratio as the ratio between the area under the concentration time curve (AUC) in ascites and the AUC in plasma. It amounted to 0.07-0.37. In pleural effusion, anidulafungin were slightly than those in ascites. The peak concentrations in the two patients were 1.02 and 2.02 mg/L.

**Conclusions**

Anidulafungin was detectable in ascites and pleural effusion after administration of standard doses. Ascites and pleural effusion concentrations exceeded the minimal inhibitory concentrations (MICs) reported for several Candida strains. However, isolates with MICs above the target-site concentrations have also been described. Investigation of target-site pharmacodynamics of anidulafungin in ascites and in pleural effusion is required.

## P449 Anidulafungin and the potential cost improvement with Beta-D-Glucan and Candida score associated rationalization of antifungal use

### S Lim, S Chana, S Patel

#### King’s College Hospital, London, United Kingdom

**Introduction**

Anidulafungin can be used first-line for invasive candidiasis and testing B-D-glucan (BDG) has been shown to both identify those with early invasive fungal infection and guide discontinuation of antifungal therapy when invasive candidiasis is unlikely. Despite the cost, anidulafungin may be started even in those whom invasive candidiasis is unlikely or for whom microbiological evidence is scant. We investigated the appropriateness of anidulafungin prescription and the cost implication of its use in this manner in our department

**Methods**

From April 1st - August 31st 2016, patients in whom anidulafungin was prescribed were identified and their notes analysed. Likelihood of fungal infection was assessed using the ‘Candida score’ (score < = 3 suggesting invasive candidiasis unlikely). Electronic patient records were used to determine whether serum BDG was used to confirm fungal infection, and where negative, whether this was used to discontinue anidulafungin.

**Results**

Anidulafungin was started with a score <3 on 25 occasions, and with a candida score > =3 on 13 occasions. Only 2 patients had definitive evidence of invasive candidiasis on blood cultures and both had a ‘Candida score’ > =3 (illustrating low scores identify low-risk of candidaemia). All patients with a score of > =3 in whom BDG was tested had a positive result whilst with a score <3, BDG was negative in 8 of 11 patients (where tested). Of the total anidulafungin prescriptions (374 days and 412 doses costing £123,595.88), 221 days (246 doses costing £73,797.54), were prescribed in those with low-risk of invasive candidiasis (Candida score <3), accounting for 59.7% of spend. A more rigorous strategy (Candida score <3 and negative BDG) would still save 85 days (93 doses; £27,899.07), representing 70.2% of spend in the BDG-tested population with a score <3.

**Conclusions**

Our pilot study corroborates prior data in which BDG testing identifies high-risk of invasive candidiasis, and a Candida score / BDG regimen could both stratify risk of invasive candidiasis and aid discontinuation of treatment in low-risk patients [1,2,3]. The potential cost improvement for rationalization of anidulafungin use is significant but requires further work.

**References**

León C et al. Critical Care Medicine, 34(3): 730–7, 2006

Leroy G et al. Annals of Intensive Care. 1: 50, 2011.

Hanson KE et al. PLoS One. 2012;7:e42282. doi: 10.1371/journal.pone.0042282.

**Table 9 (abstract P449).** See text for description

Candida score	BDG tested?	BDG result	Prescribed as per microbiolgy guidelines / advice	Total number	Total days of anidulafungin prescribed
<3 (n = 25)	Yes	Positive	Yes	2	12
			No	1	14
		Negative	Yes	6	58
			No	2	27
	No	N/A	Yes	9	66
			No	5	44
3 or more (n = 13)	Yes	Positive	Yes	3	78
			No	0	0
		Negative	Yes	0	0
			No	0	0
	No	N/A	Yes	8	62
			No	13	13
Total				38	374

Legend 1 :

## P450 Alterations on thyroid function test. predictors of mortality in critically ill patients?

### J Higuera, D Cabestrero, L Rey, G Narváez, A Blandino, M. Aroca, S Saéz, R De Pablo

#### Ramón y Cajal University Hospital, Madrid, Spain

**Introduction**

The aim of this study is describe and analyse the thyroid function in the critically ill patient, the different pattern of which of these are associate with higher mortality.

**Methods**

We enrolled all the patients admitted in the ICU of a tertiary hospital, during the period from January 2015 to August 2016, with a length of stay in the ICU of seven days or more.

**Results**

A total of 242 patients were included. 158 (65.3%) were male. Average age: 60. 49 ± 13.858 (20-88). Severity Indexes: SOFA 8.4 ± 4.2 (1-20); APACHE II 21.6 ± 12 (3-40); SAPS II 53 ± 18 (13-115). The mean length of stay was 20 ± 15.8 (8-113).

Regarding the thyroid functional test, TSH was recorded in 155 patients, mean values were 3.83 UI/ml ± 10.9 (0.001-126); T3 (n = 99) mean values was 1.42 pg/ml ± 0.42 (1-2.6), and T4 (n = 112) with mean values of 0.88 ng/dl ± 0.26 (0.4-2.1). The overall mortality in the study population was 22.3% (n = 54). The cause of admission was respiratory failure in 81 up to 242 patients (33.47%), Severe infection in 39 up to 242 of the cases (16.11%), Neurologic disorders in 64/242 (26.44%) and Miscellaneous 58/242 (23.9%)

We found significant differences in the severity scores of the study population related to mortality during the ICU stay (No survivors vs Survivors), SOFA (10.45 vs 7.9); APACHE II (24.42 vs 20.71); SAPS II (63.14 vs 50.69). In the patients with TSH analysis the mortality was 18.7% (n = 29) and the mean values in this group were 1.39 UI/ml. In the survival group we found mean values of 4.39 UI/ml. p = 0.182. The mean values of T3 in the mortality group were 1.15 pg/ml. In the survival group the mean of T3 were 1.5 pg/ml. (p = <0.001) IC 95%. (0.224 ± 0.487). In patients with T4 analysis the mortality was 21.42% (n = 24) and the mean values were 0.795 and in the survival group 0.91 mg/ml. p < 0.054.

After the subgroup analysis of the cause of admission, we didn’t found significate differences in the average values of hormones among the groups. However we found statistically difference in T3 average value in the group of survivors and no survivors in all the subgroups.

**Conclusions**

After the perform of the TSH, T3 and T4 analysis, in the survival, and mortality group, we found a statistical significant difference in the T3 group. We can hypothesize that the decrease of this thyroid hormone is in relation with worst outcome, as the severity scores. After the subgroup analysis of the cause of admission, we haven’t found differences in the mean values of hormones among the groups.

## P451 The role of glucagon during critical illness and its modulation by macronutrients

### S Thiessen^1^, I Vanhorebeek^1^, S Derde^1^, I Derese^1^, T Dufour^1^, C Nadège Albert^1^, L Langouche^1^, C Goossens^1^, N Peersman^1^, P Vermeersch^1^, S Vander Perre^1^, J Holst^2^, P Wouters^1^, G Van den Berghe^1^

#### ^1^University Hospital, Leuven, Belgium; ^2^Panum Institute, Copenhagen, Denmark

**Introduction**

Hyperglycemia, increased lipolysis, hypoaminoacidemia and profound muscle wasting are hallmarks of critical illness. Guidelines recommend amino acid administration to prevent muscle wasting. Glucagon, a catabolic hormone that affects these metabolic pathways and can induce muscle wasting, is increased during critical illness. Altered nutritional modulation of glucagon is known to contribute to the catabolic phenotype of diabetes. We assessed whether glucagon can be modulated by nutrition and investigated its metabolic role during critical illness.

**Methods**

In 174 critically ill patients and 20 matched healthy controls, plasma glucagon was quantified. In patients, the effect of glucose and insulin infusion and of parenteral nutrition containing amino acids (PN) on glucagon was documented. In critically ill mice with CLP-induced sepsis, the effect of amino acid infusion on plasma glucagon was evaluated and the impact of glucagon immunoneutralization on glucose, lipid and amino acid metabolism and muscle wasting was studied in the early (10 or 30 hrs) and/or prolonged (3 days) phase of critical illness.

**Results**

In patients, plasma glucagon concentrations were elevated from day 1 in ICU onwards up until day 7 and correlated with severity of illness. Infusing glucose with insulin did not significantly lower glucagon, whereas PN containing amino acids increased glucagon. In critically ill mice, infusion of amino acids increased plasma glucagon and up-regulated markers of amino acid catabolism in the liver without affecting markers of muscle wasting. Glucagon immunoneutralization in ill mice only transiently affected glucose and lipid metabolism, with a decrease in the illness-induced hyperglycemia and signs of stimulated rather than decreased lipolysis at 10 hrs. Glucagon immunoneutralization did not affect muscle wasting, but drastically suppressed markers of hepatic amino acid catabolism and reversed the illness-induced hypoaminoacidemia.

**Conclusions**

These data suggest that elevated glucagon availability during critical illness increases amino acid catabolism in the liver, explaining the illness-induced hypoaminoacidemia, without affecting muscle wasting and without a sustained impact on blood glucose. The data also suggest that infusion of amino acids, which is done during illness with the intention to spare muscle, is not effective and instead -via further increasing glucagon availability- merely results in a further breakdown of amino acids in the liver.

## P452 In vitro administration of ubiquinol and thiamine improve cellular oxygen consumption in patients with diabetic ketoacidosis

### X Liu, AU Uber, M Holmberg, V Konanki, M McNaughton, J Zhang, MW Donnino

#### Beth Israel Deaconess Medical Center, Boston, Massachusetts, United States

**Introduction**

The objective of this study was to investigate the effects of in vitro administration of ubiquinol and thiamine on cellular oxygen consumption in peripheral blood mononuclear cells (PBMCs) from patients with diabetic ketoacidosis (DKA). We hypothesized that DKA patients would have depressed cellular oxygen consumption compared to controls. DKA often requires significant hospital resources, is characterized by depletion of electrolytes, and may be associated with subclinical cellular injury with prolonged acidosis. Therefore, evaluating metabolic components (apart from insulin) that can more rapidly reverse DKA and protect cells may be beneficial. Thiamine and ubiquinol are essential for adequate aerobic metabolism.

**Methods**

We performed a prospective study of DKA patients and healthy controls presenting to the emergency department at an urban tertiary care center from November 2015 to June 2016. A single blood draw was performed and PMBCs were isolated from blood samples. Cells were randomly assigned to in vitro administration of 0.5 ug/mL thiamine, 1 ìg/mL ubiquinol, or placebo treatment. The complete mitochondrial respiration profiles were measured using XF Cell Stress Mito Kit (Seahorse Bioscience) to reveal the key parameters of cellular oxygen consumption. One-way ANOVA was used to analyze differences in oxygen consumption rate between groups.

**Results**

A total of 10 DKA patients and 9 controls were included. Basal (7.0 ± 2.1 pmol/min/μg protein vs. 10.2 ± 2.4, p = 0.005) and maximal oxygen consumption (16.6 ± 4.2 vs 28.3 ± 9.0, p = 0.05) were significantly lower in PBMCs in DKA compared to controls. We found a significant increase in basal (10.3 ± 2.4 pmol/min/μg protein vs. 7.0 ± 2.1, p = 0.05) and maximal (27.6 ± 5.2 vs. 16.6 ± 4.2, p = 0.04) oxygen consumption between the ubiquinol and placebo group. Additionally, we found a significant increase in basal (9.3 ± 2.4 pmol/min/μg protein vs. 7.0 ± 2.1, p = 0.05) and maximal (25.4 ± 5.0 vs. 16.6 ± 4.2, p = 0.05) oxygen consumption between the thiamine and placebo group. Neither ubiquinol nor thiamine had a significant effect on basal and maximal oxygen consumption for controls.

**Conclusions**

DKA patients had overall lower oxygen consumption compared to healthy controls. In vitro administration of thiamine and ubiquinol independently increased oxygen consumption in DKA patients, but not in controls. These findings suggest thiamine and ubiquinol may have potential as mitochondrial resuscitators in DKA and potentially states with similar metabolic stress.

## P453 The evaluation of the adherence to and outcome of the blood glucose monitoring protocol

### O Demirkiran, A Byelyalov

#### Istanbul University Cerrahpasa Medical School, Istanbul, Turkey

**Introduction**

This study aims to evaluate the adherence to and outcome of the blood glucose monitoring protocol amongst adult patients in ICU.

**Methods**

Patients above 18 years of age, who were admitted to EICU between March 2012 and March 2015 for longer than 24 hours and put on blood glucose monitoring protocol, were enrolled in this study. Patients’ demographic data, diagnosis on admission, presence of diabetes mellitus diagnosis, APACHE II score, length of stay in ICU and afterwards in the wards and health state on discharge from ICU and from the wards were recorded retrospectively. We calculated the rates of adherence to the timing of measurements and treatment protocol using patients’ blood glucose recordings, the success rate of glycemic control using blood glucose levels, and the effect of adherence to monitoring protocol on the mortality rates and length of hospital stay. We assessed if the disparity in protocol adherence is associated with diabetes mellitus diagnosis, having surgery, severe sepsis/septic shock diagnosis or different APACHE II scores. Total 453 patients in 4447 in ICU stay day 22.220 measurement were recorded.

**Results**

24% of measurement timings and 57.8% of treatments were according to the protocol. Better adherence achieved more effective hyperglycemia control and lessened the fluctuations in blood glucose levels, however it caused significantly increased rates of hypoglycemia and no change in mortality or length of hospital stay. The adherence rate was lower for patients with diabetes mellitus diagnosis, but sepsis or surgery had no effect.

**Conclusions**

We need to update our protocol according to the current guidelines in order to improve its efficacy and safety, redesign the parts that may hinder our adherence, and provide the necessary training to the staff for improving the adherence to the protocol.

**References**

48 Jacobi J, Bircher N, Krinsley JS, et al. Guidelines for the use of an insulin infusion for the management of hyperglycemia in critically ill patients. Crit Care Med 2012;40:3251–76

**Fig. 24 (abstract P453).**
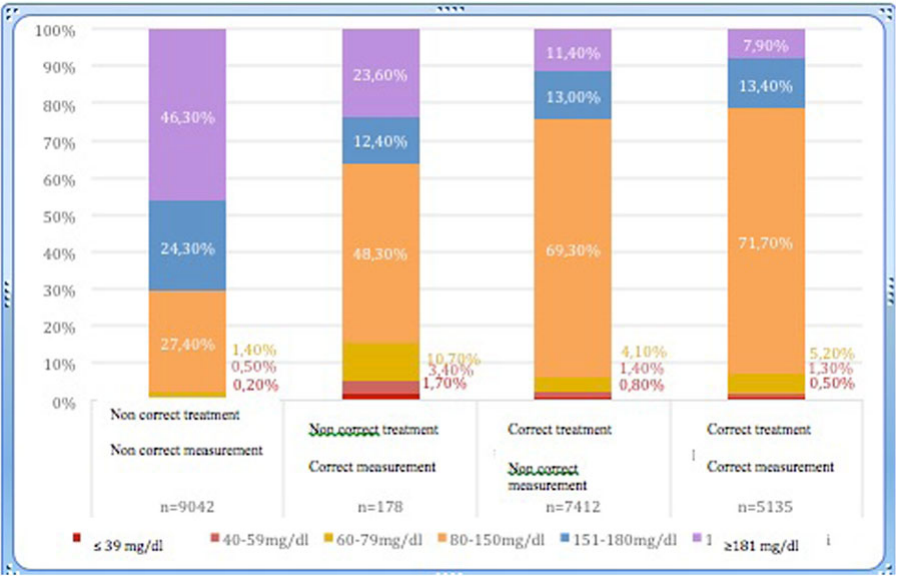
See text for description

## P454 Insulin receptor b isoforms expression: a new insulin-resistance mechanism in critically ill patients?

### C Luengo^1^, J Guerrero^2^, M Cariqueo^1^

#### ^1^Hospital Clínico Universidad de Chile, Santiago, Chile; ^2^Universidad de Chile, Santiago, Chile

**Introduction**

Two insulin receptor (IR) isoforms are responsible for insulin physiological actions. IR primary mRNA after alternative splicing generates IR-B and IR-A isoforms [1-3]. They activate different signalization pathways leading to different physiological effects; IR-B is responsible of metabolic effects. In Metabolic Syndrome, insulin resistance is associated to changes in IR isoforms ratio expression [4]. There could exist a similar situation in sepsis, but there is not data yet. The aim of this study was to assess the effects of serum septic shock on the IR-B mRNA expression.

**Methods**

RAW cells, a murine cell line, were cultured in conditioned media – enriched with 10% of human septic serum (HSS) - or usual culture media (control). HSS was obtained from critical care patients with less than 24 hours of septic shock diagnosis. Blood samples were centrifuged and the serum was obtained. Cells were culture by 16h, mRNA was extracted and quantified, cDNA was obtained by reverse transcription and using specifics primers, IR-B mRNA was amplified [5]. The cellular mRNA content was obtained by densitometric analysis. This same method was uses to assess if LPS (25 ng/mL) could have a similar effect to HSS. For statics analysis we used non-parametric test and p < 0.05 was considered significant.

**Results**

**Fig. 25 (abstract P454).**
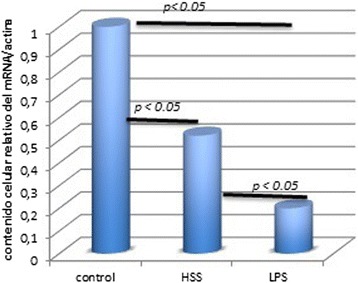
Results: IR-B mRNA cellular content

Both HSS and LPS significantly decreased IR-B mRNA content compared to control. The effect of LPS was significantly higher than observed with HSS to decrease the IR-B mRNA content (Fig. 25).

**Conclusions**

We demonstrated that HSS induces a significant reduction of IR-B expression in macrophages; the infective agent could be mediated this effect. The LPS concentration used here was greater than reported in clinics and it could explain it greater effect, if the lower content of mRNA implies decrease of the protein IR-B expression, then we are in presence of a new pathogenic mechanism for hypoglycemia in sepsis not reported before.

**References**

[1] Zauner A. Metabolism 56(1):1–5, 2007

[2] Tanti JF. Curr Opin Pharmacol 9(6):753–62, 2009

[3] Marik PE. Critical Care 17:305, 2013

[4] Besic V. PLoS ONE 10: e0119270, 2015

[5] Guerrero J. Crit Care 17: R107, 2013

## P455 Association between glycemic variability and nosocomial infection susceptibility in critically ill patients

### C Scorcella, R Domizi, E Damiani, S Tondi, S Pierantozzi, N Rossini, U Falanga, V Monaldi, E Adrario, P Pelaia, A Donati

#### Università Politecnica delle Marche, Ancona, Italy

**Introduction**

Retrospective studies showed that high blood glucose variability is associated with an increased onset of infections and mortality in critically ill patients. However, a causal link has not been established. The aim of this study is to evaluate this association in a prospectively observed large-scale Intensive Care (IC) population.

**Methods**

Multi-center prospective observational study, involving 11 Italian Intensive Care Units (ICUs). All the consecutively admitted adult patients with an expected ICU-length of stay > = 24 hours were included. Recruitment phase began in February 2016 (sample size 3300 patients). All the routinely performed blood glucose measurements are recorded. Glycemic variability is assessed by calculating the daily glycemic lability index (GLI), standard deviation (SD) and coefficient of variation (CV). Demographic, clinical, microbiological and laboratory data are being collected daily from the ICU admission to the discharge/death.

**Results**

These are the preliminary data from the first 57 patients admitted to the ICU of “Ospedali Riuniti” di Ancona (Italy). ICU Non-Survivors (n = 19) showed higher mean GLI (11.6 [6.5-26.2] versus 4.6 [2.7-13.7], p = 0.013), SD (1 [0.9-1.5] versus 0.8 [0.6-1], p = 0.009) and CV (0.14 [0.12-0.19] versus 0.12 [0.09-0.15], p = 0.024) as compared to Survivors. The mean glycaemia was similar. Mean GLI, SD and CV were able to discriminate for mortality (area under the receiver operating characteristic curve: 0.71 [95% confidence interval 0.57-0.82], 0.72 [0.58-0.83] and 0.69 [0.55-0.80], respectively) but not for infections onset. However, glycemic variability tended to be higher in patients who developed ICU-acquired infections.

**Conclusions**

These preliminary data confirmed the association between glycemic variability and mortality in critically ill patients. A higher blood glucose variability may be associated with a higher susceptibility to nosocomial infections in the ICU population but data from a larger sample of patients are needed.

**Fig. 26 (abstract P455).**
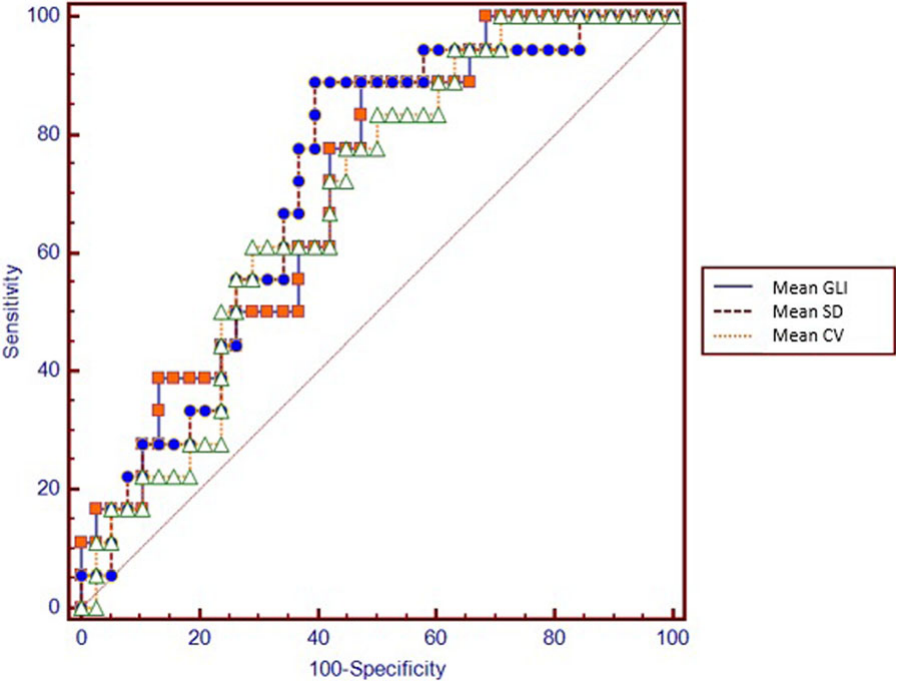
Glycemic variability indexes and mortality

## P456 Glysure versus standard blood glucose monitoring device in patients undergoing cardiac surgery

### O Cole^1^, N Scawn^2^, M Balciunas^1^, I Blascovics^1^, A Vuylsteke^1^, K Salaunkey^1^

#### ^1^Papworth Hospital, Cambridge, United Kingdom; ^2^Liverpool Heart and Chest Hospital, Liverpool, United Kingdom

**Introduction**

Meticulous euglycemia is known to improve outcomes in critically ill patients [1].

However attempting to maintain a tight glucose control regime does lead to dangerous life threatening hypoglycemic episodes [2].

Continous blood glucose monitoring (CGM) can potentially improve euglycemic control by providing immediate feedback to insulin’s effects and probably reduce the workload of the nurses.

**Methods**

After obtaining R&D approvals and patient consent, patients undergoing elective cardiac surgery in 2 centres were enrolled. A non-randomised, non-treatment, open label prospective study was performed where blood glucose results from Glysure’s CGM was compared with the conventional blood gas analyser (Siemens RAPIDLab 1200 Systems).

**Results**

27 patients were monitored for an average duration of 19.9 hrs, 161 samples (average of 6/patient) were obtained. Mean absolute relative deviation (MARD) was 10.34%. The correlation coefficient (r) between the GlySure and intermittent measurement was 0.737.

**Conclusions**

Glysure based CGM is

1.Reliable.

2.Records Glycemic trends accurately.

3.Reduces workload of nurses.

**References**

1. Van den Berghe G, et al. Intensive insulin therapy in critically ill patients. NEJM. 2001 Nov 8;345(19):1359–67.

2. Investigators TN-SS. Intensive versus Conventional Glucose Control in Critically Ill Patients. NEJM. 2009 Mar 26;360(13):1283–97.

**Fig. 27 (abstract P456).**
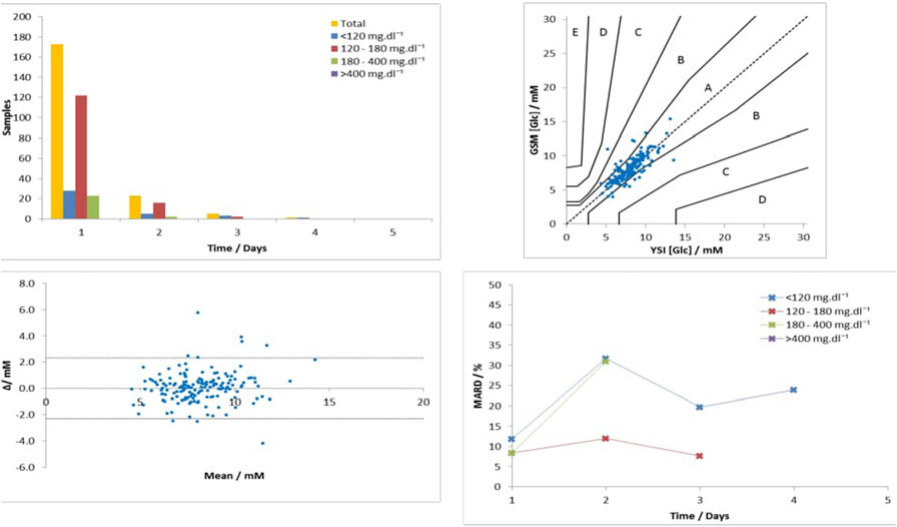
Comparison between Glysure and conventional method

## P457 Relationship between glycated hemoglobin and poor glucose control in intensive care cardiac surgery settings

### A Omar, A Salama, M Allam, A Alkhulaifi

#### Hamad medical corporation, Doha, Qatar

**Introduction**

Measuring of hemoglobin A1c (HbA1c) remain the gold standard for chronic glycemia monitoring in diabetics [1]. High HbA1c following coronary artery bypass graft could signal higher short- and long- term mortality rates [2]. We aim to find relation between preoperative HbA1C and glucose control in intensive care expressed through time in range blood glucose (TIR).

**Methods**

Two hundred twenty seven patients were involved in this single center prospective observational study. All patients were assessed by HbA1C before surgery, the glucose target was set to be 6.0 to 8.1 mmol/L. Glucose control was expressed though TIR, and based on this patients were grouped into group I who remained within the set target > 80% of the time and group II who remained < 80% within the target. Both groups were matched in terms of age, gender, race, Euro score, cardiopulmonary bypass time (CPB), aortic cross clamp time (ACC). Outcome variables were compared in diabetics and non-diabetics.

**Results**

Failure to maintain target glycemia was significantly more frequent in diabetics (p = 0.001) in patients with glycated hemoglobin (HbA1c) > 8%. Group II had significantly higher HbA1C (8 ± 2.2 vs 6.6 ± 1.7, p = 0.001), this group was significantly more liable to complications in terms of wound infection and post operative atrial fibrillation (p = 0.05 and 0.04 respectively). Lengths of ventilation, stays within ICU and hospital stay were significantly higher in group II (p = 0.03, 0.04, 0.3 respectively).

**Conclusions**

High HbA1C is likely a good predictor of poor glycemic control and post-operative adverse events.

**References**

1- Makris K, Spanou L. Is there a relationship between mean blood glucose and glycated hemoglobin? Journal of diabetes science and technology. 2011 Nov 1;5(6):1572–83.

2- Halkos ME, Lattouf OM, Puskas JD, Kilgo P, Cooper WA, Morris CD, et al. Elevated preoperative hemoglobin A1c level is associated with reduced long-term survival after coronary artery bypass surgery. Ann Thorac Surg. 2008;86(5):1431–7.

## P458 Leukocyte telomere length in critically ill children: association with outcome and impact of the nutritional management

### S Verstraete^1^, I Vanhorebeek^1^, E Van Puffelen^2^, I Derese^1^, C Ingels^1^, S Verbruggen^2^, P Wouters^1^, K Joosten^2^, J Hanot^1^, G Guerra^3^, D Vlasselaers^1^, J Lin^4^, G Van den Berghe^1^

#### ^1^KU Leuven, Leuven, Belgium; ^2^Erasmus Medical Centre, Rotterdam, Netherlands; ^3^University of Alberta, Edmonton, Canada; ^4^University of California, San Francisco, San Francisco, United States

**Introduction**

Shorter leukocyte telomeres have been associated with chronic diseases. We investigated whether telomere lengths upon admission to the paediatric intensive care unit (PICU) predict outcome and whether telomere length is altered during PICU stay in a different manner for children who receive or do not receive early parenteral nutrition (PN).

**Methods**

Telomere lengths were quantified in leukocyte DNA harvested from 342 healthy children and from 1148 critically ill children who were randomised to early or late parenteral nutrition from admission to the last day in PICU. Independent associations of telomere lengths with outcomes, with PICU mortality as primary outcome, and the impact of early PN on the change in telomere length during PICU stay were studied via multivariable logistic regression and Cox-proportional hazard analyses.

**Results**

Children admitted to PICU revealed shorter leukocyte telomeres than matched healthy children. A shorter, not longer, admission leukocyte telomere length appeared independently associated with better PICU survival (P = 0.01), a finding that was statistically fully explained by a more activated innate immune response (P = 0.04) with a higher fraction of neutrophils that have shorter telomeres. After adjustment for confounders, including neutrophil count, early PN shortened leukocyte telomere length as compared with late PN (estimate early versus late -0.02, 95% CI [-0.04;-0.004], P = 0.01), an effect that was only partially explained by its impact on the duration of PICU stay (estimate per day in PICU -0.14, 95% CI [-0.28;-0.004], P = 0.04).

**Conclusions**

Shorter leukocyte telomeres in PICU patients than in healthy children reflected the degree of innate immune response activation which independently predicted better survival. Leukocyte telomeres were shortened by early PN during paediatric critical illness. Whether shortened telomere lengths predispose PICU patients to the long-term legacy of critical illness remains to be investigated.

## P459 Assessment of skeletal muscle wasting in critically ill trauma patients using serial computed tomography imaging

### R Haines^1^, P Zolfaghari^1^, R Hewson^1^, C Offiah^1^, J Prowle^2^

#### ^1^Barts Health NHS Trust, London, United Kingdom; ^2^Queen Mary University, London, United Kingdom

**Introduction**

Muscle wasting is very common in critically ill patients and can have a profound effect on long term recovery. We aimed to quantify the extent of muscle wasting in trauma patients in the ICU. CT imaging has emerged as a method to assess body composition, with good correlation to total skeletal muscle volume [1].

**Methods**

**Fig. 28 (abstract P459).**
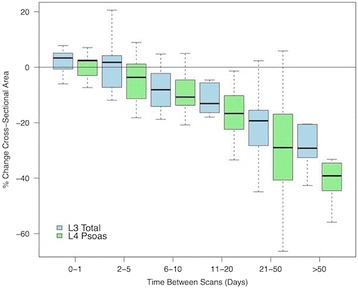
See text for description

Of 858 trauma patients admitted to ICU over a 30-month period (Jan 2012-14), 81 were identified as having had 2 abdominal CT scans. The initial trauma CT scan was compared to the subsequent scan. Two methods were used to measure muscle cross-sectional area (CSA). In the first, abdominal muscle CSA at L3 vertebrae was measured using Fig. 28 J software by tracing the abdominal musculature and applying predefined Hounsfield Units, -29 to +150 for skeletal muscle. An observer traced each psoas muscle at the L4 basivertebral vein for the second method.

**Results**

Of the 81 patients included 83% were male, median age was 39 (IQR: 23.5-53), median admission APACHE-2 was 12 (9-16) and median new injury severity score was 38 (27-50). When comparing CT scans on admission to inpatient scans performed at least 5 days later (median days between scans was 14.5, IQR: 7-28), there was an overall decrease in psoas muscle of 15.7% (IQR: 8.9-29.0) and L3 CSA of 14.3% (6.5-20.5). There was increasing loss in muscle volume for both L3 CSA and psoas with increasing time between CT scans (Fig. 28).

**Conclusions**

Skeletal muscle wasting is common and time-dependent in critically ill trauma patients.

**References**

1. Shen, W., et al., J Appl Physiol (1985), 2004. 97(6): p. 2333–8.

## P460 Predictive value of sarcopenia on outcome of sepsis in the emergency department

### H Park, B Ko

#### Kyung Hee University Hospital at Gangdong, Kyung Hee University School of Medicine, Seoul, South Korea

**Introduction**

Sarcopenia (skeletal muscle depletion) has been suggested to predict morbidity, especially infection and mortality, in various diseases. However, it is not well known whether sarcopenia has prognostic value for predicting poor outcome in sepsis. We hypothesized that sarcopenia can predict outcome in sepsis in the emergency department (ED) assessed by abdominopelvic computed tomography (CT).

**Methods**

We retrospectively reviewed the medical records of 627 patients with sepsis in the ED of a university-affiliated hospital during a 10-year period. We divided the patients into two groups according to 28-day survival and compared demographic characteristics, clinical features and presence of sarcopenia assessed by cross-sectional area of the psoas muscle at the level of the third lumbar vertebra on abdominal CT scans. Multivariate logistic regression analysis was conducted to examine the independent prognostic value of scarcopenia on the outcome of sepsis.

**Results**

A total of 274 patients with sepsis were finally included in the study: 45 (16.4%) did not survive on 28 days since ED presentation, and 77 patients (28.1%) were identified as having sarcopenia. The presence of sarcopenia could independently predict 28-day mortality on multivariate logistic analysis (OR, 2.382; 95% CI, 1.155-4.913, P = 0.019). Sarcopenia could also predict 6 month mortality (OR, 2.599; 95% CI, 1.345-5.020, P = 0.004).

**Conclusions**

Sarcopenia evaluated by CT scan can predict poor outcome of sepsis patients. Further large prospective studies on sarcopenia in sepsis and the efficacy of nutrition therapy are warranted.

**References**

1. Cosqueric G, Sebag A, Ducolombier C, Thomas C, Piette F, Weill-Engerer S. Sarcopenia is predictive of nosocomial infection in care of the elderly. Br J Nutr 2006; 96: 895–901.

2. Lieffers JR, Bathe OF, Fassbender K, Winget M, Baracos VE. Sarcopenia is associated with postoperative infection and delayed recovery from colorectal cancer resection surgery. Br J Cancer 2012; 107: 931–936.

## P461 Withdrawn

## P462 Comparison between SNAQ and phase angle to assess malnutrition at time of ICU admission

### H Buter, JA Veenstra, M Koopmans, EC Boerma

#### MCL, Leeuwarden, Netherlands

**Introduction**

Malnutrition at ICU admission is associated with increased morbidity and mortality. Prevalence of malnutrition in ICU patients varies from 17 to 78 percent in different patients groups.

Malnutrition can be assessed by questionnaires but also by bio-impedance analysis. We compared the Short Nutritional Assessment Questionnaire (SNAQ) with phase angle at ICU admission.

**Methods**

During 15 weeks consecutive patients were included. Exclusion criteria were age < 18 years, abnormalities of the limbs and ICU stay < 6 hours. Phase angle was measured short after admission, SNAQ was obtained from the patient or legal representative. Malnutrition is diagnosed by SNAQ ¡Ý 2 or phase angle of < 5¡ã for men and < 4.6¡ã for women. Demographic and clinical variables were collected. The study was approved by our local ethical committee (MCL, nWMO 77, 2015).

**Results**

**Fig. 29 (abstract P462).**
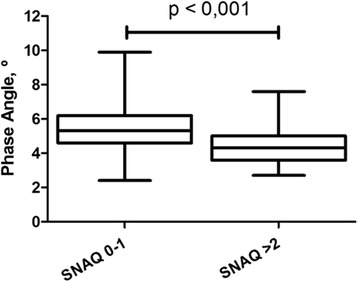
Boxplot: SNAQ and phase angle

We included 299 patients (66% male, age 66 ¡À 12 yr, BMI 27 ¡À 4 kg/m2, APACHE II 14 [11-17], elective 193 and acute 106). Hospital mortality was 7.4%. Malnutrition was present in 16% according to SNAQ and in 34% according to phase angle. There was a fair accordance between SNAQ and phase angle (Table 10). Phase angle was significantly higher in patients with SNAQ 0-1 (5.5¡ã¡À1.3) in comparison to SNAQ ¡Ý 2 (4.4¡ã¡À1.1) (Fig. 29).

**Conclusions**

Malnutrition was present in 16% according to SNAQ and 36% according to phase angle. There was a fair agreement between SNAQ and phase angle. With the use of phase angle an objective and accurate assessment of nutritional status can be made in ICU patients.

**Table 10 (abstract P462).** See text for description

	SNAQ 0-1	SNAQ > =2
Phase angle normal	180	13
Phase angle low	72	34

Legend 1: Two by Two: SNAQ and phase angle

## P463 Phase angle is an independent predictor of mortality in ICU patients

### JA Veenstra, H Buter, M Koopmans, EC Boerma

#### MCL, Leeuwarden, Netherlands

**Introduction**

Malnutrition is an important determinant of ICU outcome. An objective way to measure body composition and malnutrition is by bio-impedance analysis and calculation of phase angle (PhA). We questioned whether malnutrition, according to PhA, is associated with morbidity and mortality after ICU admission.

**Methods**

During 15 weeks consecutive patients were included. Exclusion criteria were age < 18 years, abnormalities of the limbs and ICU stay < 6 hours. PhA was measured short after admission, a PhA < 5 ° for men and < 4.6° for women was considered malnutrition. Demographic and clinical variables were collected. The study was approved by our ethical committee (nWMO 77, 2015).

**Results**

299 Patients were included. Malnutrition was established in 36% (n = 106). Patients with malnutrition were more often female, older, with lower BMI and higher APACHE II score (Table 11) and more often diagnosed with a malignancy (30 vs 14%, p < 0.001). Hospital LOS, ICU and hospital mortality were higher in patients with a low PhA.

Logistic regression analysis showed a significant relation between PhA and age, sex, BMI, malignant disease, hospital LOS and hospital mortality.

**Conclusions**

According to PhA, malnutrition was present in 36% of ICU patients. The same independent predictors of PhA (age, sex and BMI) were found as in a general hospital population. Even in this small population of a mixed ICU patients, a low phase angle was found to independently predict hospital mortality. The value of phase angle for ICU patients warrants more research.

**Table 11 (abstract P463):** See text for description

	PhA normal n = 193	PhA low n = 106	P
Male n	137	61	0.019
Age yrs	63 ± 11	71 ± 10	<0.001
BMI	27 ± 4	26 ± 4	0.034
APACHE II	13 [10-17]	15 [12-19	<0.001
LOS ICU days	0.9 [0.8-1.3]	0.9 [0.8-3]	0.234
LOS Hosp days	9 [5-16]	16 [9-25]	<0.001
Mortality ICU %	3.1	9.4	0.02
Mortality Hosp %	4.1	13.2	0.004

Legend 1: Patients characteristics

## P464 Assessment of sarcopenia by serial ultrasound measurements of quadreceps femoris cross-sectional area in patient with ischemic cardiomyopathy

### A Taha, A Shafie, S Hallaj, D Gharaibeh, H Hon

#### Sheikh Khalifa Medical City, Abu Dhabi, United Arab Emirates

**Introduction**

Ultrasound is a noninvasive method that can measure quadriceps muscle layer thickness (QMLT) and subsequently lean body mass (LBM) at the bedside,with the validity and reliability checked and confirmed in previous studies, we correlated the sarcopenia associated with heart failure in ischemic cardiomyopathy patients detected by decrease cross sectional area of Qudreceps femoris with the clinical and labortatory parameters of sarcopenia.

**Methods**

Retrospective observational study including 26 patients with ischemic cardiomyopathy admitted to our cardiac ICU were enrolled between January 2014 and september 2016 .serial measurements of quadriceps muscle cross sectional area were obtained at admission and every 3 days for intial 10 days. Measures was recorded to assess the trend and correlation between presence of muscle loss and the clinical and laboratory parameters that indicate the presence of sarcopenia despite effective feeding protocol, all enrolled patients were hemodynamically monitored and treated under the same ICU feeding and physiotherapy protocol.

**Results**

Completed data sets were obtained from 26 patients, 20 males and 6 females, mean age of 57 with age range from 43 to 90 years. The following parameters were assessed daily: Body weight, serum albumin, haemoglobin and CRP, Quadricips femoris for a total of 260 scans was analyzed and serial measurements compared, it shows statistical significant difference between recorded measures (P < 0.05) and positive correlation with clinical assessment.

**Conclusions**

In our clinical series, Assessment of sarcopenia by serial Ultrasound measurements of Quadreceps femoris cross-sectional area in patient with ischemic cardiomyopathy was correlated with clinical and laboratory assessment of sarcopenia, its an easy and reliable technique to assess for nutritional status and to evaluate our feeding protocol, Further studies needed to confirm these findings.

**References**

Tillquist M, Kutsogiannis DJ, Wischmeyer PE, Kummerlen C, Leung R, Stollery D, Karvellas CJ, Preiser JC, Bird N, Kozar R, Heyland DK. Bedside ultrasound is a practical and reliable measurement tool for assessing quadriceps muscle layer thickness. JPEN J Parenter Enteral Nutr. 2014 Sep;38(7):886–90.

## P465 Prevalence and prognosis impact of malnutrition in elderly of a Moroccan acute medical unit

### M Bizrane, A A. El Khattate, N Madani, R Abouqal, J Belayachi

#### Ibn sina university hospital, Rabat, Morocco

**Introduction**

Our study aims mainly to identify the prevalence of malnutrition, to compare elderly patients according to their nutritional status in a Moroccan AMU; then analyze the prognosis impact of malnutrition at 540 days follow-up.

**Methods**

This was a prospective cohort study conducted in the AMU; Ibn Sina University hospital, Rabat; Morocco, from June to September 2014, including patients aged of > =65 years. Demographic, anthropometric, comorbid diseases, clinical characteristics and in-hospital evolution data were included. Survival status was evaluated at 2 times; in the hospital and at 540 days follow-up. Malnutrition was evaluated using the MNA, and the health and functional status using EuroQol-5D-3L. Cox proportional hazard univariate analysis identified the factors associated with mortality at 540 days. Then, multivariable model incorporating 4 conventional risk variables was calculated with Cox proportional hazards regression analysis, and we assessed the predictive performance of this model. Statistical analyses were carried out in SPSS Statistics and STATA.

**Results**

Ninety five patients were included. Mean age was 75 ± 5.9 years; 64.2% were women. The mean ± SD length of stay in AMU was 6.2 ± 5 days. In-hospital and 540 days post-discharge follow-up mortality were respectively 16.8% and 46.3%.Median [IQR] of EQ5D-Index before acute illness was 0.29 [-0.17;0.81]. Prevalence of well-nourished, at risk of malnutrition and malnourished patients were respectively 46.3%, 27.4%, and 26.3%. Compared to well-nourished patients, patients at risk of malnutrition and malnourished patients had significantly higher mortality, with respectively (HR:2.5,1.1;5.7, p = 0.02) and (HR:5.5,2.4;12.1, p < 0.001). Cox proportional hazards multivariate analyses incorporating conventional risk variables without, and with nutritional status, showed that malnutrition was a predictor of 540 days mortality: unmarried patient (HR:2.1,1.1;4.1, p = 0.02) ; Lower Mean arterial pressure (HR:0.97,0.94;0.99, p = 0.02); Lower EQ5D index (HR:0.5,0.3;0.9, p = 0.04), malnourished patients (HR:3,1.2;7.7, p = 0.01). In addition, for increasing probability thresholds, the model incorporating nutritional status combined had higher net benefit than the conventional model, suggesting that this model has potentially higher clinical utility.

**Conclusions**

Malnutrition has a negative prognosis impact on the long-term survival status of elderly in an AMU. Therefore, its high clinical utility should be considered to guide nutrition interventions during hospital stay and even after discharge to reduce the poor outcome of elderly patients.

## P466 Achievement of 60% targeted cumulative calories in the first week of ARDS reduces 28- day mortality

### N Kongpolprom, N Sanguanwong

#### Chulalongkorn University, Bangkok, Thailand

**Introduction**

There are limited data of mortality-benefit from nutritional management in ARDS patients. This study aimed to evaluate the impact of the amount of calorie-intake during the first week of ARDS on 28-day mortality.

**Methods**

We retrospectively collected data from ARDS patients admitted in medical ICUs in our hospital between 2010 and 2014 and divided into 3 groups, namely patients receiving less than 60% of targeted cumulative calories in the first week of ARDS, those receiving between 60% and 100% of the 7-day targeted calories and those receiving at least 100% of the 7-day targeted calories. The 28-day mortalities among 3 groups were analyzed and compared.

**Results**

Totally, 114 ARDS patients were included. Baseline characteristics and clinical outcome were shown in Table 12. The 28-day mortality was significantly higher in group1, compared with groups 2 and 3 with odds ratio (OR) of 4.96 (95% CI: 1.57 to 15.65), p = 0.006 and 3.86 (95% CI: 1.53 to 9.74), p = 0.004, respectively. However, the 28-day mortalities in groups 2 and 3 were not different; OR of 0.78 (95% CI: 0.23 to 2.63), p = 0.69.

**Conclusions**

We recommended to provide at least 60% of targeted cumulative calories in the first week of ARDS to improve 28-day mortality. However, due to limited sample size, further studies are needed to confirm the results.

**Table 12 (abstract P466).** See text for description

	Group 1 < 60% of 7 day targeted calories n = 68	Group 2 60-99% of 7 day targeted calories n = 16	Group 3 ≥ 100% of 7 day targeted calories n = 30
Age mean(SD) years	54.09(17.85)	44.50(13.17)	57.30(21.42)
Sex male n (%)	43(63.2)	5(31.3)	13(43.3)
APACHE II median [IQR]	26.50[9]	24.00[7.5]	25.00[8.75]
PF ratio median [IQR]	129.40[92.25]	109.98[69.06]	107.81[72.41]
Tidal volume day1 median [IQR] ml/KgIBW	8.36[3.18]	8.99[3.04]	8.05[4.7]
PEEP day1 median [IQR] cmH2O	10.00[7]	10.00[10]	10.00[6]
7 day cumulative calories mean(SD) Kcal.	2545.96(2336.11)	7171.25(889.28)	9595.73(1888.90)
28-day mortality (%) * = p <0.05 compared with gr.1	79.4	43.8 *	50 *

Legend : Baseline characteristics and clinical outcome of ARDS patients

## P467 Effect of gastric residual volume monitoring on incidence of ventilator associated pneumonia in mechanically ventilated patients admitted to intensive care unit

### S Sanaie, A Mahmoodpoor, H Hamishehkar

#### Tabriz University of Medical Sciences, Tabriz, Iran

**Introduction**

Frequency of motility disorders in the upper or lower gastrointestinal (GI) tract is reported to be almost 80% in critically ill patients, which a delayed passage and delayed gastric emptying are very common. Impaired gastric motility can lead to regurgitation or vomiting of gastric contents which may cause aspiration and consequently pneumonia. Enteral nutrition is the preferred route of nutrient supply in critically ill patients who are not able to consume oral foods. Monitoring of upper GI function has an important role in the decision of the continuation of enteral nutrition. Measurement of gastric residual volume (GRV) is recommended for monitoring of GI function and avoidance of aspiration and pneumonia. However, there are some studies which have not shown any relationship between high GRV and risk of aspiration and pneumonia in critically ill patients. So, validity of GRV as a marker for risk of aspiration in mechanically ventilated patients is still unclear. The aim of this study was to assess the effect of GRV monitoring on incidence of ventilator associated pneumoniain (VAP) in mechanically ventilated patients admitted to intensive care unit.

**Methods**

This descriptive study was done on 150 mechanically ventilated adult patients admitted to intensive care unit and receiving enteral nutrition. GRV was measured every 3 hours and gastric intolerance was defined as GRV > 250 cc. The incidence of vomiting and VAP, GRV, length of mechanical ventilation and ICU stay, APACHE and SOFA scores, and mortality rate were noted. Data were analyzed using SPSS 18.

**Results**

The mean age of the patients was 57.79 ± 18.84 years. The incidence of VAP was 24% (36 patients). Incidence of vomiting was 50% [24 patients in VAP positive group and 51 patients in VAP negative group (P = 0.022)]. Mean ICU length of stay (LOS) was 11.59 ± 4.91 days and mechanical ventilation was 7.54 ± 3.67 days. Mean APACHE score was 25.14 ± 5.78 and mean SOFA score was 11.51 ± 2.14. Mean GRV during the ICU stay was 230.51 ± 52.63. Mortality rate was 22.1%. There was not a relationship between GRV and ICU LOS (P = 0.741), and incidence of VAP (P = 0.329). But, there was a positive relation between GRV and incidence of vomiting (P < 0.001) and mortality rate (P = 0.001).

**Conclusions**

This study showed that increased GRV is associated with increased incidence of vomiting and increased mortality rate; but it is not associated with VAP incidence in critically ill patients.

## P468 The efficacy of a novel peristaltic feeding tube (PFT) in reducing reflux and aspiration of gastric contents in mechanically-ventilated patients

### P Biderman^1^, P Van Heerden^2^, Y Avitzur^3^, S Solomon^4^, Z Iakobishvili^1^, U Carmi^1^, D Gorfil^1^, P Singer^1^

#### ^1^Rabin Medical Center, Petah Tikvah, Israel; ^2^Hadassah University Hospital, Jerusalem, Israel; ^3^Hospital for Sick Kids, Toronto, Canada; ^4^Lunguard Pty Ltd, Yavne, Israel

**Introduction**

**Fig. 30 (abstract P468).**
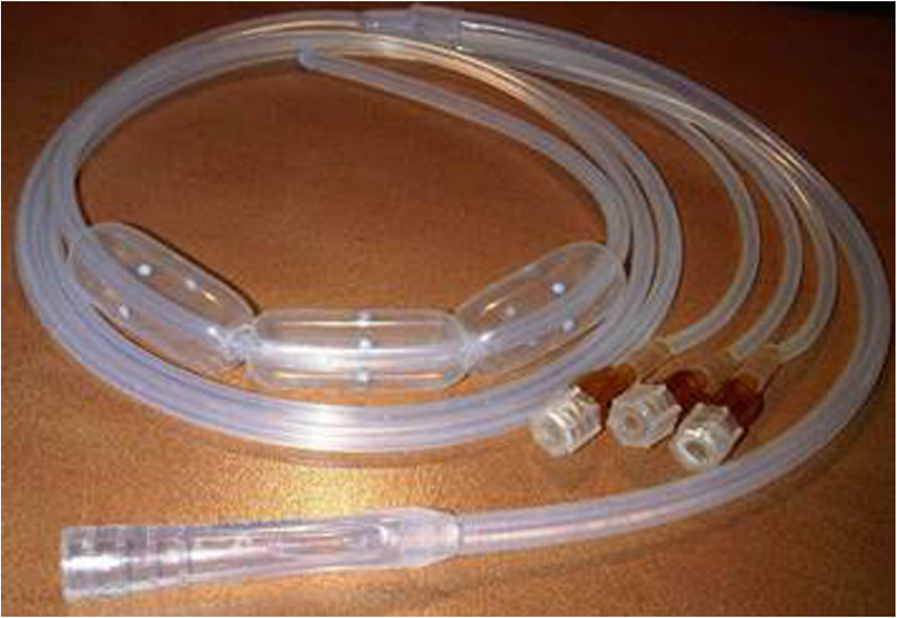
Peristaltic feeding tube

Gastro-esophageal reflux (GER) is common in ventilated patients and is a cause of VAP. We recently described the development of a unique device called the peristaltic feeding tube (PFT) (Lunguard, Yavne, Israel) [1]. The PFT (Fig. 30) uses simulated peristalsis to seal the esophagus to fluid moving retrogradely, whilst allowing normal drainage of fluid and secretions moving ante-gradely. Here we describe the first trial of the PFT in ventilated patients.

**Methods**

There were 10 subjects in the treatment (PFT) group and 10 patients in the control group, who had all undergone elective cardiac surgery and were ventilated in the ICU afterwards. The PFT was placed on admission to the ICU. In the control group a standard nasogastric tube (NGT) was inserted. Specimens were collected by suctioning from the oropharynx and from above the tracheal tube balloon every hour and from the trachea twice per eight hour shift. Samples were analyzed by ELISA for Pepsin A as a marker for secretions of gastric origin.

**Results**

The two groups were comparable (demographics and duration of ventilation). There were statistically more specimens positive for Pepsin A in the control group in the oropharynx and above the ETT cuff, but not in the trachea (Table 13).

**Conclusions**

The PFT reduced the amount of GER in ventilated patients. A larger study is required to determine whether this translates to a reduction in VAP.

**References:**

1) Avitzur Y, van Heerden PV, Dayan L et al. Development of a device to reduce gastro-esophageal reflux in critically ill patients.Clinical Nutrit Exp 2016;7:1–8

**Table 13 (abstract P468).** See text for description

Site	Treatment group	Control group	p value
Trachea	1/26	7/36	0.22
Above the ETT cuff	24/110	46/114	0.04
Oropharynx	47/110	104/108	0.0003

Legend : Positive pepsin A samples at each site

## P469 Ascertaining correct placement of nasogastric tubes: a single-centre experience

### C Paisley^1^, J Patrick-Heselton^1^, M Mogk^2^, J Humphreys^3^, I Welters^1^

#### ^1^Royal Liverpool University Hospital, Liverpool, United Kingdom; ^2^MoreData GmbH, Giessen, Germany; ^3^New Marton Farm, Oswestry, United Kingdom

**Introduction**

Failure to detect misplacement and use of a nasogastric tube in the pleura or respiratory tract prior to commencement of feeding or administration of medication has been classified as a “Never Event” by NHS England. pH testing remains the first line test that a nasogastric feeding tube is inserted correctly. Gastric pH is often falsely elevated due to administration of antacids. Chest X-ray serves as a second line check only, but is often required to ensure correct positioning of feeding tubes into the stomach. This study aimed to 1. quantify the need for chest X-rays to confirm position of nasogastric tubes, 2. determine the percentage of patients in whom gastric aspirate with pH < 5.5 could be obtained, 3. determine the time lag until enteral feed can be started after insertion of a nasogastric tube.

**Methods**

All data on insertion of new nasogastric tubes were collected retrospectively by research nurses on Intensive Care from August to November 2016. Data collected included indication for tube insertion, level of training of person inserting the tube, pH of gastric aspirate (if obtained), need for chest X-ray request, X-ray results, time lag until feeding was initiated, and treatment with antacids.

**Results**

96 events of nasogastric tube insertion were recorded. 90% of tubes were inserted by junior doctors, 5% by consultants and 2% by nursing staff. 82 patients (85.4%) received antacids (40.6% ranitidine, 44.8% proton pump inhibitors). A gastric aspirate could be obtained in 43 patients (44.8%). 14 of these patients (32.5%) had a gastric pH of 5.5 or above, thus precluding safe initiation of enteral feeding. A chest X-ray was requested for 76 patients (79.2%), which confirmed position within the stomach in 70 patients. In 7 patients a chest X-ray was requested, although the pH of the gastric aspirate was below 5.5. In 17 patients (17.7%) it took more than 4 hours until feed was started.

**Conclusions**

A main causal factor for patient harm after initiation of enteral feeding through misplaced nasogastric tubes is misinterpretation of chest x-rays1. Although chest X-rays should only be used as second line check, when a gastric aspirate cannot be obtained or pH is inconclusive, in our patient cohort circa 80% of patients required a chest x-ray to confirm position of nasogastric tubes. A nasogastric aspirate could only be obtained in about 45% of patients. Devices to facilitate gastric aspiration may be cost efficient by reducing chest X-ray requests and may prevent patient harm.

**References**

1. http://www.nrls.npsa.nhs.uk/resources/type/alerts/?entryid45=129640

## P470 Parenteral nutrition is associated with mortality in critically ill patients

### S Pierantozzi, C Scorcella, R Domizi, E Damiani, S Tondi, E Casarotta, S Bolognini, E Adrario, P Pelaia, A Donati

#### Università Politecnica delle Marche, Ancona, Italy

**Introduction**

In previous studies, parenteral nutrition (PN) was associated with higher risk for adverse outcomes as compared to enteral nutrition (EN) [1]. We aimed to test the hypothesis that critically ill patients receiving PN have higher mortality and infection rates as compared to those not receiving PN.

**Methods**

Prospective observational study on 108 consecutive adult patients admitted to our Intensive Care Unit (ICU) from February 2016. Patients with ICU-length of stay <72 hours were excluded. We recorded type of nutrition (PN, EN, none or oral feeding), daily caloric and protein intake, microbiology results, ICU-mortality.

**Results**

**Fig. 31 (abstract P470).**
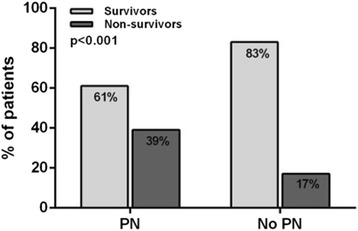
PN and mortality

**Fig. 32 (abstract P470).**
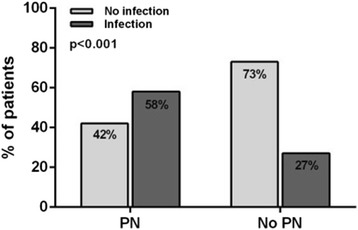
PN and infections

Thirty-one patients (29%) received PN at least one day during the ICU stay. Patients receiving PN showed higher ICU-mortality as compared to those not receiving PN (Fig. 31) and Non-survivors received PN for a longer time period as compared to Survivors (71 ± 26 versus 42 ± 25 hours, p = 0.005). The association between PN and mortality was independent of the Simplified Acute Physiology Score (adjusted odds ratio: 3.389 [95% confidence interval 1.301-8.828]). PN was associated with higher infection rate (Fig. 32).

**Conclusions**

PN was associated with higher ICU-mortality and infection rate in a general population of adult critically ill patients.

**References**

1- Elke G et al. Crit Care 2016; 20:117.

## P471 Thiamine supplementation in septic shock patients with alcohol use disorders

### MJ Holmberg, A Moskowitz, P Patel, A Grossestreuer, A Uber, LW Andersen, MW Donnino

#### Beth Israel Deaconess Medical Center, Boston, Massachusetts, United States

**Introduction**

We hypothesize that (1) septic patients with alcohol-use disorders (AUDs) do not consistently receive thiamine as part of their treatment regimen, and that (2) those not receiving thiamine have increased mortality compared to those receiving thiamine. Previous studies have shown AUDs to be associated with increased sepsis-related mortality although the mechanism through which AUDs contribute to worse outcomes has not been fully elucidated. Thiamine (vitamin B1) deficiency is a common sequela of AUDs and may contribute to impaired mitochondrial aerobic respiration leading to refractory acidosis/death if untreated.

**Methods**

We performed at retrospective analysis of all patients presenting with septic shock between 2008 and 2014 at a single tertiary care center. Patients were identified based on (1) receipt of antibiotics, (2) use of vasopressors, (3) lactate levels > 4 mmol/L, and (4) an AUD diagnosis based on ICD-9 codes. We excluded patients with seizures during the hospital stay and those with non-index events. Descriptive statistics were used to calculate the proportion of septic patients with AUDs receiving thiamine, and mortality between groups were compared using Fisher’s Exact.

**Results**

We included a total of 88 patients. The median age was 57 (quartiles: 49, 64), and 67% were male. 59/88 (67%) of all patients received thiamine and overall mortality was 43/88 (49%). In those receiving thiamine, 24/59 (41%) died, compared to 19/29 (66%) of those not receiving thiamine, p = 0.03.

**Conclusions**

In this study, we found that a considerable proportion of patients with AUDs admitted for septic shock did not receive thiamine. Thiamine administration in patients with AUDs and septic shock was associated with decreased mortality. We suspect that failure to administer thiamine in this patient population is related to a shift in clinician focus towards the septic insult and away from the AUD history, although further studies are needed to better understand this phenomenon.

## P472 Is vitamin d deficiency at ER admission associated with an increased mortality from sepsis?

### S Malinverni, D Goedeme, P Mols

#### CHU Saint Pierre, Brussels, Belgium

**Introduction**

Vitamin D deficiency has been repeatedly implicated in regulation of the innate and adaptive immune system. Evidence from ER studies investigating the association between vitamin D deficiency and mortality are currently lacking.

**Methods**

We conducted an interim analysis of a prospective study enrolling patients presenting with sepsis/septic shock at our ER. Vitamin D was sampled from patients before any fluid or drug administration. Patients were considered as vitamin D deficient if < 30 μg/L. Apache II and Charlson index were calculated for each patient.

**Results**

At November 15th 2016 96 patients completed the 3 months follow up. Vitamin D deficiency at ER admission was observed in 65.1% of patients. 42% were bacteraemic. 34.3% of patients were under pressor support during ER stay and 62% had an increased lactate. Apache II was not significantly different between groups while Charlson Age adjusted comorbidity index was significantly higher in patients with normal levels of Vitamin D (5.34 vs 7.91 p = 0.002). Mortality was higher in vitamin D deficient patients (34.2% vs 23.1% p = 0.12) in a non-significant fashion. Multivariate regression analysis showed that Apache II was independently associated with worse outcome (p < 0.001) while vitamin D status was not (p = 0.231). Mean ICU length of stay was longer for vitamin D deficient patients without reaching statistical significance (6.8 days vs 6.01 days p = 0.64).

**Conclusions**

Our interim analysis showed a trend towards an increased mortality for patients admitted to the ER for sepsis with a vitamin D deficiency. A larger sample will eventually clarify the role of vitamin D deficiency at ER admission for sepsis.

**Fig. 33 (abstract P472).**
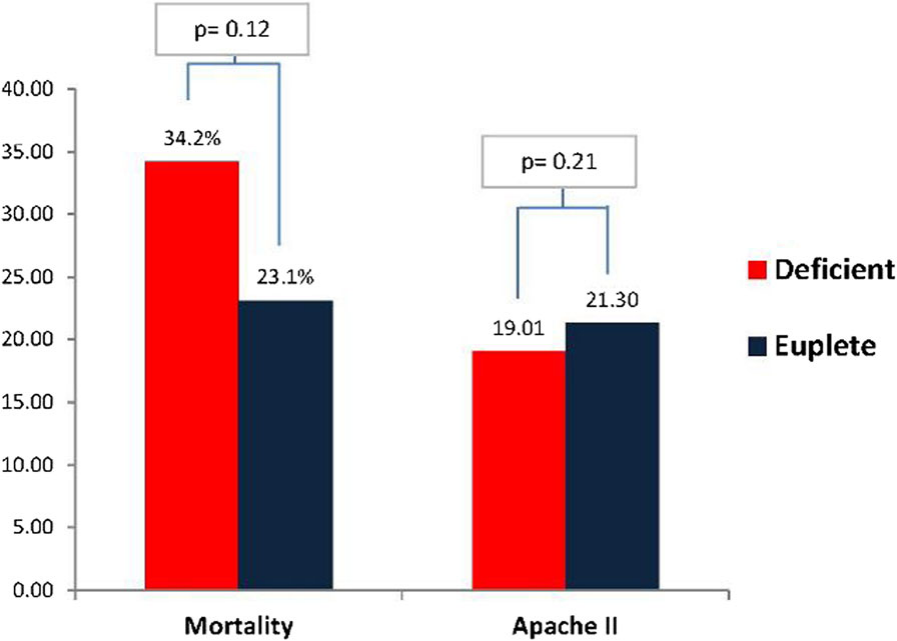
See text for description

## P473 Vitamin D supplementation in the critically ill: systematic review and meta-analysis

### P L Langlois^1^, C Szwec^2^, F D’Aragon^1^, D K Heyland^3^, W Manzanares^4^

#### ^1^Centre Hospitalier Universitaire de Sherbrooke, Sherbrooke, Canada; ^2^Hospital Posadas, Buenos Aires, Argentina; ^3^Queen’s University, Kingston, Canada; ^4^Hospital de Clinicas, Montevideo, Uruguay

**Introduction**

Vitamin D insufficiency is reported in up to 50% of the critically ill patients and is associated with adverse outcomes as increased mortality, length of stay (LOS) in ICU and hospital, and development of respiratory disorders with prolonged ventilation (1). Vitamin D supplementation is an interesting therapeutic strategy in the critically ill but clinical evidence remains unclear. The aim of this systematic review was to evaluate the clinical efficacy of vitamin D administration in critically ill patients.

**Methods**

We searched databases for randomized controlled trials (RCT) in critically ill patient comparing vitamin D administration to placebo. The studies had to report mortality, infectious complications, hospital/ICU LOS or length of mechanical ventilation. Two independent reviewers assessed eligibility, risk of bias and abstracted data onto a pretested form. When possible, we pooled data using a random effect model to estimate the relative risk (RR) for dichotomous outcome and mean difference (MD) or weighted mean difference (WMD) for continuous outcomes. Pre-defined subgroup analysis included oral-enteral vs parenteral administration, high vs low dose and sensibility analysis restricted to vitamin d deficient patient.

**Results**

**Fig. 34 (abstract P473).**
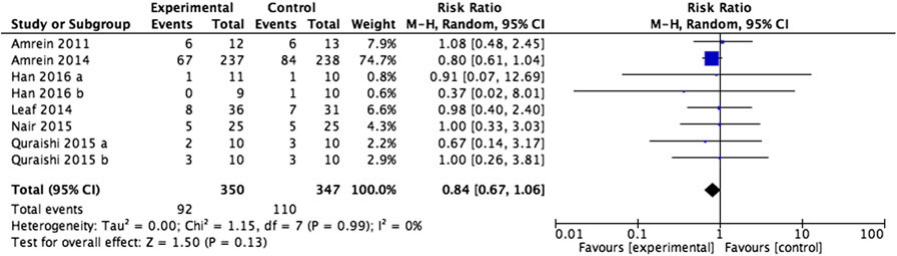
Effect of Vitamin D on mortality

Six RCTs (695 patients) met study inclusion. No overall analysis were significant. A trend towards a reduction in mortality was found when vitamin D was administered (RR = 0.84, 95% Confidence Interval [CI] 0.67-1.06; P =0.13, see Fig. 34). A trend was found towards a reduction in ICU (P = 0.17) and hospital LOS (P = 0.08). No differences in infection rate and ventilation days existed. In the oral-enteral group, a significant reduction in hospital LOS existed (P = 0.008) and a trend towards mortality reduction (P = 0.12) and ICU LOS (P = 0.12). Finally, doses higher than 300 000 IU were associated with a trend to reduce mortality (P = 0.12) and ICU LOS (P = 0.12).

**Conclusions**

In critically ill patients, Vitamin D administration does not affect overall mortality, ICU/hospital LOS, infection rate and length of mechanical ventilation. However, many analyses showed trends and seemed underpowered to detect a clinical difference, thus prompting further RCTs.

**References:** 1. Lee P et al. N Engl J Med 2009; 360:1912–14

## P474 Current pharmaconutrition practices in the ICU: results of the international nutrition survey 2014-2015

### W Manzanares^1^, C Szwec^2^, P Langlois^3^, I Aramendi^1^, D Heyland^4^

#### ^1^University Hospital, Montevideo, Uruguay; ^2^Hospital Posadas, Buenos Aires, Argentina; ^3^Université de Sherbrooke, Sherbrooke, Canada; ^4^Queen’s University, Kingston, Canada

**Introduction**

Over the last few years several randomized controlled trials examining the role of pharmaconutrition in the critical care setting have shown controversial results. The purpose of this study was to describe current pharmaconutrition practices in intensive care units (ICUs) worldwide evaluating the compliance with the evidence-based Canadian Critical Care Nutrition Clinical Practice Guidelines (CPG).

**Methods**

We conducted an international, prospective, observational, cohort study between September 2014-June 2015. Participating sites enrolled critically ill adult patients on mechanical ventilation within the first 48 hours of ICU admission and who remained for at least 72 hours in the ICU. Data on nutrition and pharmaconutrient practices were collected from admission to ICU discharge or a maximum of 12 days. Variables related to the CPG are reported as overall averages (or percentages) with the range of site averages.

**Results**

184 adult ICUs from 19 countries contributed 3926 patients to this analysis. Sixty-four percent were male, mean age was 59 (16-102) years old, average APACHE II score was 21.3 (1-55), and admission SOFA score was 6.2 (0-18).

5.4% of all patients (best site: 75.6%, and worst site: 0.0% of patients) received glutamine (GLN) by enteral, IV/parenteral or both routes. Enteral GLN was administered in 4.6%, and IV/parenteral GLN in 1.0% of patients. Moreover, 30% (0.0%-100%) of burn patients received enteral GLN, whereas this pharmaconutrient was provided in 4.4% of trauma patients (0.0%-87.5%). 6.1% of patients received selenium as single strategy or combined therapy. Most of ICUs avoid using arginine-enriched enteral formulas which were provided in 5.5% of patients (0.0%-94.4%). Fish oil (FO) enriched formula was administered in 14.7% of all patients, whereas in those with ARDS was provided in 22.4%. In patients on PN (or EN + PN) soybean oil (SO) based lipid emulsion (LE) was used in 25.3% of PN days. However, a SO sparing strategy was used in 35.6% of days, including an olive oil based LE: 13.0%, a mixture of SO, olive oil, medium chain triglycerides (MCT), and FO: 16.1%.

**Conclusions**

According to our recent data there is low utilization of enteral or parenteral pharmaconutrition in ICUs worldwide. The most frequently used pharmaconutrient is enteral GLN in the context of burn injury although we await the results of the ongoing RE-ENERGIZE trial before making stronger recommendations for its use. Finally, in compliance with current guidelines in PN patients alternative lipid emulsions are more commonly used.

## P475 Outcomes in variceal hemorrhage following the use of a balloon tamponade device

### N Stankovic, J Nadler, A Uber, M Holmberg, L Sanchez, R Wolfe, M Chase, M Donnino, M Cocchi

#### Beth Israel Deaconess Medical Center, Boston, United States

**Introduction**

With this study, we sought to describe the number of patients with variceal hemorrhage and a balloon tamponade device (BTD) who (1) survive to hospital discharge, and (2) survive to one-year following hospital discharge.

Variceal hemorrhage is associated with high morbidity and mortality. A BTD, such as the Sengstaken-Blakemore or Minnesota tube, may be used in cases of acute, uncontrollable variceal hemorrhage, when endoscopic and pharmacologic therapies prove insufficient. While the use of these devices may be effective at controlling acute bleeding, the effects on patient outcomes remain less clear.

**Methods**

In this retrospective study, we identified patients with a Sengstaken-Blakemore or Minnesota tube placement during hospitalization between 2003 and 2014 via ICD-9 coding at a single, urban, tertiary care center in Boston. Patient characteristics and outcomes were summarized using descriptive statistics.

**Results**

A total of 34 patients with a BTD were identified. Median age was 57.5 (quartiles: 47.5, 62.75) and 76% (26/34) were male. Approximately 59% (20/34) of patients survived to hospital discharge, and 41% (13/32) were alive after one year. Two patients were lost to follow-up. Of those surviving to hospital-discharge, 95% (19/20) had undergone transjugular intrahepatic portosystemic shunt (TIPS) during that hospitalization, while 36% (5/14) of patients who did not survive to hospital discharge had TIPS. One patient underwent liver transplantation during a subsequent hospitalization.

**Conclusions**

In this retrospective cohort of patients undergoing BTD therapy for variceal hemorrhage, approximately 59% of patients were alive at hospital discharge and 41% were alive after one year. Placement of a BTD as a temporizing measure in the management of acute variceal hemorrhage may be more beneficial than traditionally regarded, particularly when utilized to bridge patients to TIPS.

## P476 Psoas muscle index: an outcome predictor in living donor liver transplantation

### HK Atalan^1^, B Gucyetmez^2^, ME Kavlak^3^, S Aslan^3^, A Kargi^3^, S Yazici^3^, R Donmez^3^, KY Polat^3^

#### ^1^Acibadem Fulya Hospital, Istanbul, Turkey; ^2^Acibadem University School of Medicine, Istanbul, Turkey; ^3^Atasehir Memorial Hospital, Istanbul, Turkey

**Introduction**

Our hypothesis is that psoas muscle index (PMI) is related with outcomes in living donor liver transplantation (LDLT) patients. Liver transplantation (LT) patients usually have low physiologic reserves prior to LT [1]. In most LT centers, the MELD score is used for the prioritization of organ allocation. However, MELD score does not include the nutritional and functional status of the patients. Sarcopenia is defined as a loss of skeletal muscle mass and function with a risk of adverse outcomes and it was found to be associated with elevated postoperative complications in LT patients [2]. PMI is calculated with psoas muscle area/height2 (mm2/m2) and used to detect sarcopenia [3]. Hence, it may be an outcome predictor in LDLT patients.

**Methods**

In this study, 261 patients who underwent LDLT between 2011 and 2014 were retrospectively evaluated. Patients who were <18 years old, cadaveric liver transplant recipients and whose CT scans were missing were excluded. Demographic data, body mass index (BMI), PMI, MELD score, etiology, length of ICU stay, length of hospital stay, postoperative 7th day acute kidney injury (AKI), requirement of renal replacement therapy (RRT), reoperation, readmission and 1st year mortality were recorded.

**Results**

The first quartile of PMI for male and female patients were 397mm2/m2 and 298mm2/m2 respectively. In all patients, postoperative 7th day AKI, RRT requirement and 1st year mortality were 12.3%, 8.2% and 10.5%. There was no any correlation among PMI, BMI and MELD score. PMI was positively correlated with increase in postoperative 7th day creatinine level and length of hospital stay (r2 = 0.02 P = 0.049 and r2 = 0.06 P < 0.001). In 56 patients with sarcopenia, RRT requirement, reoperation, readmission and 1st year mortality were significantly higher than non-sarcopenia patients (P = 0.001 P = 0.003 and p < 0.001 for others). In multivariate logistic regression model, 1st year mortality was only increased 29.5-fold (8.3-104.4) by sarcopenia (P < 0.001).

**Conclusions**

BMI, MELD score or conventional evaluation of LT candidate may fail in many cases to predict prognosis after LT. Adding the PMI measurement may improve the evaluation of the patients prior to LT and can be used as an outcome predictor in LDLT patients.

**References**

1. Englesbe MJ et al. J Am Coll Surg 2010; 211:271–278

2. Masuda T et al. Liver Transpl. 2014; 20:401–407

3. Kalafateli M et al. J Cachexia Sarcopenia Muscle 2016 Feb 01

## P477 An evaluation of the usefulness of extracorporeal liver support techniques in patients hospitalized in the ICU for severe liver dysfunction with no option of transplantation

### M Piechota ^1^, A Piechota^2^, M Misztal^3^, S Bernas^4^, I Pietraszek-Grzywaczewska^4^

#### ^1^Dr Wł. Biegański Regional Specialist Hospita, Department of Anaesthesiology and Intensive Therapy – Centre for Artificial Extracorporeal Kidney and Liver Support, Łódź, Poland; ^2^University of Łódź, Department of Insurance, Łódź, Poland; ^3^University of Łódź, Chair of Statistical Methods, Łódź, Poland; ^4^Dr Wł. Biegański Regional Specialist Hospital, Department of Anaesthesiology and Intensive Therapy – Centre for Artificial Extracorporeal Kidney and Liver Support, Łódź, Poland

**Introduction**

The mortality rate in patients with severe liver dysfunction with no option of transplantation is unacceptably high [1]. The main aim of this study was to evaluate the usefulness of extracorporeal liver support (ECLS) techniques in this group of patients. Secondary aims were to identify independent risk factors and to assess the predictive values of the following scoring systems: Glasgow Coma Scale (GCS), Sequential Organ Failure Assessment (SOFA), Acute Physiology and Chronic Health Evaluation (APACHE) II, Simplified Acute Physiology Score (SAPS) II and Model of End-stage Liver Disease United Network for Organ Sharing (MELD UNOS) Modification in patients with severe liver dysfunction.

**Methods**

Data from hospital admissions of 101 patients with severe liver dysfunction (MELD UNOS > =18) who were admitted to the department of anaesthesiology and intensive therapy between 2006 and 2015 were retrospectively analysed. The study group was divided into two subgroups. SMT was a subgroup of patients receiving standard medical therapy, and SMT + ECLS was a subgroup containing patients receiving standard medical therapy complemented by at least one extracorporeal liver support procedure.

**Results**

Significantly lower ICU mortality and 30-day mortality rates were found in subgroup SMT + ECLS. In a multivariate model, independent risk factors for ICU mortality proved to be the SOFA score and prothrombin time. The highest discriminatory power for ICU mortality was demonstrated for the SOFA score, followed by APACHE II, SAPS II, MELD UNOS and GCS scores. For 30-day mortality, however, the best discriminatory power was shown for the SAPS II score, followed by SOFA, APACHE II, MELD UNOS and GCS scores.

**Conclusions**

Further studies are needed to assess the contribution of nonbiological ECLS procedures to a decrease in mortality rates in the population of patients with severe liver dysfunction. The independent risk factors for ICU mortality and 30-day mortality were demonstrated to include the SOFA score and prothrombin time. The SOFA, APACHE II, and SAPS II scores were better predictors of death than the MELD UNOS Modification score which is dedicated to the assessment of patients with liver disease.

**References**

1. Piechota M et al. An Evaluation of the Usefulness of Extracorporeal Liver Support Techniques in Patients Hospitalized in the ICU for Severe Liver Dysfunction Secondary to Alcoholic Liver Disease. Hepat Mon 16:e34127, 2016.

## P478 Degree of hyper-dynamic circulation correlates with the severity of liver disease and predicts poor outcome in spontaneous bacterial peritonitis patients in intensive care unit

### M Saleh, A Hamdy, A Hamdy, M Elhallag

#### Kasr Alainy, Cairo University, Cairo, Egypt

**Introduction**

Circulatory dysfunction is known in spontaneous bacterial peritonitis (SBP) patients. We aimed to determine whether the degree of hyper-dynamic circulation is significantly correlated with severity of liver disease and poor outcome in SBP patients or not.

**Methods**

Sixty one patients diagnosed to have SBP were enrolled. In addition to routine laboratory investigation, Child Pugh and APACHE II scores were calculated. Degree of renal impairment was defined using Acute Kidney Injury Network (AKIN) criteria. Stroke volume (SV) was measured by M mode and 2- dimensional Doppler echocardiography. Cardiac output (CO), and Systemic vascular resistance (SVR) were also calculated. All data were statistically analyzed.

**Results**

SV, CO, SVR were significantly correlated with Child Pugh score, p value <0.001, <0.001, and 0.011. In 46 (75.4%) patients had AKI, AKIN grade is significantly correlated with SVR, SV, and CO. Inpatient mortality occurred in 10 (16.4%) patients. Non-survivors had lower SVR and higher SV and CO compared to survivors (1,805 ± 105 dyn.sec/cm5, 80.3 ± 11.2 ml, and 6.7 ± 1.1 L/min vs 1,936 ± 504, 67.6 ± 12.7, and 5.7 ± 0.9, p value 0.029, 0.005, 0.007 respectively). Using the Receiver Operating Characteristics (ROC) curve, the cut-off value of CO that predict mortality was 5.89 L/min with sensitivity and specificity measuring 80%, 70.6% respectively. The area under the curve (AUC) is 0. 767 and 95% confidence interval (CI) is 0.566 to 0.968, p value 0.008. At this value, the odds ratio (OR) is 7.33 with p value 0.012.

**Conclusions**

Degree of hyper-dynamic circulation is significantly correlated with the severity of liver disease and predicts poor outcome in SBP patients.

**References**

1- Wiest R, Groszmann RJ. The paradox of nitric oxide in cirrhosis and portal hypertension: too much, not enough. Hepathology 2002;35:478–491.

2- Alessandria C, Venon WD, Marzano A, Barletti C, Fadda M, Rizzetto M: Renal failure in cirrhotic patients: Role of terlipressin in clinical approach to hepatorenal syndrome type 2. Eur J Gastroenterol Hepatol 2002;14 :1363– 1368

3- de Arujo A, Alvares da Silva MR Akin criteria as a predictor of mortality in cirrhotic patients after spontaneous bacterial peritonitis. Ann Hepatol. 2014. May- Jun; 13 (3):390–5

## P479 The i.m.p.a.c.t system® device in acute liver failure: clinical experiences in 7 adult patients

### F Atar, A Kundakci, E Gedik, H Sahinturk, P Zeyneloglu, A Pirat

#### Ankara Baskent Hospital, Ankara, Turkey

**Introduction**

The I.M.P.A.C.T (Intein Mediated Purification with a Affinity Chitin-binding Tag) System® Device is an extracorporeal support system that is used in patients awating liver transplantation, or in patients with sepsis, ARDS (acute respiratory distress syndrome), and hepatic encephalopathy in order to bind toxins and cytokines. We report a single center experience on the usefulness of the I.M.P.A.C.T System® Device in patients with acute liver failure.

**Methods**

A retrospective analysis of 7 patients who recieved treatment with the I.M.P.A.C.T System® Device due to acute or acute on chronic liver failure at Baskent University Hospital Department of Critical Care between August 2015 to August 2016.

**Results**

**Fig. 35 (abstract P479).**
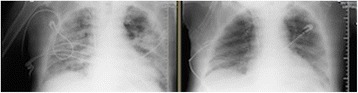
Chest X-ray findings before and after the I.M.P.A.C.T System® Device session

The mean age of 6 males and 1 female was 55.2 ± 12.8 years. Five patients had developed acute on chronic liver failure. Two patients had acute liver failure due to toxins. APACHE II (Acute Physiology and Chronic Health Evaluation) and MELD (Model for End-stage Liver Disease) scores were 17.0 ± 3.7 and 33.0 ± 4.3, respectively. At least 1 and a maximum of 6 sessions of the I.M.P.A.C.T System® Device were performed for each patient. Clinical and biochemical data were recorded before and after each of the I.M.P.A.C.T System® Device session. Following the I.M.P.A.C.T System® Device session, the mean total and direct bilirubin levels were lower when compared to the pretreatment levels. [13.7 ± 6.6 vs 17.2 ± 8.3 mg/dl (p < 0.01) and 9.1 ± 4.2 vs 11.6 ± 5.3 mg/dl (p < 0.01), respectively]. Change in serum ammonia levels were statistically significant following the I.M.P.A.C.T System® Device session (80.2 ± 80 vs 51.1 ± 25.5 mmol/l, p = 0.01). There was an improvement on chest X-ray findings after therapy among one of the patients who had acute liver failure due to intoxication (Fig. 35). Mean length of stay in the ICU was 67.1 ± 62.3 days. One patient with acute on chronic liver failure is still being treated in the ICU due to sepsis and acute kidney injury. Other 6 patients died while awaiting liver transplantation during the follow up.

**Conclusions**

Liver function tests improved after treatment with the I.M.P.A.C.T System® Device. Tolerance of therapy was acceptable as the patients’ hemodynamics were stable during and following sessions. However, controlled studies with larger number of patients are needed to show the benefit of the I.M.P.A.C.T System® Device in patients with acute liver failure.

## P480 Effects of platelet count on thromboelastometric parameters in patients with end-stage liver disease

### M Popescu, D Tomescu

#### Carol Davila University of Medicine and Pharmacy, Bucharest, Romania

**Introduction**

Patients with End-Stage Liver Disease (ESLD) have long been considered as naturally anticoagulated. Recent research has proposed a new rebalanced approach. Nevertheless thrombocytopenia remains a landmark of cirrhotic coagulopathy. Our aim was to investigate the effect of platelet count on rotational thromboelastometry (ROTEM) parameters in patients with ESLD.

**Methods**

We prospectively included patients with ESLD admitted in our ICU prior to liver transplantation. Exclusion criteria consisted of prior pro- or anti- coagulant therapy, presence of hepatocellular carcinoma or recent transfusions and splenectomy. 162 patients were included in the final analysis. Demographic parameters, liver functional tests, ROTEM assay (ExTEM, InTEM, ApTEM, FibTEM) and platelet count were noted. ROTEM parameters included: standard parameters (clotting time-CT, clot formation time-CFT, maximum clot firmness-MCF) and derived (thrombin potential index-TPI, maximum velocity of clot formation-MaxV, time to MaxV-MaxVt, area under the curve-AUC and maximum clot elasticity-MCE).

**Results**

The mean age in the study group was 50 ± 12 years and the mean severity scores (MELD, MELD-sodium) were 19.2 ± 6.1, 21.5 ± 6.0 respectively. The median platelet count was 74000 (12000-345000). A strong correlation was found between platelet count and ExTEM CFT (p = 0.03), ExTEM MCF (p < 0.01), ExTEM TPI (p < 0.01), ExTEM MaxV (p = 0.04), ExTEM AUC (p = 0.01) and ExTEM MCE (p < 0.01). We also noted a quadratic correlation between platelet count and ExTEM MCF (R2 quadratic = 0.451) and MCE (R2 quadratic = 0.362) and a linear correlation between platelet count and ExTEM TPI (R2 linear = 0.466). A cut-off value of 70000 platelets was determined below which ExTEM MCF and ExTEM TPI decreased under their normal values.

**Conclusions**

Platelet count has an important effect on extrinsically activated coagulation by ExTEM. Determining the cut-off point for clot instability as measured by derived ROTEM parameters may aid in establishing new thresholds for platelet transfusion in patients with ESLD.

## P481 Cholestatic liver injury and disturbed enterohepatic signaling due to diarrhea-induced bile salt malabsorption in critical illness

### R Van Gassel^1^, M Baggerman^1^, F Schaap^2^, M Bol^1^, G Nicolaes^2^, D Beurskens^2^, S Olde Damink^1^, M Van de Poll^1^

#### ^1^Maastricht University Medical Centre, Maastricht, Netherlands; ^2^Maastricht University, Maastricht, Netherlands

**Introduction**

We assessed the hypothesis that diarrhea during critical illness disrupts the gut-liver signaling axis through intestinal malabsorption of bile salts. Disrupted bile salt signaling could play a role in critical illness associated cholestatic liver injury, which occurs in up to 20% of ICU patients. We previously identified diarrhea as an independent risk factor for critical illness-associated liver injury (OR 4.1, 95% CI 1.2-14.5, p = 0.028, unpublished data), suggesting intestinal involvement in liver disease in the critically ill. The bile salt-induced enterokine fibroblast growth factor 19 (FGF-19) is important for normal gut-liver signaling, as it provides a negative feedback signal that limits bile salt synthesis in the liver.

**Methods**

We investigated plasma bile salt and FGF19 levels in critically ill patients. The study population consisted of enterally fed, mechanically ventilated patients admitted to our intensive care unit (ICU). There were no significant differences in demographic characteristics and ICU length of stay between groups with and without diarrhea. Patients with a primary hepato-biliary diagnosis (i.e. biliary obstruction or cholangitis) or admitted after elective surgery were excluded. After inclusion, allocation to a diarrhea group (N = 12) or no diarrhea group (N = 18) occurred based on 24 hour fecal production > = 350 ml. Data were tested for normality using the Shapiro-Wilk test and are presented as median [interquartile range] or mean ± standard deviation. Mann-Whitney or t-tests were used as appropriate.

**Results**

FGF19 levels were decreased in the diarrhea group (0.20 ± 0.12 vs. 0.29 ± 0.10 ng/mL, p = 0.03) indicating disturbed enterohepatic signaling. Plasma bile salt levels were significantly increased in patients with diarrhea compared to patients without diarrhea (9.8 [5.0-23.9] vs. 4.5 [2.9-7.4] μmol/L, p = 0.01]). Bilirubin, alkaline phosphatase (ALP) and gamma-GT levels were not significantly different between the two groups.

**Conclusions**

Diarrhea in critical illness disturbs the normal enterohepatic circulation of bile salts, as evidenced by reduced plasma levels of FGF19. This in turn may lead to unopposed bile salt synthesis in the liver. In conclusion, diarrhea and malabsorption may cause liver injury during critical illness due to disturbed enterohepatic cycling and accumulation of bile salts in the hepatocyte.

## P482 Withdrawn

## P483 The effectiveness of continuous regional arterial infusion of protease inhibitors in severe acute necrotizing pancreatitis: a retrospective multi-center cohort study

### M Horibe^1^, M Sasaki^2^, M Sanui^3^, E Iwasaki^1^, H Sawano^4^, T Goto^5^, T Ikeura^6^, T Hamada^7^, T Oda^8^, T Mayumi^9^, T Kanai^1^

#### ^1^Keio University School of Medicine, Tokyo, Japan; ^2^National Cancer Center, Tokyo, Japan; ^3^Jichi Medical University Saitama Medical Center, Saitama, Japan; ^4^Osaka Saiseikai Senri Hospital, Osaka, Japan; ^5^Hiroshima City Hiroshima Citizens Hospital, Hiroshima, Japan; ^6^Kansai Medical University, Osaka, Japan; ^7^The University of Tokyo, Tokyo, Japan; ^8^Iizuka Hospital, Fukuoka, Japan; ^9^School of Medicine University of Occupational and Environmental Health, Fukuoka, Japan

**Introduction**

Although acute pancreatitis necrotizing (ANP) has a high mortality, continuous regional arterial infusion (CRAI) of protease inhibitors has been considered to be a promising method of reducing the mortality.

**Methods**

This retrospective study was conducted among 44 institutions in Japan from 2009 through 2013. Patients 18 years or older diagnosed with ANP were consecutively enrolled.

**Results**

Of 1159 patients admitted, there were 101 (CRAI:66, non CRAI:33)patients with area of pancreatic necrosis >50%. In multivariable analysis, although CRAI of protease inhibitors was not associated with a reduction in mortality and infection rate (odds ratio [OR] 0.67, 95% confidence interval [CI] 0.19-2.36, p = 0.53; OR 0.64, 95%CI 0.18-2.25, p = 0.49; respectively), associated with need for surgical intervention (OR 0.25, 95%CI 0.07-0.85, p = 0.03).

**Conclusions**

CRAI of protease inhibitors may reduce the need for surgical intervention in the treatment of patients with severe ANP.

**References:** Horibe M, et al. PMID: 26355545.

**Fig. 36 (abstract P483).**
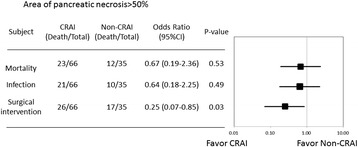
See text for description

## P484 Detection of complications after pancreas transplantation by microdialysis catheters

### G Kjøsen, R Horneland, K Rydenfelt, E Aandahl, T Tønnessen, H Haugaa

#### Oslo University Hospital, Oslo, Norway

**Introduction**

The complication rate after pancreas transplantation (PTx) is very high, with venous thrombosis being the most commonly occurring complication that leads to early graft loss. There is a lack of reliable methods to uncover complications. In this study, we investigated whether microdialysis catheters could detect complications at a timepoint where the grafts were still salvageable.

**Methods**

34 consecutive patients underwent technically similar PTx. 17 were simultaneous pancreas-kidney transplantations (SPK) and 17 were pancreas transplantation alone (PTA). The arteries were anastomosed “end-to-side” to the right common iliac artery and an elongated portal vein was anastomosed “end-to-side” to the inferior caval vein. The enteric anastomosis was performed by a duodeno-dudenostomy. At the end of surgery, two microdialysis catheters were placed anterior and posterior of the pancreas. We measured glucose, lactate, pyruvate, and glycerol bedside by sampling every 1-2 hours postoperatively.

**Results**

Of the 34 patients included, a total of nine patients developed venous thrombi. The microdialysis catheters detected all thrombi by a marked increase in lactate, which preceded any rise in blood glucose levels, and in the absence of any clinical symptoms. Median anterior lactate was 2.5 mmol/L (interquartile range (IQR) 2.2-4.1) and median posterior lactate was 2.9 (IQR 2.5-4.0) in the patients with thrombi which was significantly higher than in the uneventful group that had a median anterior and posterior lactate values of 1.1 (IQR 0.9-1.6) (p = 0.003) and 1.8 mmol/L (IQR 1.1-1.9) (p = 0.007) respectively. The presence of a thrombus was confirmed by CT. 4/9 grafts were rescued by angiographic intervention, one did not require intervention, and four grafts were explanted due to irreversible ischemic damage. All four patients with rescued grafts remain insulin-independent more than one year post transplantation.

In addition, three patients had leakages in relation to the doudeno-duodenal anastomosis. This was detected by an increase in glycerol that preceded any clinical signs for up to fourteen days. One patient required relaparotomy, another percutaneous drainage, and the last was managed with IV antibiotics alone. The three patients had higher median glycerol levels in the anterior catheter compared to the non-events, 908μM (IQR 543-1355) vs. 63μM (IQR 51-124) respectively (p = 0.002).

**Conclusions**

Monitoring lactate and glycerol with microdialysis catheters detect venous pancreas transplant thrombosis and enteral anastomosis leakage at an early stage, and may improve graft survival, patient morbidity and survival.

## P485 Measurement of LDH in acute pancreatitis - time to let go

### P Lockett, L Evans

#### Norfolk & Norwich University Hospital, Norwich, United Kingdom

**Introduction**

A widely adopted, accurate and convenient scoring system to predict severity in acute pancreatitis has eluded surgeons down the years. Despite not being supported by the most recent International Association of Pancreatology guideline the modified Glasgow score remains very widely used, in part because a score of >/=3 is often claimed (by surgeons) as an indication for admission to Critical Care Unit (CCU). The score requires measurement of Lactate dehydrogenase, as this is often not included on admission blood tests the patient must either be re-bled or the test “added-on” to stored samples. Completing the score therefore incurs an additional expense. We sought to determine whether completing the score (by measurement of LDH) has any value in predicting CCU admission.

**Methods**

All patients admitted with a diagnosis of pancreatitis to our institution in 2015 were reviewed. The components of the Glasgow score on admission were recorded along with admission to CCU, length of stay and long term outcome. Relative risk of admission to CCU was calculated in the presence or absence of LDH measurement and again for normal or abnormal levels. An LDH level of >600 U/l was regarded as abnormal. Repeated admissions of the same patient were excluded.

**Results**

There were 229 new patient episodes of acute pancreatitis in 2015. 16 (7%) were admitted to critical care. One year crude mortality was 5.7%.

Patients who had complete scoring (including LDH) had a Relative Risk of Critical Care admission of 1.27 (95% CI 0.42-3.79). Patients with an abnormal LDH had a relative risk of CCU admission of 2.57 (95% CI 0.87-7.61)

**Conclusions**

95% confidence intervals for RR of CCU admission cross 1 for both elements. These data suggest that not only is an abnormal LDH not a determinant of CCU admission but also that measuring it at all is of no clinical use. These findings support the recomendations of the recent National Confidential Enquiry into Patient Outcome and Death (NCEPOD) publication on pancreatitis. This review encouraged the adoption of the IAP guidelines in the management of pancreatitis which recommend a physiological score as sufficient in determining severity and prognosis.

LDH measurement is an unnecessary additional expense in the management of pancreatitis.

**References**

IAP/APA evidence-based guidelines for the management of acute pancreatitis. Pancreatology. 2013 Jul-Aug;13(4 Suppl 2):e1–15

Treat the Cause. A review of the quality of care provided to patients treated for acute pancreatitis. NCEPOD. 2016 July

Comparison of three Glasgow multifactor prognostic scoring systems in acute pancreatitis. Leese T1, Shaw D. Br J Surg. 1988 May;75(5):460–2

## P486 Primary anastomosis or defunctioning stoma in emergency laparotomy

### L Somerset, F Ker-Reid, S Laver, E Courtney, S Dalton, A Georgiou

#### Royal United Hospital NHS Foundation Trust, Bath, United Kingdom

**Introduction**

Emergency laparotomy is a common abdominal operation that carries a high mortality rate, recently reported in the UK at 3.6% to 41.7% [1]. With the drive to reduce associated morbidity and mortality, there is wide academic interest to examine every step in the laparotomy pathway [2]. We aimed to establish whether patients undergoing bowel resection at emergency laparotomy experience any difference in critical care or hospital outcome dependent on whether their bowel is defunctioned or a primary anastomosis is performed.

**Methods**

Retrospective review of emergency or urgent laparotomies performed at a large UK district general hospital between 2014-2015 where post-operative critical care admission was required. Data was assimilated from the electronic database. We examined: baseline patient characteristics and predicted mortality, pathology, the presence of soiling, need for repeated operation and subsequent pathology found, organ support requirements, critical care and hospital outcome and length of stay.

**Results**

We reviewed 106 patients and noted the pathology seen at initial operation varied widely between the two groups, with those defunctioned much more likely to have an inflammatory process or perforation found on surgical exploration. Patients with soiling at initial operation were 4 times more likely to have a defunctioning stoma formed. Both the APACHE II and ICNARC risk prediction models identified the defunctioned group as being at greater mortality risk; the groups were otherwise well matched at baseline. In the subgroup of patients with soiling of the abdomen at initial operation, hospital length of stay was shorter (13 vs 18 days) and more patients were discharged alive (81% vs 66%) if they had undergone defunctioning as opposed to primary anastomosis. No significant difference in organ support, need for repeated operation or overall critical care or hospital mortality or length of stay was identified between the two groups.

**Conclusions**

This is the first study to examine the impact of the decision to either anastomose or defunction bowel at emergency laparotomy and link this to post-operative morbidity and outcome. Our data suggests that patients with soiling at initial operation have a lower critical care and hospital mortality, and a shorter length of hospital stay if a defunctioning stoma is performed. No other differences were noted in any of the indices examined. This evaluation has significant implications both locally and nationally for the management of patients undergoing emergency laparotomy.

**References**

1. Saunders DI et al. Br J of Anaesth. 109:368–75, 2012

2. NELA project team. RCoA. 2015.

## P487 Pantoprazole continuous infusion versus intermittent bolus in the treatment of upper gastrointestinal bleeding

### K Robinson, T Lam, B Haas, S Reidt, K Bartlett, J Jancik

#### Hennepin County Medical Center, Minneapolis, Minnesota, United States

**Introduction**

The purpose of this study is to compare the clinical efficacy of intravenous (IV) pantoprazole intermittent bolus (IMB) dosing versus the recommended bolus and continuous infusion (CI) in subjects with upper gastrointestinal bleeding (GIB). The American College of Gastroenterology treatment guidelines for GIB recommend IV pantoprazole 80 mg bolus followed by 8 mg/h infusion [1]. In 2015, our health system switched from CI pantoprazole to IMB dosing as a response to an IV pantoprazole shortage. To our knowledge, there is currently a paucity of literature, which directly compares the two dosing strategies at consistent and specific dosages. This study aims to contribute additional data to the current body of knowledge.

**Methods**

This retrospective chart review evaluated the treatment of GIB with either pantoprazole CI or IMB. Patients who received IV pantoprazole therapy for at least 24 hours and had an upper endoscopy (EGD) with high-risk stigmata of bleeding were included. The primary outcome was rebleeding within 3 days of starting pantoprazole. Rebleeding was defined as overt bleeding with a drop in hemoglobin requiring packed red blood cell transfusion (PRBC) or additional endoscopic or surgical intervention. Secondary outcomes included rebleeding within 7 days, ICU length of stay (LOS), hospital LOS, and 72-hour PRBC requirements from start of pantoprazole. Statistical analyses were conducted using R statistical software.

**Results**

Fifty-one patients treated with IMB and 41 patients treated with CI were included. Baseline characteristics appeared to be similar between the two groups except for patients receiving an initial 80mg bolus, which occurred more frequently in the CI group (CI 78% vs. IMB 25.5%). There was no difference between either group for the primary outcome with 4 patients in the IMB group and 7 patients in the CI group who met criteria for rebleeding within 3 days (p = 0.20). Rebleeding within 7 days was similar between groups (IMB 11 vs. CI 10, p = 0.94). The mean number of PRBC received in 72 hours was 2.19 units in the IMB group and 4.5 units in the CI group (p = 0.99). There was a significant difference in ICU LOS (IMB 1 day vs. CI 2 days, p = 0.02) but not in hospital LOS (IMB 4 days vs. CI 5 days, p = 0.29).

**Conclusions**

IMB pantoprazole was comparable to the guideline recommended CI pantoprazole for the treatment of GIB in patients with endoscopic findings of high-risk stigmata of bleeding. IMB pantoprazole could be considered an equivalent alternative to CI pantoprazole in treatment of GIB and deserves further evaluation.

**References:**

1. Laine L, et al.: Am J Gastroenterol 2012; 107: 345–360

## P488 Sodium administration in a small UK ITU: audit following a change in practice

### M Bigwood

#### Queen Elizabeth Hospital, Kings Lynn, United Kingdom

**Introduction**

Our aim was to audit the amount of sodium administered to our patients and the incidence of hypernatraemia.

ITU patients receive large amounts of IV fluid, often containing high concentrations of sodium. Hypernatraemia is therefore more common and is associated with worse outcomes [1][2].

Retrospective data collection from ITU patient records over a 1 month period indicated that 96% of patients received >2mmol/kg/day of sodium. 6% became hypernatraemic. During this time, use of Hartmann’s as maintenance IV fluid (MIF) was standard practice.

NICE guideline CG174 specifies that maintenance fluid/sodium should be given by infusion of 0.18% Sodium Chloride/4% Dextrose (Dex/sal). Practice was changed and compliance audited. Audit standards used are shown in Table 14.

**Methods**

We retrospectively audited the records of all admissions to the ITU during May 2016. There were 65 patients; of which 36 were excluded (due to diagnoses that affect sodium balance), leaving 29 patients included in the audit. Data collection included patient demographics, length of stay, fluid type received, total sodium received from all sources and serial serum sodium.

**Results**

We achieved four standards and failed one (*). [Table 15]

Total sodium received per patient reduced from a mean of 936mmol to 314mmol. Sodium received in MIF as a percentage of total sodium reduced from 59% to 35% Maintenance sodium was received at a rate between 0.6-1.4mmol/kg/day in 83% of patients. 67% of patients received >2mmol/kg/day of total sodium.

96% of patients started on dex/sal had a lower serum sodium on repeat. 30% of the normonatraemic (135-145mmol/l) patients became hyponatraemic (<135mmol/l). On average these patients received 0.94mmol/kg/day maintenance sodium and 2.86mmol/kg/day total sodium. 10% of patients received <0.6mmol/kg/day of maintenance sodium, who all remained normonatraemic. There was a weak correlation between percentage serum sodium change and sodium received (total and maintenance) but no correlation if it was corrected for body weight.

**Conclusions**

We adhered to NICE standards well. We reduced total sodium administered and subsequent hypernatraemia. Hyponatraemia incidence was increased which also adversely affects patient outcome [2]. Recommendations were made to raise awareness of dex/sal induced hyponatraemia and to consider using MIF with 0.45% sodium concentration.

**References**

1. Besen BA et al. World J Crit Care Med 4(2): 116–129, 2015

2. Funk GC et al. Intensive Care Med 36(2):304–11, 2010

**Table 14 (abstract P488).** See text for description

	Guideline	Source
1	Use Dex/sal for MIF (unless documented why not)	NICE
2	Use fluid with 130–154mmol/l sodium for any resus	NICE
3	Receive 0.6-1.4mmol/Kg/day of maintenance sodium	NICE
4	Give <2.5L/day Dex/saline	NICE
5	Patients with Critical Care induced hypernatraemia	Local

Legend : Audit Standards

**Table 15 (abstract P488).** See text for description

Guideline	Target	Actual
1	100%	100%
2	100%	100%
3	75%	83%
4	100%	97%*
5	<5%	0%

Legend : Performance

## P489 Dysnatraemia and its impact on mortality on admission to intensive care

### R Hanley, P Morgan

#### East Surrey Hospital, Redhill, United Kingdom

**Introduction**

The impact of dysnatraemia on survival from Intensive Care (ICU) can be related to the degree of hypo- or hypernatraemia [1-2]. To exclude ICU acquired dysnatraemia we reviewed patients admitted to ICU with normal and abnormal sodium levels.

**Methods**

A retrospective analysis of 10,311 consecutive patients admitted to our mixed medical and surgical ICU at East Surrey Hospital was completed. We recorded their lowest sodium level within the first 24 hours of admission. Patients were grouped as follows - Sodium <114, 115-124, 125-135, 136-145, 146-155, >156 mmol/L. Statistical comparison between the groups was carried out using Chi-squared analysis.

**Results**

Of the 10311 patients collated, 401 were excluded due to incomplete data. Survival to hospital discharge was the primary endpoint. We found that severe hyponatraemia (<114 & 115-124 mmol/L) does not confer a statistically significant impact on likelihood of survival when compared with the two groups of 125-135 & 136-145. However, survival with mild hyponatraemia 125-135 mmol/l carries a mortality of 30.4% when compared with normal sodium levels (136-145 mmol/L) which has a mortality of 24.8%, p <0.0001. Patients who are hypernatraemic in the first 24 hours of ICU admission appear to have increasing mortality rates associated with the degree of hypernatraemia. A sodium level greater than 156 mmol/L has a mortality rate of 57.6% whereas 146-155 mmol/L is 43.3%. The differences between these and when compared to the normal range of sodium are statistically significant, P <0.0001

**Conclusions**

We found that hyponatraemia, unless mild, carries less statistical weight when used as a prognostic tool than hypernatraemia when measured in the first 24 hours of admission to ICU.

**References**

1. Funk, G.C., Lindner, G., Druml, W. et al. Incidence and prognosis of dysnatremias present on ICU admission Intensive Care Med (2010) 36: 304. doi:10.1007/s00134-009-1692-0.

2. Vandergyeynst F. et al. Incidence and prognosis of dysnatraemia in critically ill patients:analysis of a large prevalence study Eur J Clin Invest 2013; 43 (9): 933–948.

**Table 16 (abstract P489).** See text for description

Sodium levels (mmol/L)	Survived	Died	% Mortality
<114	25	10	28.6
115-124	119	67	36.0
125-135	1723	751	30.4
136-145	4861	1606	24.8
146-155	387	295	43.3
>156	28	38	57.6

Legend : Comparison of dysnatraemia on mortaliy

## P490 Intra-operative chloride load and peri-operative acute kidney injury in high- risk surgical patients

### D Marouli, A Chatzimichali, S Kolyvaki, A Panteli, E Diamantaki, E Pediaditis, P Sirogianni, P Ginos, E Kondili, D Georgopoulos, H Askitopoulou

#### University Hospital, Heraklion, Greece

**Introduction**

Peri-operative Acute Kidney Injury (AKI) is a common and serious complication after major surgery [1]. Whereas peri-operative risk factors associated with AKI after cardiac surgery have been well described [2], this is not the case for major non-cardiac surgery. Recently, the impact of a high chloride load on the pathogenesis of AKI has been suggested [3].

This study was performed in order to identify possible intra-operative risk factors linked to peri-operative AKI development in a group of non-cardiac surgery patients.

**Methods**

This single-centre, prospective observational study included adults undergoing elective major abdominal (including vascular) surgery. Patients with chronic kidney disease (CKD) stage IV or V were excluded. AKI was defined according to Acute Kidney Injury Network (AKIN) criteria within 48 hours after surgery [4]. Patients pre-operative demographics (sex, age, hypertension, coronary artery disease, congestive heart failure, chronic obstructive pulmonary disease, diabetes mellitus, CKD stage) and intra-operative anaesthetic management (type of surgery, intravenous fluids, blood products, vasopressors, mean arterial blood pressure, urine output and blood loss) were evaluated as possible AKI predictors. Furthermore, chloride ion content of intra-operatively administered crystalloids and colloids was estimated.

**Results**

Of 61 patients (47 males) included in the study, 10 (16.4%) developed postoperative AKI (AKI group) and 51 did not (non-AKI group). After univariate analysis, four intra-operative variables were identified as predictors of AKI: Intra-operative blood loss (p = 0.002), transfusion of fresh frozen plasma (p = 0.004) and red blood cells (p = 0.038), as well as high intra-operative chloride load (p = 0.033, AUC = 0.715 ± 0.095, cut off value >500mEq). The remaining pre- and intra-operative variables did not differ significantly between the two groups.

**Conclusions:** Isotonic saline administration has recently been associated with post-operative AKI, possibly as a result of the excess chloride load during cardiac surgery [5]. Our study’s preliminary results indicate that a high intra-operatively administered chloride load is strongly associated with increased risk of post-operative AKI in patients undergoing elective major non-cardiac surgery.

**References**

1. Hobson C et al.: Ann Surg 2015; 261:1207–1214

2. McNeal J et al.: Crit Care 2016; 20 (187):2–9

3. Krajewski ML et al.: Br J Surg 2015; 102 (101):124–36

4. Acute Kidney Injury Work Group: Kidney Intern 2012; 2:1–138

5. Kim JY et al.: Crit Care 2015; 19:350

## P491 Is chloride load an important cause of hyperchloremia in critically ill? a retrospective analysis

### FG Zampieri^1^, AB Liborio^2^, BA Besen^3^, AB Cavalcanti^1^

#### ^1^HCor-Hospital of the Heart, São Paulo, Brazil; ^2^Universidade de Fortaleza - UNIFOR, Fortaleza, Brazil; ^3^Hospital das Clínicas - FMUSP, São Paulo, Brazil

**Introduction**

High chloride (Cl) load is one of the putative causes of hyperchloremia in critically ill patients. We aimed to assess and quantify the association between chloride load during the first two days of ICU admission and changes in chloride levels between admission and day 3.

**Methods**

We analyzed adult patients with at least two days of ICU stay included in the MIMIC II. Cl load was calculated as the sum of the Cl infused in the first 48 hours from common solutions (normal saline, half normal saline, lactated ringer, HES). The association between change in Cl levels (day 3 minus day 1) and Cl load was assessed by linear regression. We performed sensitivity analysis according to serum creatinine at admission (<1 mg/dL; 1-2 mg/dL; 2-3 mg/dL or higher > 3 mg/dL), occurrence of any degree of acute kidney injury in the first 48 hours (above KDIGO 1), in quartiles of diuresis in the first 48 hours and in oliguric (<0.5 mL/kg/h in the first 48 hours) patients.

**Results**

**Fig. 37 (abstract P491).**
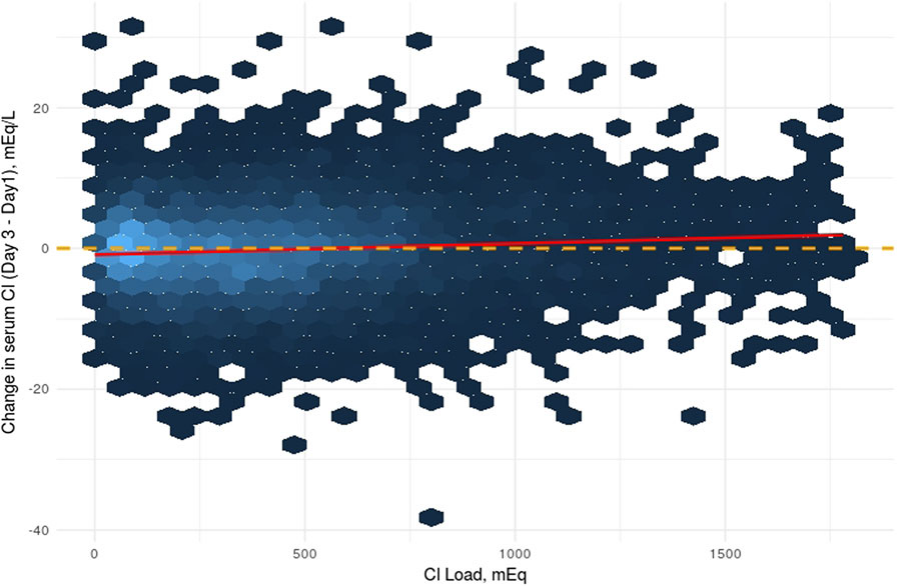
Density hexagonal binning for the association between Cl load and change in Cl

9,043 patients were included in the analysis. Median chloride load was 350 mEq (IQ 162-579 mEq). On linear regression, higher chloride load was weakly associated with an increase in serum chloride (coefficient 0.001; SE 0.0002; p < 0.001 – Fig. 37, red line), but correlation and R-squared were low (0.08 and 0.007, respectively). These findings were consistent in all subgroups assessed.

**Conclusions**

Although a high chloride load is associated with an increase in serum chloride, the association was weak even patients with low urinary output and high creatinine. This suggests that chloride load may not be the principal cause of hyperchloremia in critically ill patients.

## P492 Hypercloremia and acid-base changes in critically ill patients requiring fluid resuscitation

### C Dominedò, AM Dell’Anna, A Monayer, DL Grieco, R Barelli, SL Cutuli, A Ionescu Maddalena, E Picconi, C Sonnino, C Sandroni, M Antonelli

#### Catholic University of the Sacred Heart, A. Gemelli University Hospital, Rome, Italy

**Introduction**

Hyperchloremia is common in ICUs [1]. Short term and in vitro studies [2] show that crystalloid infusions may lead to hyperchloremia and metabolic acidosis due to low strong ion difference (SID). It is not known if changes in serum chloride (sCl) concentration in vivo yield to mid-term SID and acid-base (AB) imbalance. We performed a study to assess the effects of sCl increase on SID and AB equilibrium in critically ill patients.

**Methods**

The present monocentric, retrospective, physiological study enrolled a cohort of critically ill adult patients admitted to ICU from January 2009 to June 2015, who required fluid resuscitation and developed hyperchloremia (2 consecutive sCl values > =110 mEq/L). Changes in AB and electrolyte equilibrium within ICU admission and the day of maximum chloremia were studied. Data are expressed as median [interquartile range].

**Results**

21 patients were included in the study: age 72 [58-81] years, female 9 (43%), SAPS II 50 [38-68]. At ICU admission 9 patients (43%) had shock, SOFA was 8 [5-11], pH 7.37 [7.31-7.44], SID 27 [21-29] mEq/L, sCl concentration 112 [110-117] mEq/L; 5 patients (24%) had hyperchloremia. Maximum sCl concentration (120 [116-122] mEq/L) was observed in the remaining 16 patients 36 [24-48] hours after ICU admission and an administration of 3.8 [2.3-4.5] L of crystalloids. 9 patients (43%) died at 28 days follow up. In the overall cohort, sCl increase was not accompanied by significant changes in SID and pH. However, 7 patients (43%) developed hyperchloremic acidosis with lower SID and 9 patients (57%) hypernatremic alkalosis with higher SID. Patients with hyperchloremic acidosis during hyperchloremia showed a higher clinical severity than those with hypernatremic alkalosis (respectively, MAP 65 [57-76] vs 95 [82-116] mmHg, p 0.006; daily fluids administered 4532 [3813-5443] vs 2428 [2080-4243] mL, p 0.01; serum albumin 2.4 [2-2.8] vs 3 [2.7-3.4] mg/dL, p 0.07).

**Conclusions**

SCl concentration increase can be accompanied by pH variations, according to sodium levels and SID changes. It has been suggested that both hyperchloremic and hypernatremic patients have a higher risk of mortality. Whether and to what extent AB alterations contribute to the detrimental effects of hyperchloremia on clinical outcome remain unknown and further adequately powered studies are warranted.

**References**

1. Yunos NM, et al. Bench-to-bedside review: chloride in critical illness. Crit. Care 2010;14:226

2. Carlesso E, et al. The rule regulating pH changes during crystalloid infusion. Intensive Care Med. 2011;37:461–8.

## P493 The relationship between standard base excess and base excess chloride

### B Gucyetmez^1^, HK Atalan^2^, F Tuzuner^3^, N Cakar^1^

#### ^1^Acibadem University School of Medicine, Istanbul, Turkey; ^2^Acibadem Fulya Hospital, Istanbul, Turkey; ^3^Arnavutkoy Hospital, Istanbul, Turkey

**Introduction**

Our hypothesis is that there is an interaction between base excess chloride (BEcl) and standard base excess (SBE). We know that electrolytes are independently related with acid-base status (1). Even in acute respiratory acidosis, renal compensation occurs on urinary output of sodium and chloride (2). SBE formula derivated from Van Slyke equation doesn’t include any electrolyte effect (3). Yet, BEcl is defined as an effect of chloride on SBE and calculated with (Na-Cl-32) in accordance with partitioned base excess approach (4). An interaction between BEcl and SBE can provide better understanding primary reasons and compensations of acid-base disorders.

**Methods**

In this study, 2736 patients were retrospectively evaluated beetween 2011 and 2016. Patients who were <18 years old, readmitted, whose ICU scores, blood gases and outcomes were unknown were excluded. Demographic data, blood gases’ parameters and outcomes were recorded.

**Results**

Two thousand patients were included in this study. There were positive correlation between BEcl and each of SBE, HCO3 and PaCO2 (r2 = 0.22 P < 0.001; r2 = 0.29 P < 0.001; r2 = 0.27 P < 0.001). In patients with lactate > 5mmol L-1, there was a positive correlation between BEcl and lactate (r2 = 0.04 P = 0.011). In correlation graphic of BEcl and SBE, it was found four different areas which leaded four different clinical status such as SBE > 3 plus BEcl > 0 (the 1st area; contribution of hypochloremic alkalosis on alkalosis), SBE > 3 plus BEcl < 0 (the 2nd area; hyperchloremic acidosis plus respiratory compensation), SBE < -3 plus BEcl < 0 (the 3rd area; contribution of hyperchloremic acidosis on acidosis) and SBE < -3 plus BEcl > 0 (the 4th area; hypochloremic compensation in acidosis).

**Conclusions**

BEcl is positively correlated with SBE, HCO3 and PaCO2. Hence, it can be used to explaine the effects of sodium and chloride on SBE. Actually, BEcl may lead a primary reason or a part of compensation of any acid-base disorders.

**References**

1. Stewart PA. Can J Physiol Pharmacol 1983; 61: 1444–61

2. Ramados J et al. Can J Physiol Pharmacol 2011; 89: 227–31

3. Sigaard-Anderson et al. Acta Anaesthesiol Scand 1995; 107:123–8

4. O’Dell E et al. Crit Care 2005; 9:R464–70

## P494 A prospective observational study of rational fluid therapy in Asian intensive care units (RaFTA)

### M Jacob ^1^, S Sahu^2^, YP Singh^3^, Y Mehta^4^, KY Yang^5^, S Kuo^6^, V Rai^6^, T Cheng^7^, C Ertmer^8^

#### ^1^Klinikum St. Elisabeth Straubing GmbH, Straubing, Germany; ^2^Krishna Institue of Medical Science, Secunderabad, Andhra Pradesh, India; ^3^Max Hospital, Patparganj, New Delhi, India; ^4^Medanta-The Medicity, Haryana, India; ^5^Taipei Veterans General Hospital; School of Medicine, National Yang-Ming University, Taipei, Taiwan; ^6^University of Malaya, Kuala Lumpur, Malaysia; ^7^Sultanah Aminah Hospital, Bangunan Induk, Jalan Persiaran Abu Bakar Sultan, Johor, Malaysia; ^8^University Hospital Muenster, Muenster, Germany

**Introduction**

Fluid therapy in critically ill patients, especially timing and fluid choice, varies substantially between centres and countries. For many Asian countries, there is few data about the common practice volume therapy. We aimed to study the current approaches in India, Taiwan and Malaysia concerning patient populations, treatments and outcomes within the RaFTA registry.

**Methods**

RaFTA is an observational study in Asian ICU patients focussing on fluid therapy and related outcomes. Multivariable Cox regression was performed to identify risk factors for increased 90 day mortality and renal failure.

**Results**

24 study centres joined the RaFTA registry and collected 3,187 patient data sets from November 2011 to September 2012. A follow up was done 90 days after ICU admission. For 90 day mortality, significant risk factors in the overall population were sepsis at admission (OR 2.185 [1.799; 2.654], p < 0.001), cumulative fluid balance (1.032 [1.018; 1.047], p < 0.001), and the use of vasopressors (OR 3.409 [2.694; 4.312], p < 0.001). For the use of colloids a significant survival benefit was observed (0.655 [0.478; 0.900], p = 0.009). The initial colloid dose was not associated with an increased risk for renal failure (OR 1.094 [0.754; 1.588], p = 0.635). Exploratory research within the database suggests that timing and protocol of colloid use might affect outcome for mortality: In late colloid receivers (later than ICU day 3), the survival benefit for colloids disappeared (1.798 [0.600; 5.383], while it was more pronounced in early colloid receivers (ICU day 1, 0.523 [0.312; 0.878]).

**Conclusions**

After multivariable Cox regression analysis, colloid use was not associated with an increased risk of mortality. Furthermore, our data indicate that effects of colloids on clinical outcome may be dependent on the time of use.

## P495 Effects of balanced crystalloids and colloids on haemostasis: in vitro assessment

### P Czempik

#### Medical University of Silesia, Katowice, Poland

**Introduction**

According to current guidelines [1], crystalloids and colloids are used as the first line treatment in fluid resuscitation during massive bleeding. However, they may have deleterious impact on haemostasis. Therefore we aimed to investigate effects of balanced crystalloid and colloid solutions on coagulation and fibrinolysis in vitro.

**Methods**

Blood samples drawn from 32 young healthy males were diluted with study fluids to make a 20-vol% end-concentration. The following fluids were used: balanced crystalloid (Plasmalyte®), 4% succinylated gelatin (Geloplasma®), 6% HES 130/0.4 (Volulyte®). Rotational thromboelastometry (ROTEM®delta) and platelet aggregometry (Multiplate®) were implemented at baseline and after dilution. CBC, aPTT, PT, fibrynogen, D-dimers were also performed.

**Results**

**Fig. 38 (abstract P495):**
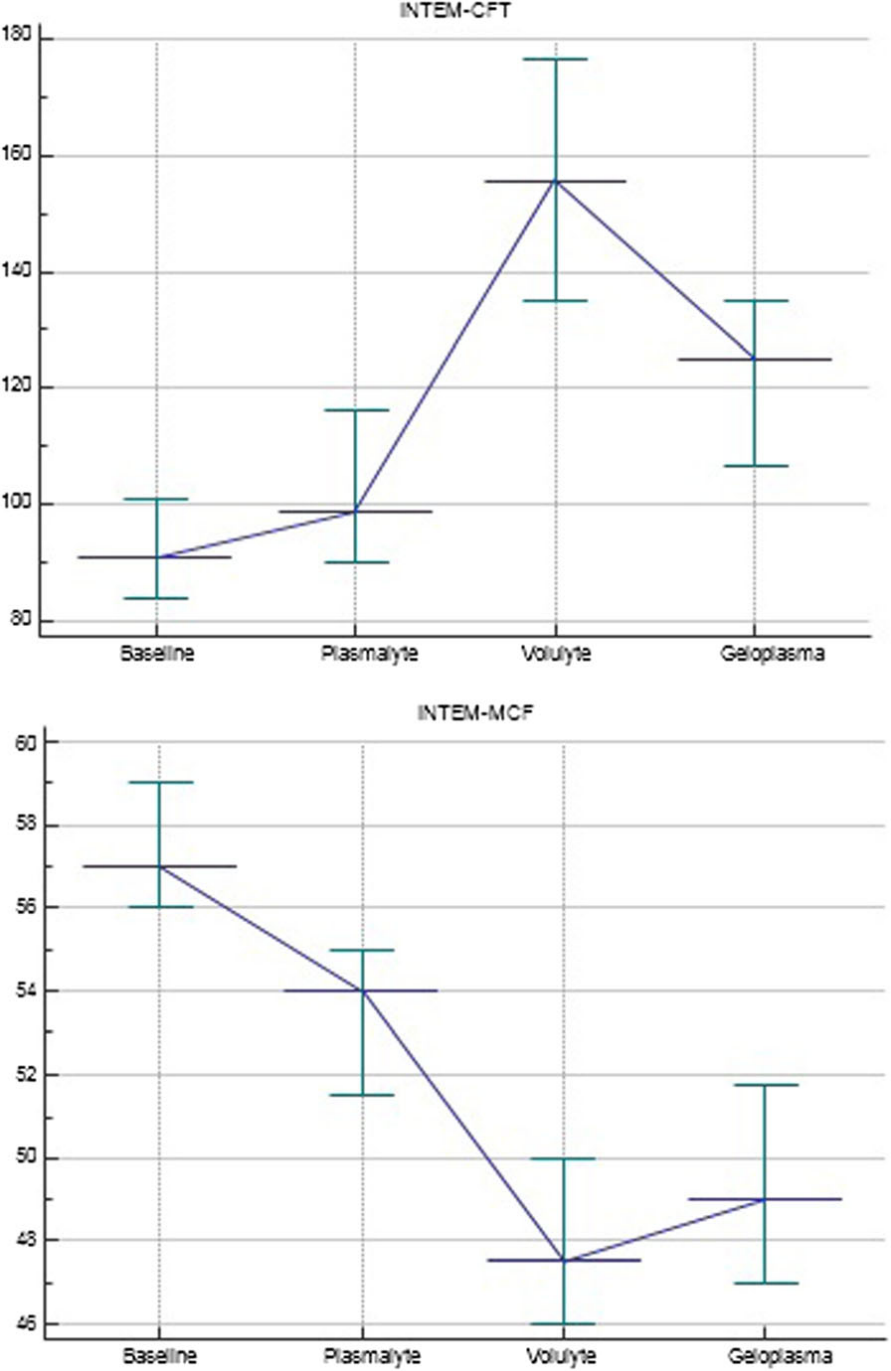
INTEM CFT and MCF after mixing with study fluids

Both succinylated gelatin and HES showed deranged INTEM (CFT, AA, A10 and MCF) and FIBTEM (A10, MCF) parameters (p < 0.01), however the effect of HES was more apparent (Fig. 38). In EXTEM, only HES significantly affected coagulation (i.e. CT prolongation). There was no effect on fibrinolysis or platelet function, as evidenced by unchanged ML and TRAP, respectively. In standard laboratory tests dilutional effect was found, however all investigated parameters stayed within reference values. No correlation was found between functional and standard coagulation tests.

**Conclusions**

Despite marked haemodilution balanced crystalloid cannot affect coagulation. Balanced colloids impair clot formation and firmness, however the effect of HES is more pronounced. None of the fluids significantly impact fibrinolysis or platelet function.

**References**

1. Rossaint R et al. The European guideline on management of major bleeding and coagulopathy following trauma: fourth edition. Crit Care. 2016.

## P496 Microcirculatory hypoperfusion combined with early fluid resuscitation with 0.9% saline predisposes to the development of trauma induced coagulopathy in a porcine model of complex haemorrhagic shock

### S Hutchings ^1^, S Watts^2^, C Wilson^2^, C Burton^2^, E Kirkman^2^

#### ^1^Kings College Hospital, London, United Kingdom; ^2^Defence Science & Technology Laboratory, Salisbury, United Kingdom

**Introduction**

We examined the sublingual microcirculation in a porcine model of THS to assess the impact of microcirculatory dysfunction on the development of TIC in animals treated with initial 0.9% saline or blood products. Development of trauma induced coagulopathy (TIC) is an important modifiable factor associated with excess morbidity and mortality following traumatic haemorrhagic shock (THS). Tissue hypoperfusion, possibly resulting from impairment of the microcirculation, is a pre-requisite for the development of TIC. The early use of blood products may attenuate TIC but is logistically difficult and identification of patients at highest risk would allow more effective targeting of this resource.

**Methods**

This study was conducted in accordance with the Animals (Scientific Procedures) Act, 1986. 19 terminally anaesthetized Large White pigs were subjected to a standardized hind-limb injury followed by a controlled haemorrhage of approximately 35% of blood volume. A 30 min period of shock was followed by 60 min of hypotensive resuscitation with either 0.9% saline or packed red cells:fresh frozen plasma after which all animals were treated with PRBC:FFP. Sublingual IDF videomicroscopy was performed and animals were divided into above and below average perfused vessel density (PVD) groups based on the lowest recorded measurement taken during the shock and initial resuscitation phases.

Coagulation was assessed using thromboelastography (TEG) and PT, APTT.

**Results**

8 animals had above average perfusion (PVD 10.5 ± 2.5 mm/mm2) and 11 below average perfusion (PVD 5.5 ± 4.1mm/mm2). For animals initially treated with 0.9% saline (n = 10) there were significant differences between those with above and below average perfusion in terms of R time (14.7 ± 2.6 v 46.6 ± 26.9 s, p <0.01), MA (72.2 ± 3.4 v 66.7 ± 4.8 mm, p 0.01) and APTT (15.0 ± 2.8 v 18.3 ± 3.9 p 0.03) 60 min after the start of resuscitation. However, animals initially treated with PRBC:FFP (n = 9) showed no such differences.

**Conclusions**

In a porcine model of complex THS the development of TIC appeared to be associated with the presence of impaired microcirculatory perfusion and initial treatment with 0.9% saline. Early administration of blood products appeared to attenuate TIC, even in the presence of tissue hypo-perfusion.

**References:** © Crown copyright (2016), Dstl

## P497 Validation of a pharmacokinetic equation for individualised phosphate replacement in critically ill patients: a retrospective observational cohort study

### D Drennan, A O’Prey, A MacKay, R Forrest

#### Queen Elizabeth University Hospital (QEUH), NHS Greater Glasgow and Clyde, Glasgow, United Kingdom

**Introduction**

Hypophosphatemia occurs in around 20% of patients in intensive care units (ICU), similar to the incidence at the QEUH. There is not a standardised phosphate (PO4) replacement calculation and current practice in the QEUH is to replace 40mmol of PO4 as standard. In 2013, a Dutch study concluded the following pharmacokinetic equation to be effective when used for individualising PO4 replacement:

Dose(mmol) = (0.5* x weight(kg)) x (1.25ǂ – plasma PO4(mmol/L)) [1]

*Volume of distribution (L/Kg)

ǂ Target plasma PO4 (mmol/L)

This study aims to assess the validity of this equation in our population.

**Methods**

Electronic medical records were reviewed retrospectively to identify patients who received PO4 replacement from August to November 2016. Patient characteristics, PO4 dosing and levels were collected. The above equation was used to calculate a theoretical dose which was then compared to the actual dose administered.

**Results**

**Fig. 39 (abstract P497).**
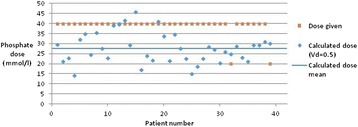
Theoretical Calculated PO4 Dose vs Administered PO4 Dose

Of 200 patients reviewed, 40 received intravenous PO4. Using the calculation, 34 patients would have received lower doses than administered following current practice (Fig. 39). Of these, 19 had PO4 within the therapeutic range (0.7-1.4) post administration of 40mmol. The theoretical dose exceeded the actual dose in five cases. In total there were 51 PO4 administrations, 40 of which did not reach the target concentration of 1.25mmol/L defined in the equation. The remaining eleven achieved levels >1.25mmol/L; five of which the plasma concentration exceeded the upper limit of 1.4mmol/L. Using the information collected a new average volume of distribution (Vd) was calculated to be 1.2L/Kg.

**Conclusions**

The proposed pharmacokinetic equation for individualising PO4 replacement is not valid for QEUH ICU patients. One possible explanation for this could be due to the difference in the apparent volume of distribution. A new volume of distribution and equation was calculated from the above data. This will be trialled and assessed for validity.

**References**

1. A Bech et al.: Journal of Critical Care 28: 838–843, 2013

## P498 Hydroelectrolitic rebalancing in diabetic ketoacidosis in children

### A Oglinda^1^, G Ciobanu^2^, M Casian^2^, C Oglinda^2^

#### ^1^Institute of Mother and Child, Chisinau mun., Moldova; ^2^State University of Medicine and Pharmacy, Chisinau, Moldova

**Introduction**

Diabetic ketoacidosis in children (DKA) is characterized by the existence of some hydroelectrolitic disorders particularly serious which not being founded and treated on time endangers life.

**Methods**

N = 116, from the ED, ICU and endocrinology for the period of 15.01.2014 and 01.09.2016. Two lots: I lot 48 children with DKA who have been under treatments in the ED of pediatric resuscitation and the II lot 68 children control group who have been under the treatment in the department of endocrinology. They have been evaluated primary and secondary emphasizing the signs of dehydration, blood sampling for determining the ions of K, Na, Cl, P, acid-base balance, glucose, and urinary ketones.

**Results**

Children were transported by the emergency in proportion of 81%. According to age children > 9 have predominated, gender: b n = 60; g n = 56 (p <0.001). Degree of dehydration: 42 children mild dehydration under 4%, in 58 cases we founded moderate signs of dehydration 4-7%, and children with serious signs of dehydration -16. We have founded the level of K ions and of ABB while being hospitalized and in dynamics. In lot I, we registered values of K under 3 mmol/l, in comparison with the values of 3.5mmoles/l of K in the control lot (p =0.001).They received infusion of crystalloid and K supplements, the necessary volume and quantity of K was calculated separately according to the known formula. After the infusion– in children from the I lot: the initial values of Ph 7.09 ± 0.07; pCO2 11 ± 1.5; BE –(-) 18.7 ± 0.9 had a tendency of amelioration after 30 minutes in 6 cases, in 19 cases just after 2 hours an amelioration, in 16 cases after 12 hours, simultaneously in other 7 cases the amelioration of the described above parameters was observed after 24 hours. No child needed the administration of Na bicarbonate for the normalization of metabolic acidosis. The average time of treatment 13 ± 0.9 days in I lot, in the control lot 7 ± 0.7 (p = 0.03).

**Conclusions**

1. Early administration of infusion with the goal of hydroeletrolitic and acid-base balancing lead to a more rapid amelioration of the hemodynamics in children with ketoacidosis and reduces the time of being in-patient unit.

2. Correct administration of infusion therapy in ketoacidosis eliminates the necessity of Sodium bicarbonate administration with the goal of metabolic acidosis.

## P499 Metformin-associated lactic acidosis requiring intensive care in a regional hospital in Hong Kong and predictive factors for mortality

### CT Lun^1^, HJ Yuen^2^, G Ng^2^, A Leung^2^, SO So^1^, HS Chan^1^, KY Lai^2^

#### ^1^Alice Ho Miu Ling Nethersole Hospital, Hong Kong, Hong Kong; ^2^Queen Elizabeth Hospital, Hong Kong, Hong Kong

**Introduction**

Metformin-associated lactic acidosis (MALA) is a severe condition with inconsistent factors for mortality in previous studies. The relationship between the timing of commencement of RRT and survival has not been investigated.

**Methods**

This is a 5-year retrospective case series in patients with MALA in a regional hospital in Hong Kong. The primary outcome is to identify factors associated with intensive care unit (ICU) mortality, specifically to investigate the relationship between mortality and time from admission to RRT. The secondary outcomes are to investigate the characteristics and outcomes of patients with different precipitating factors and to identify factors for duration of RRT dependency.

**Results**

59 patients were eligible for analysis. Compared with the 54 survivors, the 5 non-survivors had higher APACHE IV scores, APACHE IV predicted mortality risk, temperature, heart rates, PaCO2 levels, and nor-adrenaline dosage and lower first 24 hr urine volume and serum albumin. They were more likely to be on mechanical ventilation and suffer from sepsis. They had longer time from hospital admission to commencement of RRT. The ROC curve had AUC of 0.776 and sensitivity and specificity of 80% if cut off value was set at 765.5 mins. Patients with sepsis were found to be more severe with higher mortality. Sepsis as a precipitating factor and high baseline serum creatinine level were two factors identified for longer RRT dependence.

**Conclusions**

Our study identified factors associated with mortality in metformin-associated lactic acidosis, including time from admission to commencement of RRT, suggesting benefit of early RRT. Further prospective studies may be necessary to determine the optimal timing of initiation of RRT for MALA in future.

**Table 17 (abstract P499).** See text for description

Characteristics	Non-survivor n = 5	Survivor n = 54	p-value
APACHE IV score	145 (30.0)	108.9 (26.2)	0.005
PaCO2 (kPa)	4.93 (1.45)	2.89 (1.34)	0.002
24 hour urine volume (ml)	45 (17.5-110)	589.5 (153.8-1427.5	0.013
Maximum nor-adrenaline(mcg/kg/min)	2.03 (1.36-2.94)	0.54 (0.11-1.63)	0.009
Time from admission to RRT (min)	1180 (515-2070)	386.5 (136.5-662.5)	0.043

Legend : Factors associated with ICU mortality

## P500 The comparison between intravenous sodium bicarbonate plus NAC and intravenous sodium chloride plus NAC to prevent contrast induced nephropathy in ED

### P Sanguanwit^1^, W Charoensuk^2^, B Phakdeekitcharoen^3^

#### ^1^Faculty of medicine Ramathibodi, Bangkok, Thailand; ^2^Srisungwan Hospital, Mae Hong Son, Thailand; ^3^Faculty of Medicine, Ramathibodi Hospital, Mahidol University, Bangkok, Thailand

**Introduction**

Contrast-induced nephropathy (CIN) was common complication of radiologic procedures and increased morbidity, mortality, and acceleration toward ESRD. The objective was to compare IV sodium bicarbonate plus NAC and IV sodium chloride plus NAC to prevent CIN in ED.

**Methods**

We performed a prospective single-center RCT of 83 patients who had GFR between 30-60 ml/min/1.73 m2. 42 patients received IV sodium bicarbonate plus NAC 1 hour before contrast and continued for 6 hours. 41 patients received IV sodium chloride plus NAC in the same manner. In both groups, NAC (1,200-mg IV bolus before contrast injection then 1,200 mg orally twice daily for 24 hours) was administered. Primary outcome was the incidence of CIN.

**Results**

CIN occurred 4 patients (9.8%) in sodium bicarbonate group and 6 (15.4%) in sodium chloride group (P = 0.51). Sodium bicarbonate group had significant increased GFR at 72 hours {50.1 (31.3, 99.8), 57.0 (24.8, 117.8), P 0.004} than Sodium chloride group {50.5 (30.3, 105.9), 57.9 (19.2, 99.4), P 0.06}.There were not statistically significant in volume overload, renal replacement therapy and mortality at 7 days

**Conclusions**

Hydration with sodium bicarbonate might be effective as sodium chloride for prevention of CIN in emergency situations.

**References**

Recio-Mayoral A et al; JACC, 2007

**Table 18 (abstract P500).** See text for description

Baseline	Sodium Bicarbonate Plus NAC, (N = 42)	Sodium Chloride Plus NAC (N = 41)	P
Cr (mg/dl)	1.2(±0.3)	1.2(±0.3)	0.67
eGFR (ml/min)	52.1(±15.5)	51.5 (±14.3)	0.86

Legend : baseline characteristic

**Table 19 (abstract P500).** See text for description

Outcome	Sodium Bicarbonate Plus NAC (N = 42)	Sodium Chloride Plus NAC (N = 41)	p
CIN	4(9.8%)	6(15.4%)	0.51

Legend : Incidence of CIN

**Fig. 40 (abstract P500).**
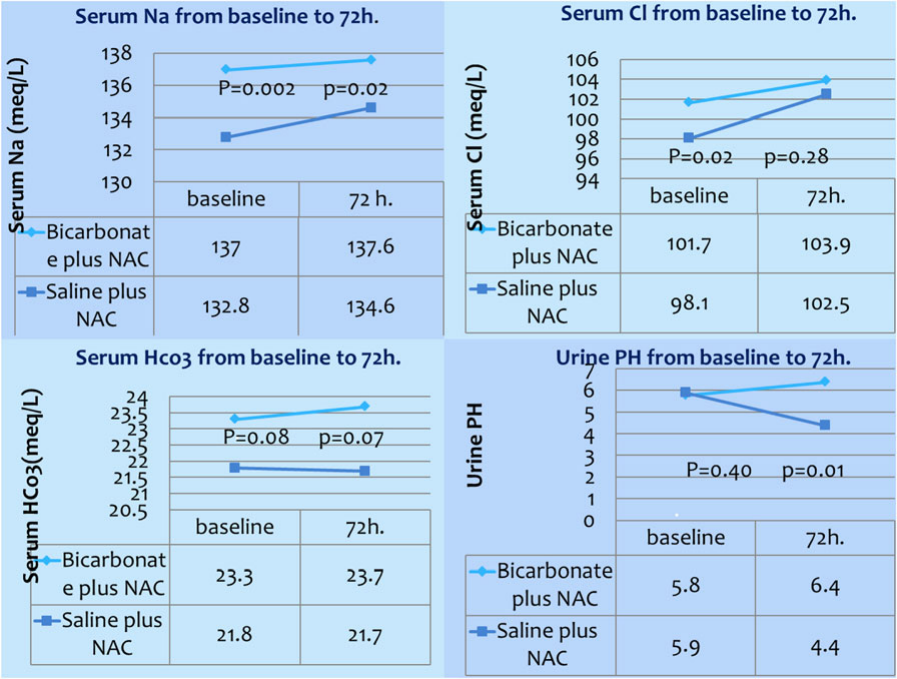
eGFR from baseline to Day 7

**Fig. 41 (abstract P500).**
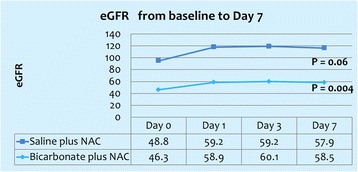
Chemistry from baseline to 72 hr

## P501 Prophylaxis of contrast-induced nephropathy: haemodynamic effects of hydration with sodium bicarbonate and theophylline

### G Batres-Baires, I Kammerzell, T Lahmer, U Mayr, R Schmid, W Huber

#### Klinikum rechts der Isar; Technical University of Munich, Munich, Germany

**Introduction**

Contrast-induced nephropathy (CIN) is one of the most frequent aetiologies af hospital-acquired acute renal failure (ARF). Strategies to prevent CIN include the renal vasodilator theophylline and hydration with sodium bicarbonate (NaBic). A recent study demonstrated synergistic prophylactic effects of theophylline and NaBic. Nevertheless, considering the total volume load of 9mL/kg of NaBic 0.154 mmol/L and the potential pro-arrhythmic effects of 200mg theophylline, haemodynamic side effects cannot be ruled out.

Therefore, we analyzed the impact of NaBic hydration and theophylline on haemodynamics in 22 ICU-patients with PiCCO-monitoring (Pulsion Medical Systems SE; Germany).

**Methods**

Patients undergoing an X-ray examination with parenteral contrast-medium (CM) received NaBic according the protocol by Merten et al. (3ml/kg/h for 1h before CM and 1ml/kg/h for 6h after CM) and 200mg of theophylline i.v. over 15min immediately before CM.

PiCCO-measurements were performed immediately before (TPTD-1) and after 1h-pre-hydration (TPTD-2), after theophylline (TPTD-3), after CM (TPTD-4) and after the 6h- post-CM-hydration (TPTD-5). Primary endpoint: Comparison of last vs. first PiCCOmeasurement. Statistics: IBM SPSS 23.

**Results**

22 CM-enhanced computed tomographies were performed.

Comparison of the last (TPTD-5) to the 1st (TPTD-1) measurement demonstrated significant increases in global end-diastolic volume index GEDVI (817 ± 270 vs. 765 ± 229mL/m^2^; p = 0.038), cardiac index CI (5.64 ± 1.81 vs. 4.34 ± 1.81 L/min/m^2^; p = 0.020) and cardiac power index (0.87 ± 0.37 vs. 0.79 ± 0.38 W/m^2^; p = 0.037). By contrast, SVRI (1254 ± 607 vs. 1513 ± 854 dyn*s*cm-5*m-2; p = 0.042) and pulmonary vascular permeability index PVPI (1.66 ± 0.59 vs. 1.94 ± 1.03; p = 0.009) significantly decreased (p = 0.009). CVP, systolic and diastolic arterial pressure, heart rate HR, dPmax, extravascular lung water index, EVLWI and global ejection fraction GEF did not change. In none of the patients there was a change in heart rhythm.

With regard to potential side effects of theophylline, we compared haemodynamics before (TPTD-2) and after theophylline application (TPTD-3). Theophylline application resulted in a moderate, but significant increases in heart rate (99 ± 22 vs. 92 ± 20/min; p < 0.001) and GEF (28.5 ± 7.0 vs. 27.7 ± 7.2%; p = 0.024). There were no changes in heart rhythm.

**Conclusions**

NaBic according to the Merten-protocol and 200mg of theophylline did not result in detrimental effects on haemodynamics. A slight increase in heart rate immediately after theophylline was transient and accompanied by an improvement in GEF.

## P502 Presepsin for early detection of acute kidney injury and mortality prediction in cardiac surgery patients

### E Spanuth^1^, H Bomberg^2^, M Klingele^3^, R Thomae^4^, H Groesdonk^2^

#### ^1^DIAneering GmbH, Heidelberg, Germany; ^2^Department of Anaesthesiology, Intensive Care Medicine and Pain Medicine, Saarland University, University Medical Centre, Homburg/Saar, Germany; ^3^Department of Medicine, Division of Nephrology and Hypertension, Saarland University, University Medical Centre, Homburg/Saar, Germany; ^4^Mitsubishi Chemical Europe, Düsseldorf, Germany

**Introduction**

Presepsin (PSEP) has been shown powerful prognostic validity in inflammatory related conditions like sepsis and association with disease severity, multi organ dysfunction syndrome and outcome. We thought to evaluate the prognostic value of PSEP for outcome prediction in patients undergoing elective cardiac surgery.

**Methods**

We included consecutive patients having cardiac surgery and measured plasma concentration of PSEP, NT-pro-BNP, PCT, leucocytes, and cystatin C. PSEP was determined by using the PATHFAST Presepsin assay (LSI Medience corporation,Tokyo). Outcome measures were in-hospital mortality, 6- month mortality and occurence of acute kidney injury (AKI) during hospitalization.

**Results**

Patients with in-hospital mortality (n = 27, 3.2%) and 6-month mortality (n = 49, 6.1%) had higher preoperative presepsin levels than survivors: 1166 ± 1453 pg/mL vs. 258 ± 391 pg/mL; p < 0.001 and 913 ± 1215 pg/mL vs. 231 ± 194 pg/mL; p < 0.001, respectively. C-statistics showed elevated presepsin level to accurately predict occurrence of in-hospital mortality (AUC 0.88) and 6-month mortality (AUC 0.87) whereas the EuroSCORE 2 showed significantly less predictive power (AUC values 0.74 and 0.76) as well as NT-pro-BNP (AUCs 0.77 and 0.79), PCT (AUCs 0.59 and 0.56), leucocytes (AUCs 0.58 and 0.63), and cystatin C (AUCs 0.76 and 0.74).

222 patients (25.9%) who developed AKI (AKI classification: 1 (n = 122), 2 (n = 54), 3 (n = 46)) revealed higher mortality and higher presepsin values compared to patients without AKI (in-hospital mortality: 8% vs 1.4%; preoperative presepsin: 441 ± 585 vs 233 ± 436 pg/mL; p < 0.001; postoperative presepsin: 927 ± 926 vs 426 ± 583 pg/mL; p < 0.001). ROC analysis of postoperative presepsin showed the highest discriminatory power for risk prediction of AKI occurence (AUC 0.78) and renal replacement therapy (AUC 0.88) during hospitalization in comparison with the other diagnostic markers and EuroSCORE 2. Even after adjustment for confounding factors (i.e. EuroSCORE 2, age, glomerular filtration rate, and operation duration) presepsin remained an independent risk predictor.

**Conclusions**

Preoperative PSEP is a predictor of postoperative mortality and AKI in cardiac surgery patients, and is a stronger predictor than commonly used factors. PSEP has proven as an independent risk indicator to predict outcome and may be used for risk stratification in patients scheduled for surgery.

**Table 20 (abstract P502).** See text for description

	AKIN = 0 (n = 635), median (IQR)	AKIN = 1,2,3 (n = 222), median (IQR)	RO-AUC
EuroScore II	3.15 (1.63-6.13)	6.23 (3.5-12.8)	0.701
PCT, ng/L	0.65 (0.30-1.59)	1.56 (0.60-5.34)	0.674
PSEP, ng/L	306 (228-436)	632 (378-1077)	0.782

Legend : Validity of EuroScore 2, PSEP, and PCT for detection of post-operative AKI

## P503 Life-threatening exertional rhabdomyolysis in a 35-year-old ultramarathon runner – case report

### S Bernas, M Piechota, K Mirkiewicz

#### Dr Wł. Biegański Regional Specialist Hospital, Department of Anaesthesiology and Intensive Therapy – Centre for Artificial Extracorporeal Kidney and Liver Support, Łódź, Poland

**Introduction**

Exertional rhabdomyolysis in long-distance runners has been reported since the late 1970s as a condition precipitated by overheating, significant physical exertion and dehydration. The case report concerns a 35-year-old man who was admitted to the ICU at the 27th hour after completing a 24-hour ultramarathon race during which he ran a distance of about 205 km, after staying for a few hours in the Cardiac Department where the man was admitted for bradycardia (ventricular rhythm rate: 25 bpm), considerable fatigue and whole body pain. During hospitalization in the Cardiac Department, the patient was diagnosed with left ventricular hypokinesis with the EF of 40%, marked hyperkalaemia (8.41 mmol/l) accompanied by hyponatraemia (123 mmol/l) and hypochloraemia (86 mmol/l) as well as elevated levels of creatinine (6.18 mg/dl), urea (220 mg/dl), liver enzymes (AST – 6,799 U/I, ALT – 2,030 U/I), CK (332,900 U/l), CK-MB mass (>300 ng/ml), troponin T (0.109 ng/ml), and anuria. The level of myoglobin determined on day 2 of the patient’s ICU stay was > 30,000 ng/ml.

**Methods**

Aside from standard treatment, with a view to managing hyperkalaemia and protecting the kidneys from further damage, CVVHD CiCa haemodialysis with an EMIC2 filter was performed, leading to a rapid stabilization of the patient’s clinical condition including sinus rhythm recovery. The EMIC2 filter and/or the CVVHD CiCa kit was/were initially replaced every 12-24 hours. At the same time, the patient, who was monitored haemodynamically, despite anuria received fluid therapy so as to achieve mild arterial hypertension due to hypervolaemia. The rate of ultrafiltration was adjusted to the patient’s hydration level.

**Results**

The treatment administered to the patient led to establishing a diuresis of over 400 ml/day on day 6 after admission. CVVHD was discontinued on day 11 of hospitalization, when the patient’s spontaneous diuresis was 1,600 ml/day. Following CVVHD discontinuation, diuresis was aided by the intake of mannitol and the continuous infusion of furosemide. The patient was discharged on day 17 of hospitalization, in the polyuric phase of up to 9,300 ml/day (eGFR 17.40 ml/min/1.73 m2).

**Conclusions**

A combination of intensive fluid therapy provided under haemodynamic monitoring with continuous renal replacement therapy using a large pore filter ensuring myoglobin filtration in patients with acute renal failure caused by exertional rhabdomyolysis is a safe method of patient management which may reduce kidney damage and speed up the recovery of normal renal function after exercise-induced rhabdomyolysis.

## P504 Impact on the development of acute renal injury of two doses of glucocorticoids during cardiopulmonary bypass in cardiac surgery

### A González Pérez, J Silva

#### Hospital Universitario Central de Asturias, Oviedo, Spain

**Introduction**

Cardiac surgery with cardiopulmonary bypass (CPB) triggers a systemic inflammatory response syndrome (SIRS) that is associated with postoperative morbidity and mortality. Steroids may attenuate this response but there is currently controversy over its use. This SIRS is associated with acute renal injury (AKI) during the ICU stay.

We aimed to assess the effects of two dosage of steroids in CPB in AKI development during ICU stay.

**Methods**

Retrospective observational study of a cohort of patients that underwent to cardiac surgery at our institution between 2006 and 2014. Two groups were compared, one group administers 1 gr of methylprednisolone (MTPN) during cardiopulmonary bypass and another group administers 2 gr. AKI development during ICU stay was defined as impaired renal function with elevation to twice baseline creatinine or need of renal replacement therapy. Demographic data, clinical and surgical variables were collected from de Hospital database. Values expressed as mean +/- SD or %. Significant variables in the univariate analysis were entered into a multivariate logistic regression model to calculate the odds ratio with confidence interval 95%.

**Results**

4573 patients; 64.8 males; 67.78+/- 11.13 years old; BMI 28.36 +/- 6.81; Logistic EuroScore 6.36 +/- 4.85; ICU stay 6.58 +/- 15.41 days; postoperative bleeding at first 24 hours 560.74 +/- 447.95 cc; preoperative haemoglobin 13.58 +/- 5.72 gr/ dL; preoperative urea and creatinine 51.39 +/- 23.76; 1.13 +/- 0.57 mg / dl respectively. By pass time 104.31 +/- 231.75; cross clamp time 71.76 +/- 32.74 minutes. Two groups were compared: group 1: MPNL (1 gr) ;group 2 with 2 gr of MPNL during CPB and analyzed its relation with the development of AKI during ICU stay. Patients who received higher doses of steroids had a lower risk of developing AKI: Relative Risk 0.891, 95% CI (0.817 to 0.972) p = 0.009. Multivariate analysis showed that a dose of 2 grams of MTPN reduced the incidence of AKI in the postoperative period of cardiac surgery OR 1.44 95% CI (1.09 to 1.91) p = 0.012.

**Conclusions**

Higher dose of steroids was shown to reduce the incidence of AKI after cardiac surgery.

## P505 Factors that predict supranormal glomerular filtration in critical diseases

### A Ramos^1^, F Acharta^1^, M Perezlindo^1^, L Lovesio^1^, P Gauna Antonelli^1^, A Dogliotti^2^, C Lovesio^1^

#### ^1^Sanatorio Parque, Rosario, Argentina; ^2^Grupo Oroño, Rosario, Argentina

**Introduction**

An augmented renal clearance (ARC) has been described in some critical patient groups [1]. The aim of this study was to identify factors predicting supranormal glomerular filtration in critically ill patients with normal plasma creatinine and polyuria.

**Methods**

This is a prospective study in a surgical and medical intensive care unit (ICU). From October 2015 to October 2016, patients with normal plasma creatinine and polyuria (>3 liters of urine / 24 hours) were included. We exclude individuals referred from another hospital. Creatinine clearance, sodium concentration and diuresis were calculated from a 24-hours urine collection. Percentile 75th was determined to define supranormal glomerular filtration (199 ml / min / 1.73 m2). The logistic regression model was used to identify independent associated variables.

**Results**

**Fig. 42 (abstract P505).**
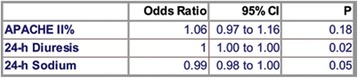
Multivariate analysis

Thirty-six patients were included in the study. Twenty three patients increased renal clearance (>130 ml / min / 1.73 m2). Nine (25%) patients were above 75th percentile (>199 ml / min / 1.73 m2). Seven patients had a neurological cause of admission. In multivariate analysis, the volume of diuresis for 24 hours predicts ARC (Fig. 42).

**Conclusions**

A relationship between ARC, urinary sodium and diuresis volume should be studied in a larger cohort of patients.

**References**

1- Udy AA et al. Augmented Renal Clearance in the ICU: Results of a Multicenter Observational Study of Renal Function in Critically Ill Patients With Normal Plasma Creatinine Concentrations. Crit Care Med 42:520–527, 2014.

## P506 Macrophage migration inhibitory factor versus neutrophil gelatinase-associated lipocalin-2 to predict acute kidney injury after liver transplantation

### J Baron, J Schiefer, DM Baron, P Faybik

#### Medical University Vienna, Vienna, Austria

**Introduction**

Several biomarkers have been suggested as early predictors of acute kidney injury (AKI) after orthotopic liver transplantation (OLT). Systemic and urinary neutrophil gelatinase-associated lipocalin-2 (NGAL) appear to be promising predictors of AKI after OLT, but their clinical benefit remains to be proven [1, 2]. Recently, systemic macrophage migration inhibitory factor (MIF) has been proposed as an early indicator for requirement of renal replacement therapy after OLT [3]. We hypothesized that either systemic or urinary MIF can predict the development of AKI after OLT with comparable power as systemic and urinary NGAL.

**Methods**

This prospective, observational pilot study was performed at the Medical University of Vienna after local ethics committee approval and registration at clinicaltrials.gov (NCT02695979). Concentrations of MIF and NGAL were measured in serum and urine samples collected from patients undergoing OLT. Acute kidney injury was classified according to the KDIGO criteria, with stages 2 and 3 summarized as severe AKI. Areas under the receiver operating curves (AUC) were calculated to assess predictive values of MIF and NGAL for the development of severe AKI.

**Results**

Forty-five patients (mean age 55 ± 8 years) were included. Nineteen patients (38%) developed severe AKI within 48 hours after OLT. After OLT, MIF concentrations in serum and urine were greater in patients with AKI than those without AKI. In contrast, only urine NGAL concentrations, but not serum NGAL concentrations were greater in patients with AKI than those without AKI. At the end of OLT, serum MIF was predictive of AKI (AUC 0.73; 95% confidence intervals, CI [0.55-0.90]; P = 0.03), while urinary MIF, serum NGAL, and urinary NGAL were not. On the first postoperative day, serum MIF (AUC 0.78; CI [0.62-0.93]; P = 0.006), urinary MIF (AUC 0.71; CI [0.53-0.88]; P = 0.03), and urinary NGAL (AUC 0.79; CI [0.64-0.93]; P = 0.02) were predictive for AKI, while serum NGAL was not.

**Conclusions**

In the setting of OLT, serum MIF predicted severe AKI earlier than serum NGAL and urinary NGAL.

**References**

1. Niemann CU et al. Liver Transplantation 15:1852–60, 2009

2. Portal AJ et al. Liver Transplantation, 16:1257–66, 2010

3. Stefaniak J et al. Liver transplantation, 21:662–9, 2015

## P507 Factors associated with lack of full renal recovery after 90 days in critically ill patients with AKI

### HP Shum^1^, WW Yan^1^, TM Chan^2^

#### ^1^Pamela Youde Nethersole Eastern Hospital, Hong Kong, Hong Kong; ^2^The University of Hong Kong, Hong Kong, Hong Kong

**Introduction**

A significant portion of patients does not achieve full renal recovery by 90 days after an episode of acute kidney injury (AKI). This study aimed to elucidate the factors that predict lack of full renal recovery on Day 90 (D-90) in critically ill patients with AKI.

**Methods**

The study population included all patients who showed KDIGO stage 2/3 AKI upon ICU admission or during ICU stay in a regional acute hospital over the period 1/1/2011-31/12/2013 and who survived on D-90. Patients with prior end stage renal failure were excluded. Renal recovery was regarded as ‘full’ if serum creatinine (SCr) < =125% baseline, which was defined as the lowest known SCr value in the preceding 3 months before ICU admission.

**Results**

Among 3687 patients admitted to ICU during the study period, baseline renal function was available in 87.3%. 843 patients fulfilled the inclusion criteria. AKI was attributed to sepsis in 52.2%. 177 patients (21.0%) did not achieve full renal recovery on D-90, and their characteristics included older age (67.2 vs. 63.3 years, p = 0.004), lower body weight (57.4 vs. 60.8 kg, p < 0.001), non-surgical admission diagnoses (cardiovascular disease RR 1.85, renal disease 1.71), presence of comorbidities (diabetes mellitus RR 1.70, malignancy 2.04), lower serum albumin level (27.0 vs. 28.7g/L, p = 0.012), lower hemoglobin level (9.5 vs. 10.4 g/dL, p < 0.001), higher admission SCr (226 vs. 158 umol/L, p < 0.001) and requirement of renal replacement therapy (34.5% vs. 21.6%, p < 0.001) during ICU stay. Hypovolemia was a less frequent cause of AKI in these patients (20.9% vs. 36.5% in patients who had full renal recovery on D-90, p < 0.001). Logistic regression analysis showed that diabetes mellitus, malignancy, admission for cardiovascular disease, AKI unrelated to hypovolemia, SCr >110umo/L and hemoglobin <10g/L on ICU admission were independent predictors for lack of full renal recovery on D-90.

**Conclusions**

Medical comorbidities (such as diabetes mellitus, malignancy, anemia and cardiovascular disease), AKI not attributed to hypovolemia, and elevated SCr level upon ICU admission are risk factors associated with failure of full renal recovery at three months after an episode of AKI in critically ill patients.

## P508 Early predictive markers of peri-operative acute kidney injury in non-cardiac major surgery

### D Marouli, A Chatzimichali, S Kolyvaki, A Panteli, E Diamantaki, E Pediaditis, P Sirogianni, P Ginos, E Kondili, D Georgopoulos, H Askitopoulou

#### University Hospital, Heraklion, Greece

**Introduction**

Peri-operative Acute Kidney Injury (AKI) is a well defined entity associated with significant morbidity and mortality [1]. While several serum and urine AKI biomarkers have been identified in the cardiac surgery population [2], the predictive value of those markers in non-cardiac surgical patients has not been extensively studied. The aim of this prospective observational study was the evaluation of different prognostic biomarkers in a cohort of patients undergoing elective major surgery.

**Methods**

61 patients, 67.1 ± 10 years old, with no chronic kidney disease (CKD) stage IV or V undergoing elective major abdominal surgery were studied. AKI was defined according to Acute Kidney Injury Network (AKIN) criteria within 48 hours after surgery [3]. At pre-defined time points [Pre-op (pre-operatively), RR (recovery room), Post-op (24 & 48 hours post-operatively)] the following biomarkers were measured: Serum creatinine (Scr), Cystatine C (Scyst), Retinol Binding Protein (SRBP), urine á1-microglobulin (Ua1-micr), â2-microglobulin (Uâ2-micr), Transferrin (Utransf) and Albumin (Ualb). Fractional excretion of sodium (FeNa) and urea (FeUr) were calculated as well.

**Results**

10 out of 61 patients (16.4%) developed AKI. In univariate analysis elevated Pre-op Ua1-micr value (p = 0.032), as well as elevated RR levels of Scyst (p = 0.004) were strongly associated with peri-operative AKI development. At 24hours, post-op Scyst (p = 0.001), SRBP (p = 0.002), Ua1-micr (p = 0.002), FeNa (p = 0.010) and FeUr (p = 0.007) were also strong markers of AKI. The remaining markers did not differ significantly between the two groups.

**Conclusions**

Urine á1-microglobulin and serum RBP have been recognised as markers of early tubular damage in diabetic nephropathy [4]. Our preliminary results identified these two low-molecular weight proteins as possible early markers of peri-operative AKI in a well-defined cohort of non-cardiac surgical patients. With the exception of diabetic nephropathy and acute-on-chronic renal injury [5-7], this is the first study recognising the potential of these two novel biomarkers for early prediction of peri-operative AKI.

**References**

1. Hobson C et al.: Ann Surg 2015; 261:1207–1214

2. Schley G et al.: PLoS ONE 2015; 10(12)

3. Acute Kidney Injury Work Group: Kidney Intern. 2012; 2:1–138

4. Nikolov G et al.: Scripta Scientifica Medica 2013; 45(3):58–64

5. Takebayashi K et al.: Journal of Clin Endocr and Met 2007; 92(7):2712–2719

6. Zahra N et al.: Int J of Biol Sc and Appl 2014; 1(1):15–18

7. Yanhong Y et al.: J Nephrol 2016 Jul 7. [Epub ahead of print]

## P509 Bioelectrical impedance analysis measurements as predictors of impaired renal function after cardiac surgery

### V Vicka, D Gineityte, D Ringaitiene, J Sipylaite, J Pekarskiene

#### Vilnius University, Vilnius, Lithuania

**Introduction**

Bioelectrical impedance analysis (BIA) is a fast and non-invasive technique used to evaluate hydration status. As acute kidney injury (AKI) remains one of the most common complications after cardiac surgery, the aim of this study is to determine whether BIA can be used to predict AKI.

**Methods**

This was a prospective observational study, conducted in cardiac surgery centre of Vilnius University hospital Santariskiu Clinics. Consecutive patients scheduled for elective cardiac surgery were enrolled during a period of one year. BIA was performed one day prior surgery; relevant demographic and clinical data was gathered. These patients were observed until discharge from hospital or up to one month. Impaired renal function was defined by the Society of Thoracic Surgeons (STS) proposed criteria of renal failure (RF). The most accurate BIA markers were selected and further analysed.

**Results**

**Fig. 43 (abstract P509).,:**
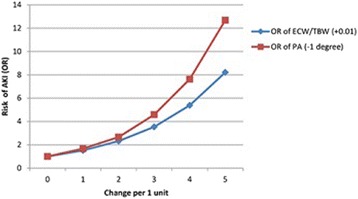
Predictive risk of ECW/TBW and phase angle for STS renal failure

618 patients were enrolled. Most of these patients were men 67% (n = 414), had a CABG procedure 61.5% (n = 380) and were at low operative risk with a median Euroscore II value of 1.76 [1.06-2.50]. The mean values of BIA parameters were as follows: ICW (mean = 25.67, SD = 7.68), ECW (mean = 16.40, SD = 4.69), TBW (mean = 42.07, SD = 12.32), ECW/TBW ratio (mean = 0.39, SD = 0.01) and phase angle (PA) (mean = 5.54, SD = 0.92). STS RF rate was 4.2% (n = 26). ROC AUC analysis of the BIA parameters revealed a positive predictive effect of ECW/TBW (AUC = 0.63 CI95%: 0.51-0.76 p = 0.021) and a negative predictive effect of PA (AUC = 0.63 CI95%: 0.51-0.74 p = 0.03) for RF. The univariate regression analysis of RF risk factors produced a standardized risk of 1.52 times (CI95%: 1.13-2.06 p = 0.006) for 0.01 increase of ECW/TBW and a risk of 1.66 times (95% CI: 1.07-2.60 p = 0.025) for a decrease of 1 degree of PA. These results are presented in Fig. 43.

**Conclusions**

BIA parameters can be used to stratify risk of RF for patients scheduled for cardiac surgery. However, further studies are needed to determine whether these factors are independent of other predictors.

## P510 Extracellular histones in machine perfusate of donation after circulatory death kidneys correlate with graft function after transplantation

### DM Beurskens^1^, TC Van Smaalen^2^, P Hoogland^2^, B Winkens^3^, MH Christiaans^2^, CP Reutelingsperger^1^, E Van Heurn^4^, GA Nicolaes^1^

#### ^1^Cardiovascular Research Institute Maastricht (CARIM), Maastricht, Netherlands; ^2^Maastricht University Medical Center (MUMC), Maastricht, Netherlands; ^3^School for Public Health and Primary Care (CAPHRI), Maastricht, Netherlands; ^4^Academic Medical Center Amsterdam, Amsterdam, Netherlands

**Introduction**

Extracellular histones are cytotoxic molecules that are associated to cell stress and cell death. Presence of extracellular histones has been linked to multiple inflammatory–driven pathologies such as auto-immunity, tissue injury and sepsis [1]. We previously showed that the presence of extracellular histones correlates with mortality in sepsis patients [2], a disease state in which histones are considered as important mediators in disease progression. A potential role for histones in another condition in which tissue condition is considered of vital importance, in organ donation and graft function and survival, is still unknown. The aim of this study was therefore to assess whether an association exists between the presence of extracellular histones in machine perfusates and deceased donor kidney viability.

**Methods**

Extracellular histone H3 was determined in machine perfusates of 390 donation after circulatory death (DCD) kidneys by semi-quantitative Western Blotting. Corresponding graft function and survival were assessed to study a potential association with the presence of histone H3 in the perfusate fluid.

**Results**

Extracellular histone H3 concentrations were significantly higher in perfusates of DCD kidneys with post-transplant graft dysfunction (primary non-function (PNF) median 0.73μg/ml (IQR, 0.44-1.00), p < .001) and delayed graft function (DGF) (median 0.70μg/ml (IQR, 0.43–0.98), p < .001)) compared to immediate functioning kidneys (median 0.42μg/ml (IQR, 0.07–0.78)). We identified histone H3 as an independent risk factor for both DGF (OR = 2.152 (1.199–3.863)) and one year graft failure (HR = 1.386 (1.037-1.853)), but not for PNF (OR = 1.342 (0.900-2.002)).

**Conclusions**

Extracellular histone concentrations in perfusates of kidneys with post-transplant graft dysfunction were significantly higher than in those grafts that show an immediate function. Follow-up studies are planned to further investigate the potential of cytotoxic extracellular histones in the assessment of post-transplant graft function and survival, as well as possible histone-based interventions to optimize graft preservation protocols.

**References**

[1] Allam R et al. J Mol Med 92:465–472, 2014

[2] Wildhagen KC et al. Thrombosis Research 136:542–7, 2015

## P511 Biomarkers of cell cycle arrest and cell death as early predictors for delayed graft function in patients following kidney transplantation – results of an observational clinical study

### FS Schmitt, ES Salgado, JF Friebe, TF Fleming, JZ Zemva, TS Schmoch, FU Uhle, LK Kihm, CM Morath, CN Nusshag, MZ Zeier, TB Bruckner, AM Mehrabi, PN Nawroth, MW Weigand, SH Hofer, TB Brenner

#### University Hospital Heidelberg, Heidelberg, Germany

**Introduction**

Prolonged cold ischemia time (CIT) is associated with an increased ischemia and reperfusion injury resulting in an increased damage to the graft, as well as a delayed graft function. The aim of this study was to figure out the impact of prolonged CIT on the clinical course, the inflammatory immune response and related cell cycle arrest, respectively cell death.

**Methods**

In total, 91 patients were evaluated for short-term complications and delayed graft function within 10 days after the kidney transplantation. 48 patients received a graft from deceased donors, whereas 43 underwent living donor transplantation. Blood and urine samples were collected before surgery, immediately after the end of the surgical procedure, and 1, 3, 5, 7 and 10 days later. Plasma and urine levels of total keratin 18 (total K18) and caspase cleaved keratin 18 (ccK18), plasma levels of Interleukin-6 (IL-6) and urine levels of insulin-like growth factor-binding protein-7 and tissue inhibitor of metalloproteinase-2 (IGFBP-7/TIMP-2) were measured.

**Results**

An initial interim analysis of one third of all participating patients revealed a significantly prolonged CIT in patients undergoing kidney transplantation from deceased donors, resulting in an increased inflammatory response as well as a significantly increased necrotic cell death. In these patients urinary IGFBP-7/TIMP-2 and plasmatic total keratin 18 was demonstrated to be of value for the detection of a delayed graft function in the early phase after kidney transplantation as assessed by receiver operating characteristic (ROC)-analyses.

.

**Conclusions**

For early identification of patients at high risk for delayed graft function, the implementation of IGFBP-7/TIMP-2 and total keratin 18 measurements in routine diagnostics after kidney transplantation should be taken into consideration.

## P512 Effectiveness of renal resistive index (RRI) in predicting acute kidney injury of early onset in critically ill patients

### G Fotopoulou^1^, I Poularas^2^, S Kokkoris^1^, E Brountzos^1^, S Zakynthinos^1^, C Routsi^1^

#### ^1^National and Kapodistrian University of Athens, Athens, Greece; ^2^General Hospital of Athens, Athens, Greece

**Introduction**

Acute kidney injury (AKI) in critically ill patients is associated with increased morbidity and mortality. The renal resistive index (RRI), derived from the Doppler spectrum of segmental and interlobar arteries, is a rapid, non-invasive diagnostic technique, mainly studied in assessing renal-allograft status, that has been recently proposed to predict renal outcomes in critically ill patients [1]. The objective of our study was to evaluate the accuracy of RRI measurement on Intensive Care Unit (ICU) admission in detecting acute kidney injury (AKI) development the upcoming 48 hours.

**Methods**

We performed a prospective observational study in a multidisciplinary university ICU. Patients requiring mechanical ventilation, without pre-existing chronic renal failure were enrolled. Clinical data including serum creatinine and urine output were collected. Illness severity scoring systems (APACHE II and SOFA) were calculated on the first ICU day. The RRI was calculated according to the following formula: RRI = (peak systolic velocity – end-diastolic velocity)/peak systolic velocity as the average of all measured values. The index was measured within the first 24 hours of ICU admission, after initial hemodynamic stabilization. AKI was defined according to the Kidney Disease Improving Global Outcomes (KDIGO) Clinical Practice Guidelines [2]. ROC curves were constructed in order to evaluate the AUC of RRI for AKI prediction.

**Results**

Sixty-three mechanically ventilated, critically ill patients were included in the study, (median age 66 years, 55% were males). Median values for APACHE II and SOFA scores were 18 and 9, respectively. AKI developed in 24 patients (38%) during the first 48h post-ICU admission; 14 patients (22%) had AKI already on ICU admission. Continuous renal replacement therapy (CRRT) underwent 44% of patients with AKI and the all-cause ICU mortality rate was 38%. AUC of RRI for AKI diagnosis on admission was 0.94 (CI: 0.86-1.0), while for AKI prognosis within the first 48h after admission (in the subset of patients who had no AKI on admission) was 0.96 (CI: 0.90-1.0). The total predictive ability of RRI for AKI development (both diagnosis and prognosis) within the first 48h after ICU admission was 0.95 (CI: 0.88-1.0). AUC of RRI for CRRT initiation prediction during ICU stay was 0.81 (CI: 0.69-0.93).

**Conclusions**

These results suggest that an increased RRI on ICU admission may be used as a predictor of AKI development early in the course of ICU stay.

**References**

1. Ninet S et al. J Crit Care 30:629–35, 2015

2. Kidney Intl. Suppl 2012; 2:1–138, 2012

## P513 Association of acute kidney injury defined by AKIN criteria with poor outcome in acute respiratory distress syndrome patients

### M Saleh, M Elghonemi

#### Kasr Alainy, Cairo University, Cairo, Egypt

**Introduction**

Few studies reported the deleterious association between Acute Respiratory Distress Syndrome (ARDS) and development of Acute Kidney Injury (AKI). We aimed to evaluate the association of AKI defined by Acute Kidney Injury Network (AKIN) criteria and poor outcome in ARDS patients.

**Methods**

Sixty four patients admitted with ARDS and mechanically ventilated were enrolled. In addition to routine labs and APACHE II score, lung injury severity scores (LISS) were calculated upon admission and after one week. Degree of renal impairment was assessed using AKIN criteria (grade 1, 2, 3). Patients were stratified into 2 groups according to their degree of renal impairment. Patients were followed during their hospital stay. All data were statistically analyzed.

**Results**

The mean age of the studied patients was 47.23 ± 10.12 years; 33 (51.6%) were males. Mean PO2/FIO2, and LISS on admission were 169.95 ± 31and 3.06 ± 0.54 respectively. In 27 (42.2%) of patients who had significant renal impairment, 15 (23.3%) and 12 (18.8%) patients had AKIN grade 2, and 3 respectively. In these patients, the follow up LISS and length of hospital stay were significantly higher compared to other patients: 3.33 ± 0.74 points, and 19.11 ± 6.37 days, and vs 2.84 ± 0.57, and 12.38 ± 4.21, with P value 0.004, and <0.001 respectively. Also, they had a higher need to use vasoactive agents; 21 (55.3%) patients vs 6 (23.1%) and spend more days on mechanical ventilation 14.18 ± 4.59 days vs 8.51 ± 3.77, with P value 0.019 and <0.001 respectively. Inpatient mortality was significantly higher in patients with significant renal impairment compared to others; 18 (66.7%) vs 6 (23.1%), P value 0.019.

**Conclusions**

AKI defined by AKIN criteria is associated with poor outcome in ARDS patients.

**References**

1- Seeley EJ. Updates in the management of acute lung injury: a focus on the overlap between AKI and ARDS: Adv Chroic Kidney Dis 2013 Jan; 20(1):14–20

2- Michael Darmon, Christophe Clec’h, Christophe Adrie, et al. Respiratory Distress Syndrome and Risk of AKI among Critically Ill Patients: Clin J Am Soc Nephrol 2014 Aug 7; 9(8): 1347–1353

## P514 The novel nitric-oxide donor pdno attenuates ischemia-reperfusion induced renal dysfunction

### KF Nilsson^1^, J Sandin^2^, L Gustafsson^2^, R Frithiof^3^

#### ^1^Örebro University Hospital, Örebro, Sweden; ^2^Karolinska Institutet, Stockholm, Sweden; ^3^Uppsala University, Uppsala, Sweden

**Introduction**

Renal ischemia-reperfusion injury is a common cause of acute kidney injury in intensive care and surgery. Besides the acute ischemic injury the reperfusion causes additional harm to the tissue, possibly by formation of free oxygen radicals and microcirculatory derangement. Recently novel mono-organic nitrites of 1, 2-propanediol (PDNO) were synthetized and shown to rapidly and controllable deploy NO in the circulation when administered intravenously. We hypothesized that intravenous infusion of PDNO during renal ischemia reperfusion would improve post-ischemic renal function and microcirculation.

**Methods**

Sixteen sheep were anesthetized, mechanically ventilated and surgically instrumented. The left renal artery was completely clamped for 90 min and the effects of ischemia and reperfusion was studied for a total of 8 hours. 15 min prior to the release of the clamp an intravenous infusion of PDNO (n = 8) or vehicle (1, 2 propanediol + inorganic nitrite, n = 8) was initiated (180 nmol/kg/min for 30 min, thereafter 60 nmol/kg/min for the remainder of the experiment).

**Results**

Renal artery blood flow, cortical and medullary perfusion, diuresis and creatinine clearance decreased in the left kidney post ischemia. However, in the sheep treated with PDNO urine production and creatinine clearance was significantly higher post ischemia compared to vehicle treated animals (1.7 ± 0.5 vs 0.7 ± 0.3 ml/kg/h, p = 0.04 and 7.5 ± 2.1 vs 1.7 ± 0.6 ml/min, p = 0.02, respectively). This was associated with an augmented improvement in left renal medullary perfusion and oxygen uptake in the PDNO-group (73.1 ± 9.2 vs 37.4 ± 4.9 percent of baseline, p = 0.004 and 2.6 ± 0.4 vs 1.6 ± 0.3 ml/min, p = 0.02, respectively. Mean arterial blood pressure was significantly reduced by PDNO (82.6 ± 2.6 vs 93.6 ± 2.8 mmHg, p = 0.008) but was still within normal limits. Importantly, total renal blood flow was not affected and there were no signs of increased blood methemoglobin concentrations.

**Conclusions**

The impairment in renal function and microcirculation caused by ischemia and reperfusion was blunted by intravenous infusion of the novel NO donor PDNO. This effect was achieved without severe systemic hypotension or a significant increase in methaemoglobinemia.

## P515 Association between fluid accumulation at initiation of acute renal replacement therapy and mortality in critically ill patients with acute kidney injury

### I Skorniakov^1^, A Varaksin^2^, D Vikulova^3^, O Shaikh^1^, C Whiteley^1^, M Ostermann^1^

#### ^1^Guy’s & St Thomas’ NHS Foundation Trust, London, United Kingdom; ^2^Institute of Industrial Ecology, Russian Academy of Sciences, Ekaterinburg, Russia; ^3^Sverdlovsk Regional Clinical Hospital 1, Ekaterinburg, Russia

**Introduction**

Our aim was to analyze the impact of cumulative fluid balance (CFB) estimated at the start of renal replacement therapy (RRT) and during treatment in critically ill patients with acute kidney injury (AKI) on outcome.

**Methods**

Retrospective analysis of patients who received RRT for AKI in a multidisciplinary University Hospital based Intensive Care Unit (ICU). Total CFB on the day of RRT initiation was estimated in ml and % of baseline body weight (BW).

Patients’ demographics, anthropometrics, laboratory data and Sequential Organ Failure Assessment (SOFA) scores were collected. Outcomes of interest were hospital mortality and length of stay in ICU.

**Results**

**Fig. 44 (abstract P515).**
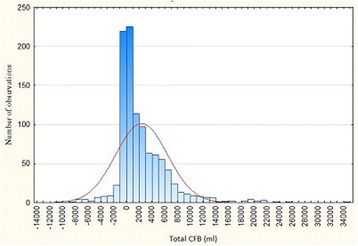
CFB on first day of RRT

1030 patients received acute RRT of whom 43.5% died in hospital. The median CFB on day of initiation of RRT was 1076ml (1.38% of BW). (Fig. 44)

Patients with CFB >10% BW had a significantly higher hospital mortality but were also sicker. (Table 21)

There was a significant association between CFB at initiation of RRT and mortality and between CFB on the last day of RRT and mortality.

**Conclusions**

In critically ill patients with AKI, CFB at initiation of RRT and at discontinuation of RRT was associated with hospital mortality.

**Table 21 (abstract P515).** See text for description

Parameters	CFB ≤10% BW	CFB >10% BW	p
Age in years, mean	62.8	63.8	0.55
SOFA score on 1st day of RRT, mean	10.1	11.5	0.0002
Days in ICU, mean	13.8	21.3	0.0001
Hospital mortality	0.42	0.54	0.025

Legend: Comparison between patients with CFB above and below 10% BW

## P516 Central venous catheter (cvc) as temporary vascular access in haemodialysis: indications, complications and follow-up

### G Di Lascio, L Anicetti, M Bonizzoli, G Fulceri, ML Migliaccio, P Sentina, M Cozzolino, A Peris

#### Careggi Teaching Hospital, Florence, Italy

**Introduction**

The use of central venous catheter (CVC) both as temporary and permanent haemodialysis access is increasing; nonetheless there are few recent studies comparing the outcome of long-term dialysis CVC (LT-CVC) and short-term dialysis CVC (ST-CVC). Consequently the aim of the present study is to analyze the side events and the complications related to ST-CVC and LT-CVC inserted in patients included in a haemodialysis program at our centre.

**Methods**

We analyzed retrospectively, based on a computerized prospectic system of data collection, personal and clinical data of a group of patients enrolled in a dialysis program, technical features of the catheters in use, unexpected events and catheter-related complications

**Results**

The outcome of 153 catheter were analysed (72 patients, 14.008 catheter-days, 34 long-term dialysis catheter, 119 short-term dialysis catheter). Removal for complications was less common with LT-CVC and jugular ST-CVC (IR respectively of 0.6 and 1.7 per 1000 catheter-days) than with femoral ST-CVC (IR of 12 per 1000 catheter-days, P < 0.001). Catheter-related bacteraemia (CRB) rates were 0.5 per 1000 catheter-days for LT-CVC, 1.4 for jugular ST-CVC and 4.0 for femoral ST-CVC. No significant differences were found between LT-CVC and jugular ST-CVC (Table 22). Complication-free survival of femoral ST-CVC was worse already within the first weeks (P < 0.001).

**Conclusions**

Based on our results, no significant difference was found in complications and unexpected events derived from the use of LT-CVC and jugular ST-CVC, while the dialysis femoral ST-CVCs show an high but predictable incidence of complications within the first weeks. Our findings point out that jugular ST-CVC could be used longer than expected but a new evaluation of catheterization techniques is still needed.

**Table 22 (abstract P516).** See text for description

Removal for:	Short-term Jugular CVC	Short-term Femoral CVC	Short term CVC tot	Long-term CVC	p
All complications (n)	5	36	41	5	<.001
CRB (n)	4	12	16	4	<.005
Dysfunction(n)	2	25	27	2	<.005

Legend Table 22:

## P517 Hyperlactatemia, lactate kinetics and a prediction of citrate accumulation in critically ill patients undergoing CVVHD with regional citrate anticoagulation

### D Khadzhynov, F Halleck, O Staeck, L Lehner, K Budde, T Slowinski

#### Dmytro Khadzhynov, Berlin, Germany

**Introduction**

Regional citrate anticoagulation (RCA) is recommended as standard anticoagulation mode for continuous renal replacement therapy (CRRT). A major complication of RCA-CRRT is systemic citrate accumulation. We studied the predictive capability of lactate concentrations and time-dependent lactate clearance for citrate accumulation.

**Methods**

We performed the retrospective, observational study at a university hospital with 6 ICUs. Included patients treated with RCA-CRRT were screened for metabolic signs of citrate accumulation during the first 48 hours of treatment.

**Results**

During the observational period of 3 years, a total of 1061 patients treated with RCA-CRRT were included in the analysis. Citrate accumulation during the first 48 hours of therapy was observed in 24 patients (2.26%). Patients were further stratified according to the initial lactate concentration before RCA-CVVHD: the incidence of citrate accumulation was 0.77%, 4.67% and 6.33% in patients with normal lactate, elevated lactate (≥2.2 mmol/L), and severe hyperlactatemia (≥4 mmol/L) respectively. ROC-AUC of initial lactate concentration for the prediction of citrate accumulation was 0.789 (95% CI 0.714–0.864, p < 0.001). Optimal cut-off from ROC (2.39 mmol/L) showed strong negative prediction (99.28%; 95% CI 98.33–99.77%), but weak positive prediction (5.21%; 95% CI 3.16–8.01%). The slope-intercept of lactate kinetic over 48h was positive and significantly higher in patients with citrate accumulation compared to those without (+0.2 vs. -0.006 mmol/L/h, p < 0.001). In the group of patients with initial severe hyperlactatemia (≥4 mmol/L) the median calculated lactate clearance at 6h, 12h and 18h was at each time-point positive and significantly higher compared to patients with citrate accumulation (24.0% vs. -9.8%, 48.1% vs. -20.5% and 59.4% vs. 2.3%, respectively; p < 0.001 for each time-point). The 12h lactate clearance showed the highest ROC-AUC value for citrate accumulation (0.839; 95% CI 0.751–0.927) with an optimal cut-off value of 24.3%.

**Conclusions**

The risk of citrate accumulation during RCA-CRRT is low even in cases with initially severe hyperlactatemia. Our study stress out the importance of lactate kinetics in assessing a risk of citrate accumulation.

## P518 Incidence of metabolic and electrolyte disturbances caused by decrease of filter clearance during regional citrate anticoagulated continuous veno-venous dialysis (RCA-CVVHD)

### T Slowinski ^1^, D Kindgen-Milles^2^, D Khadzhynov^1^

#### ^1^Charité Campus Mitte, Berlin, Germany; ^2^University Hospital Duesseldorf, Duesseldorf, Germany

**Introduction**

In all RCA protocols used for CRRT citrate is partially infused into patient and metabolized, thus leading to alkalization of blood and risk of metabolic alkalosis. When using trisodium citrate solution for RCA, protocol has to compensate for extra sodium, as well as for alkalization from metabolized citrate. In RCA-CVVHD using calcium-free, sodium-reduced, and bicarbonate-reduced dialysate (Ci-Ca Dialysate, Fresenius Medical Care) and calcium (Ca) substitution, filter clearance is essential to maintain bicarbonate (BIC) and electrolyte control. In consequence, loss of filter clearance may cause metabolic alkalosis, hypercalcemia, and hypernatremia resistant to per protocol adaptions. Aim of study: Evaluation of incidence of metabolic/electrolyte disturbances consistent with loss of filter clearance and outcome of disturbances after filter change.

**Methods**

During a 6 months period 191 consecutive patients were treated with RCA-CVVHD on 6 ICUs of a university hospital and prospectively observed for disturbances consistent with loss of filter clearance.

**Results**

From 191 patients, 13 (6.8%) showed at least one episode of metabolic alkalosis with hypernatremia and hypercalcemia, resistant to per protocol adaptations. None of the circuits gave pressure alarm. Filter was replaced after detection of disturbance. Median filter run-time until change was 63 h (range: 8 to 72). Median BIC showed significant increase at time of filter replacement (33.9 mmol/L; 95%CI 31.5–36.4; max 42.0), compared to values 48 hours (25.6 mmol/L; 95%CI 24.5–29.3) before replacement (p = 0.009). After filter change BIC decreased, reaching significant decrease at 24 h (27.9 mmol/L; 95%CI 27.0–30.7; p = 0.037). Ionized Ca also showed significant increase (1.22 mmol/L; 95%CI 1.18–1.31; max. 1.39) compared to 48 h (1.16 mmol/L; 95% CI 1.11–1.20) before replacement (p = 0.009). After filter change Ca decreased, reaching significant decrease after 16 h (1.11 mmol/L; 95%CI 1.05–1.15; p = 0.038). Blood sodium increased until filter replacement (148 mmol/L; 95%CI 145–149; max 154) compared to 48 h before change (144 mmol/L; 95% CI 142–146) without reaching significance (p = 0.140). After filter change sodium decreased to 141 mmol/L (95%CI 140-146) at 48 h (p = 0.305).

**Conclusions**

During RCA-CVVHD using calcium-free, sodium- and bicarbonate-reduced dialysate the incidence of metabolic and electrolyte disturbances consistent with reduced filter clearance is high and can occur after short filter run-time. Caution must be taken unless metabolic alkalosis can be severe and immediate filter replacement is mandatory to correct disturbances. Further analysis is necessary to understand causes of early reduced filter clearance.

## P519 Post-filter citrate concentration and ionized calcium in citrate CVVH

### N Huysmans^1^, M Vander Laenen^1^, A Helmschrodt^2^, W Boer ^1^

#### ^1^Ziekenhuis Oost Limburg, Genk, Belgium; ^2^Immundiagnostiek AG, Bensheim, Germany

**Introduction**

In citrate CVVH it has been established that a post-filter ionized Calcium (iCa) of below 1.4 mg/dL (0.35 mmol/L) is associated with a clinically significant and usable anticoagulant effect [1], promoting circuit survival. However, recent studies have demonstrated the limitations of measurements of low iCa (including high variance) on standard apparatus [2]. As part of larger prospective randomized study conducted in critically ill patients with AKI, treated with citrate CVVH, citrate concentrations were measured post-filter and compared to post-filter iCa values to establish a functional, clinically applicable threshold for anticoagulation, utilizing post-filter citrate concentration (PfC) as marker instead of iCa.

**Methods**

After written, informed consent, randomization took place to low dose citrate (2.5 mmol/L blood flow in the filter) or high dose citrate (4.5 mmol/L) as anti-coagulant, targeting a post-filter iCa of resp. 1.3-1.6 mg/dL (0.325-0.4 mmol/L) and 0.8-1.1 mg/dL (0.2-0.275 mmol/L). After adjusting blood flow for patient weight, settings on the Prismaflex® were adjusted to ensure the citrate concentrations in the filter by adjusting pre-filter citrate buffer rates (Prismocitrate 18/0®) and to correct for predilution. By adjusting buffer rates post-filter (Prismocal B22®) accordingly, a total clearance of 30 ml/kg/h was ensured. To limit the effects of citrate accumulation, values for PfC were measured 1 hour after initiation of CVVH and compared to iCa. The same blood gas analyser was used in all measurements (ABL800 radiometer®) for iCa.

**Results**

12 patients were included in the high citrate group, 12 in the low group. There were no differences between the 2 groups for gender, weight, age, or total and ionized calcium at initiation of CVVH. After 1 hour of CVVH, the median iCa in the high group was 1.13 mg/dL (IQR 0.35) vs.1.92 mg/dL (IQR 0.31) in the low group (p < 0.05). Median Pfc in high was 790 mg/L (IQR 89) vs. 512 mg/L (308) in low (p < 0.05). There was a strong inverse correlation between PfC and iCa (r = -0.641, p < 0.005).

**Conclusions**

The (inverse) correlation between PfC and iCa is robust. Based on the line of correlation plotted, a PfC value above 700 mg/L signifies clinically significant anticoagulation. Further commercialization of assays for measurement of citrate concentrations could lower costs and deem iCa measurement, with its recently described drawbacks, redundant. Further studies, utilizing PfC, could play a role in determining minimal citrate concentrations needed for circuit survival over longer periods and for the limits of citrate dosing before accumulation takes place.

**References**

1.Calatzis et al. Nephron 89:232–236, 2001.

2. Schwarzer et al. Critical Care 19:321–327, 2015

## P520 Carbon dioxide clearance is present in critically ill patients facing AKI and undergoing continuous renal replacement therapy: the hidden force of CRRT

### A Debain, J Jonckheer, W Moeyersons, K Van zwam, L Puis, K Staessens, PM Honoré, HD Spapen, E De Waele

#### UZ Brussel, Jette, Belgium

**Introduction**

Acute kidney injury (AKI) is of high prevalence in critically ill patients and is associated with an important morbidity and mortality (1). Continuous renal replacement therapy (CRRT) is a common modality in its treatment (2). Besides the primary aim to substitute impaired renal function, a secondary, unintentional phenomenon is carbon dioxide clearance. The purpose of this pilot study is to quantify the clearance of carbon dioxide by CRRT.

**Methods**

**Fig. 45 (abstract P520).**
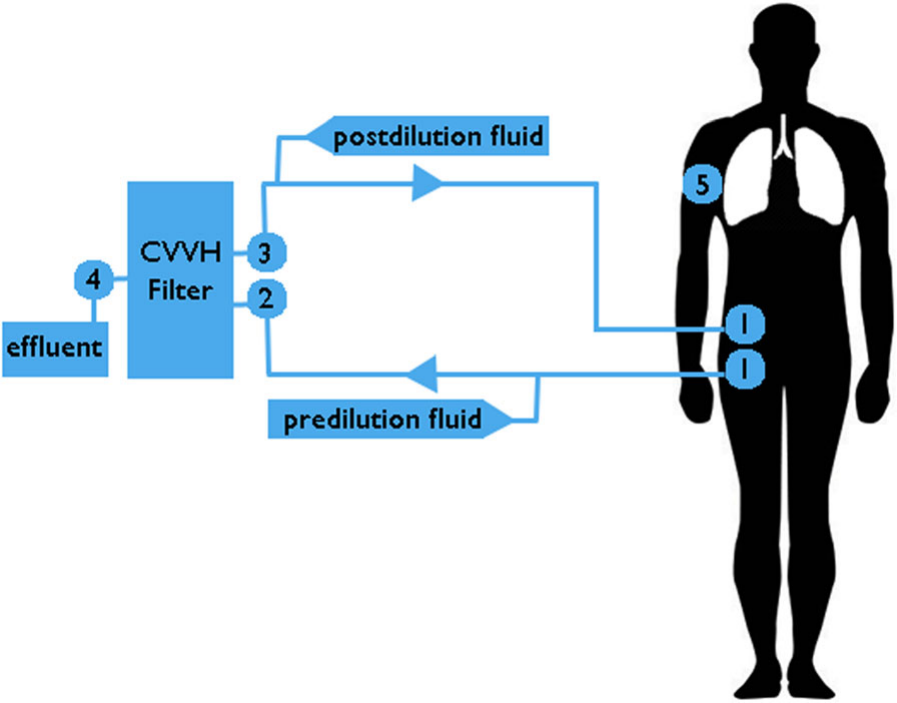
Critically ill patients facing AKI and undergoing CRRT

In a pilot setting of a hemodynamically en metabolic stable, critically ill patient with AKI and continuous venovenous hemofiltration (CVVH), on several points of the circuit (Fig. 45) blood sampling and analysis were performed. This during a standard CRRT run with and without citrate anticoagulation and without bicarbonate use in the predilution fluid. A software calculator was designed to quantify carbon dioxide removal. Necessary parameters are CVVH effluent flow, predilution and postdilution flow.

**Results**

Differences in pCO2 are not contributive as a quantitative measurement due to dilution of the blood passing through the circuit. In this context CO2 content was calculated using flow values. The overall removal of CO2 was 29ml/min or 70 mmol/h, an equivalent of 24% of CO2 content in a circuit with citrate. Without citrate we found a clearance of 27ml/min or 54 mmol/h equal to 19% of CO2 content removal.

**Conclusions**

CVVH for traditional ICU use had a hidden component which can be calculated: the extraction of CO2. This hidden force can rise to 24% of CO2 content.

**References**

1. Vincent JL et al. Contrib Nephrol 174:71–7. 2011

2. Renal Replacement Therapy Study Investigators. N Engl J Med. 361:1627–38.2009

## P521 Combined removal of 4.5 mol/day of protons and protein bound and water soluble substances in an ex vivo model for metabolic acidosis using an Advanced Organ Support (ADVOS) system based on albumin dialysis

### A Perez Ruiz de Garibay, B Ende-Schneider, C Schreiber, B Kreymann

#### Hepa Wash GmbH, Munich, Germany

**Introduction**

Metabolic acidosis is a common event among patients with multiple organ failure. When it is caused by an impaired carbohydrate metabolism due to hypoxia, lactic acidosis may occur increasing blood lactate and reducing pH. Our group has integrated the treatment of acidosis into an Advanced Organ Support (ADVOS) system based on albumin dialysis. It consists of 3 circuits that allow elimination of water and protein bound toxins, regeneration of the albumin used in the process and stabilization of pH [1]. The aim of this work is to show these features of the ADVOS system by means of lactate elimination, pH stabilization and bilirubin clearance in an ex vivo model for metabolic acidosis and comparing them with a normal renal dialysis machine (NIKKISO DBB-03).

**Methods**

An ex vivo model for metabolic acidosis with liver involvement was designed setting a continuous infusion of lactic acid into 5 liters porcine blood, which was spiked with bilirubin (275 mg/dl) before. Blood was dialyzed through the ADVOS system for 2 hours at 200 and 400 ml/min blood flow (BF). A dialysate pH of 9 was set. To determine the maximum lactate addition and removal, lactic acid infusion was progressively increased until blood pH was out of physiological ranges. Once the maximum addition was determined, tests were repeated with the NIKKISO machine using a BF of 400 ml/min. Lactate, pH and bilirubin levels were analyzed pre- and post-dialyzer every 15 minutes. Blood was checked for hemolysis at the beginning and the end of the experiments.

**Results**

The ADVOS system was able to stabilize the blood pH in a physiological range till a maximum lactic acid addition of 3.1 mmol/min (BF 400 ml/min). Moreover, with a BF of 200 ml/min up to 1.9 mmol/min (75%) of lactate was eliminated, which would result into a proton elimination of 4,464 mmol/day. Additionally, the ADVOS system removed significantly more bilirubin from blood as the NIKKISO DBB-03 machine (66% vs. 21%). Although the NIKKISO DBB-03 machine achieved a higher lactate elimination rate of 80%, blood pH decreased to 6.90.

**Conclusions**

During a continuous infusion of up to 3.1 mmol/min of lactic acid in an ex vivo model for metabolic acidosis, blood pH decreased to 6.90 under conventional hemodialysis. With the ADVOS system, blood pH remained stable between 7.35 and 7.45 and additionally an efficient elimination of bilirubin was achieved. Hence, ADVOS has a potential advantage for the treatment of critically ill patients with multiple organ failure.

**References**

1. Henschel B et al. Crit Care 19 (Suppl 1):P383, 2015.

## P522 Lung ultrasound during continuous renal replacement therapy

### A Bini^1^, E Votino^1^, G Giuliano^1^, I Steinberg^1^, L Vetrugno^2^, D Trunfio^1^, A Sidoti^1^, E Brogi^1^, F Forfori^1^

#### ^1^University of Pisa, Pisa, Italy; ^2^University of Udine, Udine, Italy

**Introduction**

Ultrasonography of inferior vena cava (IVC) is a guide to estimate central venous pressure [1]. Lung ultrasound is an effective method of evaluating extravascular lung water (EVLW) [2]. The observation of B-lines resolution and the collapse index of IVC represent a valid guide to improve continuous renal replacement therapy (CRRT) management. The aim of this work was to demonstrate the role of chest and IVC ultrasound in the assessment of the variation of fluid status in patients undergoing CRRT.

**Methods**

We enrolled patients undergoing CRRT during their ICU stay. Patients were assessed for lung and IVC ultrasound immediately before CRRT (T0) and after 12 (T12), and 24 (T24) hours. B-lines were counted and the reduction of IVC diameter was measured using the collapse index. We also recorded for each patient at T0 and T24: PaO2/FiO2, Leukocytes (WBC), Procalcitonin (PCT), and the volume of fluid removed. We obtained written informed consent from the patients.

**Results**

**Fig. 46 (abstract P522).**
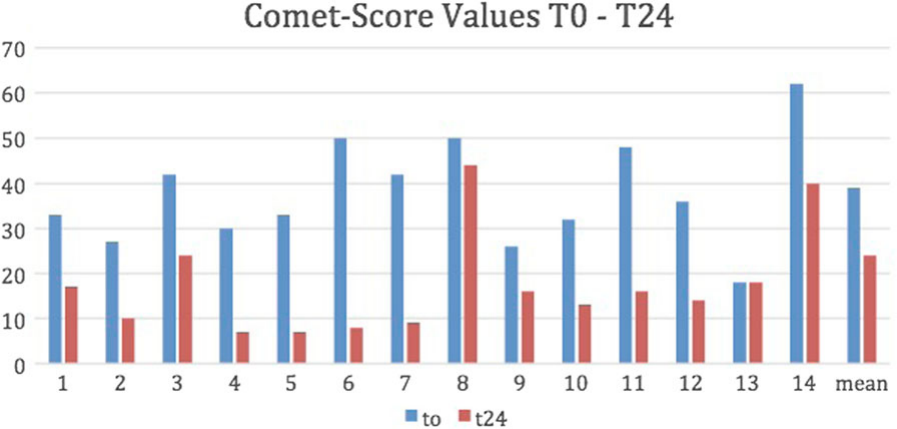
Comet-Score during 24 hours

A total of 14 patients undergoing CRRT were recruited. PaO2/FiO2 ratio, PCT and WBC count improved in all patients from T0 to T24. B-lines resolution was also reached in all patients as demonstrated by a Comet-Score. (Fig. 46)

Not all patients were evaluated for IVC ultrasound, because of open abdominal surgery. Moreover, one of them suffered from a severe tricuspidal valve failure, making it the measure not reliable. Therefore, we measured the IVC collapsibility index (Δ ø IVC %) in 10 patients; the collapsibility index did not significantly change before and after CRRT.

**Conclusions**

We found that fluid removal during CRRT can be clearly monitorized by chest ultrasound. Lung chest ultrasound is a non-invasive, simple method performed at the bedside that provides accurate information on EVLW variations and, consequently, very useful for an appropriate management during CRRT.

**References**

1. Vitturi N et al. Lung ultrasound during hemodialysis: the role in the assessment of volume status. Intern Urol Nephrol. 2014; 46(1): 169–74

2. Soldati G et al. Can lung comets be counted as “objects”? JACC Card Imaging. 2011; 4(4): 438–9

## P523 Survival and restoration of renal function among CRRT patients in an academic centre ICU

### M Conroy, B Marsh, J O’Flynn

#### Mater Misericordiae University Hospital, Dublin, Ireland

**Introduction**

Continuous renal replacement therapy (CRRT) is a common necessity in ICUs, whether for those with end-stage renal disease (ESRD) or those with acute kidney injury (AKI). Mortality is high in both situations, with reported rates above 50% in centres of excellence [1, 2]. Recovery of renal function among survivors of an acute insult is usually good [1] but some suffer long-term renal impairment as a consequence. Our aim was to examine short- and long-term mortality and recovery of renal function among patients requiring CRRT in ICU. Our goal was that findings from this study would support evidence-based decision making among intensivists when faced with critical illness and the potential need for CRRT.

**Methods**

All patients admitted to our centre’s ICU between January 1st 2012 and December 31st 2014, who required CRRT, were included in the study.

Information gathering was from the patient data on these records and public death records.

**Results**

Our cohort was of 450 patients, with a median APACHE II score on admission of 27.8. Overall in-hospital mortality among patients requiring CRRT was 46% (n = 208). Mortality in ICU was 37%. The in-hospital mortality rate in medical patients was 52%, and the rate was higher in AKI patients (48.5%) than in ESRD patients (31.7%). At one year, mortality for the total population was 54%. Outcomes for ESRD patients at one year are poorer than in the short term, with 17% of ESRD patients who survived to discharge dying within the next year, compared to 14% of AKI patients. Regarding renal function, we found a dialysis-dependency of 34% among survivors to ICU discharge; when those with known ESRD were excluded, the rate was 21%. Among those with AKI who survived to hospital discharge, the dialysis-dependency rate was 7%.

**Conclusions**

Our data show a varying pattern of concordance with previous work. Overall mortality is lower both in the short- and long-term than in other countries [1, 3], but this may reflect differences in study populations and pre-admission medical status, rather than truly different outcomes. In those with ESRD, our work shows that although they may be considered high-risk patients for ICU, their outcomes are equal and often superior to AKI patients; this may influence intensivists to more readily accept these patients to a higher level of care. Finally, regarding recovery of renal function, our analysis suggests that the risk of lasting renal failure in survivors of acute insults requiring CRRT, is low.

**References**

1. Allegretti AS et al. Crit Care. 20;17(3):R109. 2013.

2. Silvester W et al. Crit Care Med. 29(10):1910–5. 2001.

3. Laupland KB et al. Chest. 129(4):954–9. 2006.

